# New pyrimidine-N-β-D-glucosides: synthesis, biological evaluation, and molecular docking investigations

**DOI:** 10.55730/1300-0527.3553

**Published:** 2023-02-28

**Authors:** Nuran KAHRİMAN, Kıvanç PEKER, Vildan SERDAROĞLU, Ali AYDIN, Burçin TÜRKMENOĞLU, Asu USTA, Nurettin YAYLI

**Affiliations:** 1 Department of Chemistry, Faculty of Science, Karadeniz Technical University, Trabzon, Turkey; 2 Department of Basic Medical Science, Faculty of Medicine Bozok University, Yozgat, Turkey; 3 Department of Analytical Chemistry, Faculty of Pharmacy, Erzincan Binali Yıldırım University, Erzincan, Turkey; 4 Department of Chemistry, Faculty of Arts and Sciences, Recep Tayyip Erdoğan University, Rize, Turkey; 5 Faculty of Pharmacy, Karadeniz Technical University, Trabzon, Turkey

**Keywords:** 2,4,6-trisubstituted pyrimidine, *N*-β-D-glucosides, biological activity, molecular docking

## Abstract

In this study, syntheses of new pyrimidine-coupled *N*-β-glucosides and tetra-*O*-acetyl derivatives were carried out. All glycoconjugates were investigated in comparison with known chemotherapeutic agents in terms of their antimicrobial and anticancer functions and DNA/protein binding affinities. Spectral data showed that all glycoside derivatives were obtained by diastereoselectivity as β-anomers. Both tested groups exhibited strong antiproliferative activity (2.29–66.84 μg/mL), but some of them had sufficiently ideal % cytotoxicity values (10.01%–16.78%). And also all synthetic compounds exhibited remarkable antibacterial activity against human pathogenic bacteria. Binding of these compounds to CT-DNA resulted in significant changes in spectral properties, consistent with groove binding. Molecular docking studies of some compounds revealed that the docking score, complex energy, and MM-GBSA ΔG_Bind_ energy values were consistent with the experimental results, which showed that the new compounds were potent chemotherapeutic agents. Overall bioactivity results suggest that these compounds may be candidates as new chemotherapeutic agents and deserve further pharmacological evaluation.

## 1. Introduction

Cancer is described as the uncontrolled growth and spread of abnormal cells and has long been recognized as one of the most common fatal diseases of modern times. Cancer and cancer-related diseases have recently become one of the leading causes of death, right after traffic accidents and common injuries [1–2]. Chemotherapy plays a crucial role in cancer treatment, but some of the agents used in chemotherapy cause numerous side effects due to their cytotoxic and mutagenic effects on healthy cells [1–2]. The emergence of multidrug resistance (MDR) has rendered most of the existing chemotherapeutic agents ineffective [3]. Consequently, it is crucial to develop alternative drugs that have no or insignificant side effects on the human body and are not resistant to multiple drugs.

Multidrug-resistant bacteria have recently caused life-threatening infections around the world and continue to spread. The spread of antibiotic resistance has become a critical problem in the treatment of infections. Therefore, the synthesis of a novel and effective antibacterial agent is essential to combat drug resistance [4]. Moreover, some significant experimental results have shown the strong association between pathogenic microbial flora and certain diseases such as stomach, oropharyngeal, liver, urogenital, cervical cancer, and lymph node disorder [5]. Such pathogenic microbial flora has been shown to frequently cause chronic inflammation due to its toxic microbial metabolites, which may lead to an increased risk of cancer or the development of cancer-related diseases [6–7].

Dual-acting molecules with anticancer and antibacterial properties could be a good alternative to treat both cancer and inflammation. Practically, this could allow physicians to effectively treat both cancer and inflammation, provided that intelligent dose adjustment is made [8].

Carbohydrates allow almost limitless structural variation among the major classes of biomolecules, and a wide variety of combined compounds can be formed by coupling several monosaccharides with different stereochemistry [9]. The structural differences of carbohydrates enable them to be considered “exclusive” molecules and contribute to their diverse biological properties [9]. Due to their rigid structure with a high degree of functionalization and the presence of several adjacent stereogenic centers, they are also frequently used as chiral templates for the introduction of chirality in synthetic asymmetric methods [10].

Due to the role that carbohydrates play in biological processes such as immune responses, adhesion, inflammation, and cell growth [11], the chemistry and glycobiology of glycoconjugates have attracted enormous attention and gained importance in recent years [12]. Among these structures, heterocyclic coupled glycosides are essential and reliable for the improvement of many anticancer and antitumor drugs [11], and have been accepted as good glycosyl donors, in addition to their biological properties such as enzyme inhibitory activity [11]. The toxic effect of 5-fluorouracil, which is used as a common drug in cancer therapy, on mammalian cells could be reduced by synthesizing *N*–or *O*-galactopyranoside derivatives and the inhibitory effect of this drug on cancer cells could be improved [11]. Glycosylation also improves the pharmacological properties and bioavailability of compounds by contributing to the water solubility and stability of organic molecules. In addition, glycoside derivatives of physiologically active compounds such as vitamin glycosides have been reported to be useful antiallergic agents [3].

In the literature, there are many synthetic glycosides with remarkable properties. Helicid, for example, is a natural compound isolated from the fruit of *Helicia nilagirica* Beed and is used for its sedative and sleep-inducing effects in the treatment of insomnia. However, its disadvantages such as slow action and low biological utilization led to the synthesis of pyrimidine derivatives of helicid to improve its biological activities. And it was reported that all compounds showed good sedative effect [13]. In addition, chalcone-*O*-glucosides [14] and pyrazole-linked glucosides are some of the other glycoside compounds that have been reported by researchers in the literature [11]. In another study, carbohydrate conjugates of desciclovir were synthesized by Chamberlain et al. as potential prodrugs of aciclovir (a specific inhibitor of herpesvirus replication) to provide enhanced activity [15].

Although more studies have been done on *C*–and *O-*glycosides, it is known that the carbohydrates in the cell wall are bound to the proteins through the nitrogen atom of asparagine [9]. And these *N*-glycoproteins play an elementary role in biological systems through their functions in viral replication, cell growth and recognition, and immune response. The nucleoside adenosine is a good example of *N*-arylglycosides and one of the essential components of RNA. There are many marketed nucleoside analogs used as antiviral and anticancer drugs, which suggests that *N-*aryl glycosides may be regarded as drug candidates [9]. *N-*glycosides not only serve as essential components of DNA/RNA, but also as clinical drugs for tumor treatment, immunoregulation, and antiviral therapy [16]. Glycopeptides and glycoproteins are a common category of *N*-glycosides that often function as hormones, antibodies, and enzymes in living systems. Many N-glycosides have also been found as pharmaceutical molecules and natural products and have shown various biological properties such as glycogen phosphorylase and galactosidase inhibitors [16].

In recent years, the concept of hybrid drugs has gained attention, in which two or more bioactive pharmacophores are combined to show synergistic effect [17]. Using this approach, several researchers have recently reported numerous hybrid compounds in which medically privileged heterocycles are coupled with biologically important pharmacophores [11, 17].

Therefore, the efficient synthesis of heterocycle-coupled glucosides/arylglucosides is very valuable and plays an important role at the forefront of organic chemistry [18–22]. Arylglucosides can be obtained by linking a carbohydrate to an aromatic aglycone via *C*-, *O*-, and *N*-glycosidic linkages to give *C*-, *O*-, and *N*-aryl glycosides.

Due to the biological importance and limitations in the synthesis methods, there is an urgent need for the synthesis of *N*-glycosides. Since pyrimidine motifs are widely used heterocyclic structures found in natural products, functional materials, biological systems, and agrochemicals with pharmaceutical and chemical properties [23–24], in this study, the pyrimidine ring was combined with glucose to obtain sugar-modified heterocyclic derivatives.

We report here a versatile, operationally simple synthetic route for the synthesis of pyrimidine *N*-β-D-glucosides and evaluate their potent anticancer and antibacterial activities as well as their DNA/BSA binding affinities. In addition to these, molecular docking studies of some active compounds (**4**, **7**, **13**, and **16**) were also carried out with in silico approaches.

## 2. Results and discussion

### 2.1. Chemistry

The syntheses of the pyrimidine *N*-glucosides (**1–9**) and their tetra-*O*-acetyl derivatives (**10–18**) were carried out in this study. The detailed synthetic routes for the compounds **1–18** are shown in Scheme 1 [15]. The purity of all structures was checked by thin-layer chromatography (TLC), and when necessary, they were purified by column chromatography and their structures were determined spectroscopically by using NMR (^1^H, ^13^C, APT, COSY, ACD-NMR), FT-IR, LC-MS/MS spectral methods and elemental analysis.

**Figure 1:**
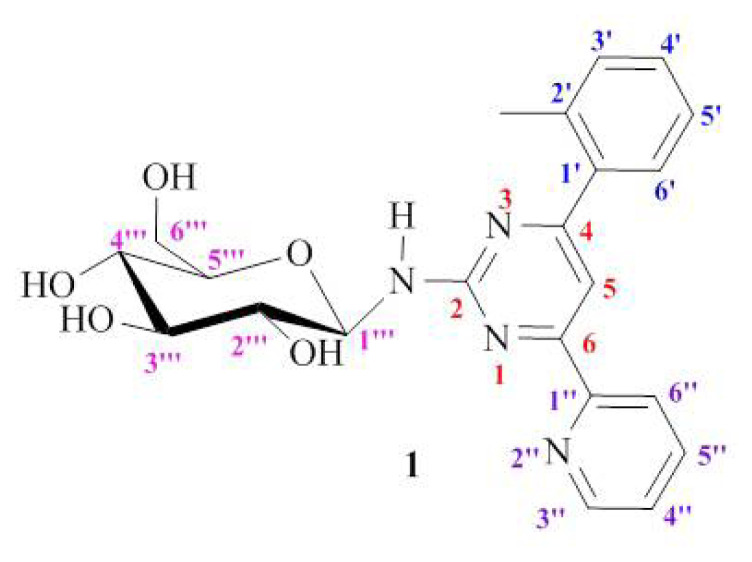
Numbering of atoms on compounds **1** and **10**.

The numbering of atoms used in spectroscopic analysis of compounds (**1–18**) was given in Figure 1 for compound **1** as an example.

In the first part, new pyrimidine-coupled *N*-β-glycosides (**1–9**) were synthesized by the condensation between 2-amino-4,6-diarylsubstituted pyrimidines and D-(+)-glucose monohydrate under acidic conditions [9, 15]. The FT-IR spectrum of compounds **1–9** showed the characteristic broad absorption bands for both –OH and –NH groups between 3400 and 3100 cm^−1^. These bands appeared due to the binding of glucopyranosyl group to pyrimidine core. LC-MS/MS spectra of **1–9** gave a pseudomolecular ion peak at *m/z* 425.00 [M + 1]^+^.

The ^1^H NMR spectrum of compounds **1–9** showed 13 proton signals for the aglycone moiety (2-amino-4,6- diarylsubstituted pyrimidines) and seven protons at δ 3.7–5.5 ppm for the glycone part of **1–9**. The ^1^H-NMR spectrum showed one anomeric proton signal assignable to H-1‴ of glucosyl moiety at δ ~ 5.4 (d, *J* = 8.8–9.2 Hz). The ^13^C-NMR spectrum of compounds **1–9** gave one anomeric carbon signal detected at δ ~ 82.5 (C-1‴) bound to the anomeric proton of **1–9**. The coupling constant (8.8–9.2 Hz) of the anomeric proton indicated a β-glucopyranosyl unit which were in good agreement with the literature [11,12,25–26]. All the compounds obtained adopted the β-configuration. This is because for pyranoses, the usual energetically preferred form is the equatorial orientation of the substituents [27]. The other H-atoms of the glucopyranosyl (H-2‴-H-5‴) formed a coupling system and the multiplet peak arising at 3.4–3.6 ppm was defined as the H-2‴-H-5‴ protons. Due to the different chemical environment, the −CH_2_ protons (H-6a‴/6b‴) exhibited different chemical shifts. The H-6a‴ was observed as a doublet (d, *J* = 11.8 Hz) or doublet of doublets (dd, *J* = 11.8/2.2 Hz) at δ 3.9 ppm. The peak belonging to H-6b‴ appeared as dd at 3.7 ppm with coupling constant *J* = 11.8–12.0/4.0–5.2 Hz. On the other hand, due to the dissolution of almost all *N-β*-D-glucopyranoside derivatives in methanol and the preparation of NMR samples in deuteromethanol (CD_3_OD), the −NH and −OH protons of the glycopyranosyl ring were exposed to deuterium exchange in the solvent and their peaks could not be observed in the ^1^H-NMR spectra. Preliminary assignments of ^13^C NMR data were made using Attached Proton Test (APT) experiments. ^13^C NMR spectra gave the peaks at δ 166.9–103.0 ppm for aglycone parts and peaks at δ 82.5–62.0 ppm appeared for glycone unit of compounds **1–9**, respectively.

In the second part of the study, compounds **1–9** were acetylated then tetra-*O*-acetyl derivatives (**10–18**) were obtained in high yields (78%–93%) as a result of the nucleophilic acyl substitution of **1–9** with acetic anhydride in the basic medium [28]. The FT-IR spectrum of compounds **10–18** showed the characteristic broad absorption bands for the −NH and C=O groups at 3420 cm^−1^ and 1740 cm^−1^, respectively. LC-MS/MS spectra of **10–18** gave a pseudomolecular ion peak at *m/z* 615.00 [M + Na]^+^. In ^1^H-NMR spectrum, as a result of the deshielding effect of the C=O or *O*-acetylated glucopyranosyl group by the electron-withdrawing property, the anomeric H-1‴ appeared as a doublet in the low field (δ 6.1–5.9 ppm) with a coupling constant between 9.0 and 10 Hz. The coupling constant also confirmed the β-glucopyranosyl linkage. The acetyl groups’ H-atoms of compounds **10–18** resonated in the range of 2.0–1.9 ppm in ^1^H-NMR spectra, and the ^13^C-NMR signals were observed around 21.6–19.2 ppm. In addition, four new quaternary peaks were observed in the range of 171.1–169.6 ppm, originating from the carbonyl groups and confirming the structure. All the spectra of **1–18** were given in the supplementary data.

### 2.2. Biological evaluation

#### 2.2.1. Evaluation of anticancer properties of the compounds

It is widely known that natural or synthetic glycosides play many important roles in living systems, such as apoptotic or arrhythmic effects. As the findings imply that glycosides are cytotoxic, glycosides can also be attributed to promising drug candidates that exhibit significant anticancer activity. Therefore, the synthesis and biological evaluation of novel glycosides are of interest to our research group. Accordingly, the anticancer effects of 18 new compounds synthesized as pyrimidinecoupled *N*-β-glucosides (**1–9**) and their tetra-*O*-acetyl derivatives (**10–18**) were evaluated using the MTT protocol. The half-maximal inhibitory concentration 50 (IC_50_) inhibition values, generally used for inhibition studies, and growth inhibiting concentration 50 (GI_50_), total growth inhibition (TGI), and lethal concentration 50 (LC_50_) were determined using spectrophotometric data obtained from the MTT assay using cisplatin and 5-fluorouracil (5 FU) as anticancer control drugs, as recommended by the NCI. When the synthetic compounds were compared with the control group (Tables 1 and 2), they were not sufficiently antiproliferative in the A549 cancer cell line, even at a high concentration of 500 μg/mL. Among the pyrimidine *N*-β-D-glucosides (**1–9**), compounds **1–5**, **7**, and **8** in C6 cell line (with IC_50_ values between 27.78 and 49.71 μg/mL, TGI values between 20.46 and 31.57 μg/mL) and compounds **1**, **4**, **7**, and **8** in the C6 and HeLa cell lines (with IC_50_ values between 31.85 and 81.26 μg/mL, TGI values between 21.60 and 50.07 μg/mL) exhibited high antiproliferative properties (Tables 1 and 2). Compounds **5** and **16** in MCF7 and Hep3B cells, and **4** in Hep3B cells showed potent anticancer properties with IC_50_ values between 4.12 and 66.84 μg/mL and TGI values between 4.29 and 75.66 μg/mL. In the HT29 cell line, compounds **3–5**, **7**, and **8** achieved sufficient antiproliferative activity (IC_50_ values between 26.69 and 78.38 μg/mL, TGI values between 31.94 and 230.10 μg/mL). When the effect of the pyrimidine-coupled pyrimidine *N*-β-D-glucosides on cells was investigated according to the screening method recommended by the NCI, compound **5** with low GI_50_ and high LC_50_ values could be considered a candidate for further pharmacological testing (Tables 1 and 2).

When the IC_50_ and TGI data of the test results were examined, the tetra-*O*-acetyl-*N*-β-D-glucopyranoside derivatives (**10–13, 15–16, 18**), with the exception of **14** and **17**, showed similar or stronger antiproliferative properties than 5-fluorouracil (5FU) and cisplatin (Table 2). When the GI_50_ values presented in Table 1 were examined, the tetra-*O*-acetyl derivatives showed greater growth inhibition than cisplatin, and all compounds also caused greater inhibition than 5FU in the C6 cell line. Of the tetra-*O*-acetyl derivatives (**10–18**) in Tables 1 and 2, compound **16** showed very successful anticancer properties against the C6 cell line as well as against HeLa, HT29, MCF7, and Hep3B cells (IC_50_ values between 3.26 and 9.13 μg/mL and TGI values ranging from 2.80 to 7.33 μg/mL). Among the other antiproliferative compounds, compounds **13** and **18** against HeLa cells and compound **10** against HT29 cell line showed potent anticancer activity. Considering the obtained growth inhibition values (GI_50_ and TGI values), it could be said that all compounds except **14** and **17** have the potential to be used for pharmacological studies for the treatment of glioma cancer in the brain. While compounds **4**, **7**, **13**, **15**, **16**, and **18** showed lower values than cisplatin in terms of lethal concentration (LC_50_), compounds **1–3**, **5**, **8**, and **10–12** showed higher LC_50_ values than both cisplatin and 5FU (Tables 1 and 2). Higher lethal concentration values indicate that the cytotoxic effects of the test substances are lower, which is desirable. Lower GI_50_ and TGI concentration values indicate that the cytotoxic effects of the test substances are greater, which is also desirable. When the IC_50_ data of the test results were examined, only compounds **13**, **16**, and **18** were found to be effective for the HeLa cell line. On the other hand, the GI_50_ and LC_50_ values of these compounds showed that all compounds were more effective than the control compounds 5FU and cisplatin. However, the TGI values showed that these compounds were toxic to the HeLa cell line except for compounds **16** and **18**. In this case, these compounds cannot be used for further studies because they do not have the molecular structure that can be used in cervical cancer research. However, the design of these molecules can be redesigned with Lipinski’s rules in mind, and the toxic effects can be reduced to reasonable levels without reducing the antiproliferative effects. However, it should be kept in mind that the MTT method only measures mitochondrial activity of living cells and FL cells may have lower mitochondrial activity than cancer cells. According to this principle, the cells of FL could have lower mitochondrial activity, which could lead to an increased antiproliferative effect. To address this issue, we used LDH-based cytotoxicity measurement in addition to MTT. In evaluating the results of LDH activity measurement, which is our second important test to elucidate the cytotoxicity of these compounds, it was found that the above anticancer compounds (**1–3, 5, 7, 8, 10–13, 15–16**, and **18**) caused membrane damage of approximately 10%–20% at effective concentrations (IC_50_ values in the range of approximately 25–50 μg/mL) (Tables 3 and 4).

These values were very close to the values of percent cytotoxicity caused by the positive controls used (5FU and cisplatin) (Table 3 and 4).

However, the membrane damage caused by these compounds with potent anticancer properties should have more pharmacologically reliable values without altering the anticancer properties of these molecules. According to the proliferation measurements performed on the normal cell line (FL), compounds **1**, **4**, **5**, **13**, **16**, and **18**, which had high LC_50_ values (>500 μg/mL), were also found to cause membrane damage of 15%–20% in toxicity tests with the same molecules. The results of these pharmacological measurements on the normal cell line FL are largely consistent with NCI criteria. Therefore, they may be candidates for ADME/Tox and advanced phase studies due to the significant in vitro biological activity exhibited by the respective test compounds.

When the qualitative structure-activity relationship of the compounds was discussed, it was seen that the 2′–and 3′-pyridinyl compounds (**4**, **5**, **7**, and **8**) in which the methyl group was in the meta and para positions were both anticancer and antimicrobial active (Table 5). However, acetylated derivatives of these compounds (**13**, **14**, **16**, and **17**) showed inactive behavior against cells (Table 6). In general, the position of methyl and pyridine nitrogen affected the biological activities, while acetylation resulted in different cytotoxicities. The decrease in hydrogen bonding capacity by acetylation may have caused this behavior. As can be understood from docking studies, the three-dimensional structure of molecules regulates their interactions with biomolecules. Therefore, the movement of the methyl group and nitrogen atom and acetylation caused the bioactivity of the compound to change.

#### 2.2.2. Morphological changes of molecules on cells

**Figure 2:**
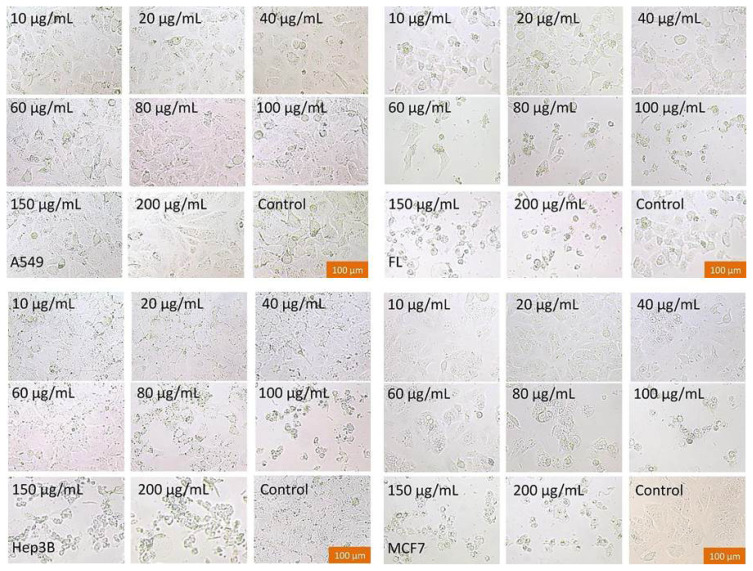
Effect of **5** on the morphologies of A549, FL, Hep3B, and MCF7 cell lines. Exponentially growing cells were incubated overnight with various concentrations of **5** at 37 °C. Control cells were treated with only DMSO. All measurements were 100 μm.

At various concentrations, the effects of pyrimidine-coupled *N*-β-glucosides and tetra-O-acetyl derivatives on the cell morphology of C6, Hela, A549, Hep3B, MCF7, and FL were visualized and examined by phase-contrast microscopy. The control cells shown in Figure 2 exhibited fibroblast–or epithelium-like normal cell morphology and served as the benchmark for our assessments. The first impression we got from the phase-contrast microscopy images was that the cells began to detach from the flask surface in a concentration-dependent manner. During this detachment, the cells lost their fibroblastic or epithelial normal shape and started to change into round shapes. Then the cells underwent some morphological changes, such as cytoplasmic blistering and spiking, abnormal spherical structures, and apoptotic bodies, and finally the cells floated (indicating that the cells were dead). As we found, concentrations of 60 μg/mL and above caused cells to separate, to be smaller, to be seen in smaller numbers, and to have insufficient cell adhesion. Furthermore, these images may indicate a decrease in cell viability leading to poor proliferation, small cell size, and apoptosis. We observed that the cells could maintain their normal fibroblast-like appearance even under the conditions of the test substance at low concentrations (< 40 μg/mL). We also found that apoptosis-like images as well as partial necrotic damage affected the growth of cells treated with high concentrations of the compounds (>82 μg/mL) (Figure 2).

#### 2.2.3. Evaluation of Antibacterial Effects of the Compounds

Indeed, the development not only of new anticancer drugs but also of antibiotic derivatives is of paramount importance to the community. This is because the bacterium methicillin-resistant *Staphylococcus aureus* (MRSA) has been reported to cause more deaths than HIV/AIDS annually worldwide, particularly in the United States [29]. In light of this literature information, the antibacterial activity of the newly synthesized compounds on some gram-positive and gram-negative bacteria that cause disease in the human body was investigated using the minimum inhibitory concentration (MIC) method. Among our test compounds, those with MIC values not exceeding 125 μg/mL and below these dose levels were found to have antibacterial activity. This evaluation was based on the MIC values of antimicrobial drugs in use today. When the MIC values of the newly synthesized compounds were examined, it was found that the antibacterial activity of the compounds was quite high (**10** for *E. faecalis* VRE ATCC 19433 (125 μg/mL), **11** and **13–18** for *E. faecalis* ATCC 29212 (31.25–125 μg/mL), **2**, **5**, and **8** for *S. aureus* ATCC 25923 (15.62–125 μg/mL), **2**, **5**, **6**, **8**, **9**, **11**, **13**, **14**, and **17** for *S. aureus* MSSA ATCC 29213 (62.5–125 μg/mL), **2** and **8** in *S. aureus* MRSA ATCC 46300 (125 –< 7.81 μg/mL each), **1**, **4**, **7**, and **12–18** in *S. mutans* ATCC 35668 (in the range of 62.5–125 μg/mL) and **5** in *S. gordonii* NCTC 7870 (125 μg/mL)) and these compounds were at least as sensitive as the positive control SCF antibiotic against the tested bacteria (Tables 7 and 8).

However, none of the new compounds showed strong antibacterial activity on *E. coli* ATCC 25922, *E. coli* ESBL ATCC 35218, and *P. aeruginosa* AGME ATCC 27853 strains. When the results of the in vitro antibacterial assays of these structures were evaluated as a whole, it was found that the pyrimidine *N-β*-D-glucosides (**1–9**) and the tetra-*O*-acetyl derivatives (**10–18**) exhibited similar antibacterial properties. Compound **8** showed the best result with a strong antimicrobial effect on *S. aureus* MRSA ATCC 46300 (<7.81 μg/mL). Although both groups of compounds were more active on gram (+) bacteria, their activities were much lower on gram (−) bacteria. In addition, potent antimicrobial activity against resistant strains such as VRE, MRSA, ESBL, and AGME was not achieved at the desired level and remained at the same level as the control drug, SCF. In general, both functional groups were found to be effective against some bacteria causing diseases in the human body, and we strongly recommend that some of them should enter advanced pharmacological research immediately and others after remodeling according to Lipinski’s rules.

#### 2.2.4. Analyzing of DNA/BSA binding properties of the compounds

The vast majority of anticancer agents used in pharmaceutical chemistry act on functional macromolecules such as DNA and proteins. Given this well-known fact, the relationship between newly developed anticancer drug candidates and macromolecules should be studied in detail. The interaction of molecules with DNA leads to significant changes in the helical structure of DNA, which can be observed by spectrophotometric techniques. In general, the changes in DNA caused by the compounds appear as hyperchromic or hypochromic effects in their absorption spectra [30]. The hypochromic effect shows a decrease in absorbance when the DNA concentration is increased, while the hyperchromic effect shows an increase in absorbance when the DNA concentration is increased. While the hypochromic effect causes changes in DNA structure and shrinkage or shortening of DNA along the helical axis, the hyperchromic effect causes the twisting of DNA in the helical configuration of DNA. Also, the red or blue shift in the absorption bands of the compounds can be an indication of the stability of the compound and the DNA structure. The DNA/BSA binding properties of the new chemicals synthesized by our group were determined using a UV-Vis spectrophotometer. The interactions of these compounds with DNA were as follows. In the spectra obtained by UV-Vis studies of the compounds, a single maximum absorption peak was observed and this peak had no clear bathochromic or hypsochromic effect. When increasing amounts of CT-DNA were added to the reaction mixture, the decrease in the absorption intensities of **3** and **9** of pyrimidine-*N-β*-D-glucopyranosylamines resulted in a hyperchromic effect, whereas the increase in the absorption intensities of compounds **1**, **2**, and **4–8** resulted in a hypochromic effect. Similarly, the addition of CT-DNA in increasing amounts led to an increase in the absorption intensities of compounds **10–15**, **17**, and **18** (hyperchromic effect), while the absorption intensity of compound **16** decreased (hypochromic effect). The physical interactions of our newly synthesized compounds with BSA caused the formation of the spectral bands described below. According to the spectrophotometric analysis of the interaction of pyrimidine-coupled *N-β*-glucosides with increasing amounts of BSA, compounds **1–3**, **5**, **7**, and **8** showed a hypochromic effect, while compounds **4**, **6**, and **9** from the same group exhibited hyperchromic activity. Similarly, compounds **10–15**, **17**, and **18** were found to have a hyperchromic appearance, while only compound **16** behaved hypochromically. As a result of the easy-to-perform spectrophotometric studies, the binding constants (Kb) of the new pyrimidine-coupled *N*-glucosides and tetra-*O*-acetyl derivatives showing the affinity of the compound for DNA were determined by the following equation [31]: [DNA] / (ɛa-ɛf) = [DNA] / (ɛb −ɛf) + 1/Kb(ɛb −ɛf), the symbol [DNA] in this equation is the DNA concentration in the base pairs, and the symbols ɛa, ɛf, and ɛb are the molar absorption coefficients of the solutions Aobserved/[compound], free compound, and compound-DNA, respectively. Kb is the binding constant related to the complex to DNA and can be calculated algebraically from the slope of the line drawn between [DNA] / (ɛa-ɛf) and [DNA]. As indicated in Table 9, the Kb values of the compounds ranged from 2.4 × 10^2^ to 7.1 × 10 × M^−1^, and high Kb values indicate strong binding of the compounds to DNA. According to the CT-DNA binding experiments performed in the literature with the anticancer drugs cisplatin and 5FU, the binding constant of cisplatin was reported to be 5.73 × 10^4^ M^−1^ and the binding constant of 5FU was 9.7 × 10^4^ M^−1^ [32–33].

When we compared the DNA binding affinities of these anticancer drugs commonly used in the clinic with the DNA binding affinity of the compounds tested, we concluded that the new pyrimidine glycopyranosyl derivatives bind strongly enough to DNA. In particular, compound **1** was found to show greater interest in DNA than the anticancer drugs cisplatin and 5FU.

### 2.3. In silico studies

Molecular docking studies were performed to determine the interactions of compounds **4**, **7**, **13**, and **16** and different crystal structures (PDB IDs: 4QL3, 6MPP, 4E26, 6QGG, 5MU8, 4QUG, 2DBF, 6SL6, 1CGL, 5Z62, 6EB6, 5ITD, 4EKK, 6GU7, 7BG9, 3GUS). [34]. Schrödinger 2021–2 molecular modeling software was used to determine these interactions. Parameters such as MM-GBSA ΔG_Bind_, docking score, and complex energy values were calculated with Schrödinger 2021–2. According to these values, the strength of the interactions between the ligand and the target was calculated and the values were compared among themselves and the proteins that could have the best interaction were determined. The free binding energy, docking score and complex energy values of the compounds interacting in silico with the proteins obtained from the protein database are presented in Table 10. The values in Table 10 indicated that **NRAS, BRAF, PI3K alpha, Cytochrome c oxidase**, and **Akt1** were more effective than the other targets listed in the table.

It was analyzed considering the best values in Table 10. The ΔG_Bind_, docking score, and complex energy values of compound **13** interacting with **NRAS** (PDB ID:6MPP) were found −72.17, −5.605, and −12,873.728 kcal/mol, respectively. The ΔG_Bind_, docking score, and complex energy values of **BRAF** (PDB ID:4E26) interacting with compound **7** were calculated as −73.54, −8.034, and −11,680.540, respectively. The best binding parameter values for **Akt1** (PDB ID: 4EKK) were determined using compound **4**, and these values (ΔG_Bind_, docking score, complex energy) were calculated as −54.84, −6.141 and −14,235.655, respectively. The ΔG_Bind_, docking score and complex energy values of **Cytochrome c oxidase** (PDB ID:5Z62) and compound **7** were found −72.35, −7.643,−19,700.590 kcal/mol, respectively.

The proteins used in in silico approaches were determined based on the DNA/BSA binding properties of the compounds. These proteins are known to be important in the pathway. The values calculated for the in silico approaches showed us this. Although the data for the mentioned proteins are very good, according to Table 10, we can say that values of the binding parameters of **PI3K alpha** (PDB ID: 5TID) were the best.

When the interaction results of **PI3K alpha** with molecular docking were examined in detail, the values of ΔG_Bind_, docking score and complex energy were found −67.86, −7.306, −19,700.406 kcal/mol for compound **4**, −62.40, −7.328, −43,526.159 kcal/mol for compound **7**, −78.69, −6.754, −43,635.851 kcal/mol for compound **13** and −58.34, −4.641, −43,615.402 kcal/mol for compound **16**.

**Figure 3:**
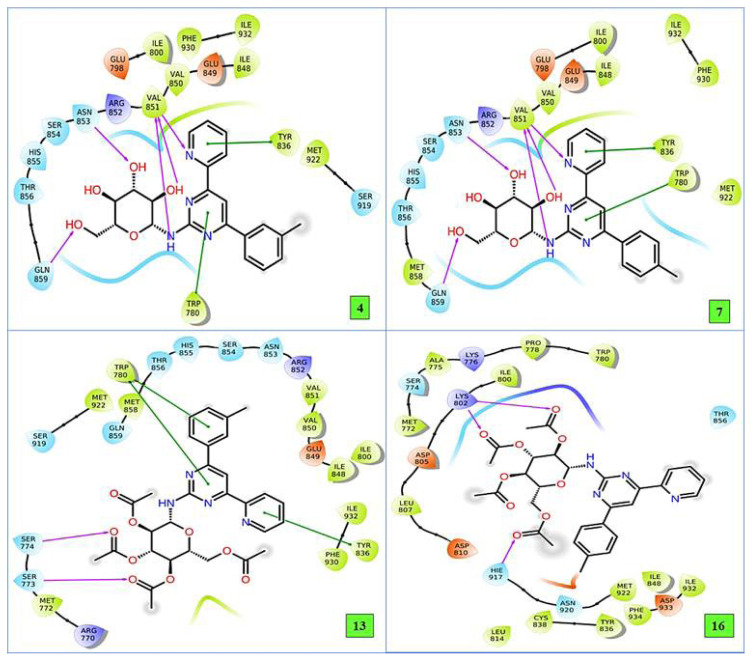
2D interaction diagrams of compounds (**4**, **7**, **13**, **16**) with the PI3K alpha protein (PDB ID: 5ITD).

When performing molecular docking analysis, the amino acid residues determined in the active binding site are as important as the energy values of the binding parameters shown in Table 10. The 2D interaction diagrams of the compounds in the binding site for **PI3K alpha**, calculated according to in silico approaches and having the best binding parameter values, are presented in Figure 3.

In Figure 3, there is a hydrogen bond with important amino acids Val851, Asn853, Gln859, and a π-π π-π stacking bond with residue Tyr836, Trp780 in the 2-dimensional interaction diagram between compound **4** and **PI3K alpha**. When this interaction was analyzed for compound **7**, it made hydrogen bonds with Val851, Asn853, Gln859 amino acid residues, and π-π stacking interaction bonds with Trp780, Tyr836. In the interaction between compound **13** and **PI3K alpha** in Figure 3, there is Trp780, π-π stacking interaction of amino acids Tyr836, and hydrogen bond interaction with amino acid residues Ser774 and Ser773. On the other hand, Lys802 in the active binding site compound **16** made hydrogen bonds with Hie917 amino acid residues.

**Figure 4:**
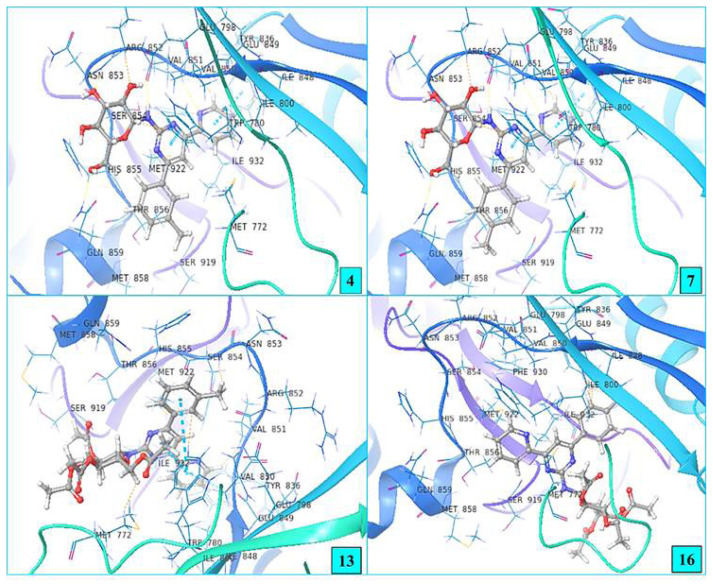
3D interaction of compounds (**4, 7, 13, 16**) with the **PI3K alpha** protein (PDB ID: 5ITD).

3D diagrams of amino acid residues of compounds interacting with **PI3K alpha** are presented in Figure 4. It is understood from Figure 4 that the compounds bind and interact to the active binding site of the crystal structure of the target from the same region.

## 3. Conclusion

In summary, pyrimidine *N*-β-D-glycosides (**1–18**) were synthesized in good yields and characterized by spectroscopic methods. The synthesis of compounds **1–9** allowed us to obtain only β-anomer of pyrimidine *N*-glucosides [10]. The β-anomeric forms for the sugar derivatives were assigned using NMR studies [12]. A total of 18 compounds were synthesized, and the literature search revealed that all of them are new.

Biological evaluations (antiproliferative, cytotoxic properties and DNA/protein binding affinities) of **1–18** were investigated. Proliferation measurements revealed that compounds **1**, **4**, **5** of the pyrimidine *N*-glucosides and **13**, **16**, and **18** of the tetra-*O*-acetyl derivatives exhibited high antiproliferative activity. They were also found to cause membrane damage of 15%–20% in toxicity tests with the same molecules. The results of these pharmacological measurements on the cell line were largely consistent with NCI criteria. As a result, when the in vitro antibacterial test results of these complexes were evaluated overall, it was found that there are similar antibacterial properties between the pyrimidine *N-β*-D-glucosides (**1–9**) and the tetra-*O*-acetyl derivatives (**10–18**). Moreover, the new synthesized molecules can bind sufficiently strongly to DNA. In particular, Compound **1** has more affinity for DNA than cisplatin. For this reason, they could be candidates for ADME/Tox and advanced phase studies due to the significant in vitro biological activity exhibited by the respective test compounds.

Recently, calculations with in silico approaches have been applied to support the experimental results. By molecular docking calculations, it was determined that the compounds **4**, **7**, **13**, and **16** had the best interaction with the PI3K alpha protein. By showing the effects of these compounds on ligands in NRAS, BRAF, Akt1, receptor/enzymes, it can be a lead drug candidate research study. Overall bioactivity results suggest that these compounds may be candidates as new chemotherapeutic agents and deserve further pharmacological evaluation.

## 4. Experimental

The materials and equipment used in this study are presented in the supplementary information.

### 4.1. Methods

#### 4.1.1. General procedure for synthesis of compounds 1–9

A mixture of 2,4,6-trisubstituted pyrimidine (15 mmol, 3.93 g), D-glucose monohydrate (15 mmol, 2.97 g), and glacial acetic acid (15 mmol, 0.85 mL) in dimethyl sulfoxide (6 mL) was heated and refluxed at 100 °C for 24 h under progress control by TLC assay [15]. TLCs were carried on silica gel (Kieselgel 60 F_254_, Merck) plates and the spots were visualized by UV lamp or spraying with 10% alcoholic H_2_SO_4_ and heating. After the TLC control, reactions were stopped, the mixtures were allowed to cool to room temperature and placed in the separating funnel, shaken by adding chloroform and then distilled water. When the shaken mixture came to rest, phase separation and the formation of a precipitate between the two phases were observed. The precipitate was obtained after separation of the solvents and was washed again with chloroform and water in a separatory funnel to try to remove impurities that might have come from unreacted pyrimidine and glucose. The purity of the precipitate was checked again with TLC and dried with a freeze dryer. The structure was confirmed by spectroscopic methods (^1^H, ^1^H-^1^H COSY and ^13^C–APT NMR, LC-MS /MS and FT-IR) and elemental analysis.

#### 5.1.2. General procedure for synthesis of compounds 10–18

Pyrimidine *N*-glycoside (1–9) (3 mmol, 1.3 g each), acetic anhydride (12 mmol, 1.2 g) and Na_2_CO_3_ (12 mmol, 1.1 g) were mixed and this mixture was stirred at 100 °C under reflux conditions for 10–20 min, and the progress was monitored by TLC [28]. Water and then chloroform were added to the finished reaction, and the mixture was placed in the separatory funnel, shaken, and allowed to rest. After phase separation, the organic phase was taken and the solvent was removed in vacuo. The product was washed with diethyl ether and dried at room temperature. This class of compounds was found to dissolve in chloroform. Purity control of the compounds was performed by TLC and their structures were elucidated by spectroscopic methods (^1^H, ^1^H-^1^H COSY and ^13^C–APT NMR, LC-MS /MS and FT-IR) and elemental analysis.

#### 4.1.3. Pharmacology

Pharmacological experiments that include the preparation of cell culture, cell proliferation assay (MTT assay), cytotoxic activity assay, microdilution assay, and DNA binding studies were performed [35] and provided in supplementary information. The calculation of IC_50_ and %inhibition was also explained in the supplementary information.

#### 4.1.4. In silico studies

Molecular docking studies were applied to determine the amino acid residues in the active site of compounds **4, 7, 13**, and **16**, which interact with all crystal structures in in silico approaches, respectively, and to calculate the binding parameters. Schrödinger 2021–2 software (Schrödinger Release 2021–2: Glide, LLC New York, USA) [36] was used to investigate the binding modes of **4, 7, 13**, and **16** compounds.

##### 4.1.4.1. Preparation of ligands

Compounds **4, 7, 13**, and **16** were optimized using the LigPrep wizard (Schrödinger Release 2021–2:LigPrep) [37] utility of the software Schrödinger 2021–2 (Schrödinger, LLC New York, USA). With this method, a net negative change in substituents was produced in each case using possible tautomeric states Epic at pH 7.0 ± 2.0 [38–39].

##### 4.1.4.2. Determination and preparation of proteins

The crystal structures of the proteins with which compounds **4, 7, 13**, and **16** interact were obtained from the Protein Data Bank (https://www.rcsb.org/). Based on the pathways in the research study, crystal structures with PDB access codes 4QL3 [40], 6MPP [41], 4E26 [42], 6QGG [43], 5MU8 [44], 4QUG [45], 2DBF, 6SL6 [46], 1CGL [47], 5Z62 [48], 6EB6 [49], 5ITD [50], 4EKK [51], 6GU7 [52], 7BG9 [53], 3GUS [54], respectively, were used. All crystal structures have different resolution values and binding sites. Crystal structures obtained from the protein database, respectively, were prepared separately with the “Protein Preparation Wizard” [55] module of Schrödinger 2021–2 software. Proteins were prepared by sequential processes such as deletion of water molecules, addition of missing side chains and hydrogen atoms, protonation states, assignment of partial charges, optimization, and minimization using the OPLS-2005 force field [39].

##### 4.1.4.3. Molecular docking and MM-GBSA

Molecular docking studies were applied for compounds **4, 7, 13**, and **16**, which are compounds with the best cytotoxicity effect. After ligand and proteins were prepared separately, the docking score was calculated by interacting with the ligand docking wizard. Ligands were docked using Schrödinger 2021–2 software (Schrödinger Release 2021–2: Glide, LLC New York, USA) [36] to investigate the binding modes of their compounds. The protocol used in molecular docking was applied as in previous studies [38–39, 56].

Prime MM/GBSA (Schrödinger Release 2021–2: Prime) [57] analysis was used to calculate ligand binding energies using the OPLS_2005 force field and the VSGB solvent model. MM-GBSA analysis was applied to calculate [58] the free binding energies and complex energies of compounds **4, 7, 13**, and **16** with all proteins mentioned in the pathway, respectively.

## Supplementary file

### 1. Experimental

#### 1.1. Materials and equipment

All starting chemical reagents and solvents used in the synthesis, purification,n and biological activity investigations were high-grade commercial products purchased from Aldrich, Fluka, Sigma, Merck, Amresco, Carlo-Erba, Lonza, Roche and used without further purification. Thin-layer chromatography (TLC) and column chromatography were performed on Merck precoated 60 Kieselgel F_254_ analytical aluminum acidic plates and silica gel 60 (0.040{0.063 mm), respectively. All reactions were monitored using TLC. ^1^H and ^13^C NMR spectra were recorded on a Bruker 400 MHz NMR in CDCl_3_, CDCl_3_/CD_3_OD, CD_3_OD, DMSO-d_6_ with tetramethyl-silane (TMS) as an internal standard. The elemental analyses were performed by using a Costech ECS 4010 instrument. Mass spectral analyses were performed on a Micromass Quattro LCMS/MS spectrophotometer. Infrared spectra were obtained using a PerkinElmer 1600FT-IR (4000–400 cm^−1^) spectrometer. Melting points were determined using a Stuart SMP10 apparatus.

#### 1.2. Characterization of compounds

##### 1.1.1. N-[4-(2-methylphenyl)-6-pyridin-2-ylpyrimidin-2-yl]-β-D-glucopyranosylamine (1)

Yield: 69%. White solid, M.p.: 106–108 °C. Rf: 0.67 (Ethyl acetate-methanol: 1:1).

FT-IR (cm^−1^): 3323 (N-H/O-H, broad), 2920 (-CH), 1578 (C=N), 1542 (C=C), 1230 (C-O), 1008 (C-N).

^1^H-NMR (400 MHz, CDCl_3_/CD_3_OD (5:1), ppm): δ = 7.8 (s, 1H, H-5); δ = 7.3–7.4 (m, 3H, H-3′/H-4′/H-5′); δ = 7.5 (d, *J* = 6.8 Hz, 1H, H-6′); δ = 8.7 (d, *J* = 4.0 Hz, 1H, H-3″); δ = 7.5 (t, *J* = 8.0 Hz, 1H, H-4″); δ = 7.9 (t, *J* = 8.0 Hz, 1H, H-5″); δ = 8.5 (d, *J* = 8.0 Hz, 1H, H-6″); δ = 5.4 (d, *J* = 8.0 Hz, 1H, H-1‴); δ = 3.4–3.6 (m, 3H, H-2‴/H-3‴/H-4‴); δ = 3.6 (m, 1H, H-5‴); δ = 3.9 (d, *J* = 11.8, 1H, H-6a‴); δ = 3.7 (dd, *J* = 11.8/4.8 Hz, 1H, H-6b‴); δ = 2.5 (s, 3H, −CH_3_).

^13^C-NMR (100 MHz, CDCl_3_/CD_3_OD, ppm): 169.7 (C-2), 163.9 (C-4), 109.0 (C-5), 154.3 (C-6), 136.0 (C-1′), 138.3 (C-2′), 130.9 (C-3′), 129.3 (C-4′), 129.1 (C-5′), 125.9 (C-6′), 161.6 (C-1″), 149.2 (C-3″), 125.3 (C-4″), 137.4 (C-5″), 122.0 (C-6″), 82.5 (C-1‴), 70.2 (C-2‴), 77.4 (C-3‴), 72.9 (C-4‴), 77.2 (C-5‴), 61.8 (C-6‴), 20.1 (-CH_3_).

Poz. LC-MS/MS m/z (%): 263 (100) [M - glucopyranosyl) + 2]^+^, 425 (18) [M + 1]^+^, 447 (22) [M + Na]^+^.

Anal. cal. for C_22_H_24_N_4_O_5_ (424.45 g/mol): C 62.25, H 5.70, N 13.20, found: C 62.27, H 5.68, N 13.22.

##### 1.1.2. N-[4-(2-methylphenyl)-6-pyridin-3-ylpyrimidin-2-yl]-β-D-glucopyranosylamine (2)

Yield:61%. White solid, M.p.: 160–162 °C. Rf: 0.78 (Ethyl acetate-methanol: 1:1).

FT-IR (cm^−1^): 3273 (N-H/O-H, broad), 2921 (−CH), 1582 (C=N), 1550 (C=C), 1215 (C-O), 1079, 1013 (C-N).

^1^H-NMR (400 MHz, CD_3_OD, ppm): δ = 7.4 (s, 1H, H-5); δ = 7.3 (m, 1H, H-3′); δ = 7.4 (m, 1H, H-4′); δ = 7.3 (m, 1H, H-5′); δ = 7.5 (d, *J* = 7.0 Hz, 1H, H-6′); δ = 9.3 (bs, 1H, H-2″); δ = 8.7 (d, *J* = 4.0 Hz, 1H, H-4″); δ = 7.6 (t, *J* = 6.5 Hz, 1H, H-5″); δ = 8.6 (d, *J* = 8.0 Hz, 1H, H-6″); δ = 5.4 (d, *J* = 8.8 Hz, 1H, H-1‴); δ = 3.4–3.6 (m, 4H, H-2‴/ H-3‴/ H-4‴/ H-5‴); δ = 3.9 (d, *J* = 11.8, 1H, H-6a‴); δ = 3.7 (dd, *J* = 11.9/4.8 Hz, 1H, H-6b‴); δ = 2.5 (s, 3H, −CH_3_).

^13^C-NMR (100 MHz, CD_3_OD, ppm): 166.6 (C-2), 162.8 (C-4), 104.1 (C-5), 162.3 (C-6), 136.0 (C-1′), 138.5 (C-2′), 130.6 (C-3′), 129.0 (C-4′), 128.9 (C-5′), 125.6 (C-6′), 133.6 (C-1″), 150.3 (C-2″), 147.8 (C-4″), 123.9 (C-5″), 135.2 (C-6″), 82.5

(C-1‴), 70.6 (C-2‴), 77.9 (C-3‴), 73.1 (C-4‴), 77.9 (C-5‴), 61.6 (C-6‴), 19.2 (−CH_3_).

Poz. LC-MS/MS m/z (%):425 (100) [M + 1]^+^, 426 (88) [M + 2]^+^.

Anal. cal. for C_22_H_24_N_4_O_5_ (424.45 g/mol): C 62.25, H 5.70, N 13.20, found: C 62.26, H 5.68, N 13.21.

##### 1.1.3. N-[4-(2-methylphenyl)-6-pyridin-4-ylpyrimidin-2-yl]-β-D-glucopyranosylamine (3)

Yield: 73%. White solid, M.p.: 144–146 °C. Rf: 0.78 (Ethyl acetate-methanol: 1:1).

FT-IR (cm^−1^): 3304 (N-H/ O-H, broad), 2911 (−CH), 1579 (C=N), 1538 (C=C), 1245 (C-O), 1076, 1015 (C-N).

^1^H-NMR (400 MHz, CD_3_OD, ppm): δ = 7.4 (s, 1H, H-5); δ = 7.3 (m, 1H, H-3′); δ = 7.4 (m, 1H, H-4′); δ = 7.3 (m, 1H, H-5′); δ = 7.5 (d, *J* = 7.5 Hz, 1H, H-6′); δ = 8.1 (d, *J* = 6.0 Hz, 1H, H-2″); δ = 8.7 (bs, 1H, H-3″); δ = 8.7 (bs, 1H, H-5″); δ = 8.1 (d, *J* = 6.0 Hz, 1H, H-6″); δ = 5.4 (d, *J* = 8.8 Hz, 1H, H-1‴); δ = 3.4–3.6 (m, 4H, H-2‴/ H-3‴/ H-4‴/ H-5‴); δ = 3.9 (d, *J* = 11.9, 1H, H-6a‴); δ =3.7 (dd, *J* = 11.9/5.0 Hz, 1H, H-6b‴); δ = 2.5 (s, 3H, −CH_3_).

^13^C-NMR (100 MHz, CD_3_OD, ppm): 170.2 (C-2), 162.5 (C-4), 108.1 (C-5), 162.4 (C-6), 138.4 (C-1′), 145.7 (C-2′), 130.7 (C-3′), 129.1 (C-4′), 128.9 (C-5′), 125.6 (C-6′), 140.3 (C-1″), 121.4 (C-2″), 145.7 (C-3″), 145.7 (C-5″), 121.4 (C-6″), 82.5 (C-1‴), 70.6 (C-2‴), 77.9 (C-3‴), 73.0 (C-4‴), 77.9 (C-5‴), 61.6 (C-6‴), 19.2 (−CH_3_).

Poz. LC-MS/MS m/z (%): 109 (100) [Methylphenyl + H_2_O]^+^, 263 (50) [M - glucopyranosyl + 2]^+^, 425 (30) [M + 1]^+^.

Anal. cal. for C_22_H_24_N_4_O_5_ (424.45 g/mol): C 62.25, H 5.70, N 13.20, found: C 62.30, H 5.66, N 13.22.

##### 1.1.4. N-[4-(3-methylphenyl)-6-pyridin-2-ylpyrimidin-2-yl]-β-D-glucopyranosylamine (4)

Yield: 80%. White solid, M.p.: 136–138 °C. Rf: 0.78 (Ethyl acetate-methanol: 1:1).

FT-IR (cm^−1^): 3317 (N-H/ O-H, broad), 2920 (−CH), 1573 (C=N), 1544 (C=C), 1241 (C-O), 1079, 1012 (C-N).

^1^H-NMR (400 MHz, CDCl_3_/CD_3_OD (5:1), ppm): δ = 8.1 (s, 1H, H-5); δ = 7.9 (s, 1H, H-2′); δ = 7.3 (d, *J* = 7.5 Hz, 1H, H-4′); δ = 7.4 (m, 1H, H-5′); δ = 7.9 (m, 1H, H-6′); δ = 8.7 (d, *J* = 4.0 Hz, 1H, H-3″); δ = 7.4 (m, 1H, H-4″); δ = 7.9 (m, 1H, H-5″); δ = 8.4 (d, *J* = 7.8 Hz, 1H, H-6″); δ = 5.5 (d, *J* = 9.0 Hz, 1H, H-1‴); δ = 3.4–3.7 (m, 4H, H-2‴/ H-3‴/ H-4‴/ H-5‴); δ = 3.9 (dd, *J* = 12.0/2.3, 1H, H-6a‴); δ = 3.8 (dd, *J* = 12.1/4.5 Hz, 1H, H-6b‴); δ = 2.5 (s, 3H, −CH_3_).

^13^C-NMR (100 MHz, CDCl_3_/CD_3_OD, ppm): 166.7 (C-2), 138.4 (C-4), 105.2 (C-5), 162.2 (C-6), 137.2 (C-1′), 131.5 (C-2′), 154.4 (C-3′), 127.8 (C-4′), 126.6 (C-5′), 125.3 (C-6′), 164.2 (C-1″), 149.1 (C-3″), 124.4 (C-4″), 137.4 (C-5″), 121.9 (C-6″), 82.6 (C-1‴), 70.3 (C-2‴), 77.5 (C-3‴), 73.0 (C-4‴), 77.4 (C-5‴), 61.9 (C-6‴), 21.2 (−CH_3_).

Poz. LC-MS/MS m/z (%): 425 (100) [M + 1]^+^.

Anal. cal. for C_22_H_24_N_4_O_5_ (424.45 g/mol): C 62.25, H 5.70, N 13.20, found: C 62.27, H 5.69, N 13.23.

##### 1.1.5. N-[4-(3-methylphenyl)-6-pyridin-3-ylpyrimidin-2-yl]-β-D-glucopyranosylamine (5)

Yield: 81%. White solid, M.p.: 152–154 °C. Rf: 0.81 (Ethyl acetate-methanol: 1:1).

FT-IR (cm^−1^): 3312 (N-H/ O-H, broad), 2900 (−CH), 1579 (C=N), 1545 (C=C), 1203 (C-O), 1079, 1030 (C-N).

^1^H-NMR (400 MHz CDCl_3_/CD_3_OD (5:1), ppm): δ = 8.0 (s, 1H, H-5); δ = 7.8 (s, 1H, H-2′); δ = 7.3 (d, *J* = 8.0 Hz, 1H, H-4′); δ = 7.4 (t, *J* = 8.0 Hz, 1H, H-5′); δ = 8.0 (d, *J* = 8.0 Hz, 1H, H-6′); δ = 9.4 (s, 1H, H-2″); δ = 8.7 (d, *J* = 4.8 Hz, 1H, H-4″); δ = 7.6 (dd, *J* = 8.0/4.0 Hz 1H, H-5″); δ = 8.6 (d, *J* = 8.0 Hz, 1H, H-6″); δ = 5.5 (d, *J* = 9.2 Hz, 1H, H-1‴); δ = 3.4–3.6 (m, 4H, H-2‴/ H-3‴/ H-4‴/ H-5‴); δ = 3.9 (d, *J* = 12.0 Hz, 1H, H-6a‴); δ = 3.7 (dd, *J* = 12.0/4.0 Hz, 1H, H-6b‴); δ = 2.5 (s, 3H, −CH_3_).

^13^C-NMR (100 MHz, DMSO-d_6_, ppm): 169.2 (C-2), 139.0 (C-4), 105.0 (C-5), 169.2 (C-6), 138.1 (C-1′), 136.1 (C-2′), 139.0 (C-3′), 129.8 (C-4′), 131.2 (C-5′), 126.2 (C-6′), 135.0 (C-1″), 153.2 (C-2″), 150.2 (C-4″), 126.0 (C-5″), 133.3 (C-6″), 82.6 (C-1‴), 70.4 (C-2‴), 78.1 (C-3‴), 72.2 (C-4‴), 77.8 (C-5‴), 61.0 (C-6‴), 20.9 (−CH_3_).

Poz. LC-MS/MS m/z (%):426 (25) [M + 2]^+^.

Anal. cal. for C_22_H_24_N_4_O_5_ (424.45 g/mol): C 62.25, H 5.70, N 13.20, found: C 62.27, H 5.71, N 13.20.

##### 1.1.6. N-[4-(3-methylphenyl)-6-pyridin-4-ylpyrimidin-2-yl]-β-D-glucopyranosylamine (6)

Yield: 59 %. White solid, M.p.: 160–162 °C. Rf: 0.80 (Ethyl acetate-methanol: 1:1).

FT-IR (cm^−1^): 3382 (N-H/ O-H, broad), 2913 (−CH), 1583 (C=N), 1540 (C=C), 1249 (C-O) 1079, 1027 (C-N).

^1^H-NMR (400 MHz, CDCl_3_/CD_3_OD (5:1), ppm): δ = 7.9 (s, 1H, H-5); δ = 7.6 (s, 1H, H-2′); δ = 7.3 (d, *J* = 8.0 Hz, 1H, H-4′); δ = 7.4 (t, *J* = 8.0 Hz, 1H, H-5′); δ = 7.9 (bs, 1H, H-6′); δ = 8.1 (bs, 2H, H-2″/ H-6″); δ = 8.7 (d, *J* = 4.0 Hz, 2H, H-3″/H-5″); δ = 5.5 (d, *J* = 12.0 Hz, 1H, H-1‴); δ = 3.4–3.6 (m, 4H, H-2‴/ H-3‴/ H-4‴/ H-5‴); δ = 3.9 (d, *J* = 12.0 Hz, 1H, H-6a‴); δ = 3.8 (dd, *J* = 12.0/4.0 Hz, 1H, H-6b‴); δ = 2.4 (s, 3H, −CH_3_).

^13^C-NMR (100 MHz, CD_3_OD, ppm): 166.9 (C-2), 161.0 (C-4), 104.1 (C-5), 145.9 (C-6), 136.9 (C-1′), 131.2 (C-2′), 162.3 (C-3′), 127.5 (C-4′), 128.3 (C-5′), 124.2 (C-6′), 138.2 (C-1″), 121.5 (C-2″), 149.3 (C-3″), 149.3 (C-5″), 121.5 (C-6″), 82.5 (C-1‴), 70.4 (C-2‴), 78.1 (C-3‴), 72.9 (C-4‴), 77.8 (C-5‴), 61.5 (C-6‴), 20.1 (−CH_3_).

Poz. LC-MS/MS m/z (%):425 (100) [M + 1]^+^.

Anal. cal. for C_22_H_24_N_4_O_5_ (424.45 g/mol): C 62.25, H 5.70, N 13.20, found: C 62.27, H 5.71, N 13.22.

##### 1.1.7. N-[4-(4-methylphenyl)-6-pyridin-2-ylpyrimidin-2-yl]-β-D-glucopyranosylamine (7)

Yield: 63 %. White solid, M.p.: 154–156 °C. Rf: 0.85 (Ethyl acetate-methanol: 1:1).

FT-IR (cm^−1^): 3311 (N-H/ O-H, broad), 2921 (−CH), 1579 (C=N), 1542 (C=C), 1235 (C-O), 1080, 1011 (C-N).

^1^H-NMR (400 MHz, CDCl_3_/CD_3_OD (5:1), ppm): δ = 8.1 (s, 1H, H-5); δ = 8.1 (d, *J* = 8.0 Hz, 2H, H-2′/ H-6′); δ = 7.3 (d, *J* = 8.0 Hz, 2H, H-3′/H-5′); δ = 8.7 (d, *J* = 4.4 Hz, 1H, H-3″); δ = 7.5 (dd, *J* = 8.0/4.0 Hz, 1H, H-4″); δ = 7.9 (t, *J* = 8.0 Hz, 1H, H-5″); δ = 8.4 (d, *J* = 8.0 Hz, 1H, H-6″); δ = 5.5 (d, *J* = 9.2 Hz, 1H, H-1‴); δ = 3.4–3.6 (m, 4H, H-2‴/ H-3‴/ H-4‴/ H-5‴); δ = 3.9 (dd, *J* = 12.0/2.0, 1H, H-6a‴); δ = 3.7 (dd, *J* = 12.0/5.2 Hz, 1H, H-6b‴); δ = 2.4 (s, 3H, −CH_3_).

^13^C-NMR (100 MHz, CDCl_3_/CD_3_OD, ppm): 164.0 (C-2), 141.0 (C-4), 104.1 (C-5), 162.5 (C-6), 134.5 (C-1′), 129.2 (C-2′), 127.0 (C-3′), 141.0 (C-4′), 127.0 (C-5′), 129.2 (C-6′), 162.5 (C-1″), 148.9 (C-3″), 125.2 (C-4″), 137.5 (C-5″), 122.0 (C-6″), 82.6 (C-1‴), 70.5 (C-2‴), 77.9 (C-3‴), 73.0 (C-4‴), 77.7 (C-5‴), 61.6 (C-6‴), 20.5 (−CH_3_).

Poz. LC-MS/MS m/z (%):109 (100) [methylphenyl + H_2_O] ^+^, 263 (95) [M-glucopyranosyl + 2]^+^, 425 (55) [M + 1]^+^, 447 (50) [M + Na]^+^.

Anal. cal. for C_22_H_24_N_4_O_5_ (424.45 g/mol): C 62.25, H 5.70, N 13.20, found: C 62.30, H 5.71, N 13.24.

##### 1.1.8. N-[4-(4-methylphenyl)-6-pyridin-3-ylpyrimidin-2-yl]-β-D-glucopyranosylamine (8)

Yield: 68 %. White solid, M.p.: 142–144 °C. Rf: 0.85 (Ethyl acetate-methanol: 1:1).

FT-IR (cm^−1^): 3337 (N-H/ O-H, broad), 2920 (−CH), 1585 (C=N), 1542 (C=C), 1216 (C-O), 1081, 1019 (C-N).

^1^H-NMR (400 MHz, CD_3_OD, ppm): δ = 7.6 (s, 1H, H-5); δ = 8.1 (d, *J* = 8.0 Hz, 2H, H-2′/ H-6′); δ = 7.3 (d, *J* = 8.0 Hz, 2H, H-3′/H-5′); δ = 9.3 (s, 1H, H-2″); δ = 8.6 (d, *J* = 4.0 Hz, 1H, H-4″); δ = 7.6 (dd, *J* = 8.0/4.0 Hz, 1H, H-5″); δ = 8.6 (d, *J* = 8.0 Hz, 1H, H-6″); δ = 5.5 (d, *J* = 8.8 Hz, 1H, H-1‴); δ = 3.4–3.6 (m, 4H, H-2‴/ H-3‴/ H-4‴/ H-5‴); δ = 3.9 (d, *J* = 11.8, 1H, H-6a‴); δ = 3.7 (dd, *J* = 11.9/5.5 Hz, 1H, H-6b‴); δ = 2.4 (s, 3H, −CH_3_).

^13^C-NMR (100 MHz, DMSO-d_6_, ppm): 162.3 (C-2), 141.2 (C-4), 103.3 (C-5), 162.3 (C-6), 134.6 (C-1′), 129.8 (C-2′), 127.6 (C-3′), 141.2 (C-4′), 127.6 (C-5′), 129.8 (C-6′), 133.2 (C-1″), 151.7 (C-2″), 148.8 (C-4″), 124.2 (C-5″), 134.9 (C-6″), 82.9 (C-1‴), 70.8 (C-2‴), 78.9 (C-3‴), 72.8 (C-4‴), 78.4 (C-5‴), 61.6 (C-6‴), 21.5 (−CH_3_).

Poz. LC-MS/MS m/z (%): 425 (100) [M + 1]^+^, 426 (78) [M + 2]^+^.

Anal. cal. for C_22_H_24_N_4_O_5_ (424.45 g/mol): C 62.25, H 5.70, N 13.20, found: C 62.28, H 5.74, N 13.22.

##### 1.1.9. N-[4-(4-methylphenyl)-6-pyridin-4-ylpyrimidin-2-yl]-β-D-glucopyranosylamine (9)

Yield: 73 %. White solid, M.p.: 173–175 °C. Rf: 0.67(Ethyl acetate-methanol: 1:1).

FT-IR (cm^−1^): 3287 (N-H/ O-H, broad), 2914 (−CH), 1581 (C=N), 1535 (C=C), 1218 (C-O), 1079, 1023 (C-N).

^1^H-NMR (400 MHz, CD_3_OD, ppm): δ= 7.8 (s, 1H, H-5); δ = 8.2 (d, *J* = 5.8 Hz, 1H, H-2′); δ = 7.3 (d, *J* = 8.0 Hz, 1H, H-3′); δ = 7.3 (d, *J* = 8.0 Hz, 1H, H-5′); δ = 8.2 (d, *J* = 5.8 Hz, 1H, H-6′); δ = 8.9 (d, *J* = 8.3 Hz, 1H, H-2″); δ = 8.7 (d, *J* = 4.3 Hz, 1H, H-3″); δ = 8.7 (d, *J* = 4.3 Hz, 1H, H-5″); δ = 8.1 (d, *J* = 8.3 Hz, 1H, H-6″); δ = 5.5 (d, *J* = 8.8 Hz, 1H, H-1‴); δ = 3.4–3.6 (m, 4H, H-2‴/ H-3‴/ H-4‴/ H-5‴); δ = 3.9 (dd, *J* = 11.8/2.2, 1H, H-6a‴); δ = 3.7 (dd, *J* = 11.9/5.2 Hz, 1H, H-6b‴); δ = 2.4 (s, 3H, −CH_3_).

^13^C-NMR (100 MHz, CD_3_OD, ppm): 167.1 (C-2), 146.1 (C-4), 103.9 (C-5), 162.9 (C-6), 141.2 (C-1′), 129.1 (C-2′), 121.4 (C-3′), 146.0 (C-4′), 121.4 (C-5′), 129.1 (C-6′), 134.3 (C-1″), 127.0 (C-2″), 149.5 (C-3″), 149.5 (C-5″), 127.0 (C-6″), 82.7 (C-1‴), 70.7 (C-2‴), 78.1 (C-3‴), 73.1 (C-4‴), 78.0 (C-5‴), 61.7 (C-6‴), 20.0 (−CH_3_).

Poz. LC-MS/MS m/z (%):425 (100) [M + 1]^+^, 426 (85) [M + 2]^+^.

Anal. cal. for C_22_H_24_N_4_O_5_ (424.45 g/mol): C 62.25, H 5.70, N 13.20, found: C 62.28, H 5.71, N 13.22.

##### 1.1.10. 2,3,4,6-tetra-O-acetyl-N-[4-(2-methylphenyl)-6-pyridin-2-ylpyrimidin-2-yl]-β-D-glucopyranosylamine (10)

Yield: 89 %. White solid, M.p.: 171–173 °C. Rf: 0.75 (Diethyl ether-ethyl acetate: 1:1).

FT-IR (cm^−1^): 3410 (N-H), 2966 (−CH), 1739 (C=O), 1577 (C=N), 1545 (C=C), 1221 (C-O), 1031 (C-N).

^1^H-NMR (400 MHz, CDCl_3_, ppm): δ = 8.0 (s, 1H, H-5); δ = 7.3 (bs, 1H, H-3′); δ = 7.3 (m, 1H, H-4′); δ = 7.3 (m, 1H, H-5′); δ = 7.5 (bs, 1H, H-6′); δ = 8.7 (d, *J* = 3.9 Hz, 1H, H-3″); δ = 7.4 (t, *J* = 6.1 Hz, 1H, H-4″); δ = 7.9 (t, *J* = 7.4 Hz, 1H, H-5″); δ = 8.4 (d, *J* = 7.9 Hz, 1H, H-6″); δ = 6.0 (d, *J* = 9.6 Hz, 1H, H-1‴); δ = 5.7 (t, *J* = 9.2 Hz, 1H, H-2‴); δ = 5.2 (m, 1H, H-3‴); δ = 5.4 (t, *J* = 8.3 Hz, 1H, H-4‴); δ = 5.2 (m, 1H, H-5‴); δ = 4.3 (dd, *J* = 12.0/4.4 Hz, 1H, H-6a‴); δ = 4.1 (d, *J* = 11.8 Hz, 1H, H-6b‴); δ = 2.5 (s, 3H, −CH_3_); δ = 3.9 (bs, 1H, NH); δ = 1.9–2.0 (m, 12H, acetyl CH_3_).

^13^C-NMR (100 MHz, CDCl_3_, ppm): 160.7 (C-2), 154.4 (C-4), 109.5 (C-5), 138.3 (C-6), 136.3 (C-1′), 136.9 (C-2′), 129.5 (C-3′), 129.3 (C-4′), 129.3 (C-5′), 125.6 (C-6′), 138.3 (C-1″), 149.4 (C-3″), 125.1 (C-4″), 131.2 (C-5″), 121.6 (C-6″), 81.2 (C-1‴), 68 (C-2‴), 73.3 (C-3‴), 73.1 (C-4‴), 70.5 (C-5‴), 62.1 (C-6‴), 20.7–20.6 (acetyl CH_3_), 170.7/ 170.6/ 170.1/ 169.6 (C=O), 20.6 (−CH_3_).

Poz. LC-MS/MS m/z (%):615 (100) [M + Na]^+^.

Anal. cal. for C_30_H_32_N_4_O_9_ (592.60 g/mol): C 60.80, H 5.44, N 9.45, found: C 60.78, H 5.49, N 9.50.

##### 1.1.11. 2,3,4,6-tetra-O-acetyl-N-[4-(2-methylphenyl)-6-pyridin-3-ylpyrimidin-2-yl]-β-D-glucopyranosylamine (11)

Yield: 82 %. White solid, M.p.: 179–181 °C. Rf: 0.54 (Diethyl ether-ethyl acetate: 1:1).

FT-IR (cm^−1^): 3406 (N-H), 2966 (−CH), 1740 (C=O), 1581 (C=N), 1543 (C=C), 1220 (C-O), 1034, 1023 (C-N).

^1^H-NMR (400 MHz, CDCl_3_, ppm): δ = 7.3 (s, 1H, H-5); δ = 7.3 (bs, 1H, H-3′); δ = 7.4 (m, 1H, H-4′); δ = 7.3 (bs, 1H, H-5′); δ = 7.4 (m, 1H, H-6′); δ = 9.3 (bs, 1H, H-2″); δ = 8.7 (bs, 1H, H-4″); δ = 7.5 (m, 1H, H-5″); δ = 8.4 (d, *J* = 7.0 Hz, 1H, H-6″); δ = 6.1 (bs, 1H, H-1‴); δ = 5.7 (t, *J* = 9.2 Hz, 1H, H-2‴); δ = 5.2 (m, 1H, H-3‴); δ = 5.4 (t, *J* = 8.8 Hz, 1H, H-4‴); δ = 5.1 (m, 1H, H-5‴); δ = 4.3 (dd, *J* = 12.0/3.5 Hz, 1H, H-6a‴); δ = 4.1 (d, *J* = 11.0 Hz, 1H, H-6b‴); δ = 2.5 (s, 3H, −CH_3_); δ = 3.9 (bs, 1H, NH); δ = 1.9–2.0 (m, 12H, acetyl CH_3_).

^13^C-NMR (100 MHz, CDCl_3_, ppm): 161.0 (C-2), 161.0 (C-4), 109.0 (C-5), 151.4 (C-6), 136.2 (C-1′), 138.0 (C-2′), 129.6 (C-3′), 129.5 (C-4′), 129.3 (C-5′), 126.1 (C-6′), 132.8 (C-1″), 148.5 (C-2″), 134.5 (C-4″), 123.6 (C-5″), 131.3 (C-6″), 81.1 (C-1‴), 68.6 (C-2‴), 73.3 (C-3‴), 73.2 (C-4‴), 70.5 (C-5‴), 62.1 (C-6‴), 20.7–20.6 (acetyl CH_3_), 170.7/ 170.5/ 170.0/ 169.6 (C=O), 20.6 (−CH_3_).

Poz. LC-MS/MS m/z (%):615 (100) [M + Na]^+^, 616 (94) [M + Na + 1]^+^.

Anal. cal. for C_30_H_32_N_4_O_9_ (592.60 g/mol): C 60.80, H 5.44, N 9.45, found: C 60.78, H 5.47, N 9.52.

##### 1.1.12. 2,3,4,6-tetra-O-acetyl-N-[4-(2-methylphenyl)-6-pyridin-4-ylpyrimidin-2-yl]-β-D-glucopyranosylamine (12)

Yield: 78 %. White solid, M.p.: 192–194 °C. Rf: 0.50 (Diethyl ether-ethyl acetate: 1:1).

FT-IR (cm^−1^): 3429 (N-H), 2944 (−CH), 1740 (C=O), 1581 (C=N), 1535 (C=C), 1217 (C-O), 1030 (C-N).

^1^H-NMR (400 MHz, CDCl_3_, ppm): δ = 7.3 (s, 1H, H-5); δ = 7.3 (bs, 1H, H-3′); δ = 7.5 (bs, 1H, H-4′); δ = 7.3 (bs, 1H, H-5′); δ = 8.0 (d, *J* = 6.6 Hz, 1H, H-6′); δ = 7.9 (bs, 1H, H-2″); δ = 8.8 (bs, 1H, H-3″); δ = 8.8 (bs, 1H, H-5″); δ = 7.9 (bs, 1H, H-6″); δ = 6.1 (d, *J* = 10.0 Hz, 1H, H-1‴); δ = 5.7 (m, 1H, H-2‴); δ = 5.2 (m, 1H, H-3‴); δ = 5.5 (m, 1H, H-4‴); δ = 5.1 (m, 1H, H-5‴); δ = 4.3 (d, *J* = 11.8 Hz, 1H, H-6a‴); δ = 4.1 (d, *J* = 11.8 Hz, 1H, H-6b‴); δ = 2.5 (s, 3H, −CH_3_); δ = 3.9 (bs, 1H, NH); δ = 1.9–2.0 (m, 12H, acetyl CH_3_).

^13^C-NMR (100 MHz, CDCl_3_, ppm): 161.5 (C-2), 161.1 (C-4), 109.4 (C-5), 144.4 (C-6), 136.3 (C-1′), 141.6 (C-2′), 131.3 (C-3′), 129.7 (C-4′), 129.3 (C-5′), 127.1 (C-6′), 137.9 (C-1″), 121.0 (C-2″), 150.6 (C-3″), 150.6 (C-5″), 121.0 (C-6″), 81.2 (C-1‴), 68.6 (C-2‴), 73.2 (C-3‴), 70.4 (C-4‴), 69.0 (C-5‴), 62.2 (C-6‴), 20.7–20.6 (acetyl CH_3_), 170.6/ 170.0/ 170.0/ 169.5 (C=O), 20.6 (−CH_3_).

Poz. LC-MS/MS m/z (%):593 (80) [M+1]^+^, 616 (34) [M+Na+1]^+^.

Anal. cal. for C_30_H_32_N_4_O_9_ (592.60 g/mol): C 60.80, H 5.44, N 9.45, found: C 60.78, H 5.50, N 9.47.

##### 1.1.13. 2,3,4,6-tetra-O-acetyl-N-[4-(3-methylphenyl)-6-pyridin-2-ylpyrimidin-2-yl]-β-D-glucopyranosylamine (13)

Yield: 87 %. White solid, M.p.: 176–178 °C. Rf: 0.83 (Diethyl ether-ethyl acetate: 1:1).

FT-IR (cm^−1^): 3416 (N-H), 2945 (−CH), 1741 (C=O), 1576 (C=N), 1548 (C=C), 1219 (C-O), 1032 (C-N)

^1^H-NMR (400 MHz, CDCl_3_, ppm): δ = 8.3 (s, 1H, H-5); δ = 8.0 (bs, 1H, H-2′); δ = 7.3 (bs, 1H, H-4′); δ = 7.4 (bs, 1H, H-5′); δ = 8.0 (bs, 1H, H-6′); δ = 8.7 (bs, 1H, H-3″); δ = 7.4 (bs, 1H, H-4″); δ = 7.9 (m, 1H, H-5″); δ = 8.4 (d, *J* = 7.0 Hz, 1H, H-6″); δ = 6.0 (d, *J* = 9.6 Hz, 1H, H-1‴); δ = 5.8 (t, *J* = 9.2 Hz 1H, H-2‴); δ = 5.2 (m, 1H, H-3‴); δ = 5.5 (t, *J* = 9.6 Hz, 1H, H-4‴); δ = 5.2 (m, 1H, H-5‴); δ = 4.3 (dd, *J* = 11.4/4.8 Hz, 1H, H-6a‴); δ = 4.2 (d, *J* = 11.8 Hz, 1H, H-6b‴); δ = 2.5 (s, 3H, −CH_3_); δ = 4.0 (bs, 1H, NH); δ = 1.9–2.0 (m, 12H, acetyl CH_3_).

^13^C-NMR (100 MHz, CDCl_3_, ppm): 161.2 (C-2), 154.5 (C-4), 105.5 (C-5), 154.5 (C-6), 137.1 (C-1′), 131.5 (C-2′), 138.3 (C-3′), 127.9 (C-4′), 128.6 (C-5′), 125.1 (C-6′), 141.3 (C-1″), 149.3 (C-3″), 124.5 (C-4″), 136.9 (C-5″), 121.6 (C-6″), 81.4 (C-1‴), 68.9 (C-2‴), 73.3 (C-3‴), 73.2 (C-4‴), 70.6(C-5‴), 62.2 (C-6‴), 21.5–20.7 (acetyl CH_3_), 170.7/ 170.7/ 170.1/ 169.6 (C=O), 20.6 (−CH_3_).

Poz. LC-MS/MS m/z (%):197 (100) [M - (methylphenyl + tetra-*O-*Ac-Glucopyranosyl) + 2]^+^.

Anal. cal. for C_30_H_32_N_4_O_9_ (592.60 g/mol): C 60.80, H 5.44, N 9.45, found: C 60.81, H 5.51, N 9.48.

##### 1.1.14. 2,3,4,6-tetra-O-acetyl-N-[4-(3-methylphenyl)-6-pyridin-3-ylpyrimidin-2-yl]-β-D-glucopyranosylamine (14)

Yield: 91 %. White solid, M.p.: 211–213 °C. Rf: 0.50 (Diethyl ether-ethyl acetate: 1:1).

FT-IR (cm^−1^): 3421 (NH), 2945 (C-H), 1740 (C=O), 1572 (C=N), 1546 (C=O), 1217 (C-O), 1031 (C-N).

^1^H-NMR (400 MHz, CDCl_3_, ppm): δ = 7.9 (s, 1H, H-5); δ = 7.6 (s, 1H, H-2′); δ = 7.3 (d, *J* = 6.6 Hz, 1H, H-4′); δ = 7.4 (m, 1H, H-5′); δ = 8.0 (bs, 1H, H-6′); δ = 9.3 (bs, 1H, H-2″); δ = 8.7 (d, *J* = 4.0 Hz, 1H, H-4″); δ = 7.4 (m, 1H, H-5″); δ = 8.4 (d, *J* = 7.5 Hz, 1H, H-6″); δ = 6.0 (d, *J* = 9.6 Hz, 1H, H-1‴); δ = 5.8 (t, *J* = 9.2 Hz 1H, H-2‴); δ = 5.2 (m, 1H, H-3‴); δ = 5.5 (t, *J* = 9.2 Hz, 1H, H-4‴); δ = 5.2 (m, 1H, H-5‴); δ = 4.5 (dd, *J* = 12.0/4.8 Hz, 1H, H-6a‴); δ = 4.1 (d, *J* = 12.2 Hz, 1H, H-6b‴); δ = 2.5 (s, 3H, −CH_3_); δ = 4.0 (bs, 1H, NH); δ = 1.9–2.0 (m, 12H, acetyl CH_3_).

^13^C-NMR (100 MHz, CDCl_3_, ppm): 161.5 (C-2), 105.2 (C-5), 153.5 (C-6), 136.9 (C-1′), 134.5 (C-2′), 138.6 (C-3′), 127.8 (C-4′), 128.8 (C-5′), 124.3 (C-6′), 133.0 (C-1″), 151.4 (C-2″), 148.5 (C-4″), 123.6 (C-5″), 131.7 (C-6″), 81.2 (C-1‴), 68.8 (C-2‴), 73.3 (C-3‴), 73.3 (C-4‴), 70.5 (C-5‴), 62.2 (C-6‴), 21.6–20.6 (acetyl CH_3_), 170.7/ 170.1/ 170.1/ 169.6 (C=O), 20.0 (−CH_3_).

Poz. LC-MS/MS m/z (%):615 (100) [M + Na]^+^, 616 (94) [M +1 + Na]^+^.

Anal. cal. for C_30_H_32_N_4_O_9_ (592.60 g/mol): C 60.80, H 5.44, N 9.45, found: C 60.83, H 5.50, N 9.46.

##### 1.1.15. 2,3,4,6-tetra-O-acetyl-N-[4-(3-methylphenyl)-6-pyridin-4-ylpyrimidin-2-yl]-β-D-glucopyranosylamine (15)

Yield: 83 %. White solid, M.p.: 146–148 °C. Rf: 0.50 (Diethyl ether-ethyl acetate: 1:1).

FT-IR (cm^−1^): 3420 (N-H), 2972 (−CH), 1741 (C=O), 1582 (C=N), 1538 (C=C), 1220 (C-O), 1033 (C-N).

^1^H-NMR (400 MHz, CDCl_3_, ppm): δ = 7.9 (s, 1H, H-5); δ = 7.6 (s, 1H, H-2′); δ = 7.4 (bs, 1H, H-4′); δ = 7.4 (t, *J* = 7.0 Hz, 1H, H-5′); δ = 7.9 (bs, 1H, H-6′); δ = 8.0 (d, *J* = 3.1 Hz, 1H, H-2″); δ = 8.8 (bs, 1H, H-3″); δ = 8.8 (bs, 1H, H-5″); δ = 8.0 (d, *J* = 3.1 Hz, 1H, H-6″); δ = 6.0 (d, *J* = 10.0 Hz, 1H, H-1‴); δ = 5.8 (t, *J* = 8.8 Hz 1H, H-2‴); δ = 5.2 (m, 1H, H-3‴); δ = 5.5 (t, *J* = 9.7 Hz, 1H, H-4‴); δ = 5.2 (m, 1H, H-5‴); δ = 4.3 (dd, *J* = 11.8/5.7 Hz, 1H, H-6a‴); δ = 4.2 (d, *J* = 11.4 Hz, 1H, H-6b‴); δ = 2.5 (s, 3H, −CH_3_); δ = 4.0 (bs, 1H, NH); δ = 1.9–2.0 (m, 12H, acetyl CH_3_).

^13^C-NMR (100 MHz, CDCl_3_, ppm): 161.6 (C-2), 160.6 (C-4), 105.5 (C-5), 153.3 (C-6), 136.6 (C-1′), 131.9 (C-2′), 160.6 (C-3′), 127.8 (C-4′), 128.8 (C-5′), 124.3 (C-6′), 136.6 (C-1″), 121.1 (C-2″), 150.6 (C-3″), 150.6 (C-5″), 121.1 (C-6″), 81.2 (C-1‴), 68.8 (C-2‴), 73.2 (C-3‴), 70.5 (C-4‴), 70.5 (C-5‴), 62.2 (C-6‴), 21.6–20.6 (acetyl CH_3_), 170.7/ 170.6/ 170.1/ 169.6 (C=O), 20.6 (−CH_3_).

Poz. LC-MS/MS m/z (%):594 (62) [M + 2]^+^, 616 (40) [M +1 + Na]^+^.

Anal. cal. for C_30_H_32_N_4_O_9_ (592.60 g/mol): C 60.80, H 5.44, N 9.45, found: C 60.78, H 5.50, N 9.42.

##### 1.1.16. 2,3,4,6-tetra-O-acetyl-N-[4-(4-methylphenyl)-6-pyridin-2-ylpyrimidin-2-yl]-β-D-glucopyranosylamine (16)

Yield: 93 %. White solid, M.p.: 178–180 °C. Rf: 0.85 (Diethyl ether-ethyl acetate: 1:1).

FT-IR (cm^−1^): 3414 (N-H), 2989 (−CH), 1741 (C=O), 1578 (C=N), 1544 (C=O), 1221 (C-O), 1033 (C-N).

^1^H-NMR (400 MHz, CDCl_3_/CD_3_OD (5:1), ppm): δ = 8.3 (s, 1H, H-5); δ = 8.1 (d, *J* = 7.0 Hz, 1H, H-2′); δ = 7.3 (d, *J* = 7.0 Hz, 1H, H-3′); δ = 7.3 (d, *J* = 7.0 Hz, 1H, H-5′); δ = 8.9 (d, *J* = 7.0 Hz, 1H, H-6′); δ = 8.7 (bs, 1H, H-3″); δ = 7.4 (t, *J* = 6.6 Hz, 1H, H-4″); δ = 7.9 (t, *J* = 7.0 Hz, 1H, H-5″); δ = 8.4 (d, *J* = 7.0 Hz, 1H, H-6″); δ = 5.9 (d, *J* = 9.3 Hz, 1H, H-1‴); δ = 5.8 (t, *J* = 8.4 Hz 1H, H-2‴); δ = 5.2 (m, 1H, H-3‴); δ = 5.5 (t, *J* = 9.2 Hz, 1H, H-4‴); δ = 5.2 (m, 1H, H-5‴); δ = 4.3 (dd, *J* = 11.8/4.0 Hz, 1H, H-6a‴); δ = 4.1 (d, *J* = 11.4 Hz, 1H, H-6b‴); δ = 2.4 (s, 3H, −CH_3_); δ = 4.0 (bs, 1H, NH); δ = 1.9–2.0 (m, 12H, acetyl CH_3_).

^13^C-NMR (100 MHz, CDCl_3_/ CD_3_OD (5:1), ppm): 161.2 (C-2), 154.6 (C-4), 105.2 (C-5), 154.6 (C-6), 134.4 (C-1′), 129.4 (C-2′), 127.2 (C-3′), 141.1 (C-4′), 127.2 (C-5′), 129.4 (C-6′), 141.1 (C-1″), 149.3 (C-3″), 129.4 (C-4″), 136.9 (C-5″), 121.7 (C-6″), 81.4 (C-1‴), 68.9 (C-2‴), 73.4 (C-3‴), 73.3 (C-4‴), 70.6 (C-5‴), 62.2 (C-6‴), 20.7–20.5 (acetyl CH_3_), 170.7/ 170.7/170.1/ 169.6 (C=O), 20.6 (−CH_3_).

Poz. LC-MS/MS m/z (%): 615 (100) [M + Na]^+^.

Anal. cal. for C_30_H_32_N_4_O_9_ (592.60 g/mol): C 60.80, H 5.44, N 9.45, found: C 60.78, H 5.48, N 9.44.

##### 1.1.17. 2,3,4,6-tetra-O-acetyl-N-[4-(4-methylphenyl)-6-pyridin-3-ylpyrimidin-2-yl]-β-D-glucopyranosylamine (17)

Yield: 87 %. White solid, M.p.: 182–184 °C. Rf: 0.50 (Diethyl ether-ethyl acetate: 1:1).

FT-IR (cm^−1^): 3421 (N-H), 2989 (−CH), 1741 (C=O), 1585 (C=N), 1542 (C=C), 1220 (C-O), 1032 (C-N).

^1^H-NMR (400 MHz, CDCl_3_/ CD_3_OD (5:1), ppm): δ = 7.8 (s, 1H, H-5); δ = 8.1 (d, *J* = 8.0 Hz, 1H, H-2′); δ = 7.3 (d, *J* = 7.8 Hz, 1H, H-3′); δ = 7.3 (d, *J* = 7.8 Hz, 1H, H-5′); δ = 8.1 (d, *J* = 8.0 Hz, 1H, H-6′); δ = 9.3 (bs, 1H, H-2″); δ = 8.7 (d, *J* = 3.8 Hz, 1H, H-4″); δ = 7.6 (t, *J* = 7.6 Hz, 1H, H-5″); δ = 8.6 (d, *J* = 7.5 Hz, 1H, H-6″); δ = 5.9 (d, *J* = 9.3 Hz, 1H, H-1‴); δ = 5.5 (t, *J* = 9.5 Hz 1H, H-2‴); δ = 5.1 (m, 1H, H-3‴); δ = 5.2 (t, *J* = 9.5 Hz, 1H, H-4‴); δ = 5.1 (m, 1H, H-5‴); δ = 4.3 (dd, *J* = 11.2/ 5.5 Hz, 1H, H-6a‴); δ = 4.2 (d, *J* = 12.2 Hz, 1H, H-6b‴); δ = 2.4 (s, 3H, −CH_3_); δ = 4.1 (bs, 1H, NH); δ = 1.9–2.0 (m, 12H, acetyl CH_3_).

^13^C-NMR (100 MHz, CDCl_3_/ CD_3_OD, ppm): 162.1 (C-2), 153.2 (C-4), 104.0 (C-5), 153.2 (C-6), 134.2 (C-1′), 127.0 (C-2′), 123.9 (C-3′), 141.3 (C-4′), 123.9 (C-5′), 127.0 (C-6′), 133.6 (C-1″), 150.3 (C-2″), 147.8 (C-4″), 129.1 (C-5″), 135.2 (C-6″), 80.8 (C-1‴), 69.1 (C-2‴), 73.9 (C-3‴), 73.1 (C-4‴), 71.1 (C-5‴), 62.3 (C-6‴), 20.0–19.2 (acetyl CH_3_), 171.0/ 170.5/ 170.4/ 170.1 (C = O), 19.1 (−CH_3_).

Poz. LC-MS/MS m/z (%):615 (100) [M + Na]^+^, 616 (92) [M +1+ Na]^+^, 593 (90) [M + 1]^+^.

Anal. cal. for C_30_H_32_N_4_O_9_ (592.60 g/mol): C 60.80, H 5.44, N 9.45, found: C 60.82, H 5.49, N 9.46.

##### 1.1.18. 2,3,4,6-tetra-O-acetyl-N-[4-(4-methylphenyl)-6-pyridin-4-ylpyrimidin-2-yl]-β-D-glucopyranosylamine (18)

Yield: 81 %. White solid, M.p.: 189–191 °C. Rf: 0.65 (Diethyl ether-ethyl acetate: 1:1).

FT-IR (cm^−1^): 3365 (N-H), 2979 (−CH), 1749 (C=O), 1579 (C=N), 1534 (C=C), 1211 (C-O), 1033 (C-N).

^1^H-NMR (400 MHz, CDCl_3_, ppm): δ = 7.6 (s, 1H, H-5); δ = 8.0 (d, *J* = 7.0 Hz, 1H, H-2′); δ = 7.3 (d, *J* = 7.4 Hz, 1H, H-3′); δ = 7.3 (d, *J* = 7.4 Hz, 1H, H-5′); δ = 8.0 (d, *J* = 7.0 Hz, 1H, H-6′); δ = 7.9 (bs, 1H, H-2″); δ = 8.8 (bs, 1H, H-3″); δ = 8.8 (bs, 1H, H-5″); δ = 7.9 (bs, 1H, H-6″); δ = 6.0 (d, *J* = 9.2 Hz, 1H, H-1‴); δ = 5.7 (t, *J* = 9.2 Hz 1H, H-2‴); δ = 5.2 (m, 1H, H-3‴); δ = 5.5 (t, *J* = 9.2 Hz, 1H, H-4‴); δ = 5.2 (m, 1H, H-5‴); δ = 4.3 (dd, *J* = 11.6/4.8 Hz, 1H, H-6a‴); δ = 4.1 (d, *J* = 12.0 Hz, 1H, H-6b‴); δ = 2.5 (s, 3H, −CH_3_); δ = 4.1 (bs, 1H, NH); δ = 1.9–2.0 (m, 12H, acetyl CH_3_).

^13^C-NMR (100 MHz, CDCl_3_, ppm): 161.5 (C-2), 144.7 (C-4), 105.1 (C-5), 144.8 (C-6), 134.0 (C-1′), 129.6 (C-2′), 127.1 (C-3′), 141.6 (C-4′), 127.1 (C-5′), 129.6 (C-6′), 141.6 (C-1″), 121.0 (C-2″), 150.6 (C-3″), 150.6 (C-5″), 121.0 (C-6″), 81.2 (C-1‴), 68.8 (C-2‴), 73.3 (C-3‴), 73.2 (C-4‴), 70.5 (C-5‴), 62.2 (C-6‴), 21.5–20.6 (acetyl CH_3_), 170.7/ 170.7/ 170.1/ 169.6 (C=O), 20.6 (−CH_3_).

Poz. LC-MS/MS m/z (%):615 (100) [M + Na]^+^.

Anal. cal. for C_30_H_32_N_4_O_9_ (592.60 g/mol): C 60.80, H 5.44, N 9.45, found: C 60.84, H 5.48, N 9.46.

#### 1.3. Pharmacology

##### 1.3.1. Preparation of cell culture

The procedure of the pharmacological experiments that include the preparation of cell culture, cell proliferation assay (MTT assay), cytotoxic activity assay, microdilution assay, and DNA binding studies are provided in supplementary information. The calculation of IC_50_ and three dose response parameters were explained in the supplementary information.

The anticancer potential of the compounds was investigated on cancerous HeLa (ATCC^®^ CCL2™), HT29 (ATCC^®^ HTB38™), MCF7 (ATCC^®^ HTB22™), A549 (ATCC^®^ CCL185™), C6 (Rat brain glioma, ATCC*^®^* CCL-107™), and Hep3B (ATCC^®^ HB8064™) and normal FL cells (ATCC^®^ CCL62™). The cell lines were cultured in a cell medium (Dulbecco’s modified eagle’s or RPMI 1640) enriched with 10% (v/v) fetal bovine serum and 2% (v/v) Penicillin-Streptomycin (10,000 U/mL). First, old medium was removed out of the flask when the cells reached approximately 80% confluence. Next, cells were taken from the flasks surface using trypsin-EDTA solution and then subjected to centrifugation. Following, the cell pellet was suspended with fresh media and was inoculated into wells.

##### 1.3.2. Cell proliferation assay (MTT assay)

A cell suspension containing approximately 1 × 10^4^ cells in 100 μL was seeded into the wells of 96-well culture plates. The cells were treated with the compounds and control drug, cis-platin and 5 fluorouracil (5FU), dissolved in sterile DMSO (max 0.5% of DMSO) at final concentrations of 1.96, 3.91, 7.81, 15.625, 31.25, 62.5, 125, and 250 μg/mL at 37 **°C with 5% CO**_2_ for overnight. The final volume of the wells was set to 200 μL by medium. Cell proliferation assay was evaluated by MTT (yellow tetrazolium MTT (3-(4,5-dimethylthiazolyl-2)-2,5-diphenyltetrazolium bromide) methods. Briefly, an MTT stock solution (5 mg of MTT/mL of distilled water) was filtered and kept at −20 **°C until use. The cells were exposed to a**n MTT reagent (consisting of one parts of MTT stock solutions and nine parts of fresh RPMI 1640 without phenol red) for 4 h to form MTT formazan dye followed by the dye dissolved in DMSO with Sorenson’s buffer for 30 min at room temperature and then the plate was measured at 560 nm, with 690 nm as a reference interval, using a microplate reader. Each experiment was repeated at least three times for each cell line.

##### 1.3.3. Cytotoxic activity assay

The cytotoxicity of the compounds, cisplatin and 5 fluorouracil on cells was determined through a Lactate Dehydrogenase Assay Kit according to the manufacturer’s instructions. Approximately 5 **×** 10^3^ cells in 100 **μ**L were placed into 96-well plates as triplicates and treated with 25, 50, 75, and 100 **μ**g/mL concentrations of test compounds at 37 **°C with 5% CO**_2_ for 24 h. LDH activity was obtained by determining absorbance at 492–630 nm using a microplate reader. The cytotoxicity assay results were noted as the percent cytotoxicity according to the following formula: % Cytotoxicity = [(Experimental Value - Low Control / High Control - Low Control) **×** 100].

##### 1.3.4. Microdilution assay

MIC values of the compounds against bacterial strains were determined on the basis of a microwell dilution method. To determine the minimal inhibitory concentration (MIC) values, inocula of bacteria were prepared using 12 h broth cultures and suspensions were adjusted to 0.5 McFarland standard turbidity. Each substance dissolved in dimethyl sulfoxide (DMSO) and serial twofold dilutions were made in a concentration range from 7.81–1000 μg/mL in microplate wells containing nutrient broth. Growth of microorganisms was determined visually after incubation for 24 h at 35 °C. The lowest concentration at which there was no visible growth (turbidity) was taken as the MIC.

##### 1.3.5. DNA binding studies

To find the interaction of the compounds with calf thymus DNA and to calculate the binding constants (*Kb*) UV–Visible absorption spectroscopy technique was used. A 2.5-mg calf thymus DNA was dissolved in 10.0 mL Tris–HCl buffer (20 mM Tris–HCl, 20 mM NaCl, pH 7.0) and stabile during one week in the refrigerator. The concentration of calf thymus DNA was obtained spectrophotometrically with help of ɛ value of 6600 M^−1^ cm^−1^ at 260 nm. After dissolving the calf thymus DNA fibers in Tris–HCl buffer, the purity of this solution was checked from the absorbance ratio A260/A280. The calf thymus DNA solution at A260/A280 ratio was equal to 1.87, implying that the DNA was pure enough. These compounds were diluted with Tris–HCl buffer to obtain 25 μM concentrations. Test compounds in the solutions were incubated at room temperature for nearly 30 min before the process. The UV-visible spectral studies were performed in mixed solvent system (1/9 DMSO/Tris–HCl buffer) using eight points that the fine structure is observed for these compounds in this system by UV-visible absorption. The UV absorption titrations were conducted by keeping the concentration of these compounds fixed while varying the CT-DNA concentrations (6.5–800 μM). Absorption spectra were recorded by using 1-cm-path quartz cuvettes at room temperature. To evaluate the interaction of the compounds with BSA, UV–Visible absorption spectroscopy technique was also used. A 2.5 mg BSA was dissolved in 10.0 mL of Tris–HCl buffer (5 mM Tris–HCl, 10 mM NaCl at pH 7.4) and stored in the refrigerator. The UV–Visible absorption spectra of the BSA solutions (6.5–800 μM) in the presence of a conserved concentration of the compounds (25 μM) were scanned in the wavelength range from 300 to 550 nm.

##### 1.3.6. Calculation of IC_50_ and % inhibition

IC_50_ value is a concentration that inhibits half of the cells in vitro. The half maximal inhibitory concentration (IC_50_) of the test and control compounds was calculated using XLfit5 or excel spreadsheet and represent in μM at 95% confidence intervals. The proliferation assay results were expressed as the percent inhibition according to the following formula: % Inhibition = [1 - (Absorbance of Treatments /Absorbance of DMSO) **×** 100]. Three dose response parameters (GI_50_, TGI, LC_50_) were calculated according to the following formulas using the absorbance measurements of time zero (Tz), control growth (C), and test growth in the presence of drug (Ti). Growth inhibition of 50% (GI_50_) was calculated from [(Ti-Tz)/(C-Tz)] **×** 100 = 50, which is the drug concentration resulting in a 50% reduction in the net growth increase in control cells during the drug incubation. The drug concentration resulting in total growth inhibition (TGI) was calculated from Ti = Tz. The LC_50_ indicating a net loss of cells following treatment was calculated from [(Ti-Tz)/Tz] **×** 100 = −50.

Figure S1 ^1^H-NMR spectrum of compound **1** (400 MHz, CDCl_3_/CD_3_OD (5:1)).

Figure S2 ^13^C-APT NMR spectrum of compound **1** (100 MHz, CDCl_3_/CD_3_OD (5:1)).

Figure S3 LC-MS/MS spectrum of compound **1**.

Figure S4 FT-IR spectrum of compound **1**.

Figure S5 ^1^H-NMR spectrum of compound **2** (400 MHz, CD_3_OD)

Figure S6 ^13^C-APT NMR spectrum of compound **2** (100 MHz, CD_3_OD)

Figure S7 LC-MS/MS spectrum of compound **2**

Figure S8 FT-IR spectrum of compound **2**

Figure S9 ^1^H-NMR spectrum of compound **3** (400 MHz, CD_3_OD).

Figure S10 ^13^C-APT NMR spectrum of compound **3** (100 MHz, CD_3_OD).

Figure S11 LC-MS/MS spectrum of compound **3**.

Figure S12 FT-IR spectrum of compound **3**.

Figure S13 ^1^H-NMR spectrum of compound **4** (400 MHz, CDCl_3_/CD_3_OD (5:1)).

Figure S14 ^13^C-APT NMR spectrum of compound **4** (100 MHz, CDCl_3_/CD_3_OD (5:1)).

Figure S15 LC-MS/MS spectrum of compound **4**.

Figure S16 FT-IR spectrum of compound **4**.

Figure S17 ^1^H-NMR spectrum of compound **5** (400 MHz, CDCl_3_/CD_3_OD (5:1)).

Figure S18 ^13^C-APT NMR spectrum of compound **5** (100 MHz, DMSO-d_6_).

Figure S19 LC-MS/MS spectrum of compound **5**.

Figure S20 FT-IR spectrum of compound **5**.

Figure S21 ^1^H-NMR spectrum of compound **6** (400 MHz, CDCl_3_/CD_3_OD (5:1)).

Figure S22 ^13^C-APT NMR spectrum of compound **6** (100 MHz, CD_3_OD).

Figure S23 LC-MS/MS spectrum of compound **6**.

Figure S24 FT-IR spectrum of compound **6**.

Figure S25 ^1^H-NMR spectrum of compound **7** (400 MHz, CDCl_3_/CD_3_OD (5:1)).

Figure S26 ^13^C-APT NMR spectrum of compound **7** (100 MHz, CDCl_3_/CD_3_OD (5:1)).

Figure S27 LC-MS/MS spectrum of compound **7**.

Figure S28 FT-IR spectrum of compound **7**.

Figure S29 ^1^H-NMR spectrum of compound **8** (400 MHz, CD_3_OD).

Figure S30 ^13^C-APT NMR spectrum of compound **8** (100 MHz, DMSO-d_6_).

Figure S31 LC-MS/MS spectrum of compound **8**.

Figure S32 FT-IR spectrum of compound **8**.

Figure S33 ^1^H-NMR spectrum of compound **9** (400 MHz, CD_3_OD).

Figure S34 ^13^C-APT NMR spectrum of compound **9** (100 MHz, CD_3_OD).

Figure S35 LC-MS/MS spectrum of compound **9**.

Figure S36 FT-IR spectrum of compound **9**.

Figure S37 ^1^H-NMR spectrum of compound **10** (400 MHz, CDCl_3_, ppm).

Figure S38 ^13^C-APT NMR spectrum of compound **10** (100 MHz, CDCl_3_, ppm).

Figure S39 LC-MS/MS spectrum of compound **10**.

Figure S40 FT-IR spectrum of compound **10**.

Figure S41 ^1^H-NMR spectrum of compound **11** (400 MHz, CDCl_3_, ppm).

Figure S42 ^13^C-APT NMR spectrum of compound **11** (100 MHz, CDCl_3_, ppm).

Figure S43 LC-MS/MS spectrum of compound **11**.

Figure S44 FT-IR spectrum of compound **11**.

Figure S45 ^1^H-NMR spectrum of compound **12** (400 MHz, CDCl_3_, ppm).

Figure S46 ^13^C-APT NMR spectrum of compound **12** (100 MHz, CDCl_3_, ppm).

Figure S47 LC-MS/MS spectrum of compound **12**.

Figure S48 FT-IR spectrum of compound **12**.

Figure S49 ^1^H-NMR spectrum of compound **13** (400 MHz, CDCl_3_, ppm).

Figure S50 ^13^C-APT NMR spectrum of compound **13** (100 MHz, CDCl_3_, ppm).

Figure S51 LC-MS/MS spectrum of compound **13**.

Figure S52 FT-IR spectrum of compound **13**.

Figure S53 ^1^H-NMR spectrum of compound **14** (400 MHz, CDCl_3_, ppm).

Figure S54 ^13^C-APT NMR spectrum of compound **14** (100 MHz, CDCl_3_, ppm).

Figure S55 LC-MS/MS spectrum of compound **14**.

Figure S56 FT-IR spectrum of compound **14**.

Figure S57 ^1^H-NMR spectrum of compound **15** (400 MHz, CDCl_3_, ppm).

Figure S58 ^13^C-APT NMR spectrum of compound **15** (100 MHz, CDCl_3_, ppm).

Figure S59 LC-MS/MS spectrum of compound **15**.

Figure S60 FT-IR spectrum of compound **15**.

Figure S61 ^1^H-NMR spectrum of compound **16** (400 MHz, CDCl_3_/CD_3_OD (5:1), ppm).

Figure S62 ^13^C-APT NMR spectrum of compound **16** (100 MHz, CDCl_3_/CD_3_OD (5:1), ppm).

Figure S63 LC-MS/MS spectrum of compound **16**.

Figure S64 FT-IR spectrum of compound **16**.

Figure S65 ^1^H-NMR spectrum of compound **17** (400 MHz, CDCl_3_/CD_3_OD (5:1), ppm).

Figure S66 ^13^C-APT NMR spectrum of compound **17** (100 MHz, CDCl_3_, ppm).

Figure S67 LC-MS/MS spectrum of compound **17**.

Figure S68 FT-IR spectrum of compound **17**.

Figure S69 ^1^H-NMR spectrum of compound **18** (400 MHz, CDCl_3_, ppm).

Figure S70 ^13^C-APT NMR spectrum of compound **18** (100 MHz, CDCl_3_, ppm).

Figure S71 LC-MS/MS spectrum of compound **18**.

Figure S72 FT-IR spectrum of compound **18**.

Figure S73 UV–Visible absorption spectra of 25 μM these compounds in the absence (a) and presence of 6.25 μM (b), 12.5 μM (c), 25 μM (d) 50 μM (e), 100 μM (f), 200 μM (g), 400 μM (h), and 800 μM (i) DNA. Note: The direction of arrow demonstrates increasing concentrations of DNA. Inside graph is the plot of [DNA] versus [DNA]/***ɛa*** – ***ɛf*** to find the binding constant of complex–DNA adduct.

Figure S74 UV–Visible absorption spectra of 25 μM these compounds in the absence (a) and presence of 6.25 μM (b), 12.5 μM (c), 25 μM (d) 50 μM (e), 100 μM (f), 200 μM (g), 400 μM (h) and 800 μM (i) BSA.

## Figures and Tables

**Scheme:**
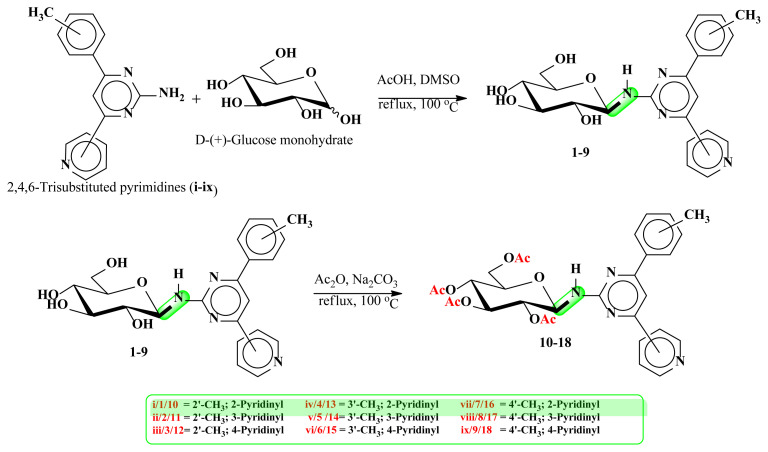
Consecutive synthesis of pyrimidine-N-glycosides (**1–18**).

**Table 1:** GI_50_, TGI, LC_50_ and IC_50_ values for **1–**18 against C6, HeLa, and HT29.

Compound (μg/mL)	C6				HeLa				HT29			
	GI_50_	TGI	LC_50_	IC_50_	GI_50_	TGI	LC_50_	IC_50_	GI_50_	TGI	LC_50_	IC_50_
1	6.34	24.27	245.39	41.21	10.51	35.15	218.36	54.38	3.41	>500	>500	197.80
**2**	9.17	31.57	216.51	49.71	>500	>500	>500	>500	2.46	>500	>500	452.79
**3**	4.37	17.41	251.95	33.49	>500	>500	>500	>500	2.22	230.10	>500	78.38
**4**	8.13	20.46	77.38	27.78	21.92	46.04	115.58	52.22	4.63	31.94	>500	26.69
**5**	4.56	18.06	249.03	33.50	>500	>500	>500	>500	4.12	126.23	>500	72.62
**6**	>500	>500	>500	>500	>500	>500	>500	>500	4.29	>500	>500	>500
**7**	8.15	21.46	88.30	29.27	7.17	21.60	120.40	31.85	2.50	49.16	>500	29.91
**8**	8.71	30.70	225.15	48.97	14.11	50.07	325.09	81.26	4.43	60.05	>500	42.49
**9**	>500	>500	>500	>500	>500	>500	>500	>500	4.30	>500	>500	>500
**1**0	2.21	9.19	486.04	21.89	>500	>500	>500	>500	2.10	>500	>500	71.11
**11**	2.10	8.83	577.93	21.86	>500	>500	>500	>500	2.75	>500	>500	>500
**12**	2.03	6.49	138.62	12.98	>500	>500	>500	>500	2.23	>500	>500	297.89
**13**	1.97	4.70	33.89	7.55	4.48	17.29	224.19	60.37	2.94	>500	>500	>500
**14**	>500	>500	>500	>500	>500	>500	>500	>500	2.28	>500	>500	>500
**15**	2.21	6.30	71.25	11.23	>500	>500	>500	>500	5.00	>500	>500	>500
**16**	1.40	2.80	22.85	4.38	1.49	3.28	34.11	9.13	1.47	3.88	121.77	3.26
**17**	>500	>500	>500	>500	>500	>500	>500	>500	3.09	>500	>500	>500
**18**	2.23	5.21	29.75	30.15	1.03	1.29	7.40	2.29	7.77	>500	>500	>500
**Cisplatin**				33.08				50.29				40.39
**5FU**				54.30				61.59				65.19

**Table 2:** GI_50_, TGI, LC_50_, and IC_50_ values for **1–18** against MCF7, A549, Hep3B, and FL.

Compound (μg/mL)	MCF7				A549				Hep3B				FL			
	GI_50_	TGI	LC_50_	IC_50_	GI_50_	TGI	LC_50_	IC_50_	GI_50_	TGI	LC_50_	IC_50_	GI_50_	TGI	LC_50_	IC_50_
1	1.81	>500	>500	>500	67.79	>500	>500	>500	5.38	>500	>500	>500	3.10	81.60	>500	77.38
**2**	1.69	>500	>500	>500	11.57	>500	>500	>500	3.85	>500	>500	>500	3.68	>500	>500	456.69
**3**	2.23	>500	>500	>500	6.49	>500	>500	>500	3.83	>500	>500	>500	6.56	>500	>500	>500
**4**	3.44	>500	>500	>500	19.45	>500	>500	>500	4.40	67.85	>500	65.54	6.77	79.78	>500	78.11
**5**	2.53	75.66	>500	66.84	23.69	>500	>500	>500	3.61	20.53	>500	20.10	4.16	25.82	>500	25.34
**6**	2.29	>500	>500	>500	192.15	>500	>500	>500	4.07	>500	>500	>500	8.04	>500	>500	>500
**7**	2.04	>500	>500	>500	125.31	>500	>500	>500	4.26	193.81	>500	182.57	6.10	266.05	>500	254.76
**8**	1.70	>500	>500	>500	>500	>500	>500	>500	6.38	>500	>500	>500	5.64	>500	>500	>500
**9**	1.89	>500	>500	>500	>500	>500	>500	>500	4.61	>500	>500	>500	7.04	>500	>500	>500
**1**0	1.66	>500	>500	>500	>500	>500	>500	>500	7.53	>500	>500	>500	22.76	>500	>500	>500
**11**	4.46	>500	>500	>500	>500	>500	>500	>500	4.25	>500	>500	>500	11.16	>500	>500	>500
**12**	1.22	>500	>500	>500	>500	>500	>500	>500	4.88	>500	>500	>500	3.68	>500	>500	>500
**13**	1.84	397.22	>500	296.56	>500	>500	>500	>500	2.56	>500	>500	>500	1.63	19.08	>500	18.19
**14**	1.95	>500	>500	>500	430.48	>500	>500	>500	4.90	>500	>500	>500	117.77	>500	>500	>500
**15**	4.10	>500	>500	>500	>500	>500	>500	>500	5.55	>500	>500	>500	26.66	>500	>500	>500
**16**	1.41	4.29	495.06	4.12	23.79	>500	>500	>500	1.99	7.33	326.12	7.18	1.71	6.44	>500	6.31
**17**	2.19	>500	>500	>500	7.85	>500	>500	>500	3.86	>500	>500	>500	17.18	>500	>500	>500
**18**	1.79	>500	>500	>500	15.54	>500	>500	>500	6.15	>500	>500	>500	2.01	7.58	352.27	7.46
**Cisplatin**				63.79				60.49				48.69				52.79
**5FU**				74.19				69.79				62.89				59.09

**Table 3:** % cytotoxicity of **1–18** at various concentrations against C6, HeLa, and HT29.

Compound *(μg/mL)*	C6				HeLa				HT29			
	25	50	75	100	25	50	75	100	25	50	75	100
**1**	28.69	28.69	72.07	97.85	30.21	46.99	65.29	88.73	29.32	48.01	68.90	83.66
**2**	30.84	49.84	72.70	99.75	30.53	47.25	49.72	72.13	27.74	44.78	72.70	94.62
**3**	25.27	32.11	73.97	99.68	16.28	33.38	40.60	56.87	33.12	49.91	71.12	98.73
**4**	22.10	49.27	68.90	89.17	26.92	64.28	77.14	97.15	28.18	35.72	62.57	74.92
**5**	26.92	44.08	62.57	87.14	19.82	35.34	40.34	54.27	24.64	39.39	75.87	96.77
**6**	27.93	38.25	63.14	84.86	14.31	29.20	44.52	47.75	24.83	34.45	68.90	85.62
**7**	21.98	48.58	69.54	85.75	11.91	25.14	38.44	50.66	25.97	40.66	75.87	91.83
**8**	28.37	45.41	63.20	88.73	12.54	34.96	41.99	48.96	20.58	34.45	75.81	95.88
**9**	28.37	34.07	70.17	90.56	10.01	34.33	49.65	55.92	25.71	40.85	74.73	81.76
**10**	22.80	54.34	77.14	93.54	16.78	40.72	51.68	58.39	29.45	51.80	64.47	95.19
**11**	26.35	42.24	76.95	84.80	17.23	29.83	44.97	49.78	36.10	49.34	63.84	90.56
**12**	21.15	46.74	75.17	87.40	18.81	30.84	42.62	49.59	30.72	41.86	62.57	77.64
**13**	15.01	51.17	77.14	98.35	18.11	27.99	42.75	50.92	31.67	45.47	66.37	79.23
**14**	18.43	35.34	70.17	86.70	16.59	28.82	33.50	48.77	21.03	44.65	61.81	72.20
**15**	20.39	54.97	77.14	83.28	18.43	31.73	37.11	55.98	23.81	45.03	62.57	78.59
**16**	17.10	28.94	63.20	84.74	35.66	62.57	77.14	99.24	26.41	34.39	48.64	70.23
**17**	32.05	44.65	67.64	81.82	16.66	23.81	32.36	48.58	21.66	35.78	51.04	75.74
**18**	27.23	44.14	75.24	86.45	14.25	38.95	46.11	54.21	27.87	45.47	58.14	82.84
**Cisplatin**	9.04				9.85				11.23			
**5FU**	10.01				8.83				7.91			

**Table 4:** % cytotoxicity of **4–9** at various concentrations against A549, Hep3B, MCF7, and FL.

Compound *(μg/mL)*	A549				Hep3B				MCF7			FL				
	25	50	75	100	25	50	75	100	25	50	75	100	25	50	75	100
4	26.03	33.25	61.68	72.51	19.57	28.94	42.05	64.79	11.59	22.48	29.96	40.22	24.45	32.43	48.26	69.47
**5**	24.38	31.29	51.55	66.31	14.12	25.52	42.75	52.50	8.23	12.22	24.95	36.42	16.59	32.93	62.57	76.76
**6**	26.98	31.98	48.07	72.83	10.01	15.45	35.85	41.10	7.22	12.48	31.79	41.86	19.13	27.55	50.85	63.39
**7**	24.32	24.38	27.42	40.60	9.37	17.04	34.26	44.52	4.05	20.14	26.60	40.72	18.11	30.53	46.55	73.21
**8**	28.12	25.97	47.44	58.14	15.64	20.52	36.16	52.94	10.01	20.14	32.81	43.45	20.84	34.14	40.98	69.92
**9**	27.42	28.50	48.39	63.20	16.53	24.38	41.48	49.40	11.08	19.19	27.49	36.54	18.56	30.02	60.61	70.99
**Cisplat**in	8.63				8.46				10.71				8.33			
**5FU**	9.19				9.67				7.69				8.44			

**Table 5:** IC_50_ of pyrimidine-coupled *N*-β-glycosides (**1–**9) against the cell lines.

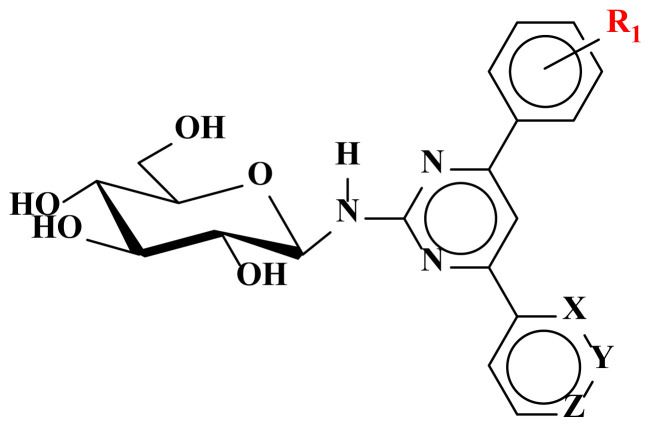											
Compounds	Functional Groups				IC_50_ (μg/mL)						
	R1	X	Y	Z	C6	HeLa	HT29	MCF7	A549	Hep3B	FL
**1**	2′-CH_3_	N	H	H	41.21	54.38	197.80	>500	>500	>500	77.38
**2**	2′-CH_3_	H	N	H	49.71	>500	452.79	>500	>500	>500	456.69
**3**	2′-CH_3_	H	H	N	33.49	>500	78.38	>500	>500	>500	>500
**4**	3′-_C_H_3_	N	H	H	27.78	52.22	26.69	>500	>500	65.54	78.11
**5**	3′-CH_3_	H	N	H	33.50	>500	72.62	66.84	>500	20.10	25.34
**6**	3′-CH_3_	H	H	N	>500	>500	>500	>500	>500	>500	>500
**7**	4′-^C^H_3_	N	H	H	29.27	31.85	29.91	>500	>500	182.57	254.76
**8**	4′-CH_3_	H	N	H	48.97	81.26	42.49	>500	>500	>500	>500
**9**	4′-CH_3_	H	H	N	>500	>500	>500	>500	>500	>500	>500

**Table 6:** IC_50_ of tetra-*O*-acetyl derivatives of pyrimidine-coupled *N*-β-glycosides (**10–**18) against the cell lines.

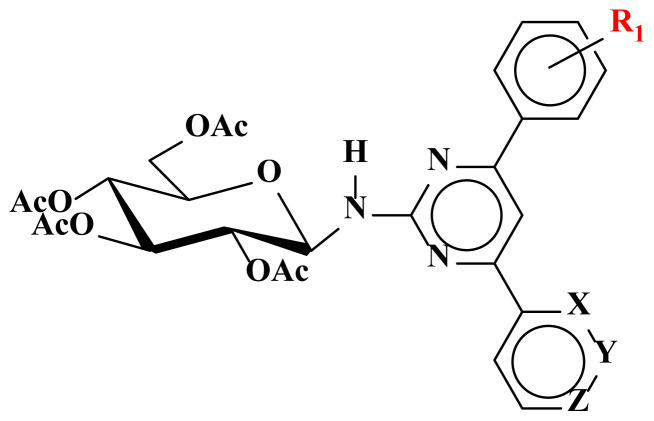											
Compound	Functional Groups				IC_50_ (μg/mL)						
	R1	X	Y	Z	C6	HeLa	HT29	MCF7	A549	Hep3B	FL
**10**	2′-CH_3_	N	H	H	21.89	>500	71.11	>500	>500	>500	>500
**11**	2′-CH_3_	H	N	H	21.86	>500	>500	>500	>500	>500	>500
**12**	2′-CH_3_	H	H	N	12.98	>500	297.89	>500	>500	>500	>500
**13**	3′-CH_3_	N	H	H	7.55	60.37	>500	296.56	>500	>500	18.19
**14**	3′-CH_3_	H	N	H	>500	>500	>500	>500	>500	>500	>500
**15**	3′-CH_3_	H	H	N	11.23	>500	>500	>500	>500	>500	>500
**16**	4′-CH_3_	N	H	H	4.38	9.13	3.26	4.12	>500	7.18	6.31
**17**	4′-CH_3_	H	N	H	>500	>500	>500	>500	>500	>500	>500
**18**	4′-CH_3_	H	H	N	30.15	2.29	>500	>500	>500	>500	7.46

**Table 7:** Minimum-inhibitory concentrations (MIC, in μg/mL) of **1–9**.

Microorganisms	MIC (μg/mL)										
	1	2	3	4	5	6	7	8	9	KCN	SCF
*E. faecalis* **VRE** ATCC 19433	>1000	>1000	>1000	>1000	>1000	1000	1000	500	1000	NE	250
*E. faecalis* ATCC 29212	1000	>1000	>1000	250	>1000	1000	250	1000	1000	NE	62.5
*S. aureus* ATCC 25923	500	125	500	250	125	500	250	15.62	1000	NE	250
*S. aureus* **MSSA** ATCC 29213	500	125	1000	250	125	125	250	62.5	125	NE	NA
*S. aureus* **MRSA** ATCC 46300	1000	125	1000	500	250	250	250	<7.81	1000	NE	250
*E. coli* ATCC 25922	>1000	>1000	>1000	>1000	>1000	>1000	>1000	1000	>1000	NE	15.62
*E. coli* **ESBL** ATCC 35218	>1000	>1000	>1000	>1000	>1000	>1000	>1000	>1000	>1000	NE	31.25
*P. aeruginosa* **AGME** ATCC 27853	>1000	>1000	>1000	>1000	>1000	>1000	>1000	>1000	>1000	NE	250
*S. mutans* ATCC 35668	62.5	1000	500	125	500	500	125	250	250	NE	125
*S. gordonii* NCTC 7870	250	500	1000	250	125	250	500	250	1000	NE	125
*A. actinomycetemcomitans* ATCC 33384	500	250	500	250	250	500	250	500	500	NE	62.5

SCF: sulbactam (30 μg) + cefoperazone (75 μg), as a positive control KCN: potassium cyanide, as a negative control, NE: No effect. NA: Not available

**Table 8:** Minimum-inhibitory concentrations (MIC, in μg/mL) of **10–18**.

Microorganisms	MIC (μg/mL)										
	10	11	12	13	14	15	16	17	18	KCN	SCF
*E. faecalis* **VRE** ATCC 19433	125	>1000	1000	1000	1000	1000	1000	1000	1000	NE	250
*E. faecalis* ATCC 29212	500	125	250	31.25	31.25	31.25	125	125	125	NE	62.5
*S. aureus* ATCC 25923	>1000	>1000	>1000	500	500	500	>1000	>1000	500	NE	250
*S. aureus* **MSSA** ATCC 29213	>1000	125	1000	125	125	250	1000	125	1000	NE	NA
*S. aureus* **MRSA** ATCC 46300	1000	1000	500	500	250	250	>1000	1000	500	NE	250
*E. coli* ATCC 25922	>1000	>1000	>1000	>1000	>1000	>1000	>1000	>1000	>1000	NE	15.62
*E. coli* **ESBL** ATCC 35218	>1000	>1000	>1000	>1000	>1000	>1000	>1000	>1000	>1000	NE	31.25
*P. aeruginosa* **AGME** ATCC 27853	>1000	>1000	>1000	>1000	>1000	>1000	>1000	>1000	>1000	NE	250
*S. mutans* ATCC 35668	500	250	62.5	125	62.5	62.5	125	125	125	NE	125
*S. gordonii* NCTC 7870	>1000	>1000	>1000	>1000	>1000	>1000	>1000	>1000	>1000	NE	125
*A. actinomycetemcomitans* ATCC 33384	>1000	>1000	>1000	>1000	>1000	>1000	>1000	>1000	>1000	NE	62.5

SCF: sulbactam (30 μg) + cefoperazone (75 μg), as a positive control

KCN: potassium cyanide, as a negative control, NE: No effect. NA: Not available

**Tablo 9:** The binding constants (K_b_) of compounds.

No	K_b_ (M^−1^)	No	K_b_ (M^−1^)
**1**	7.1 × 10^4^	**10**	4.9 × 10^4^
**2**	1.4 × 10^4^	**11**	4.0 × 10^4^
**3**	1.2 × 10^4^	**12**	1.9 × 10^4^
**4**	3.5 × 10^4^	**13**	3.7 × 10^4^
**5**	2.6 × 10^4^	**14**	2.4 × 10^4^
**6**	4.4 × 10^2^	**15**	2.7 × 10^4^
**7**	4.2 × 10^4^	**16**	3.7 × ^1^04
**8**	2.4 × 10^2^	**17**	3.4 × 10^4^
**9**	1.2 × 10^4^	**18**	5.2 × 10^4^

**Table 10:** MM-GBSA ΔG_Bind_, docking score and complex energy values of compounds **4, 7, 13**, and **16** interacting with different identified proteins.

Protein	PDB ID	4			7			13			16		
		MM-GBSA ΔG_Bind_	Docking score	Complex energy	MM-GBSA ΔG_Bind_	Docking score	Complex energy	MM-GBSA ΔG_Bind_	Docking score	Complex energy	MM-GBSA ΔG_Bind_	Docking score	Complex energy
**KRAS**	***4QL3***	−41.82	−4.584	−8017.943	−47.29	−3.637	−8031.976	−50.23	−3.459	−8114.199	−66.75	−4.022	−8135.716
**NRAS**	***6MPP***	**−65.37**	**−5.955**	**−12780.426**	**−59.62**	**−5.979**	**−12779.884**	**−72.17**	**−5.605**	**−12873.728**	**−71.78**	**−5.178**	**−12879.108**
**BRAF**	***4E26***	**−61.07**	**−7.443**	**−11675.540**	**−73.54**	**−8.034**	**−11680.540**	**−79.49**	**−7.459**	**−11775.205**	**−62.39**	**−5.379**	**−11756.811**
**Bcl–2**	***6QGG***	−56.04	−4.136	−6675.966	−55.63	−5.433	−6680.625	−50.49	−3.678	−6756.208	−57.31	−3.927	−6769.754
**TNF–alfa**	***5MU8***	−46.68	−3.994	−5942.692	−39.64	−3.549	−5935.130	−57.20	−4.230	−6036.630	−48.31	−4.180	−6030.498
**Caspase 3**	***4QUG***	−44.35	−4.868	−10640.208	−52.53	−5.622	−10648.018	−66.79	−4.299	−10753.002	−59.44	−4.703	−10746.972
**NF–kB p105 subunit**	***2DBF***	−34.90	−3.924	−4178.232	−51.98	−4.439	−4190.774	−29.74	−2.398	−4261.872	−36.06	−2.807	−4269.187
**p53**	***6SL6***	−30.28	−3.130	−8809.743	−48.09	−4.472	−8830.267	−41.55	−2.164	−8910.086	−44.08	−2.195	−8913.086
**Fibroblast collagenase**	***1CGL***	−44.25	−4.913	−7363.285	−47.77	−5.623	−7364.559	−46.86	−4.264	−7451.086	−53.44	−4.618	−7462.722
**Cytochromec oxidase**	***5Z62***	**−67.86**	**−7.306**	**−19700.406**	**−72.35**	**−7.643**	**−19700.590**	−78.31	3.558	−19773.817	−48.69	1.230	−19764.553
**BAX W139A**	***6EB6***	−49.01	−4.498	−7774.971	−45.50	−4.590	−7772.048	−57.21	−3.682	−7874.098	−58.99	−3.536	−7881.547
**PI3K alpha**	***5ITD***	**−61.96**	**−7.265**	**−43523.368**	**−62.40**	**−7.328**	**−43526.159**	**−78.69**	**−6.754**	**−43635.851**	**−58.34**	**−4.641**	**−43615.402**
**Akt1**	***4EKK***	**−54.84**	**−6.141**	**−14235.655**	**−43.09**	**−5.635**	**−14217.350**	**−58.54**	**−5.430**	**−14327.489**	**−51.71**	**−3.587**	**−14321.026**
**Cdk1**	***6GU7***	−51.24	−6.045	−12061.439	−45.52	−5.468	−12059.532	−62.79	−5.267	−12167.405	−49.79	−4.048	−12157.146
**Tert**	***7BG9***	−40.50	−4.475	−34228.139	−41.37	−3.698	−34231.456	−39.16	−3.298	−34311.267	−49.49	−3.389	−34322.747
**Gstp1**	***3GUS***	−65.77	−5.681	−9438.680	−61.99	−4.657	−9440.069	−65.46	−5.223	−9534.460	−68.34	−5.931	−9538.395

## References

[1] Kumar S Lim SM Ramasamy K Vasudevan M Shah SAA Synthesis, molecular docking and biological evaluation of bis-pyrimidine Schiff base derivatives Chemistry Central Journal 2017 11 1 16 10.1186/s13065-017-0322-0 29086867 PMC5603458

[2] Kocyigit UM Budak Y Gürdere MB Ertürk F Yencilek B Synthesis of chalcone-imide derivatives and investigation of their anticancer and antimicrobial activities, carbonic anhydrase and acetylcholinesterase enzymes inhibition profiles Archives of Physiology and Biochemistry 2018 124 61 68 10.1080/13813455.2017.1360914 28792233

[3] Gao L Liu Q Ren S Wan S Jiang T Synthesis of a novel series of (e,e)-4,6–bis(styryl)-2-*O*-glucopyranosyl-pyrimidines and their potent multidrug resistance (mdr) reversal activity against cancer cells Journal of Carbohydrate Chemistry 2012 31 620 633 10.1080/07328303.2012.689041

[4] Prasad S Radhakrishna V Ravi TK Synthesis, spectroscopic and antibacterial studies of some schiff bases of 4-(4-bromophenyl)-6-(4-chlorophenyl)-2-aminopyrimidine Arabian Journal of Chemistry 2016 12 3943 3947 10.1016/j.arabjc.2016.03.003

[5] Parsonnet J Bacterial infection as a cause of cancer Environmental Health Perspectives 1995 103 263 268 10.2307/3432323 8741796 PMC1518971

[6] Hullar MAJ Burnett-Hartman AN Lampe JW Gut microbes, diet, and cancer Cancer Research and Treatment 2014 159 377 399 10.1007/978-3-642-38007-5_22 PMC4121395 24114492

[7] Jimenez J Chakraborty I Rojas-Andrade M Mascharak PK Silver complexes of ligands derived from adamantylamines: Water-soluble silver-donating compounds with antibacterial properties Journal of Inorganic Biochemistry 2017 168 13 17 10.1016/j.jinorgbio.2016.12.009 27997857 PMC5728992

[8] Sirajuddin M Ali S McKee V Sohail M Pasha H Potentially bioactive organotin (IV) compounds: synthesis, characterization, in vitro bioactivities and interaction with SS-DNA European Journal of Medicinal Chemistry 2014 84 343 363 10.1016/j.ejmech.2014.07.028 25036793

[9] Fosso MY Nziko VPN Chang CWT Chemical synthesis of *N*-aryl glycosides Journal of Carbohydrate Chemistry 2021 31 603 619 10.1080/07328303.2012.699575

[10] Hamadi NB Msaddek M Synthesis and reactivity of N-sugar-maleimides: an access to novel highly substituted enantiopure pyrazolines Tetrahedron Asymmetry 2012 23 1689 1693 10.1016/j.tetasy.2012.11.005

[11] Liu YY Shi H He GK Song GL Zhu HJ Synthesis, crystal structures, and fungicidal activity of novel 1,5-diaryl-3-(glucopyranosyloxy)-1H-pyrazoles Helvetica Chimica Acta 2012 95 1645 1656 10.1002/hlca.201100509

[12] Hemamalini A Nagarajan S Das TM A facile synthesis of sugar-pyrazole derivatives Carbohydrate Research 2011 346 1814 1819 10.1016/j.carres.2011.06.019 21784420

[13] Yin X Zheng L Li Y Yin S Synthesis and calming activity of 2-amino-4-(4-*β*-d-allopyranoside-phenyl)-6–3(4)-substituted phenylpyrimidines Chemistry of Natural Compounds 2010 46 779 782 10.1007/s10600-010-9739-6

[14] Çelik G New chalcone-3-O-glycoside derivatives: Synthesis and characterization Journal of Chemical Research 2020 44 598 601 10.1177/1747519820915165

[15] Chamberlain SD Moorman AR Burnette TC de Miranda P Krenitsky TA Novel carbohydrate conjugates as potential prodrugs of acyclovir Antiviral Chemistry and Chemotherapy 1994 5 64 73

[16] Wang Y Yao H Hua M Jiao Y He H Direct *N*-glycosylation of amides/amines with glycal donors The Journal of Organic Chemistry 2020 85 7485 7493 10.1021/acs.joc.0c00975 32400156

[17] Alwan WS Karpoormath R Palkar MB Patel HM Rane RA Novel imidazo[2,1-*b*]-1,3,4-thiadiazoles as promising antifungal agents against clinical isolate of *Cryptococcus neoformans* European Journal of Medicinal Chemistry 2015 95 514 525 10.1016/j.ejmech.2015.03.021 25847769

[18] Garg HG von dem Bruch K Kunz H Developments in the synthesis of glycopeptides containing glycosyl L-asparagine, L-serine, and L-threonine Advances in Carbohydrate Chemistry and Biochemistry 1994 50 277 310 https://10.1016/s0065-2318(08)60153-5 7942256 10.1016/s0065-2318(08)60153-5

[19] Schmidt RR Kinzy W Anomeric-oxygen activation for glycoside synthesis: the trichloroacetimidate method Advances in Carbohydrate Chemistry and Biochemistry 1994 50 21 123 https://doi:10.1016/s0065-2318(08)60150-x 7942254 10.1016/s0065-2318(08)60150-x

[20] Váradi A Lévai D Tóth G Horváth P Noszál B Hosztafi S Glucosides of morphine derivatives: synthesis and characterization Monatshefte fur Chemie 2013 144 255 262 10.1007/s00706-012-0868-4

[21] Yamazoe A Hayashi K Kuboki A Ohira S Nozaki H The isolation, structural determination, and total synthesis of terfestatin A, a novel auxin signaling inhibitör from Streptomyces sp Tetrahedron Letters 2004 5 8359 8362 10.1016/j.tetlet.2004.09.055

[22] Wang Q Duan J Tang P Chen G He G Synthesis of non-classical heteroaryl C-glycosides *via* Minisci-type alkylation of *N*-heteroarenes with 4-glycosyl-dihydropyridines Science China Chemistry 2020 63 1613 1618 10.1007/s11426-020-9813-5

[23] Kahriman N Serdaroğlu V Peker K Aydın A Usta A Synthesis and biological evaluation of new 2,4,6-trisubstituted pyrimidines and their *N*-alkyl derivatives Bioorganic Chemistry 2019 83 580 594 10.1016/j.bioorg.2018.10.068 30471580

[24] Kahriman N Peker K Serdaroğlu V Aydın A Usta A Novel 2-amino-4-aryl-6-pyridopyrimidines and *N*-alkyl derivatives: Synthesis, characterization and investigation of anticancer, antibacterial activities and DNA/BSA binding affinities Bioorganic Chemistry 2020 99 103805 10.1016/j.bioorg.2020.103805 32272366

[25] Xiang S Ma J Gorityala BK Liu X-W Stereoselective synthesis of b-N-glycosides through 2-deoxy-2-nitroglycal Carbohydrate Research 2011 346 2957 2959 10.1016/j.carres.2011.01.032 22030462

[26] Chen B Liu Y Liu H-W Wang N-L Yang B-F Iridoid and aromatic glycosides from *Scrophularia ningpoensis* Hemsl. and their inhibition of [Ca^2+^]*_i_* increase induced by KCl Chemistry & Biodiversity 2008 5 1723 1735 10.1002/cbdv.200890161 18816525

[27] Bubb WA NMR spectroscopy in the study of carbohydrates: Characterizing the structural complexity Concepts in Magnetic Resonance Part A 2003 19A 1 1 19 10.1002/cmr.a.10080

[28] Kataev VE Strobykina IYu Andreeva OV Garifullin BF Sharipova RR Synthesis and antituberculosis activity of derivatives of *Stevia rebaudiana* glycoside steviolbioside and diterpenoid isosteviol containing hydrazone, hydrazide, and pyridinoyl moieties Russian Journal of Bioorganic Chemistry 2011 37 483 491 10.1134/S1068162011030095 22096997

[29] Klevens RM Morrison MA Nadle J Petit S Gershman K Invasive Methicillin-resistant *Staphylococcus aureus* infections in the United States The Journal of the American Medical Association 2007 298 1763 1771 10.1001/jama.298.15.1763 17940231

[30] Sirajuddin M Ali S Badshah A Drug-DNA interactions and their study by UV-Visible, fluorescence spectroscopies and cyclic voltammetry Journal of Photochemistry and Photobiology B: Biology 2013 124 1 19 10.1016/j.jphotobiol.2013.03.013 23648795

[31] Pyle AM Rehmann JP Meshoyrer R Kumar CV Turro NJ Mixed-ligand complexes of ruthenium(II): factors governing binding to DNA Journal of the American Chemical Society 1989 111 3051 3058 10.1021/ja00190a046

[32] N’soukpoé-Kossi CN Descôteaux C Asselin E Tajmir-Riahi HA Bérubé G DNA interaction with novel antitumor estradiol-platinum(II) hybrid molecule: A comparative study with cisplatin drug DNA and Cell Biology 2008 27 101 1077 10.1089/dna.2007.0669 17970617

[33] Jangir DK Charak S Mehrotra R Kundu S FTIR and circular dichroism spectroscopic study of interaction of 5-fluorouracil with DNA Journal of Photochemistry and Photobiology B: Biology 2011 105 143 148 10.1016/j.jphotobiol.2011.08.003 21940176

[34] Aydın A Korkmaz N Kısa D Türkmenoğlu B Karadağ A Dicyanoargentate(I)-based complexes induced in vivo tumor inhibition by activating apoptosis-related pathways Applied Organometallic Chemistry 2022 36 e6844 10.1002/aoc.6844

[35] Yaylı N Kılıç G Çelik G Kahriman N Kanpolat Ş Synthesis of hydroxy benzoin/benzil analogs and investigation of their antioxidant, antimicrobial, enzyme inhibition, and cytotoxic activities Turkish Journal of Chemistry 2021 45 788 804 10.3906/kim-2012-25 PMC10454678 37635901

[36] Schrödinger Release 2021-2 Glide S, LLC New York, NY 2021

[37] Schrödinger Release 2021 2 LigPrep S, LLC New York, NY 2021

[38] Merde İB Önel GT Türkmenoğlu B Gürsoy Ş Dilek E Synthesis of (p-tolyl)-3 (2H) pyridazinone derivatives as novel Acetylcholinesterase inhibitors ChemistrySelect 2022 7 e202201606 10.1002/slct.202201606

[39] Türkmenoğlu B Investigation of novel compounds via in silico approaches of EGFR inhibitors as anticancer agents Journal of the Indian Chemical Society 2022 99 100601 10.1016/j.jics.2022.100601

[40] Hunter JC Manandhar A Carrasco MA Gurbani D Gondi S Biochemical and structural analysis of common cancer-associated KRAS mutations Molecular Cancer Research 2015 13 1325 1335 10.1158/1541-7786.MCR-15-0203 26037647

[41] McShan AC Devlin CA Overall SA Park J Toor JS Molecular determinants of chaperone interactions on MHC-I for folding and antigen repertoire selection Proceedings of the National Academy of Sciences 2019 116 25602 25613 10.1073/pnas.191556211 PMC6926029 31796585

[42] Qin J Xie P Ventocilla C Zhou G Vultur A Identification of a novel family of BRAFV600E inhibitors Journal of Medicinal Chemistry 2012 55 5220 5230 10.1021/jm3004416 22537109 PMC3383862

[43] Murray JB Davidson J Chen I Davis B Dokurno P Establishing drug discovery and identification of hit series for the anti-apoptotic proteins, Bcl-2 and Mcl-1 ACS Omega 2019 4 8892 8906 10.1021/acsomega.9b00611 31459977 PMC6648477

[44] Blevitt JM Hack MD Herman KL Jackson PF Krawczuk PJ Structural basis of small-molecule aggregate induced inhibition of a protein– protein interaction Journal ofMedicinal Chemistry 2017 60 3511 3517 10.1021/acs.jmedchem.6b01836 28300404

[45] Cade C Swartz P MacKenzie SH Clark AC Modifying caspase-3 activity by altering allosteric networks Biochemistry 2014 53 7582 7595 10.1021/bi500874k 25343534 PMC4263430

[46] Langenberg T Gallardo R van der Kant R Louros N Michiels E Thermodynamic and evolutionary coupling between the native and amyloid state of globular proteins Cell Reports 2020 31 107512 10.1016/j.celrep.2020.03.076 32294448 PMC7175379

[47] Borkakoti N Winkler FK Williams DH D’Arcy A Broadhurst MJ Structure of the catalytic domain of human fibroblast collagenase complexed with an inhibitor Nature Structural & Molecular Biology 1994 1 106 110 10.1038/nsb0294-106 7656013

[48] Zong S Wu M Gu J Liu T Guo R Structure of the intact 14-subunit human cytochrome c oxidase Cell Research 2018 28 1026 1034 10.1038/s41422-018-0071-1 30030519 PMC6170408

[49] Dengler MA Robin AY Gibson L Li MX Sandow JJ BAX Activation: Mutations near its proposed non-canonical BH3 binding site reveal allosteric changes controlling mitochondrial association Cell Reports 2019 27 359 373e6 10.1016/j.celrep.2019.03.040 30970242

[50] Hoegenauer K Soldermann N Stauffer F Furet P Graveleau N Discovery and pharmacological characterization of novel quinazoline-based PI3K delta-selective inhibitors ACS Medicinal Chemistry Letters 2016 7 762 767 10.1021/acsmedchemlett.6b00119 27563400 PMC4983741

[51] Lin K Lin J Wu WI Ballard J Lee BB An ATP-site on-off switch that restricts phosphatase accessibility of Akt Science Signaling 2012 5 ra37 10.1126/scisignal.2002618 22569334

[52] Wood DJ Korolchuk S Tatum NJ Wang L-Z Endicott JA Differences in the conformational energy landscape of CDK1 and CDK2 suggest a mechanism for achieving selective CDK inhibition Cell Chemical Biology 2019 26 121 130 10.1016/j.chembiol.2018.10.015 30472117 PMC6344228

[53] Ghanim GE Fountain AJ Van Roon A-MM Rangan R Das R Structure of human telomerase holoenzyme with bound telomeric DNA Nature 2021 593 449 453 10.1038/s41586-021-03415-4 33883742 PMC7610991

[54] Federici L Lo Sterzo C Pezzola S Di Matteo A Scaloni F Structural basis for the binding of the anticancer compound 6-(7-nitro-2,1,3-benzoxadiazol-4-ylthio)hexanol to human glutathione s-transferases Cancer Research 2009 69 8025 8034 10.1158/0008-5472.Can-09-1314 19808963

[55] Schrödinger Release 2021-2, Protein Preparation Wizard Epik S, LLC New York, NY 2021

[56] Çöl ÖF Bozbey İ Türkmenoğlu B Uysal M 3(2H)-pyridazinone derivatives: Synthesis, in-silico studies, structure-activity relationship and invitro evaluation for acetylcholinesterase enzyme inhibition Journal of Molecular Structure 2022 1261 132970 10.1016/j.molstruc.2022.132970

[57] Schrödinger Release 2021-2 Prime S LLC New York, NY 2021

[58] Kuzu B Hepokur C Turkmenoglu B Burmaoglu S Algul O Design, synthesis and in vitro antiproliferation activity of some 2-aryl and –heteroaryl benzoxazole derivatives Future Medicinal Chemistry 2022 14 1027 1048 10.4155/fmc-2022-0076 35703122

