# Supplementary figures and images for: New pyrimidine-N-β-D-glucosides: synthesis, biological evaluation, and molecular docking investigations
Source: Turk J Chem. 2023 Feb 28;47(2):476–94. doi: 10.55730/1300-0527.3553 (PMC10387993; doi:10.55730/1300-0527.3553)

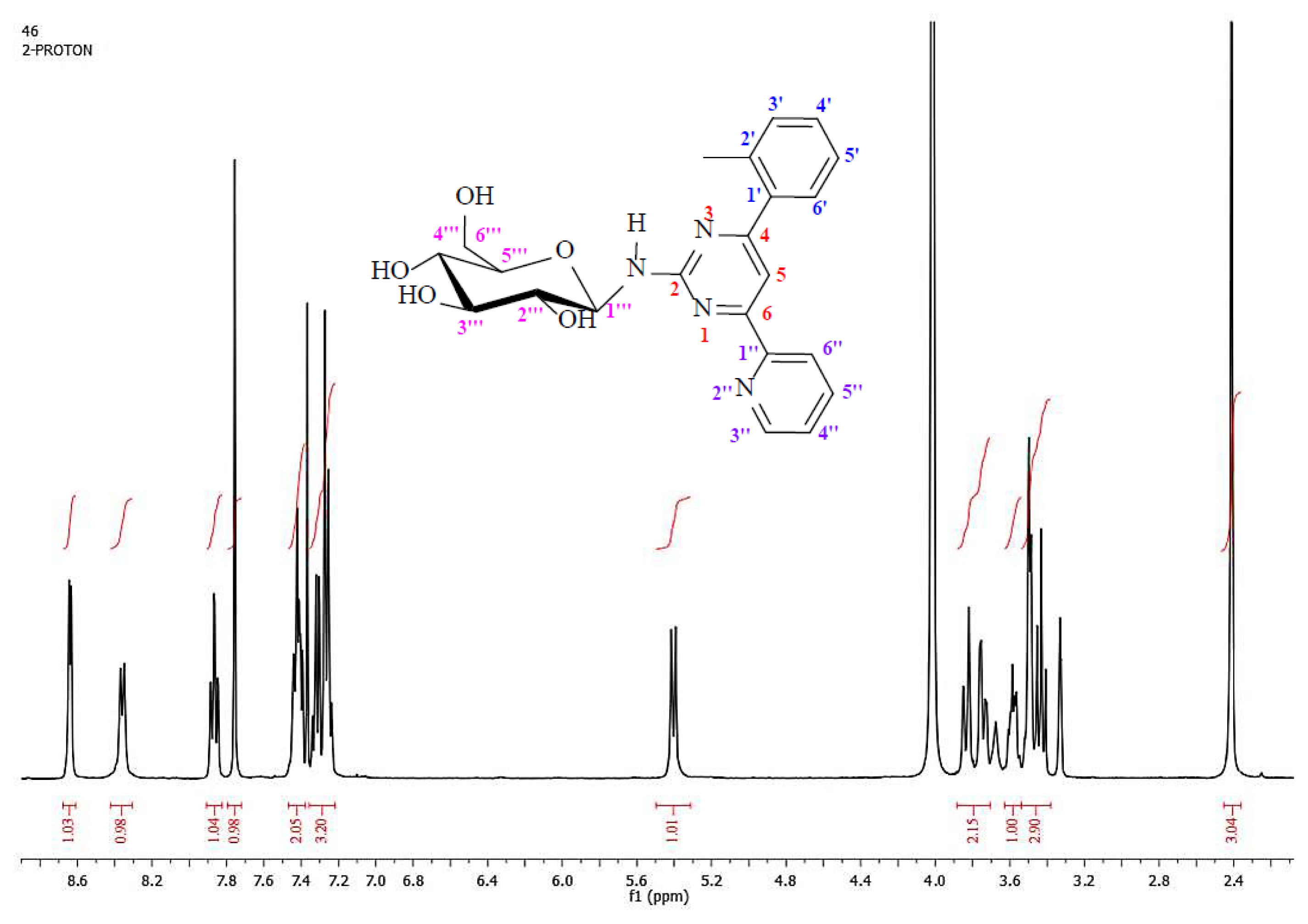

Supplement: Figure S1 — 1H-NMR spectrum of compound 1 (400 MHz, CDCl3/CD3OD (5:1)). [file turkjchem-47-2-476s1.tif]

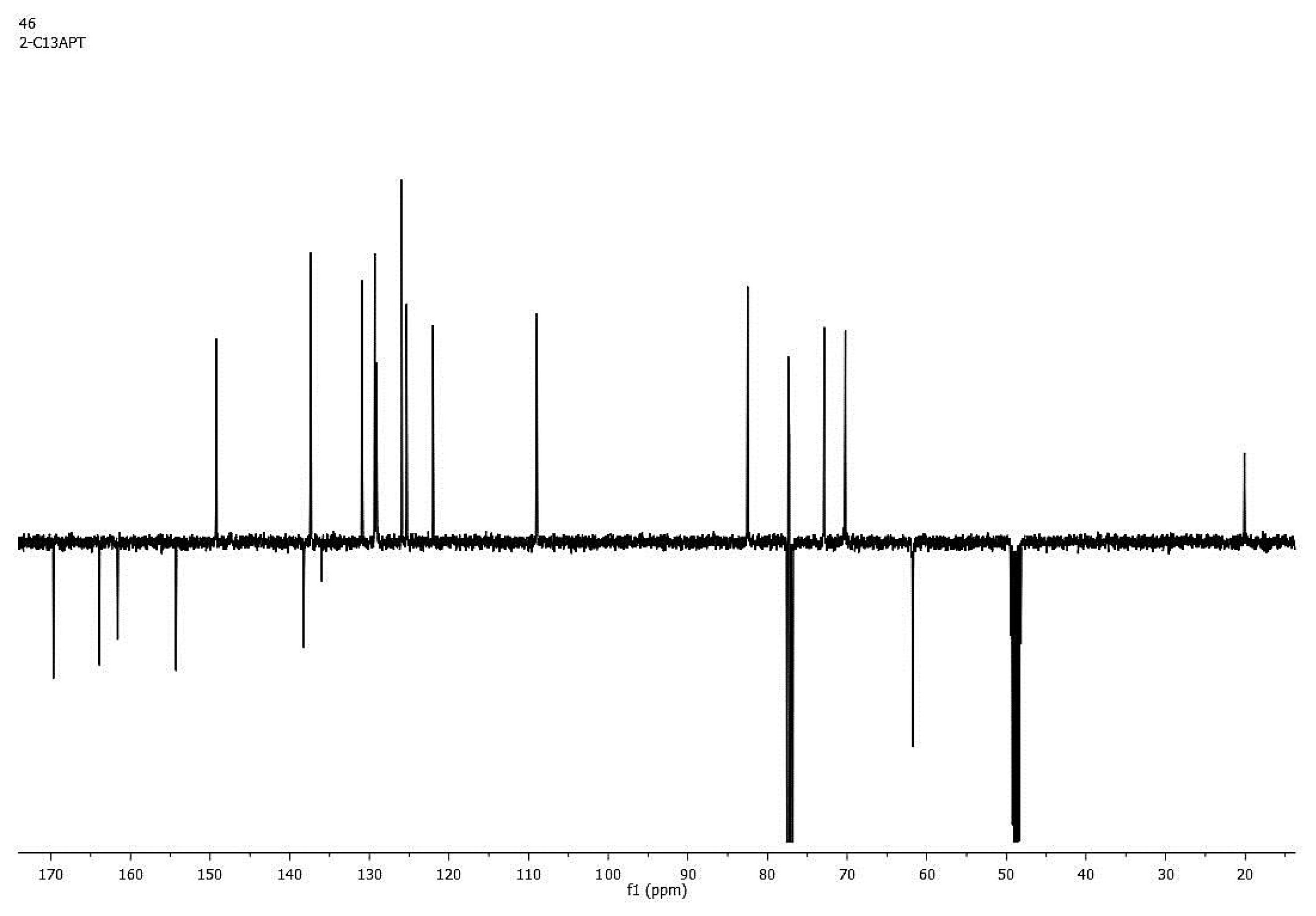

Supplement: Figure S2 — 13C-APT NMR spectrum of compound 1 (100 MHz, CDCl3/CD3OD (5:1)). [file turkjchem-47-2-476s2.tif]

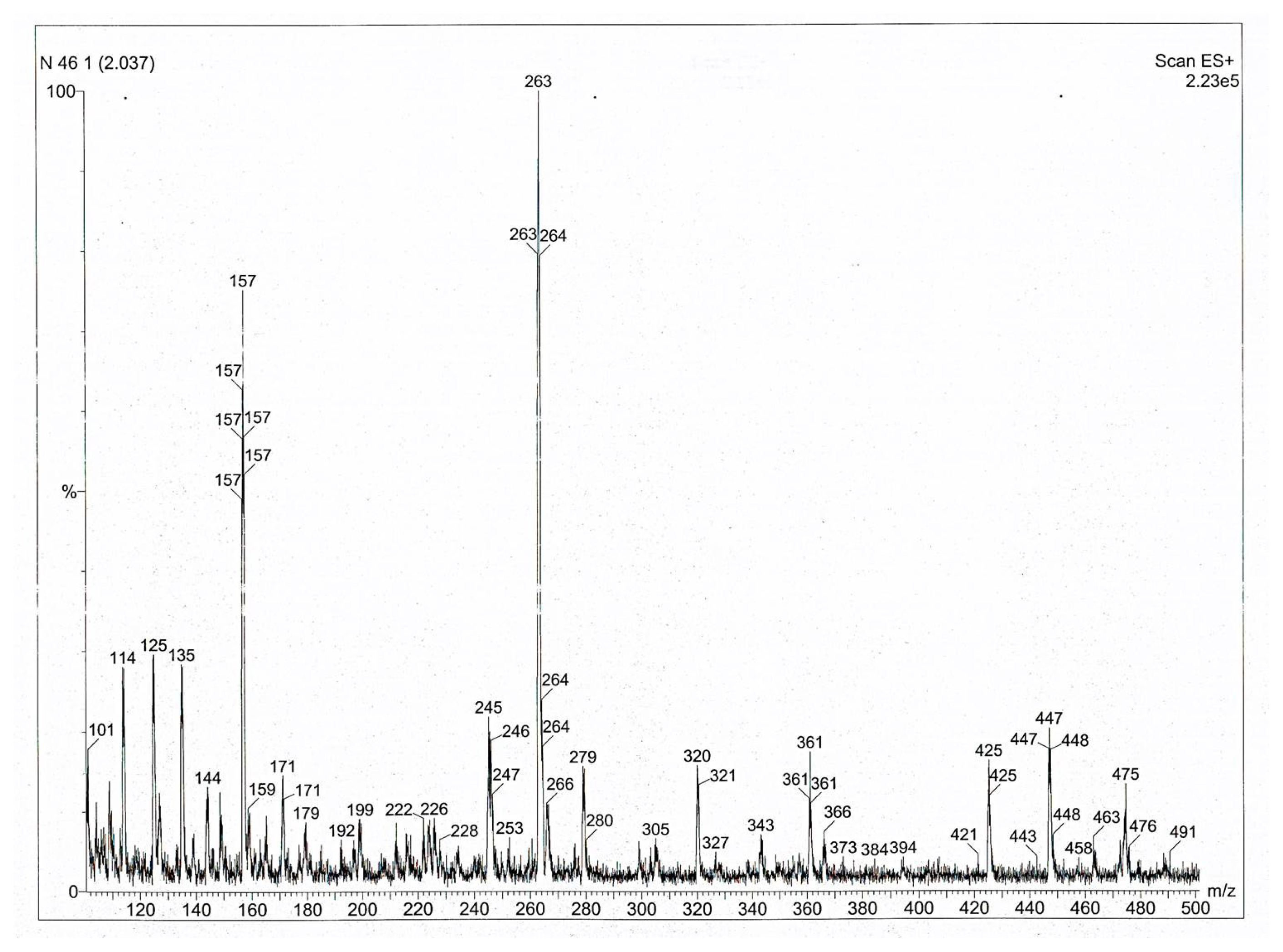

Supplement: Figure S3 — LC-MS/MS spectrum of compound 1. [file turkjchem-47-2-476s3.tif]

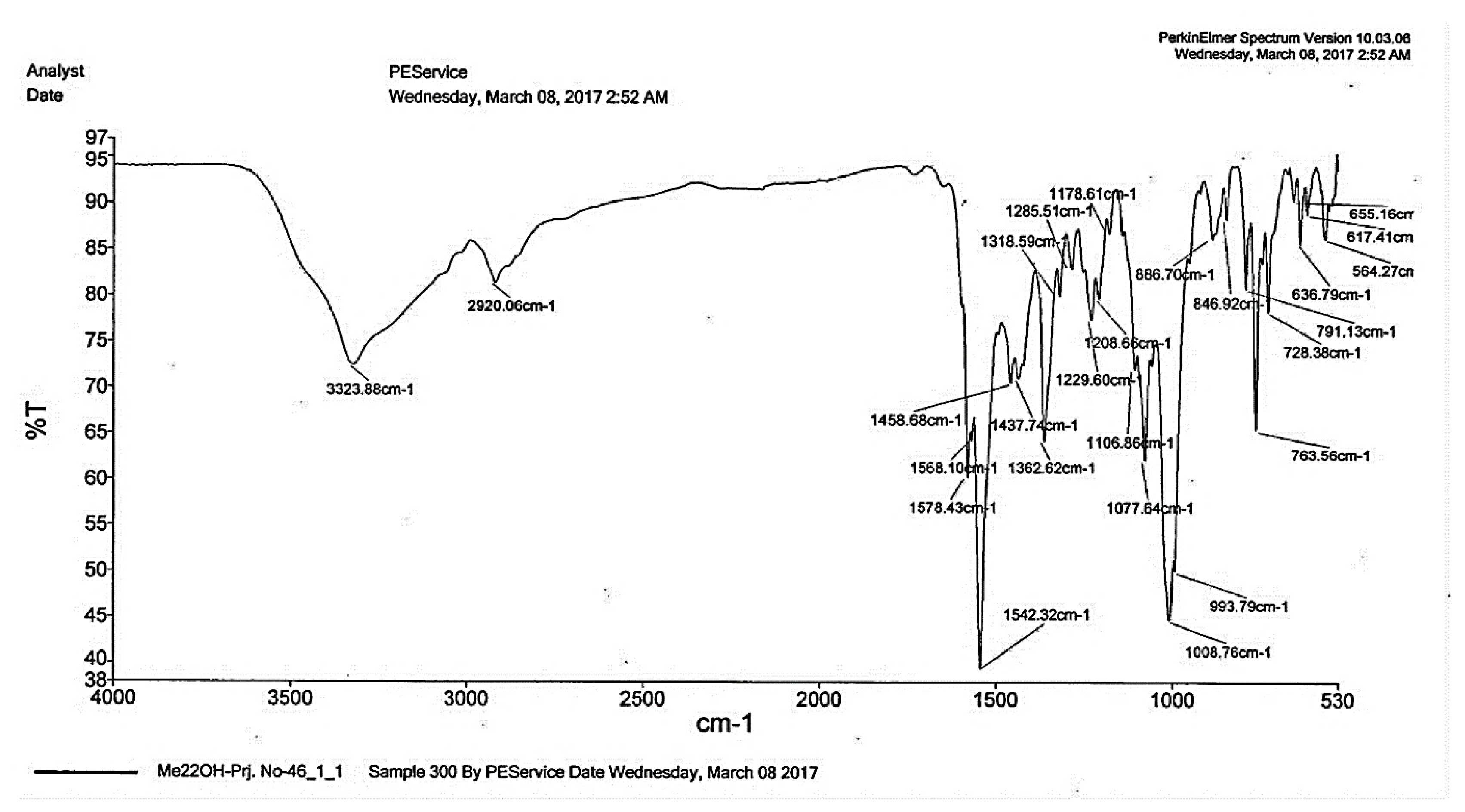

Supplement: Figure S4 — FT-IR spectrum of compound 1. [file turkjchem-47-2-476s4.tif]

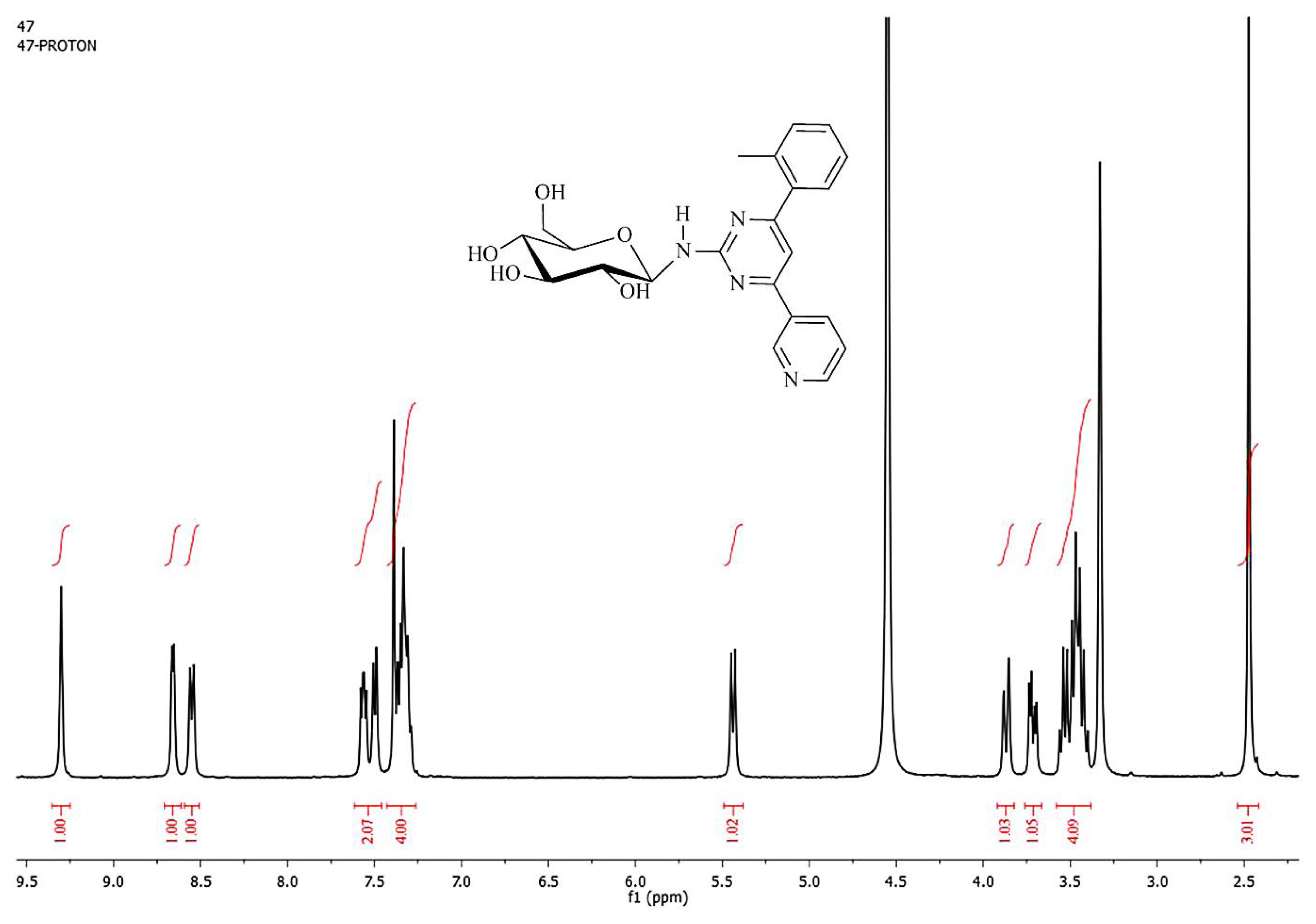

Supplement: Figure S5 — 1H-NMR spectrum of compound 2 (400 MHz, CD3OD) [file turkjchem-47-2-476s5.tif]

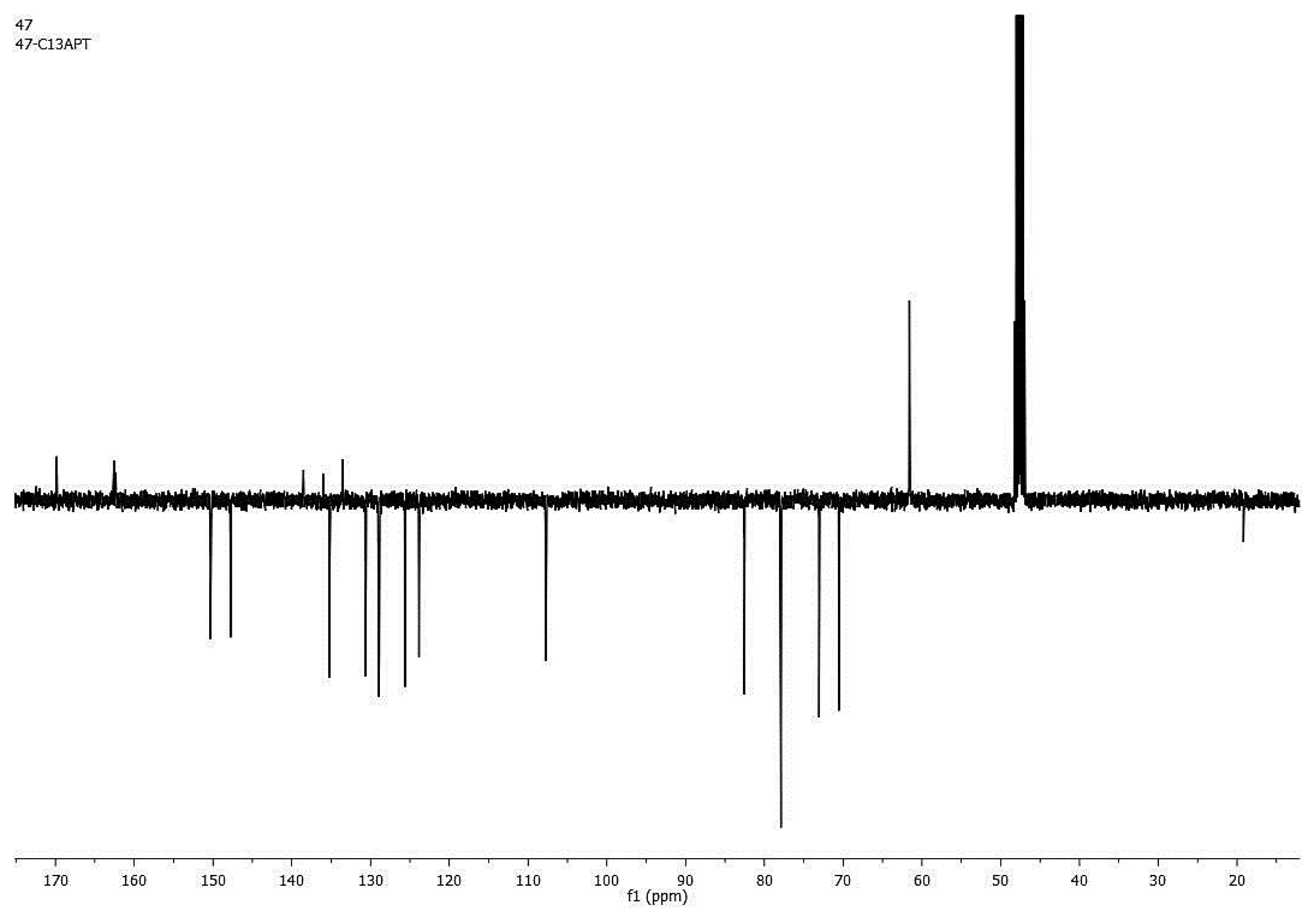

Supplement: Figure S6 — 13C-APT NMR spectrum of compound 2 (100 MHz, CD3OD) [file turkjchem-47-2-476s6.tif]

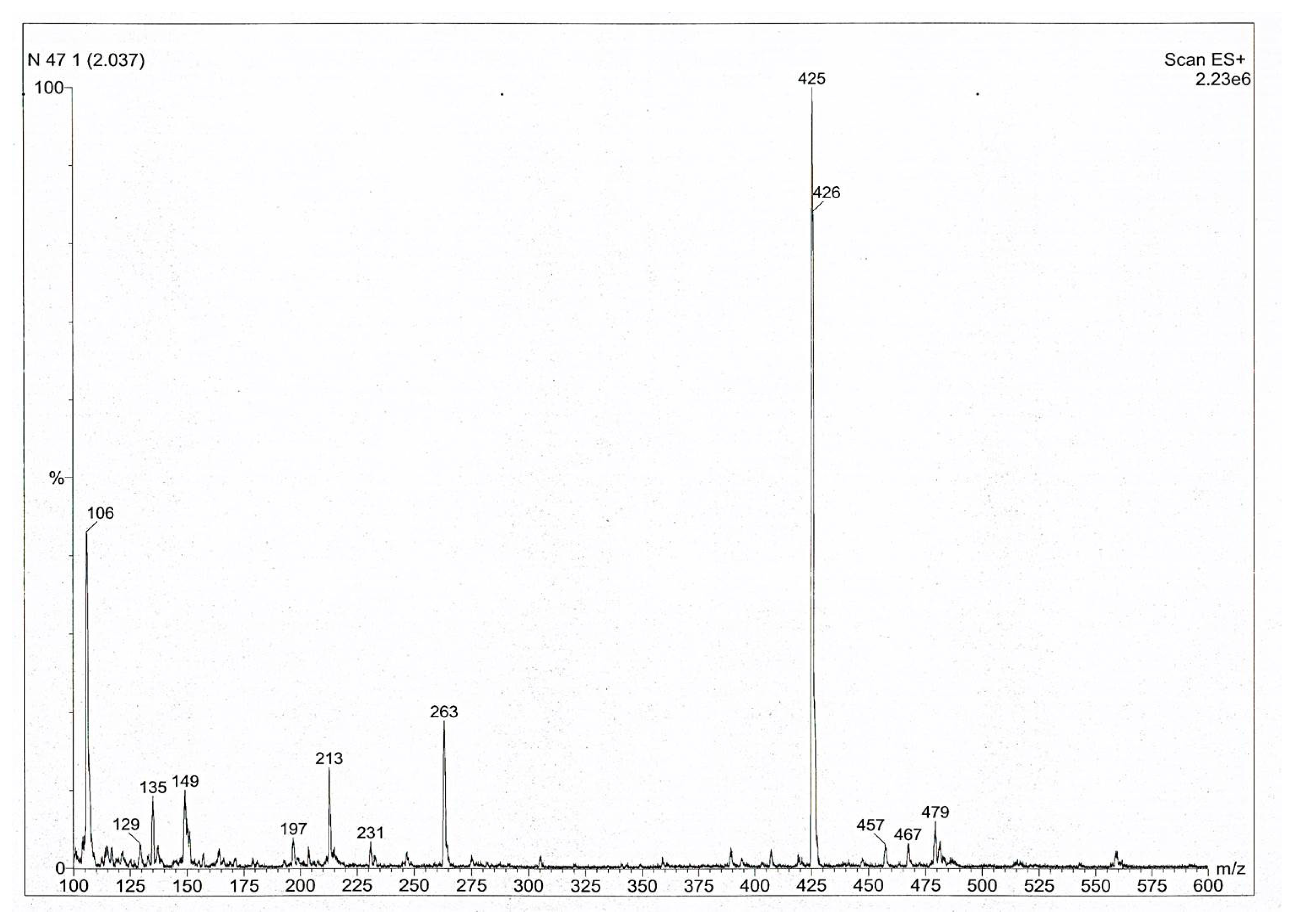

Supplement: Figure S7 — LC-MS/MS spectrum of compound 2 [file turkjchem-47-2-476s7.tif]

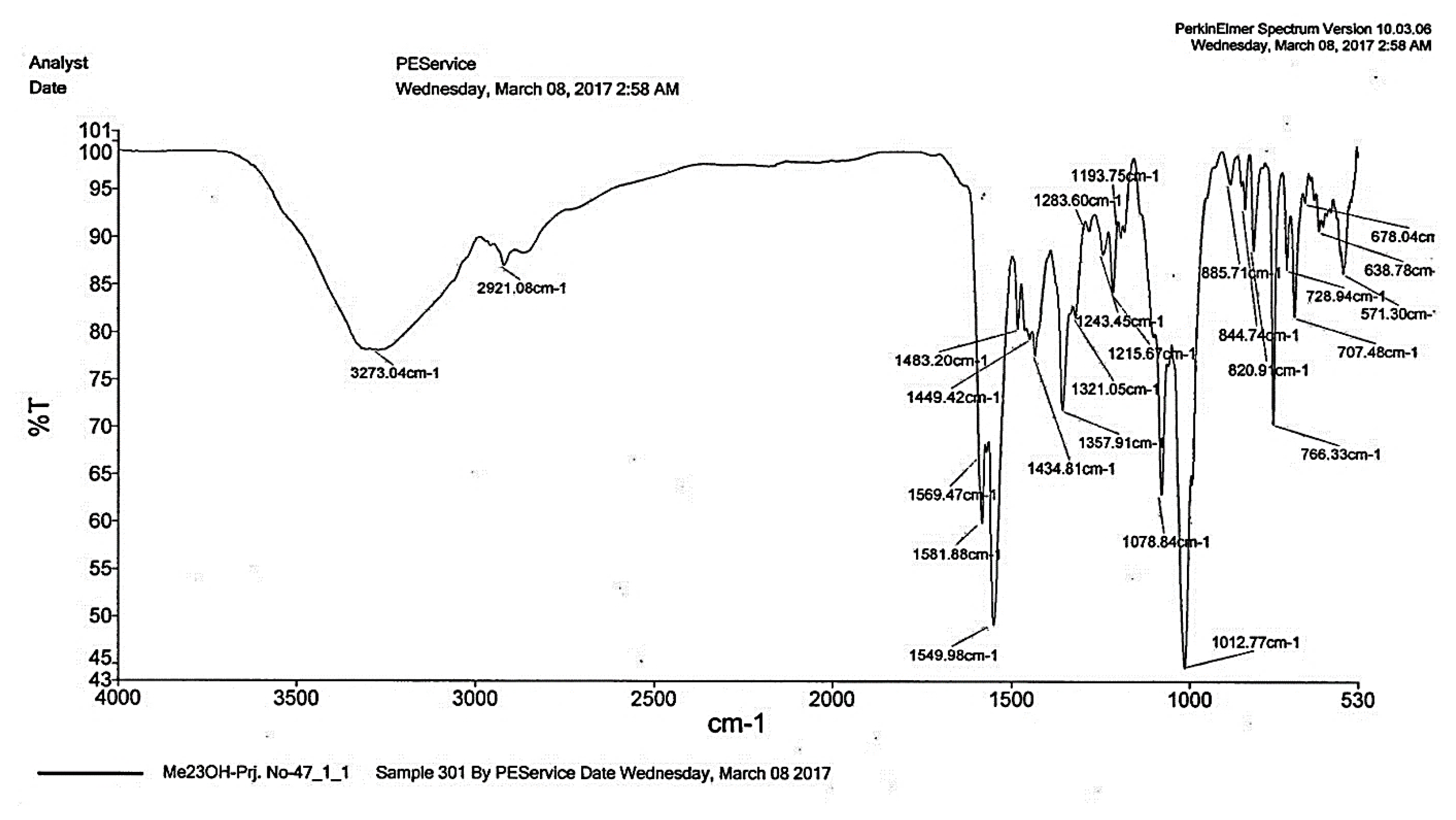

Supplement: Figure S8 — FT-IR spectrum of compound 2 [file turkjchem-47-2-476s8.tif]

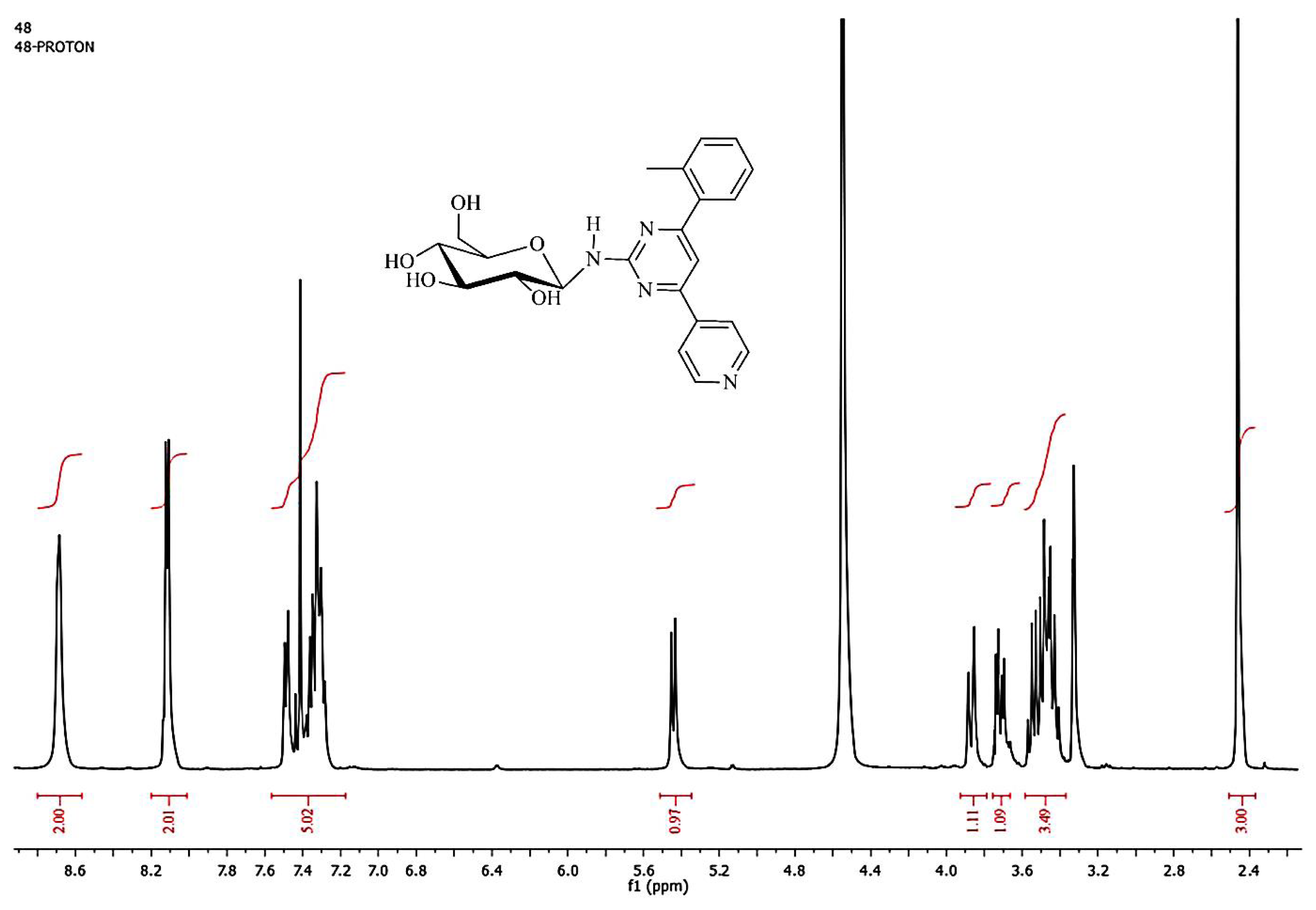

Supplement: Figure S9 — 1H-NMR spectrum of compound 3 (400 MHz, CD3OD). [file turkjchem-47-2-476s9.tif]

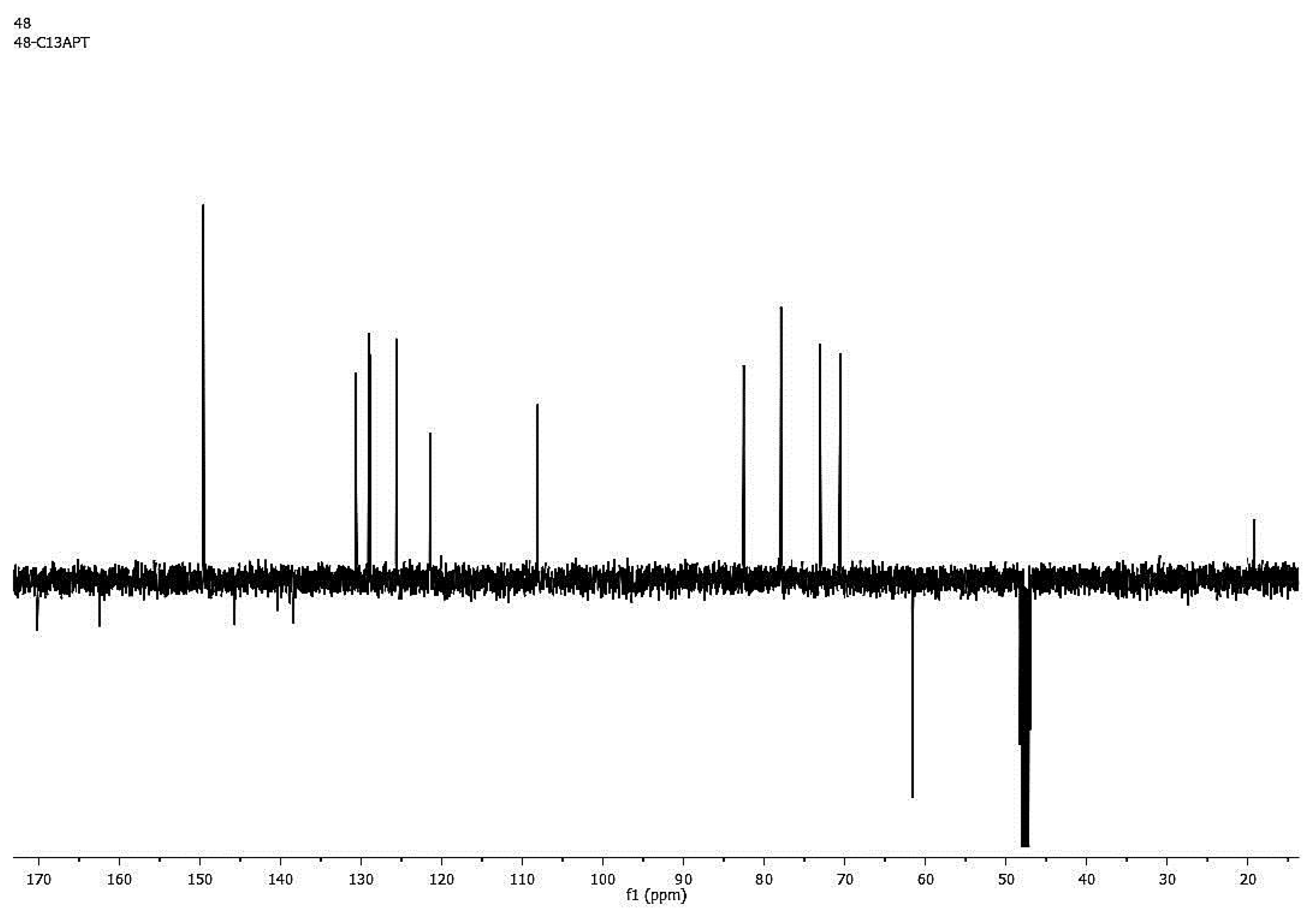

Supplement: Figure S10 — 13C-APT NMR spectrum of compound 3 (100 MHz, CD3OD). [file turkjchem-47-2-476s10.tif]

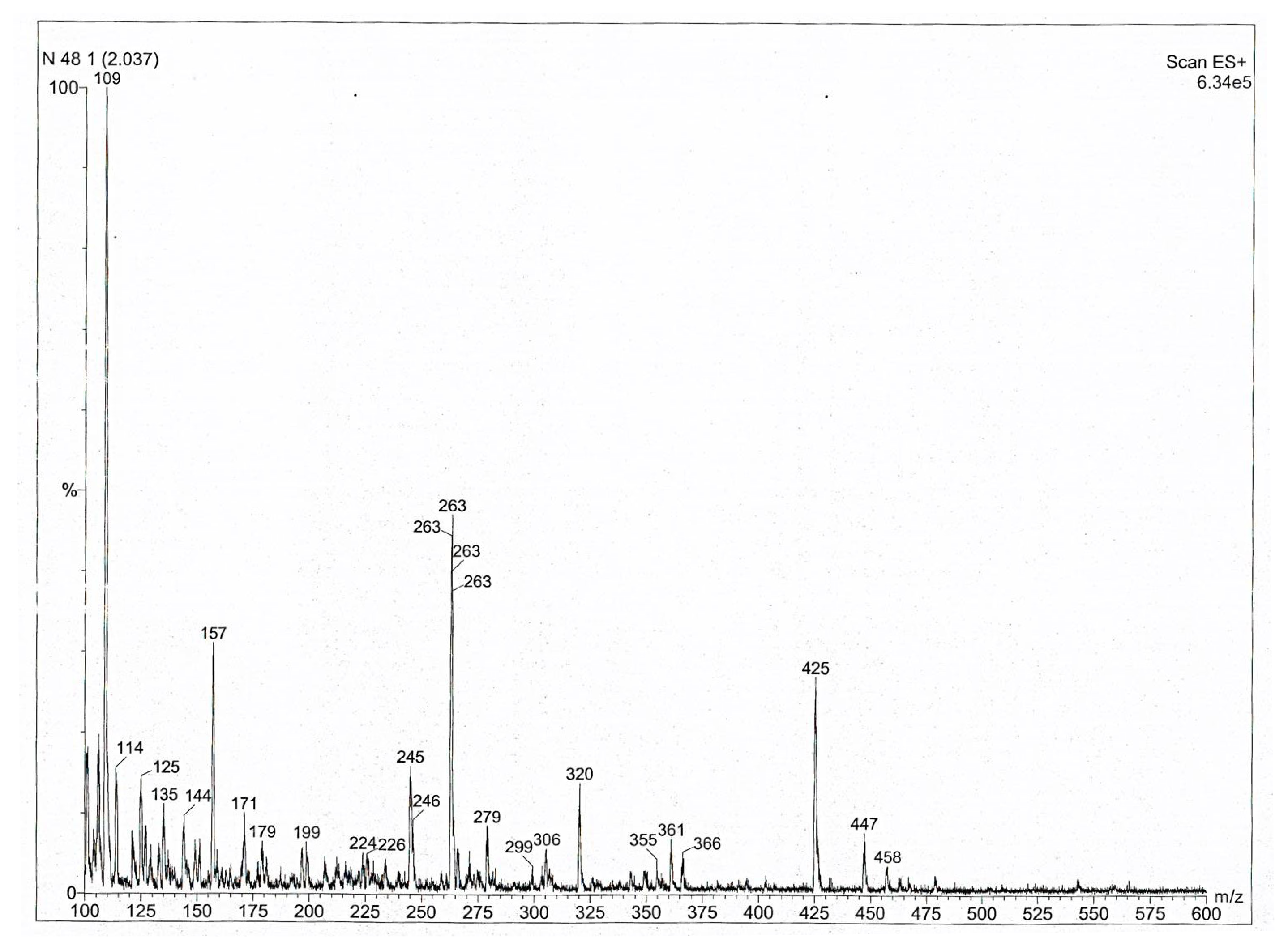

Supplement: Figure S11 — LC-MS/MS spectrum of compound 3. [file turkjchem-47-2-476s11.tif]

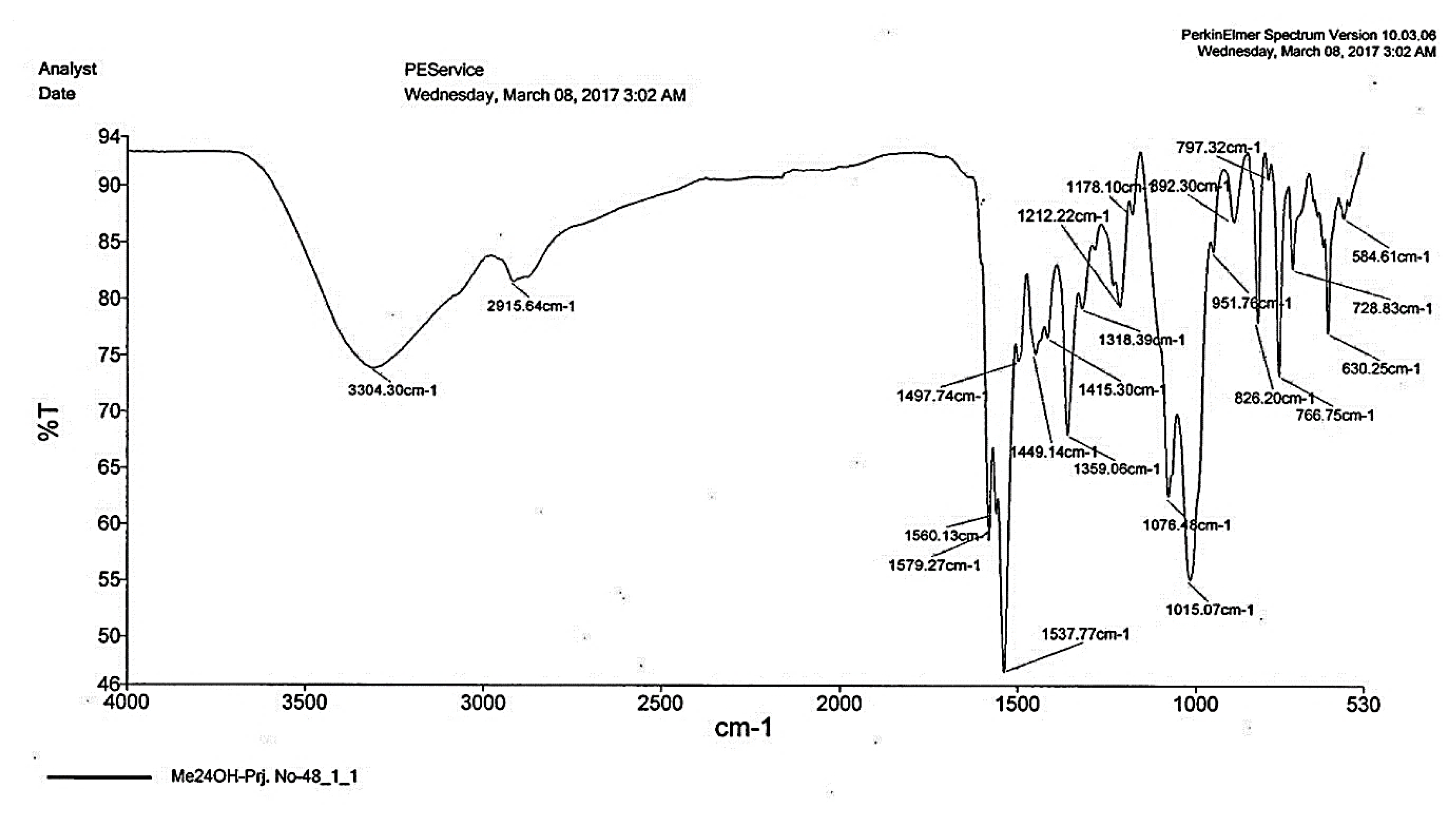

Supplement: Figure S12 — FT-IR spectrum of compound 3. [file turkjchem-47-2-476s12.tif]

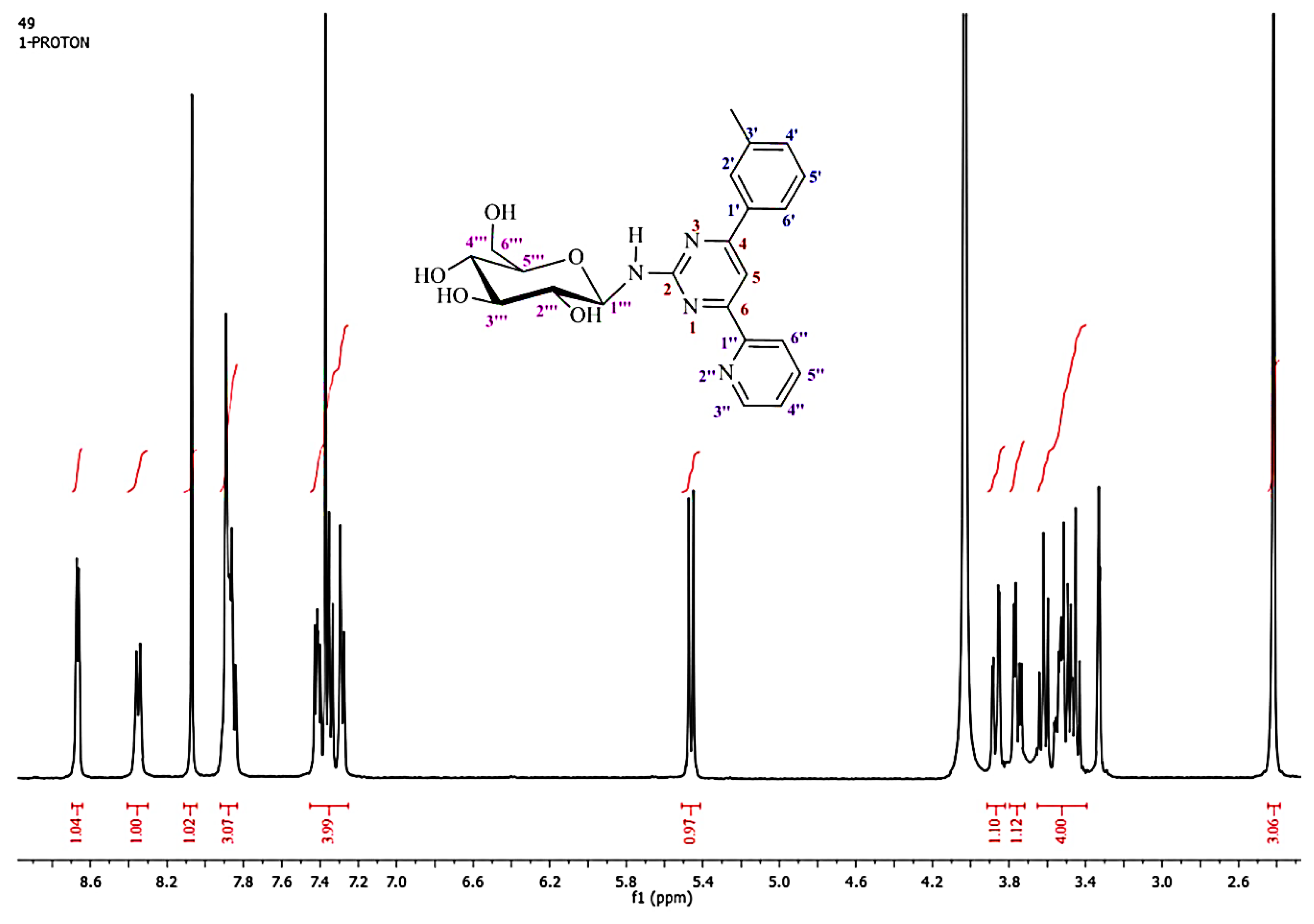

Supplement: Figure S13 — 1H-NMR spectrum of compound 4 (400 MHz, CDCl3/CD3OD (5:1)). [file turkjchem-47-2-476s13.tif]

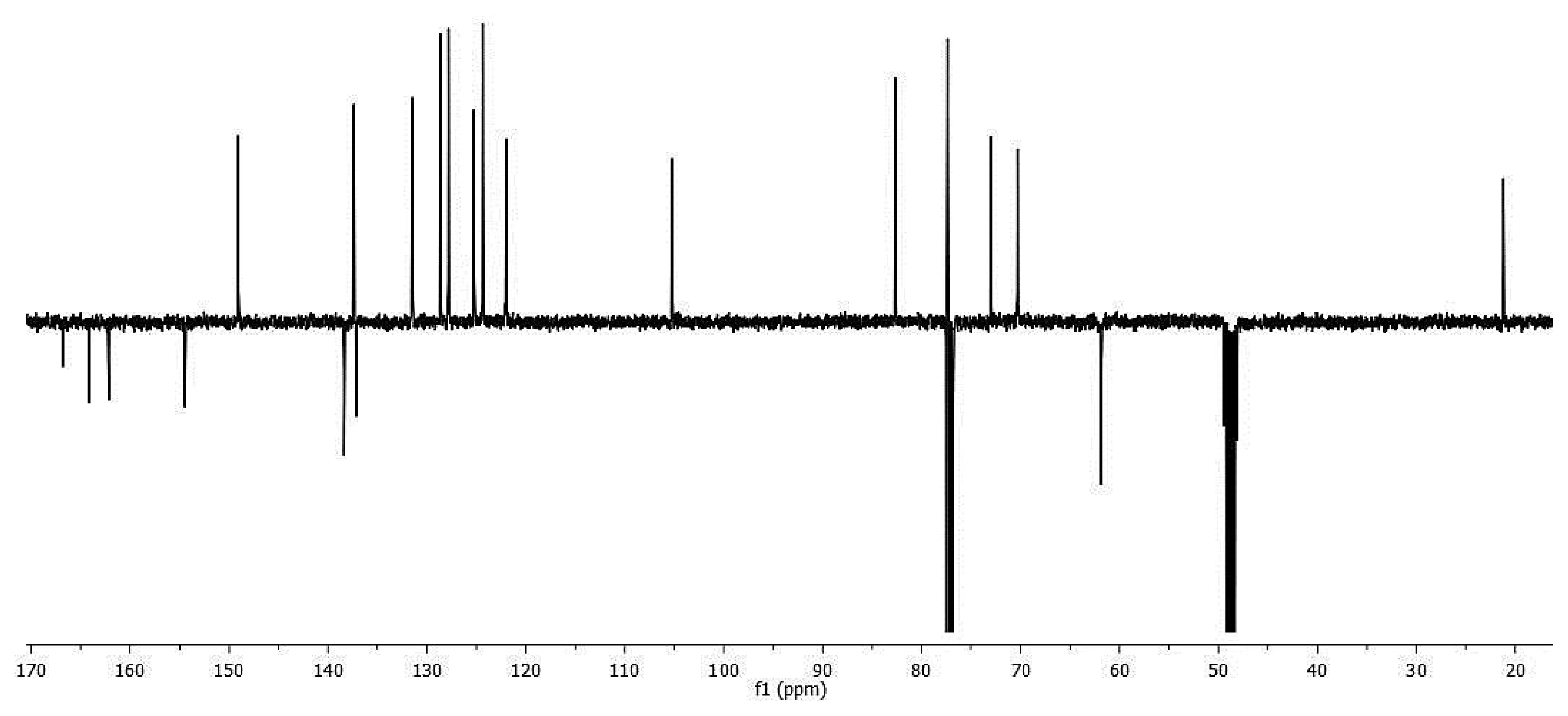

Supplement: Figure S14 — 13C-APT NMR spectrum of compound 4 (100 MHz, CDCl3/CD3OD (5:1)). [file turkjchem-47-2-476s14.tif]

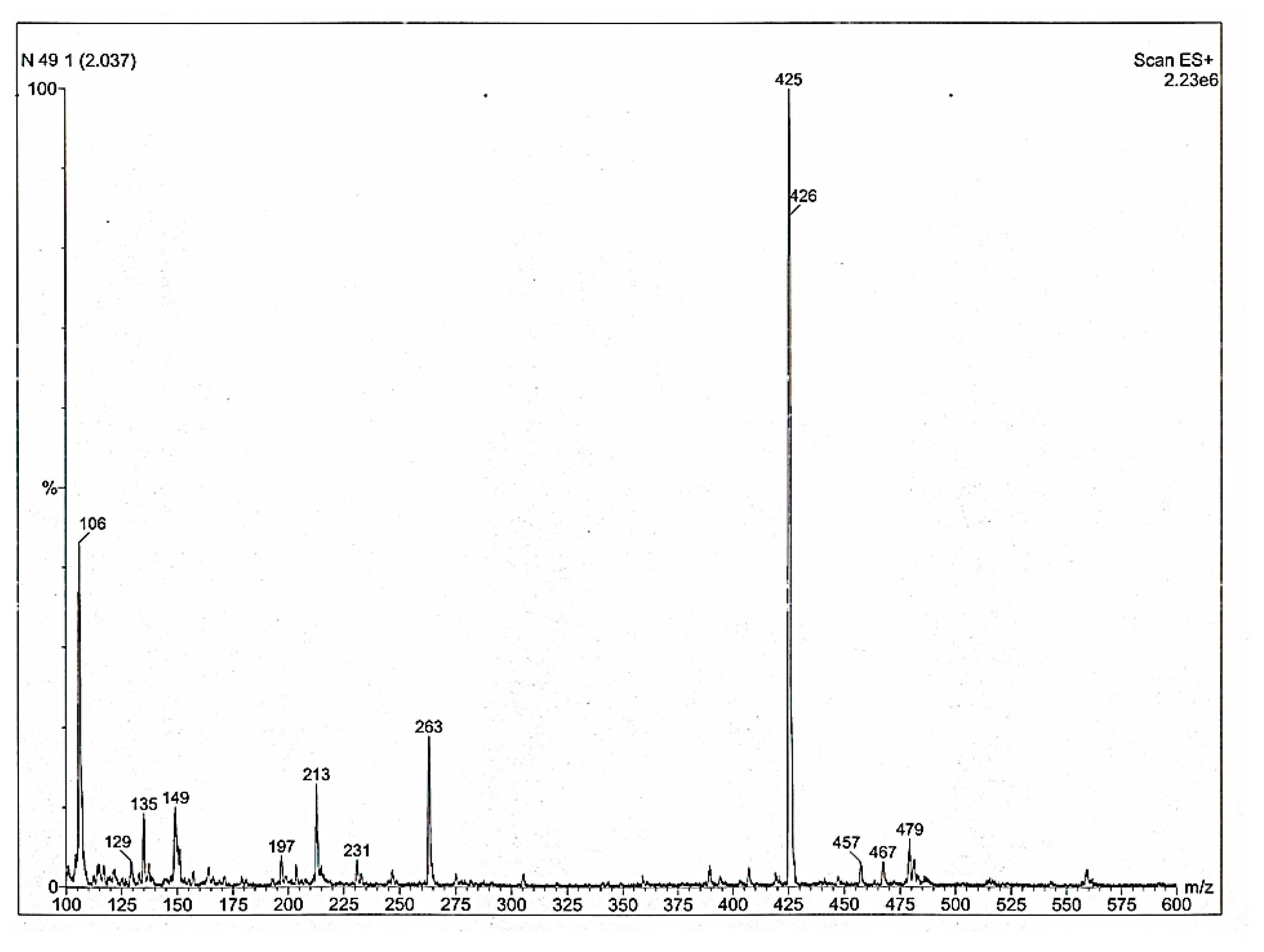

Supplement: Figure S15 — LC-MS/MS spectrum of compound 4. [file turkjchem-47-2-476s15.tif]

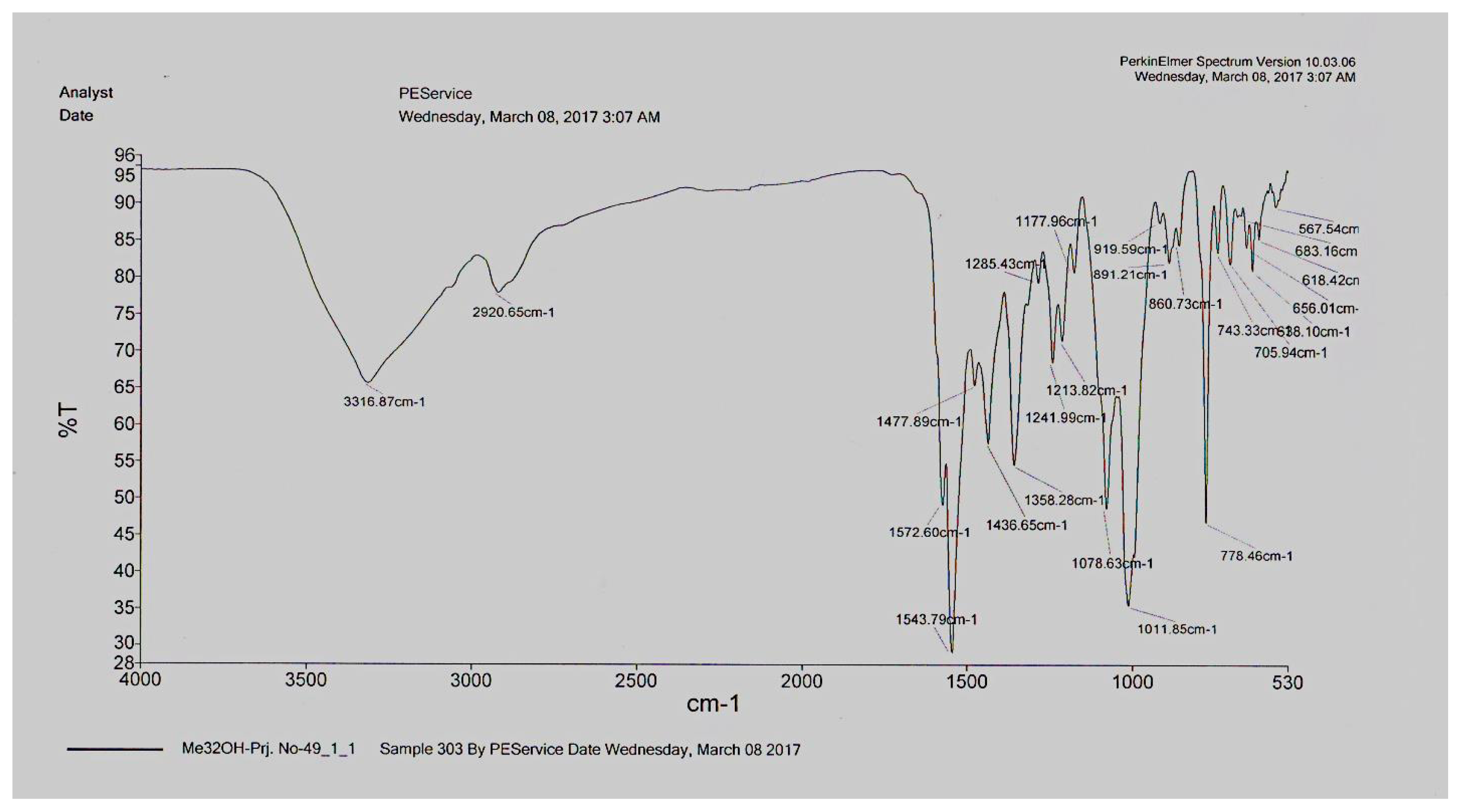

Supplement: Figure S16 — FT-IR spectrum of compound 4. [file turkjchem-47-2-476s16.tif]

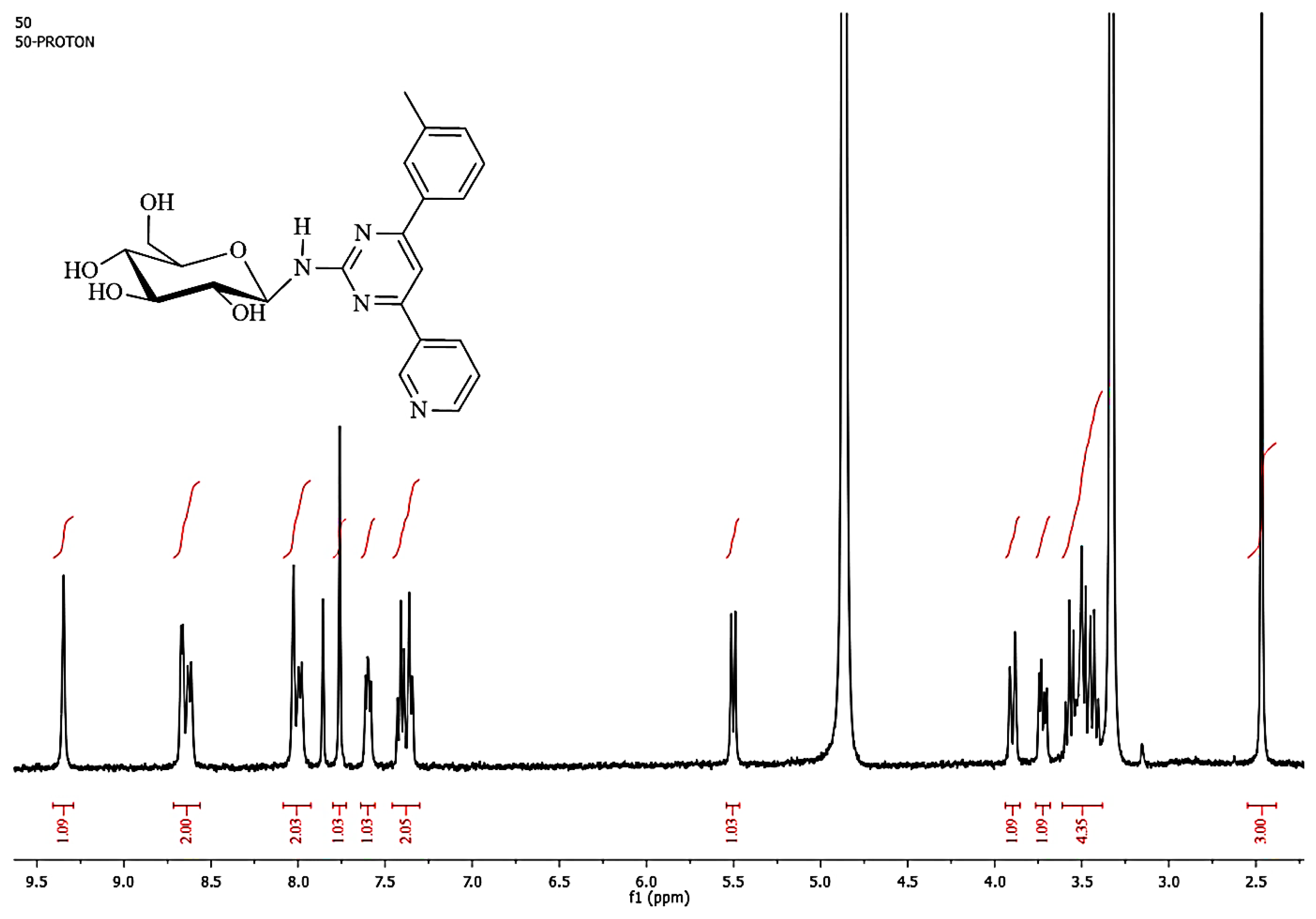

Supplement: Figure S17 — 1H-NMR spectrum of compound 5 (400 MHz, CDCl3/CD3OD (5:1)). [file turkjchem-47-2-476s17.tif]

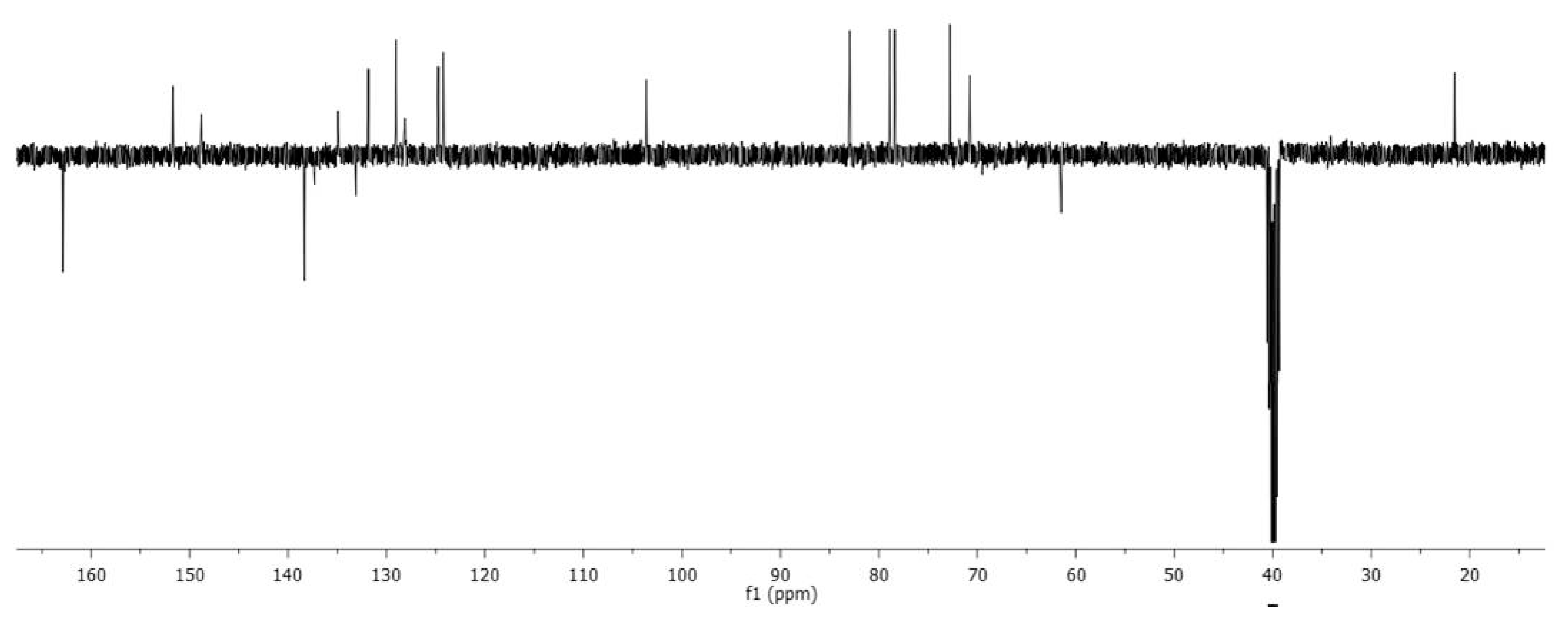

Supplement: Figure S18 — 13C-APT NMR spectrum of compound 5 (100 MHz, DMSO-d6). [file turkjchem-47-2-476s18.tif]

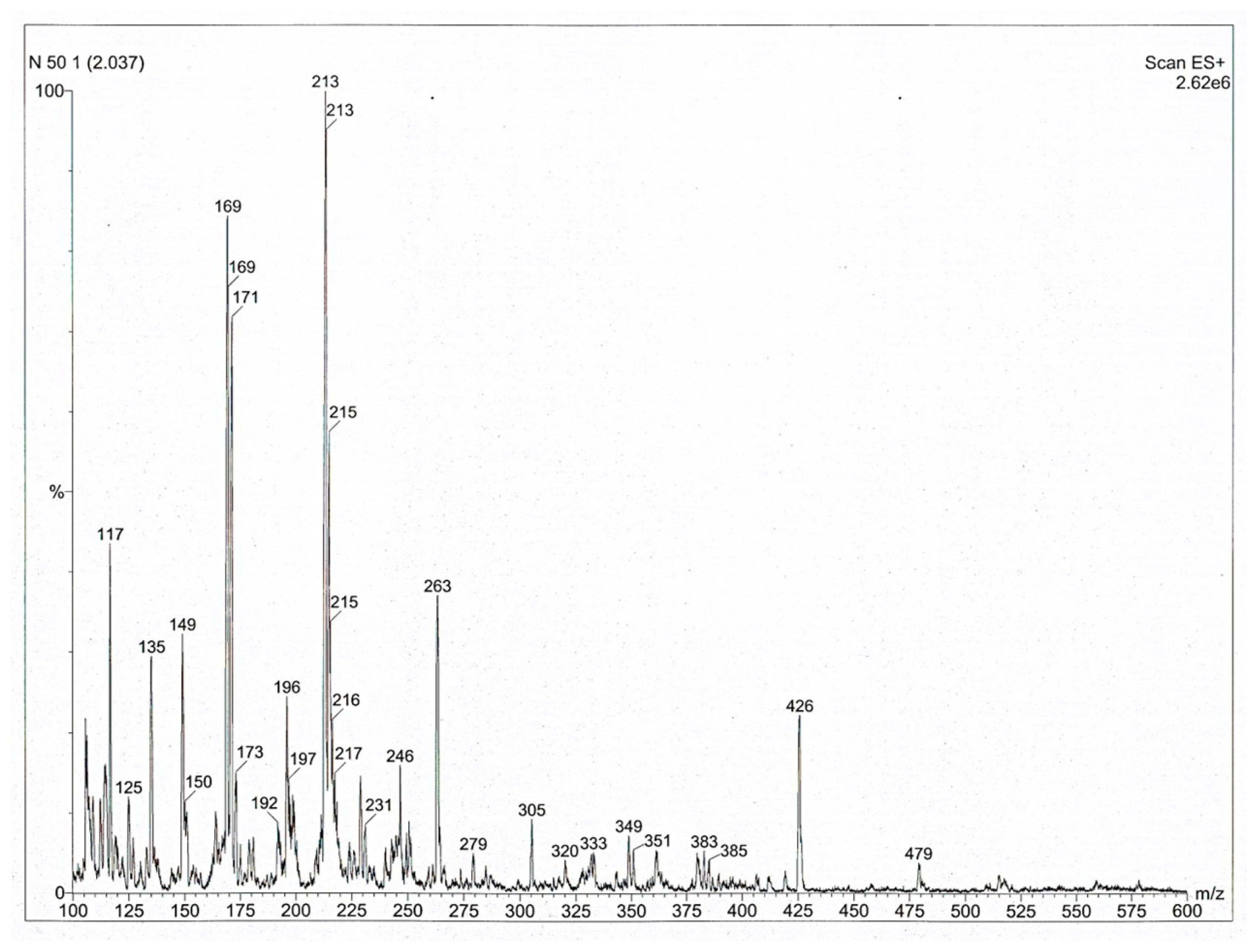

Supplement: Figure S19 — LC-MS/MS spectrum of compound 5. [file turkjchem-47-2-476s19.tif]

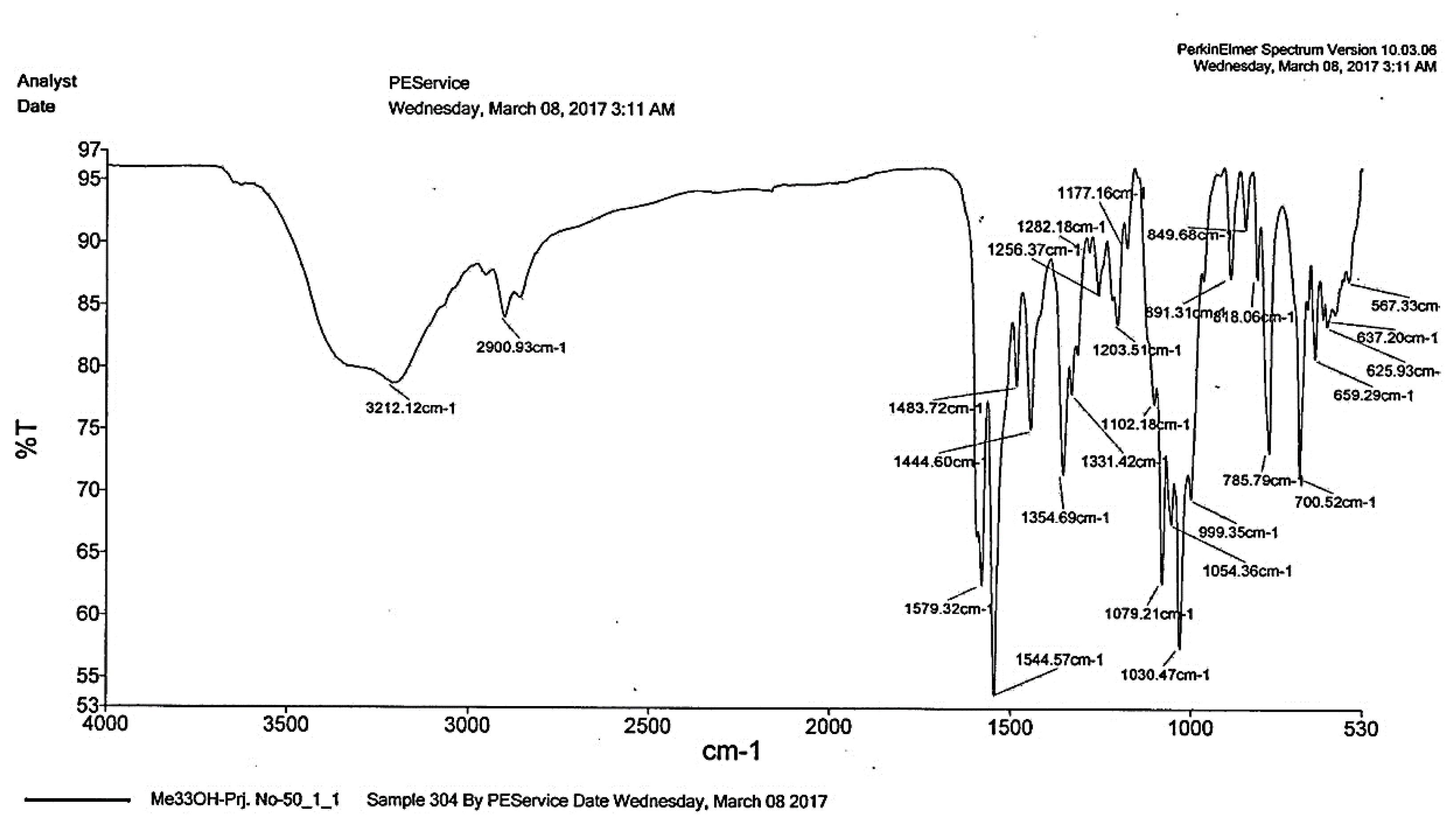

Supplement: Figure S20 — FT-IR spectrum of compound 5. [file turkjchem-47-2-476s20.tif]

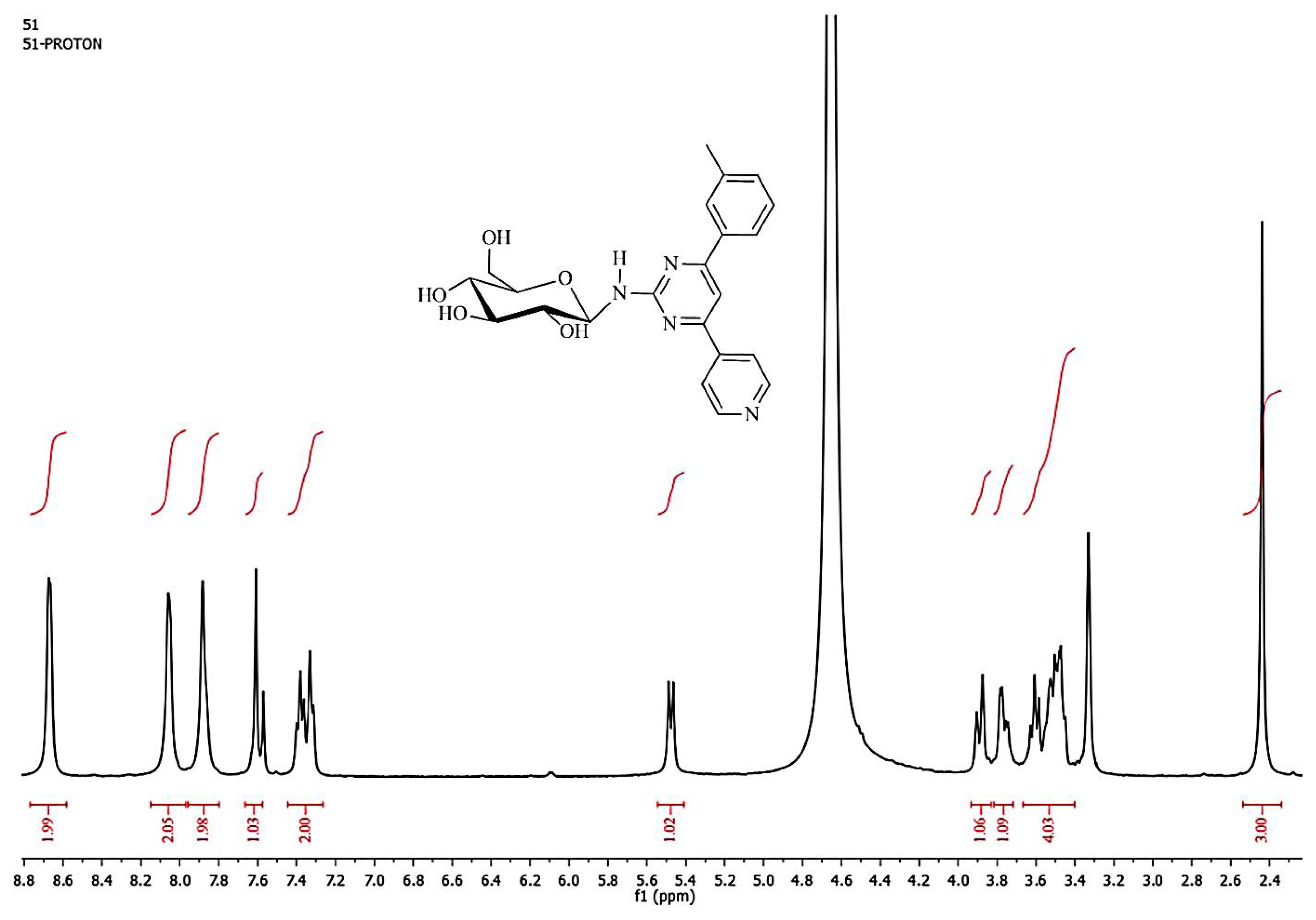

Supplement: Figure S21 — 1H-NMR spectrum of compound 6 (400 MHz, CDCl3/CD3OD (5:1)). [file turkjchem-47-2-476s21.tif]

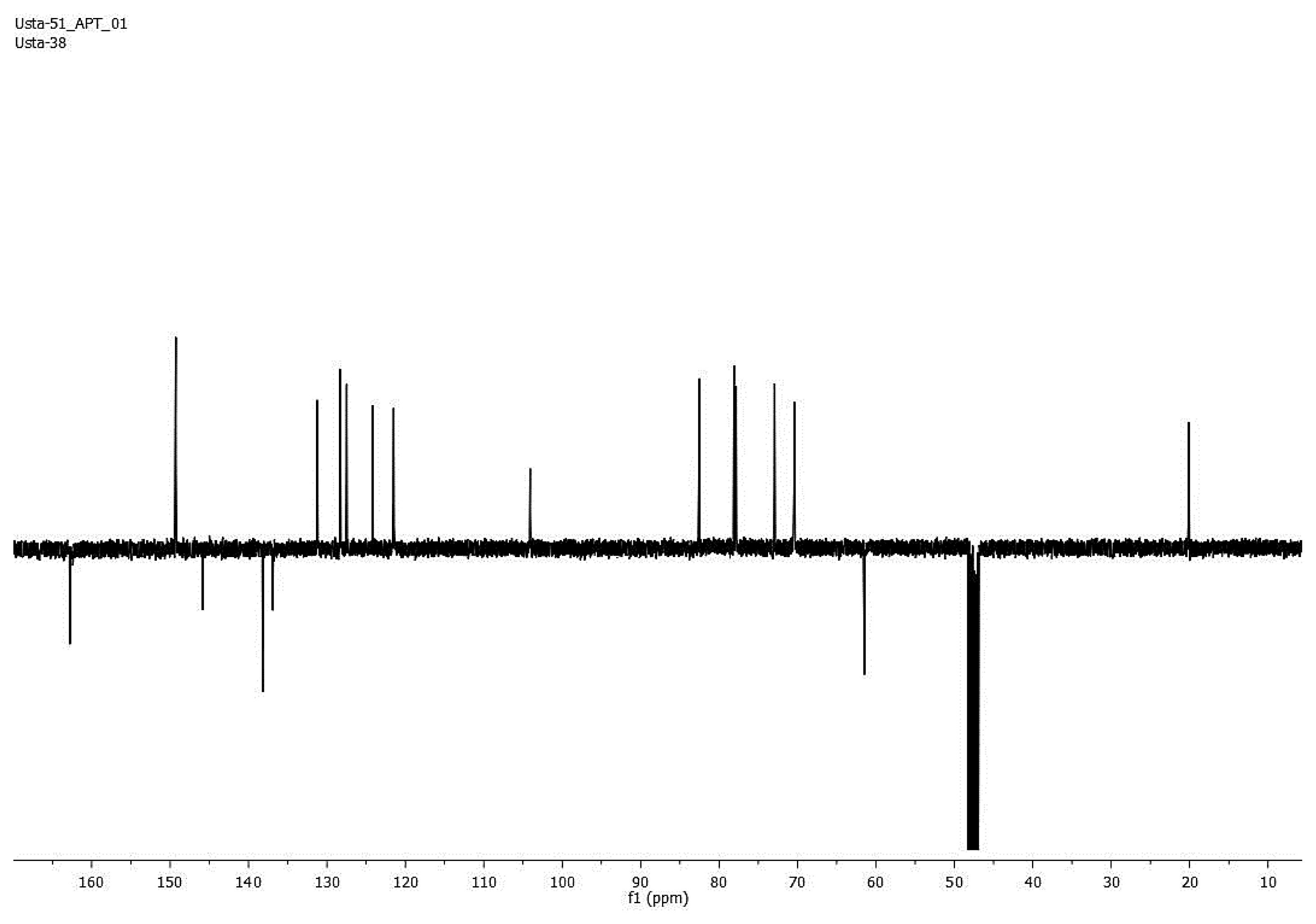

Supplement: Figure S22 — 13C-APT NMR spectrum of compound 6 (100 MHz, CD3OD). [file turkjchem-47-2-476s22.tif]

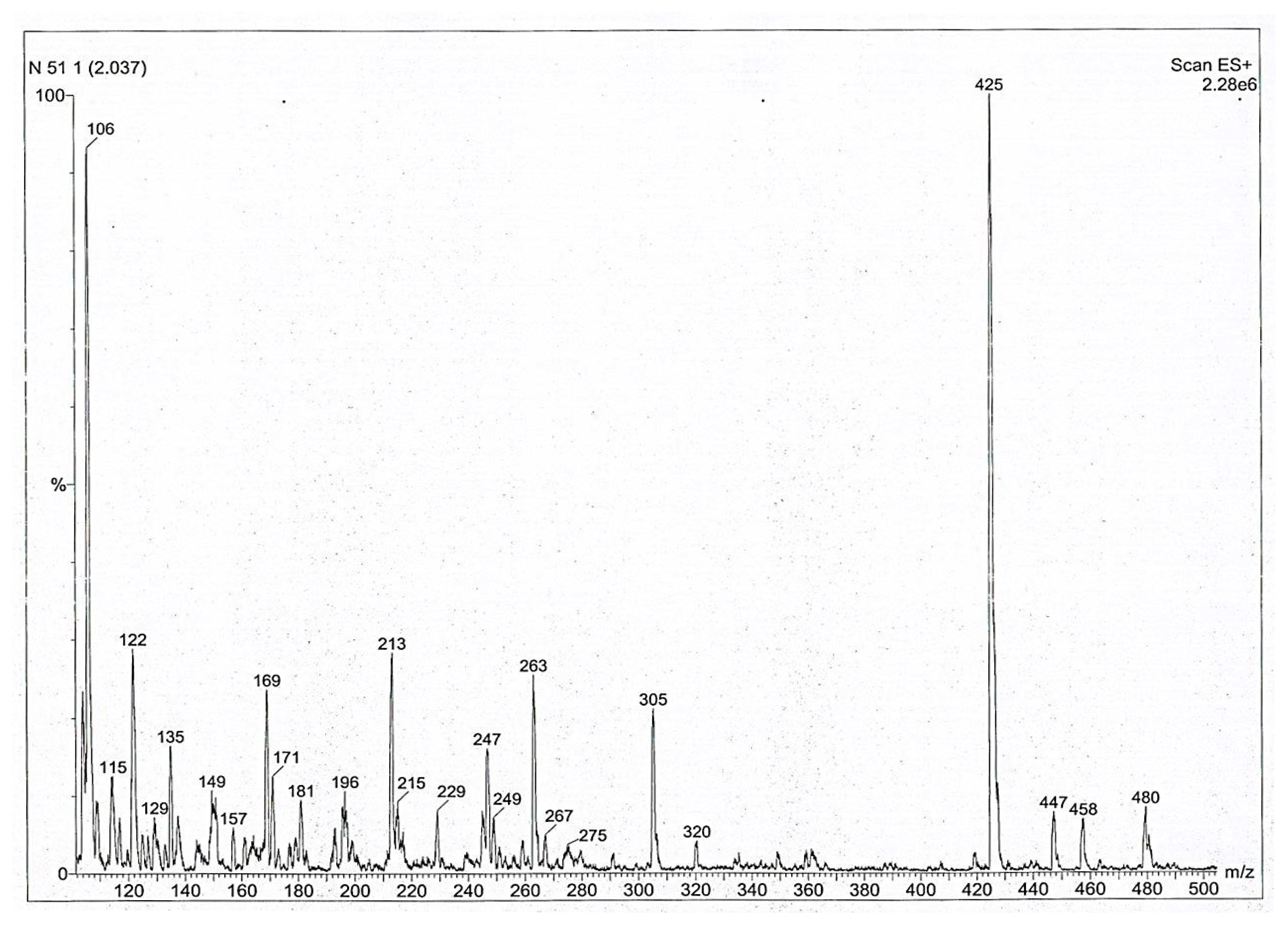

Supplement: Figure S23 — LC-MS/MS spectrum of compound 6. [file turkjchem-47-2-476s23.tif]

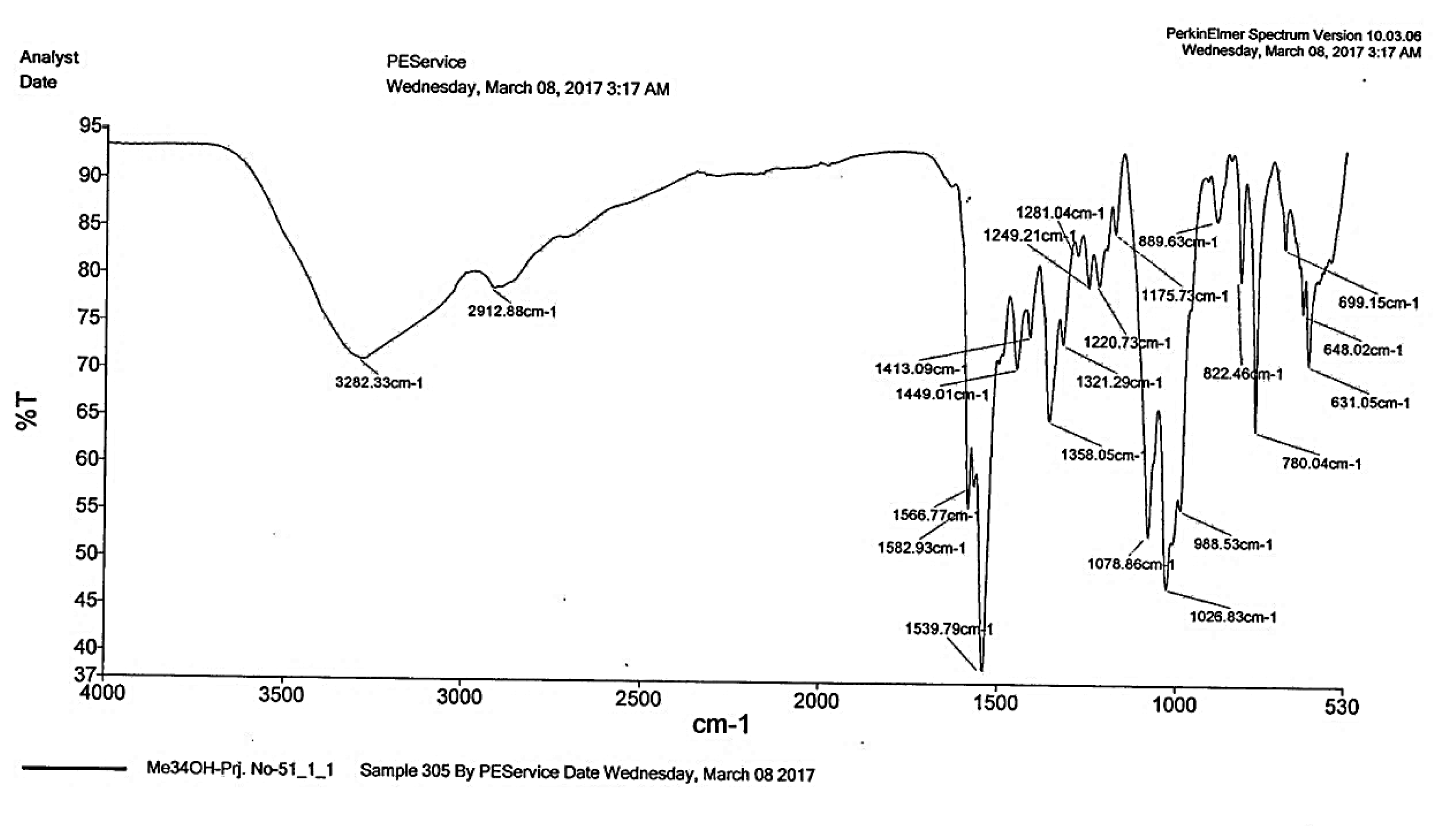

Supplement: Figure S24 — FT-IR spectrum of compound 6. [file turkjchem-47-2-476s24.tif]

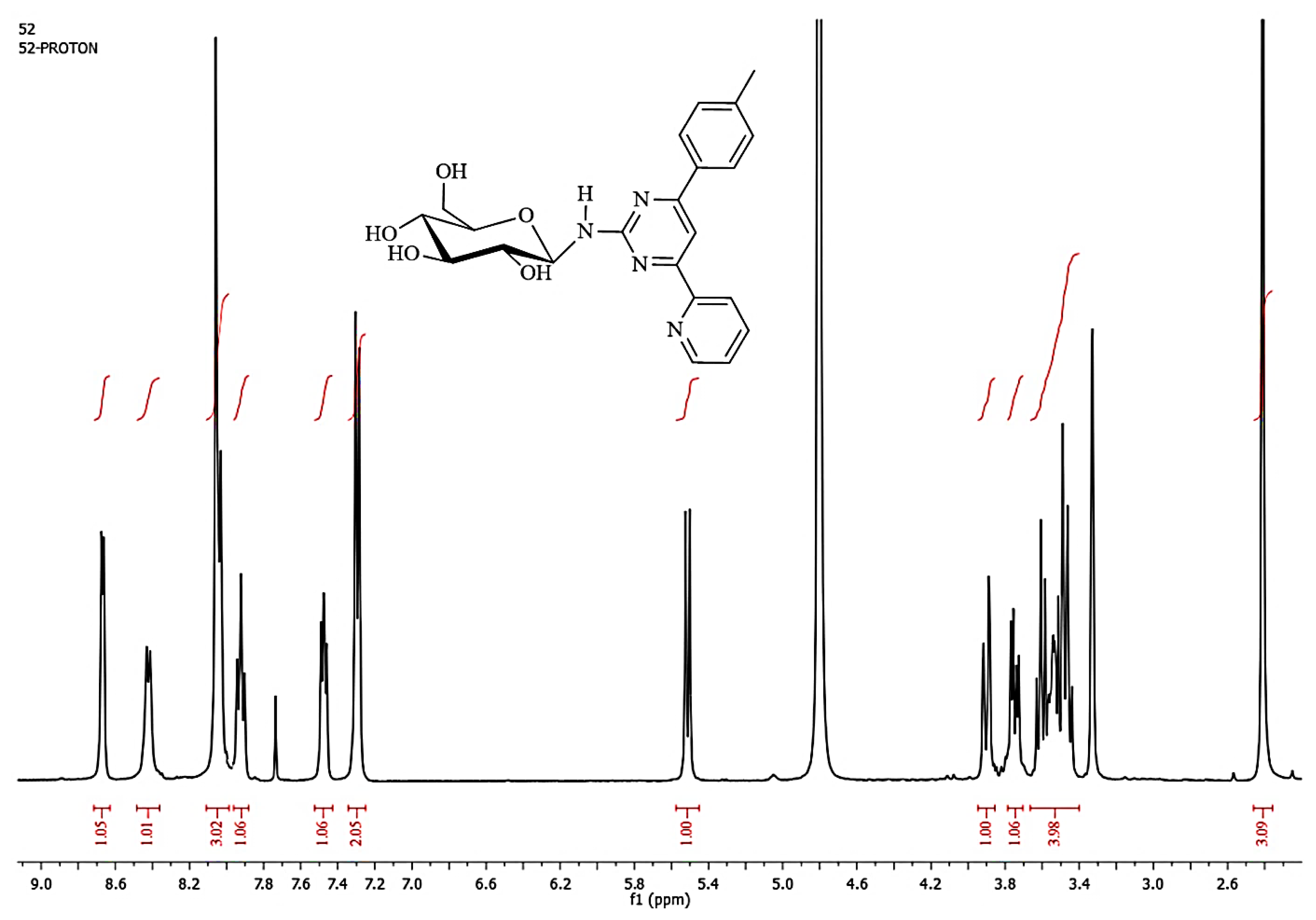

Supplement: Figure S25 — 1H-NMR spectrum of compound 7 (400 MHz, CDCl3/CD3OD (5:1)). [file turkjchem-47-2-476s25.tif]

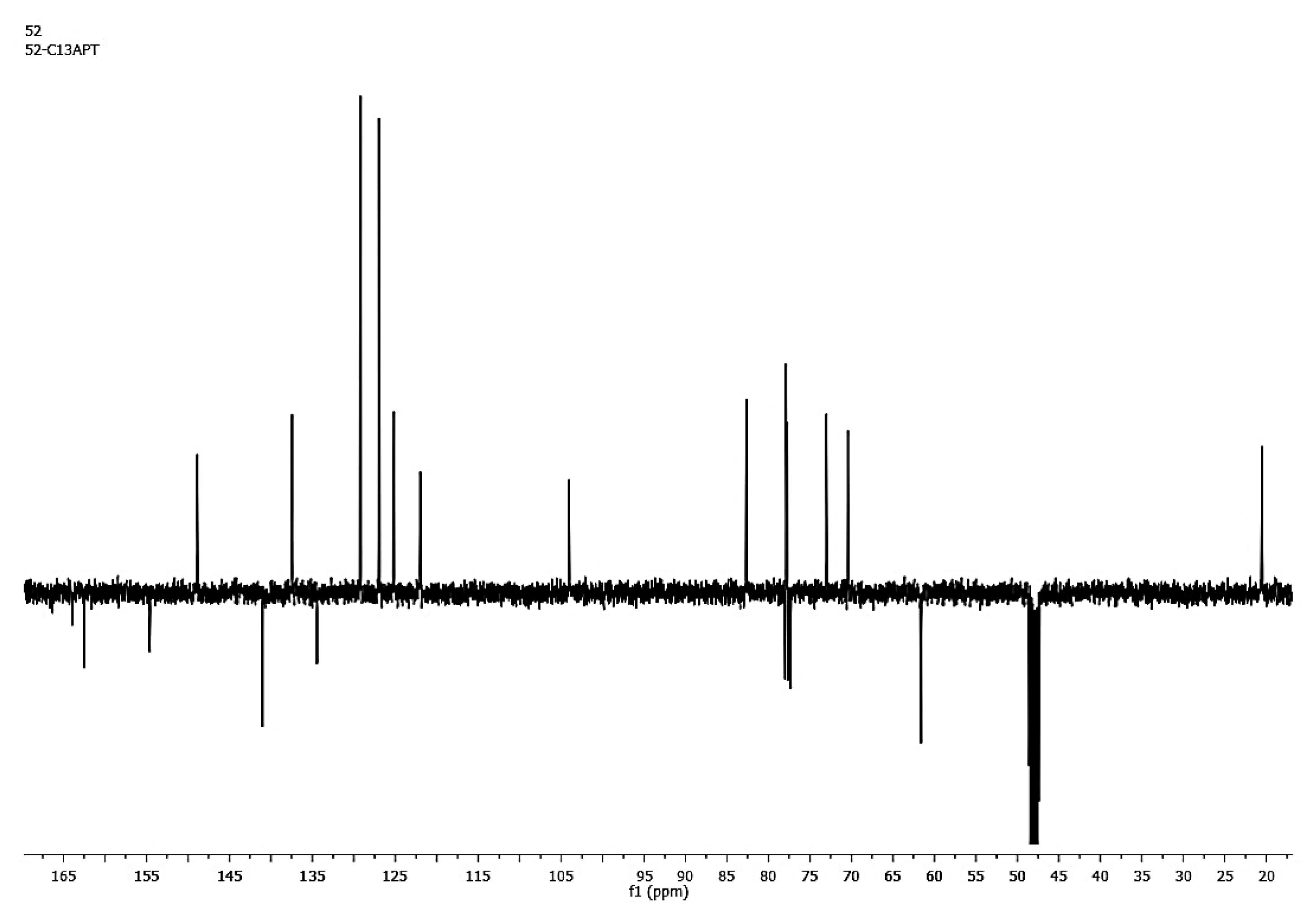

Supplement: Figure S26 — 13C-APT NMR spectrum of compound 7 (100 MHz, CDCl3/CD3OD (5:1)). [file turkjchem-47-2-476s26.tif]

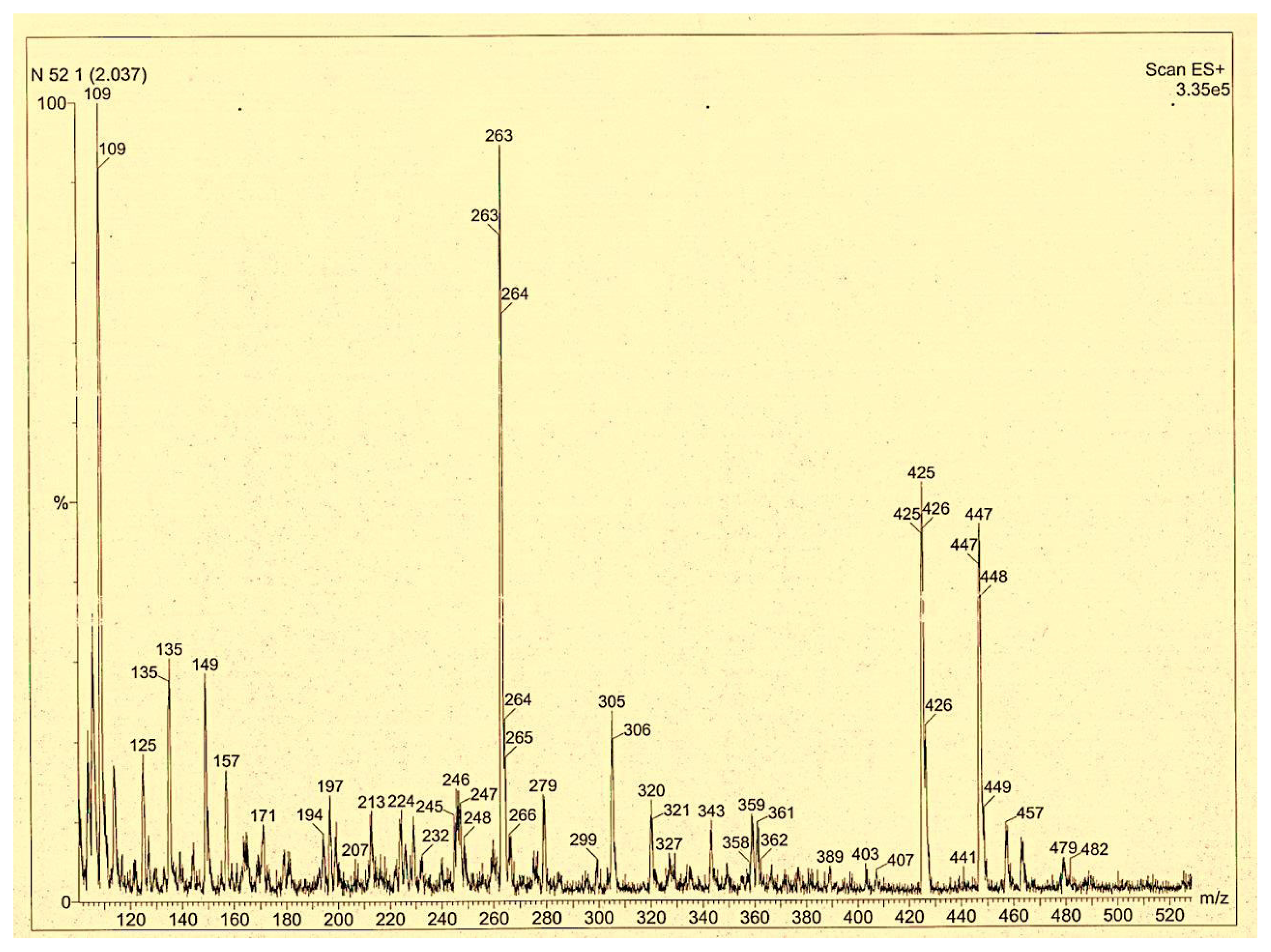

Supplement: Figure S27 — LC-MS/MS spectrum of compound 7. [file turkjchem-47-2-476s27.tif]

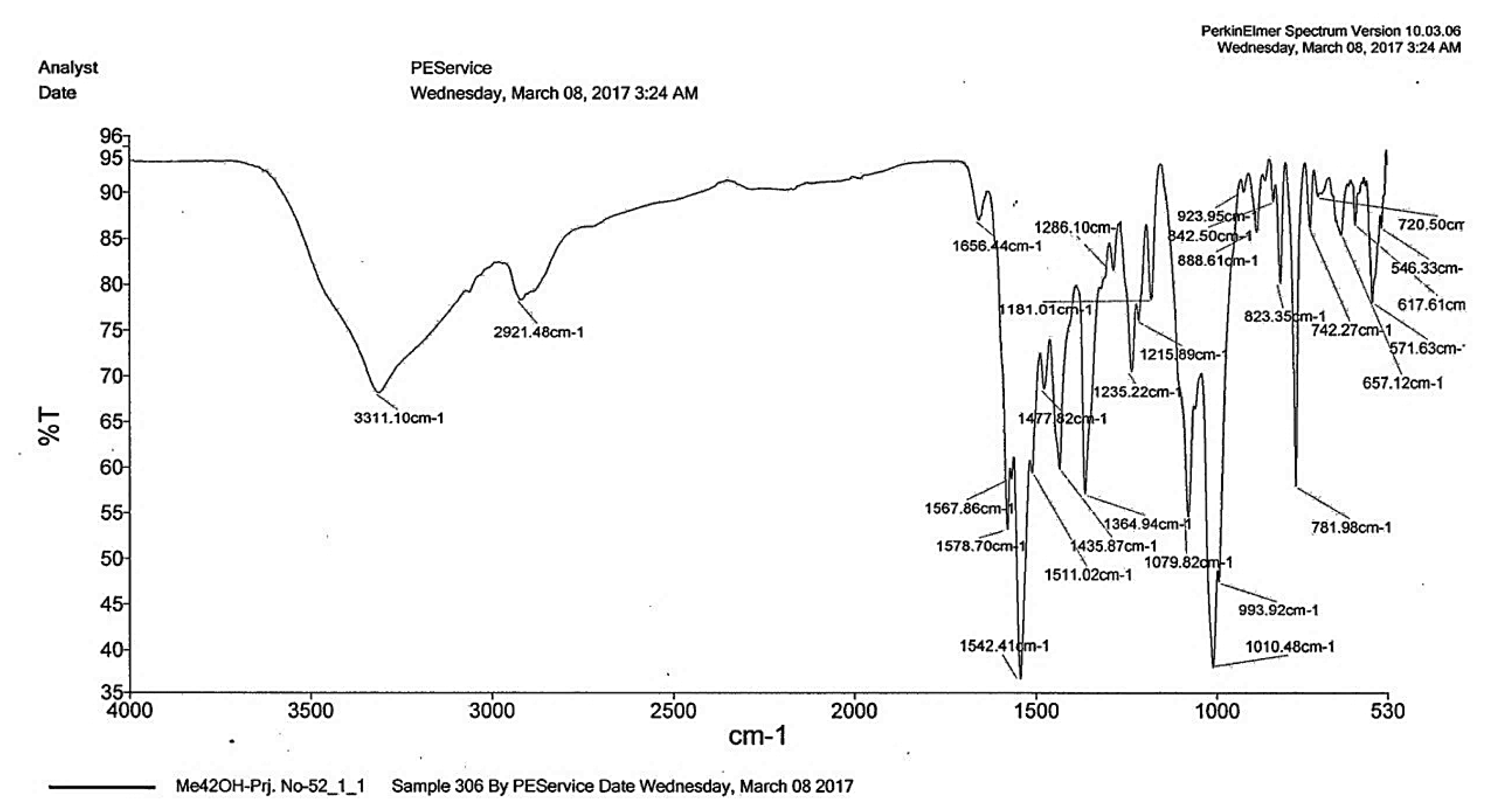

Supplement: Figure S28 — FT-IR spectrum of compound 7. [file turkjchem-47-2-476s28.tif]

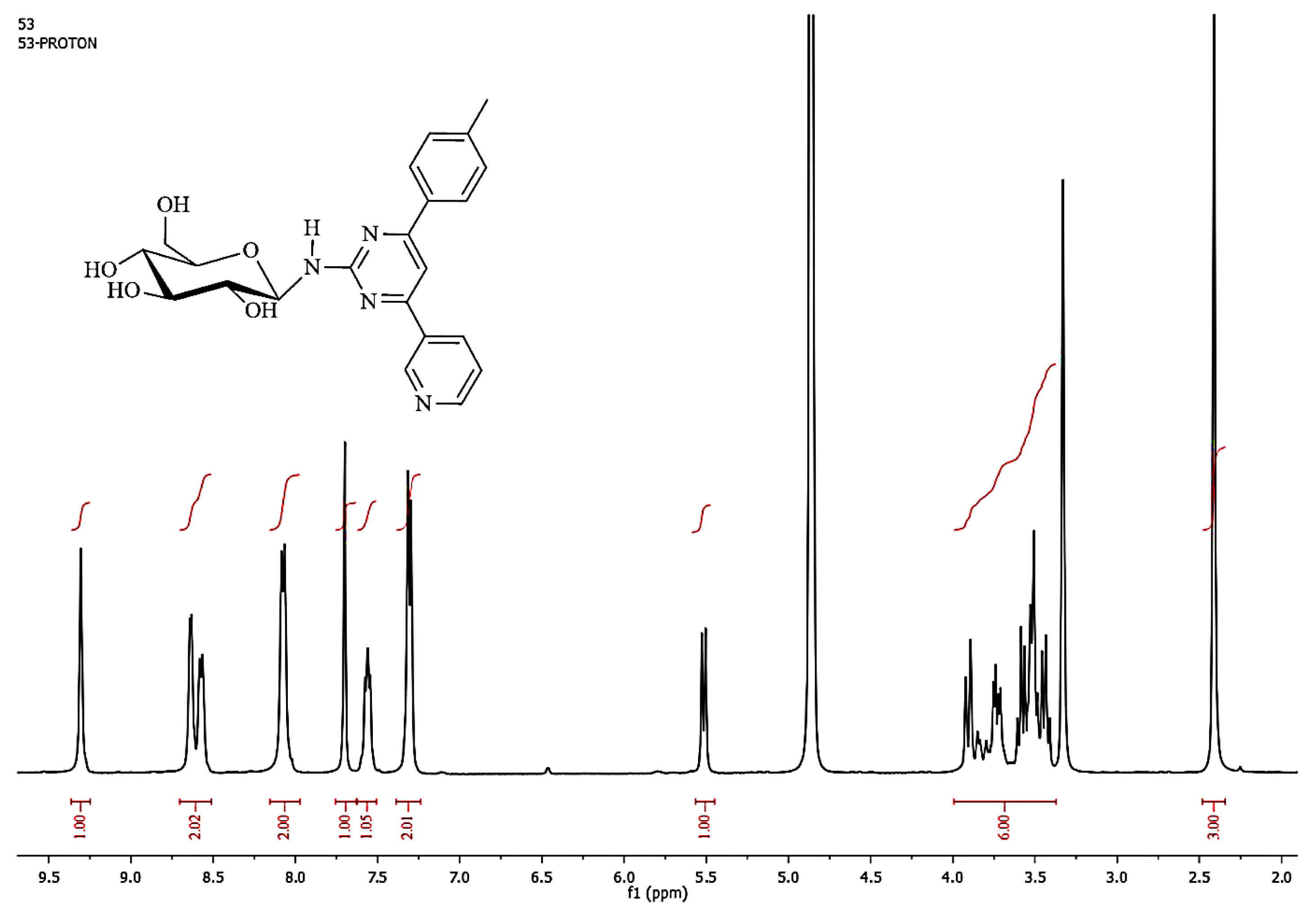

Supplement: Figure S29 — 1H-NMR spectrum of compound 8 (400 MHz, CD3OD). [file turkjchem-47-2-476s29.tif]

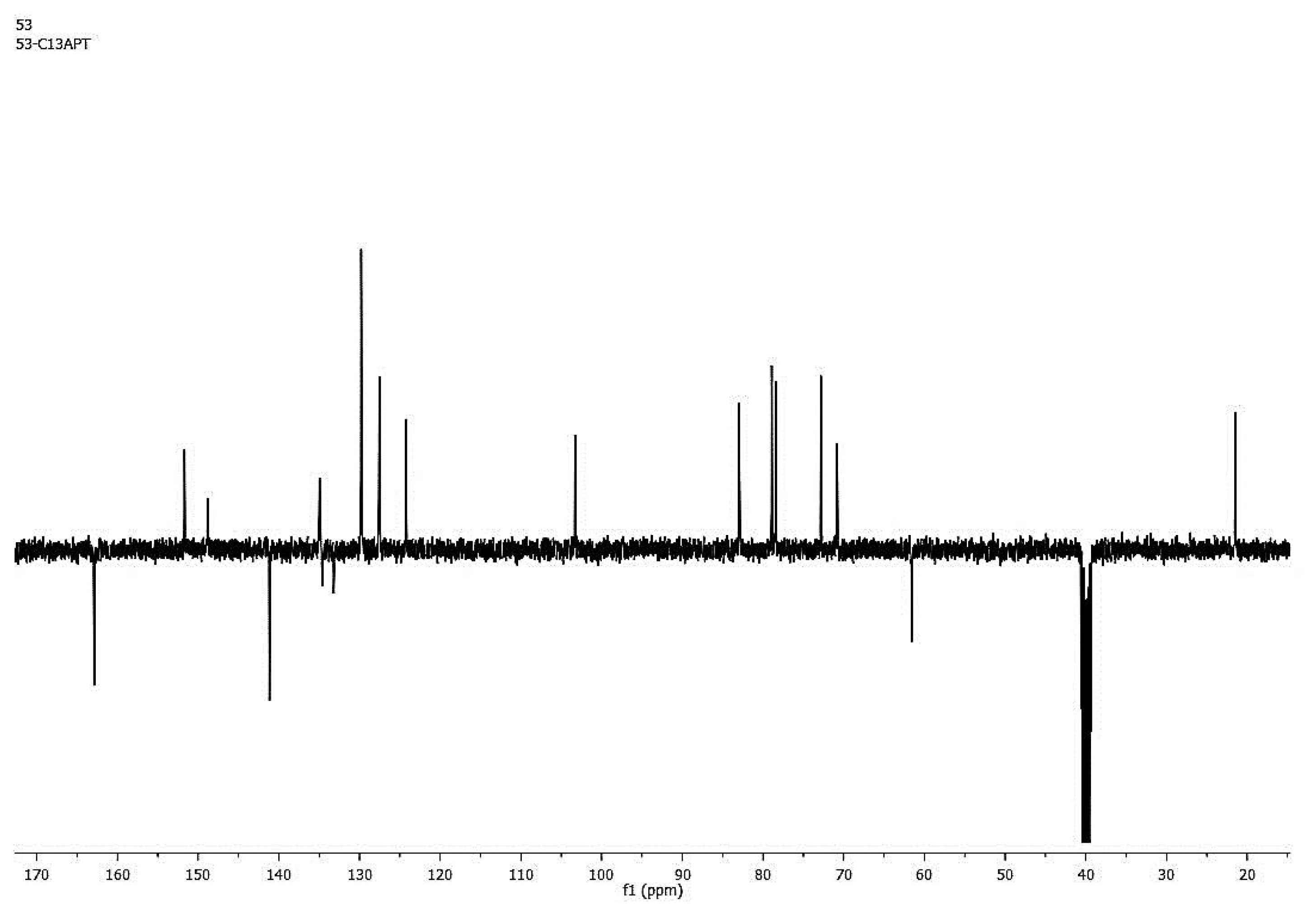

Supplement: Figure S30 — 13C-APT NMR spectrum of compound 8 (100 MHz, DMSO-d6). [file turkjchem-47-2-476s30.tif]

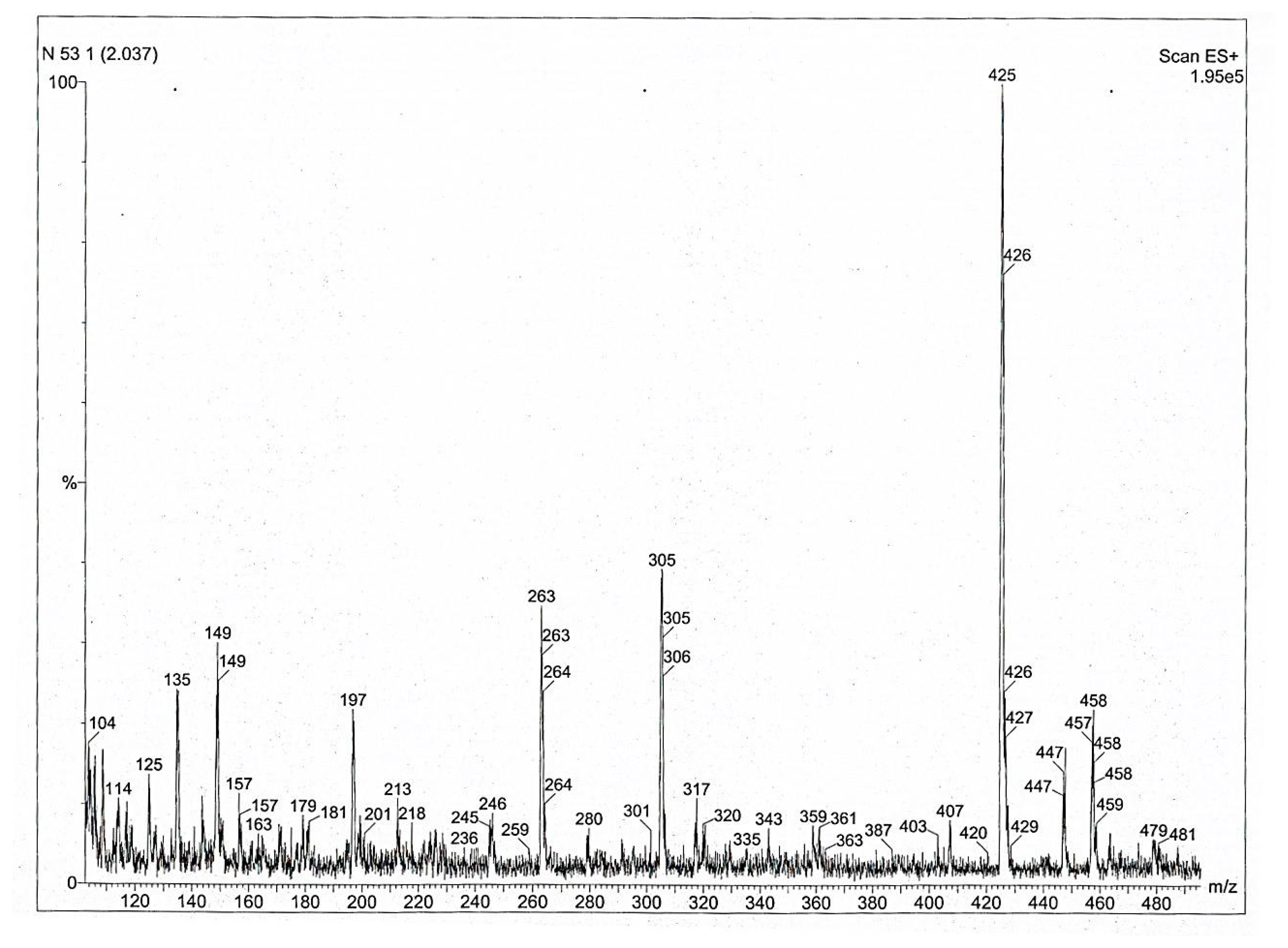

Supplement: Figure S31 — LC-MS/MS spectrum of compound 8. [file turkjchem-47-2-476s31.tif]

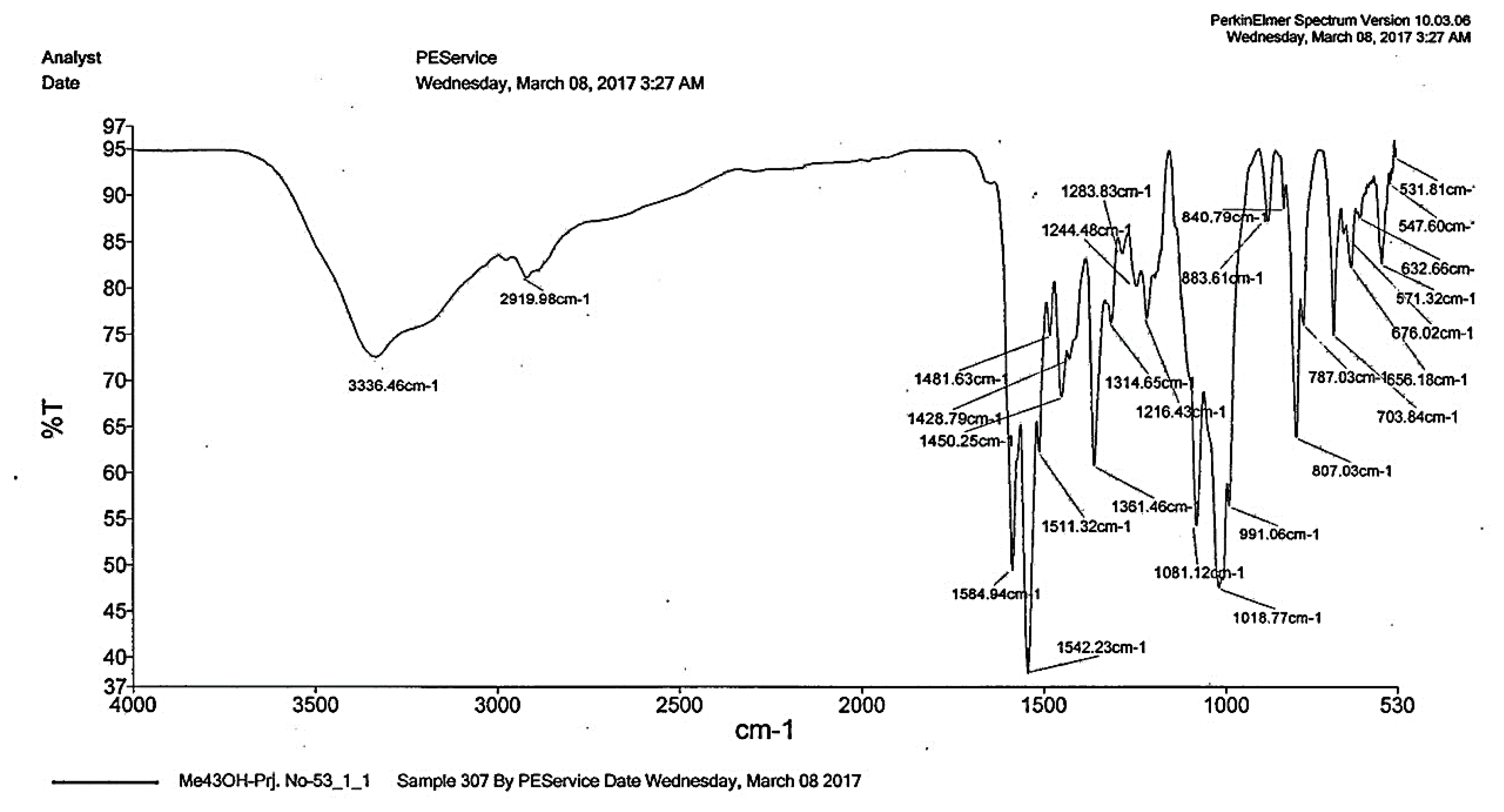

Supplement: Figure S32 — FT-IR spectrum of compound 8. [file turkjchem-47-2-476s32.tif]

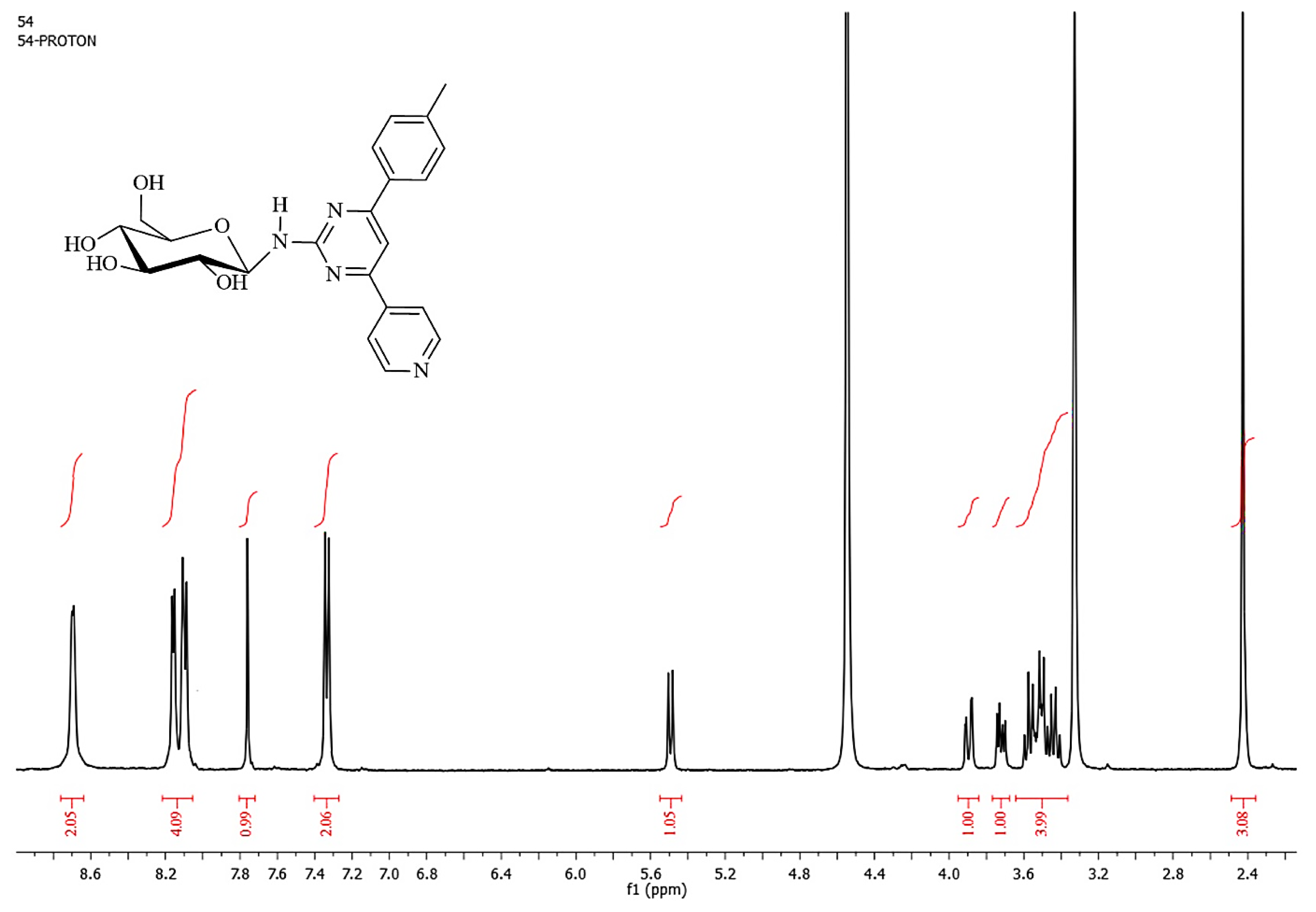

Supplement: Figure S33 — 1H-NMR spectrum of compound 9 (400 MHz, CD3OD). [file turkjchem-47-2-476s33.tif]

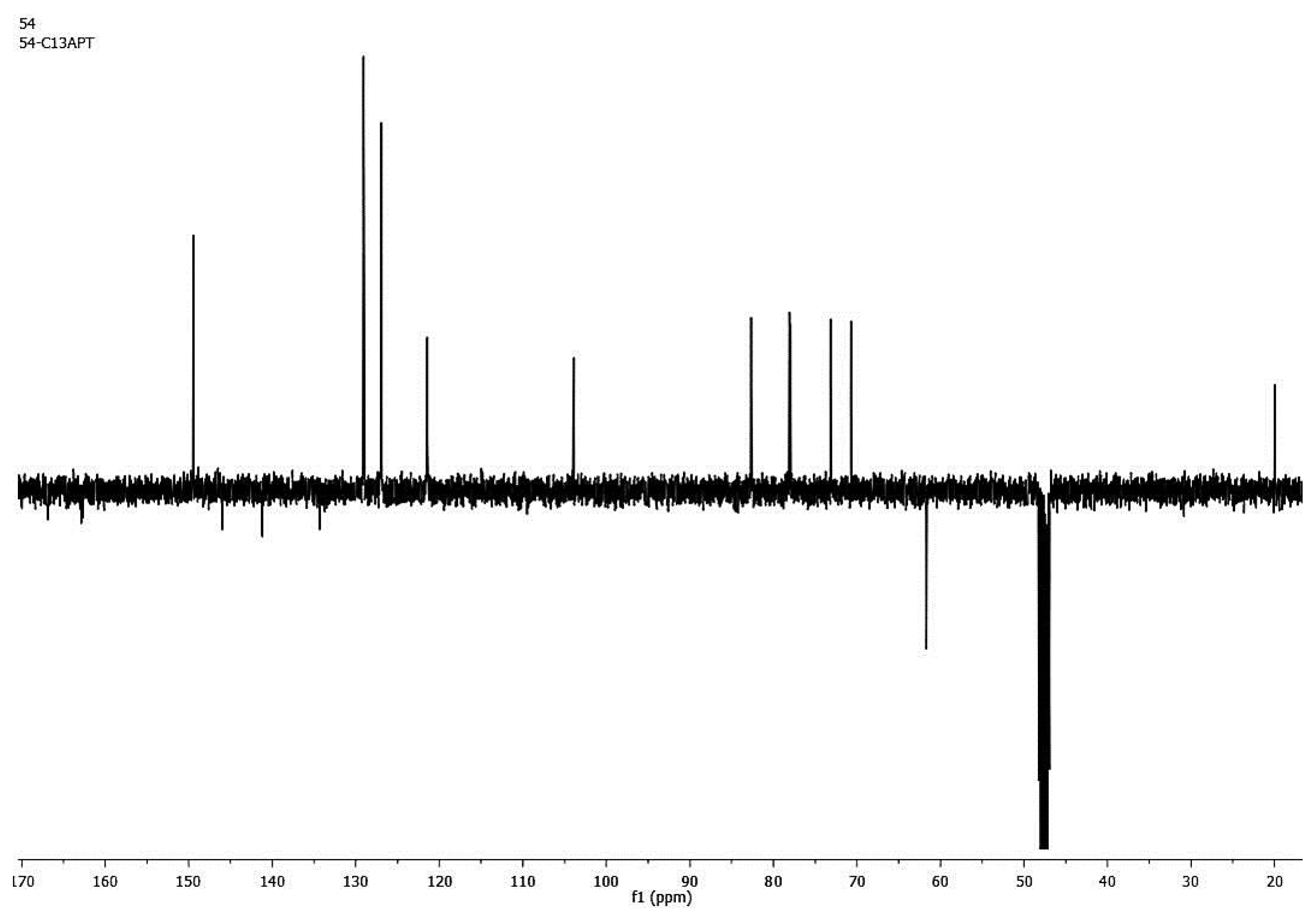

Supplement: Figure S34 — 13C-APT NMR spectrum of compound 9 (100 MHz, CD3OD). [file turkjchem-47-2-476s34.tif]

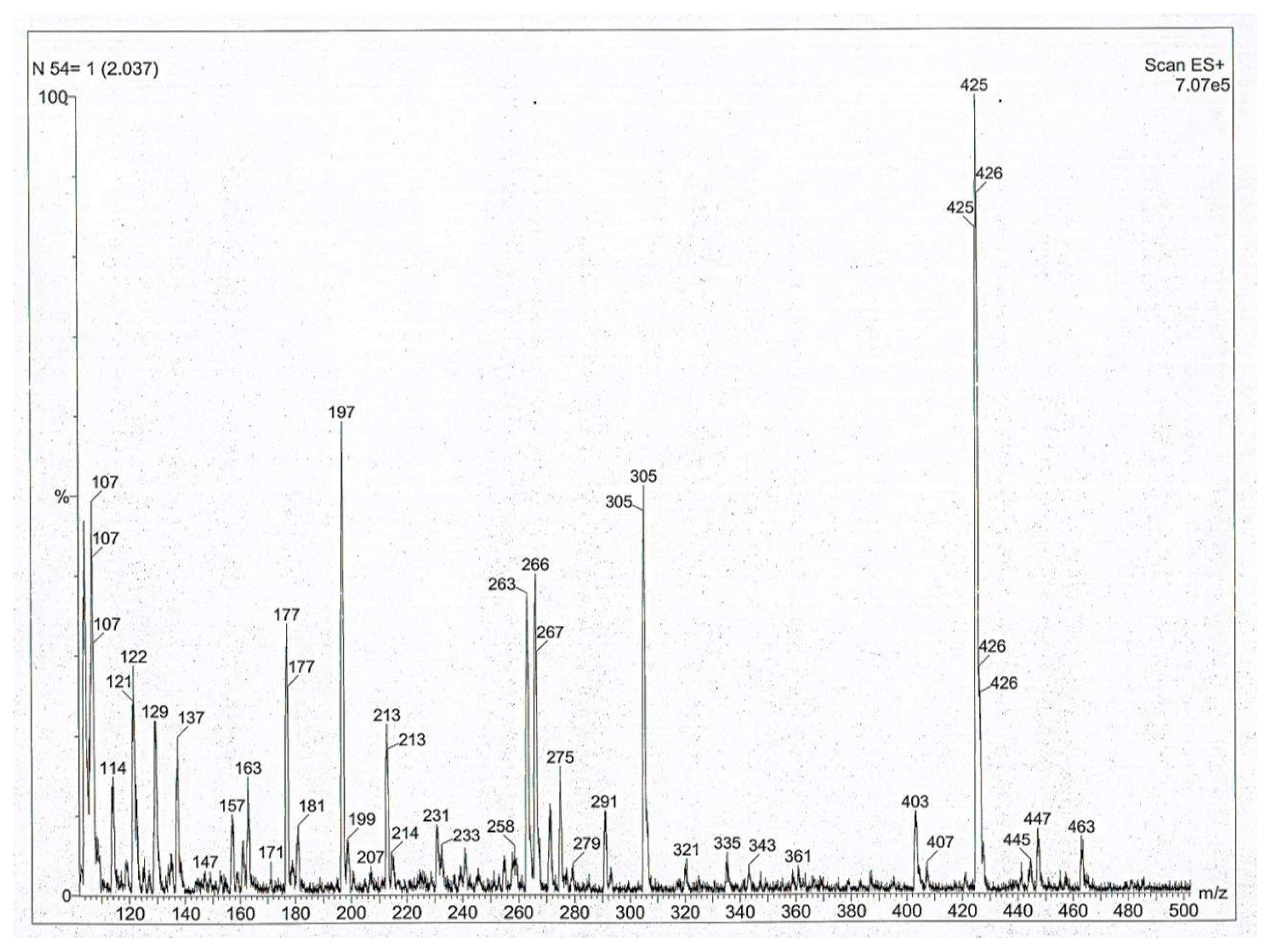

Supplement: Figure S35 — LC-MS/MS spectrum of compound 9. [file turkjchem-47-2-476s35.tif]

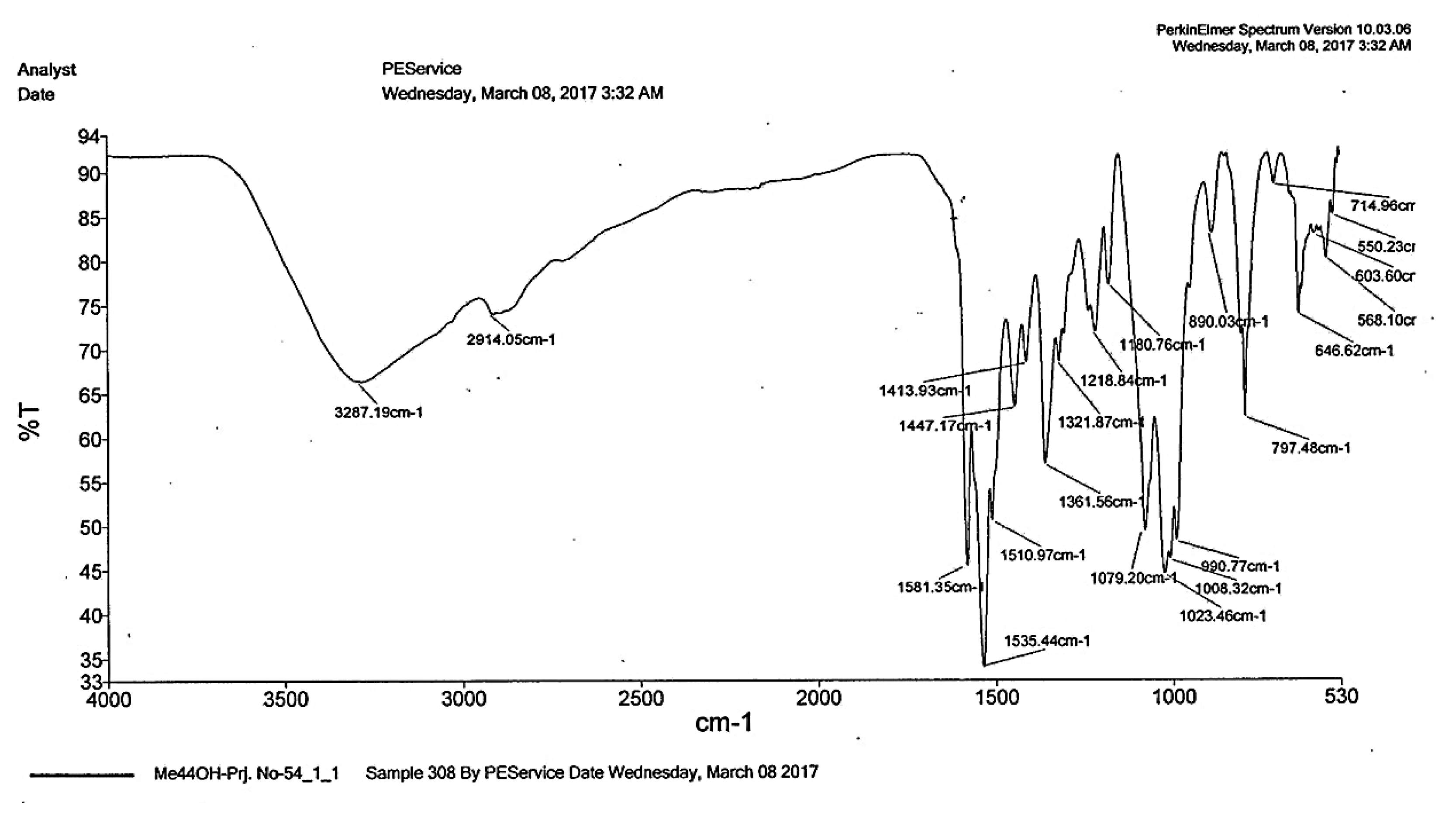

Supplement: Figure S36 — FT-IR spectrum of compound 9. [file turkjchem-47-2-476s36.tif]

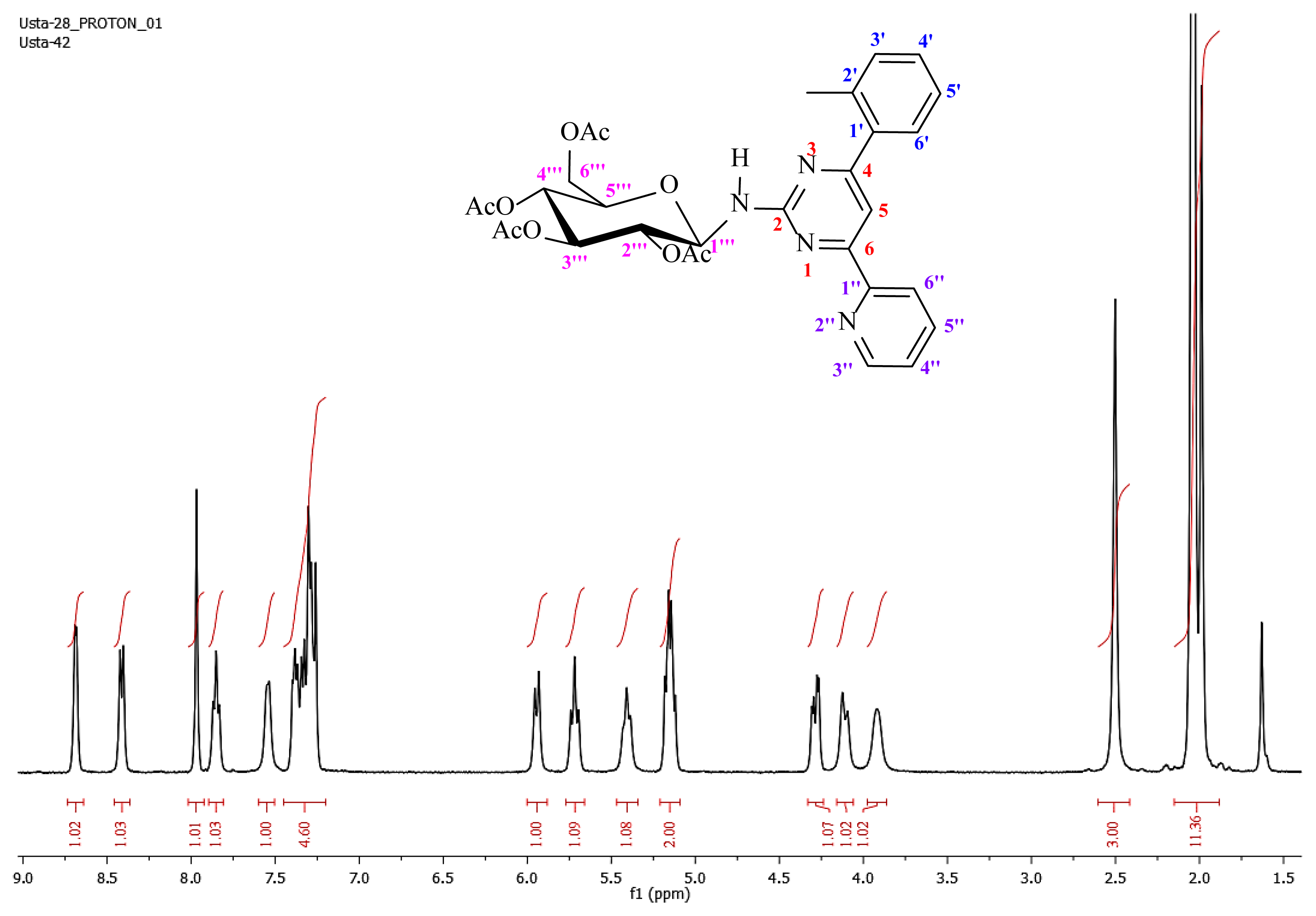

Supplement: Figure S37 — 1H-NMR spectrum of compound 10 (400 MHz, CDCl3, ppm). [file turkjchem-47-2-476s37.tif]

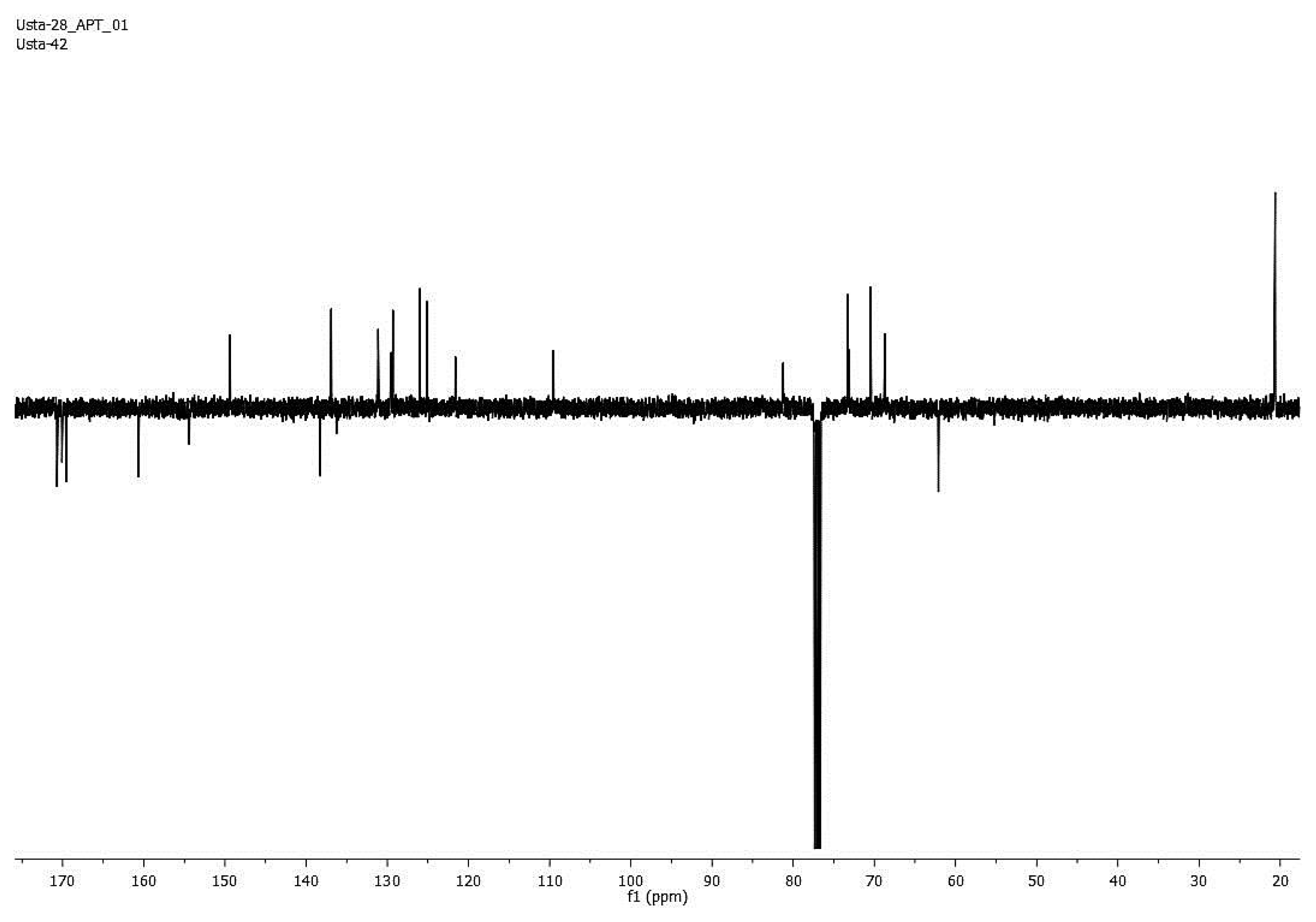

Supplement: Figure S38 — 13C-APT NMR spectrum of compound 10 (100 MHz, CDCl3, ppm). [file turkjchem-47-2-476s38.tif]

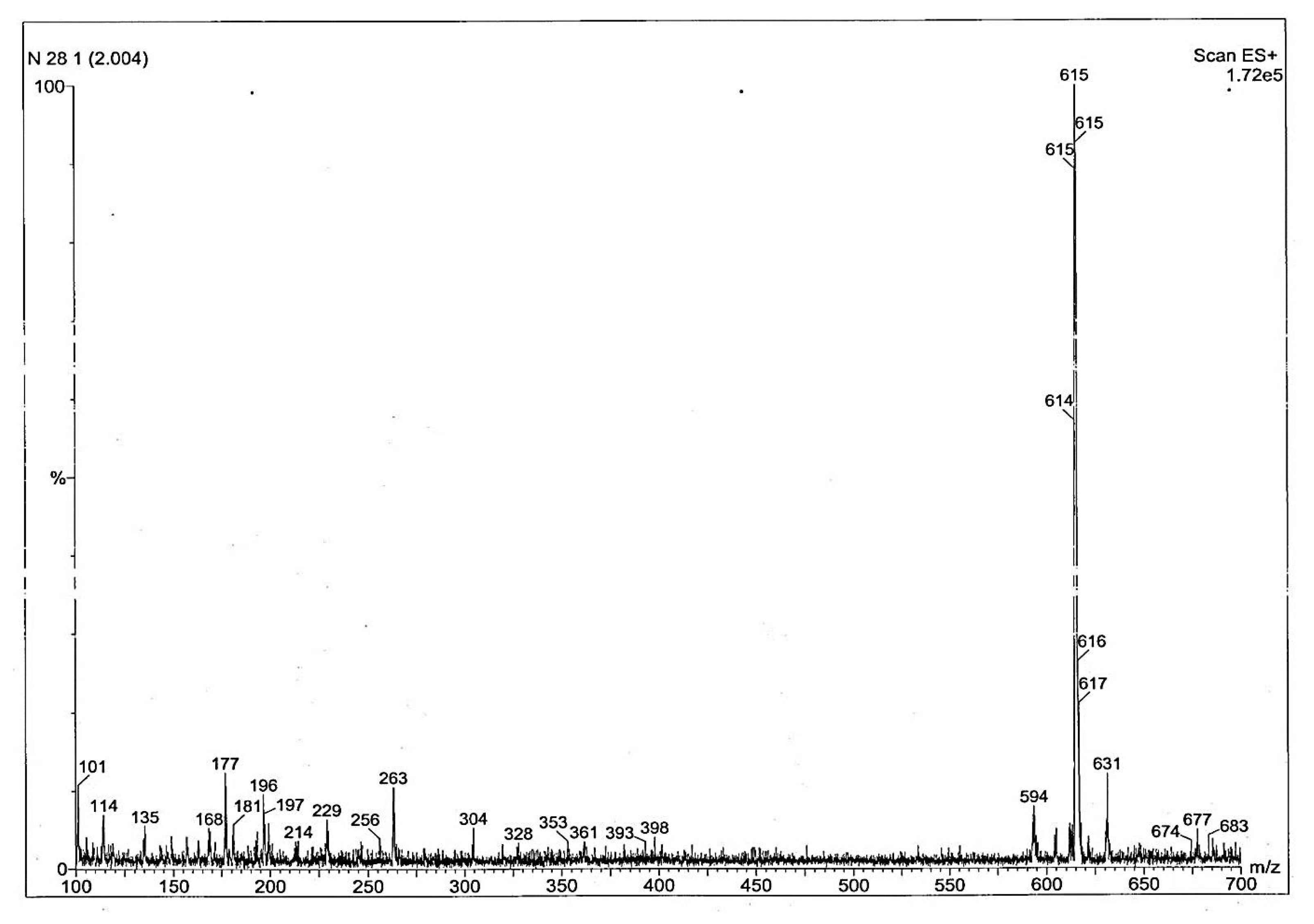

Supplement: Figure S39 — LC-MS/MS spectrum of compound 10. [file turkjchem-47-2-476s39.tif]

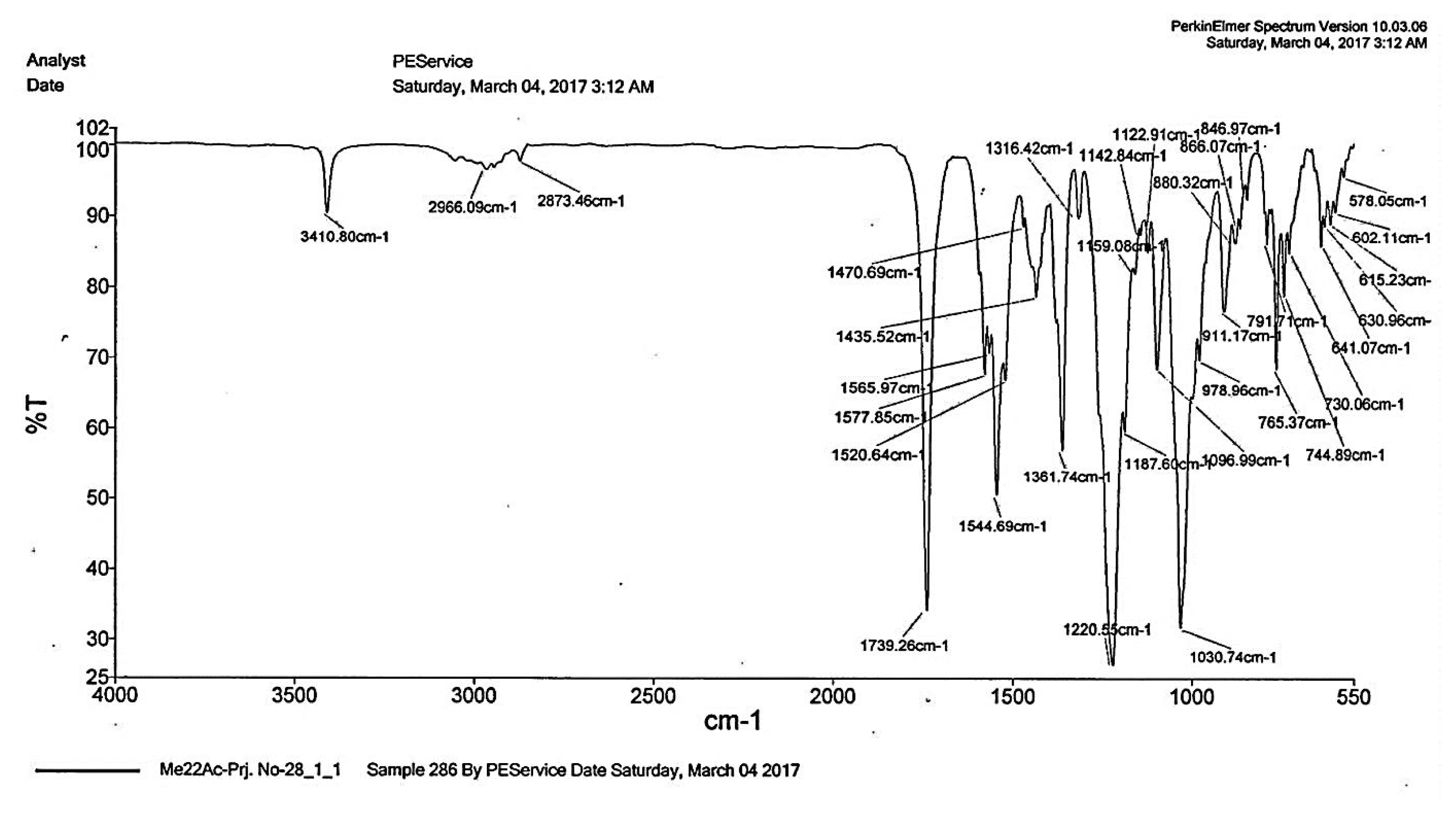

Supplement: Figure S40 — FT-IR spectrum of compound 10. [file turkjchem-47-2-476s40.tif]

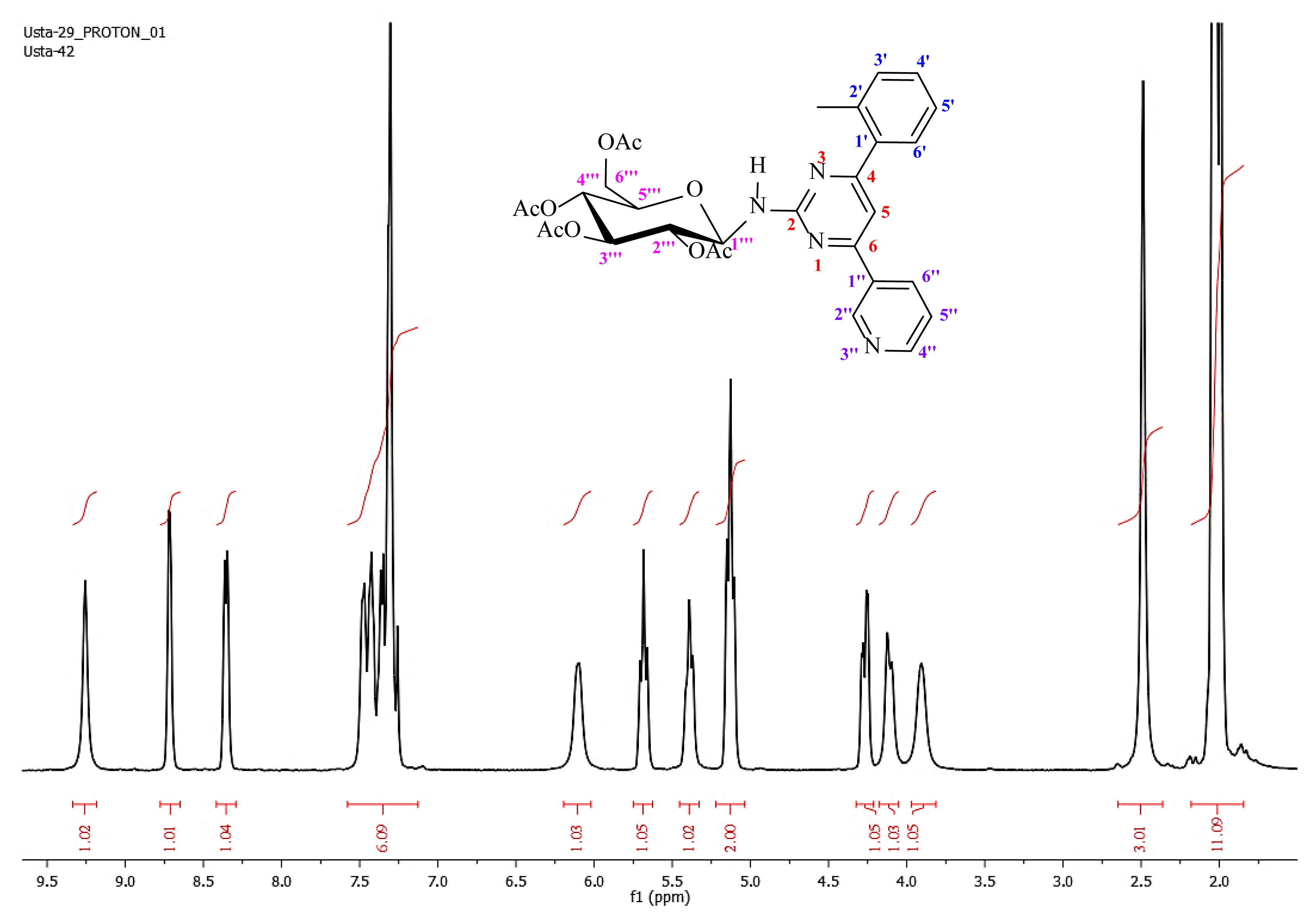

Supplement: Figure S41 — 1H-NMR spectrum of compound 11 (400 MHz, CDCl3, ppm). [file turkjchem-47-2-476s41.tif]

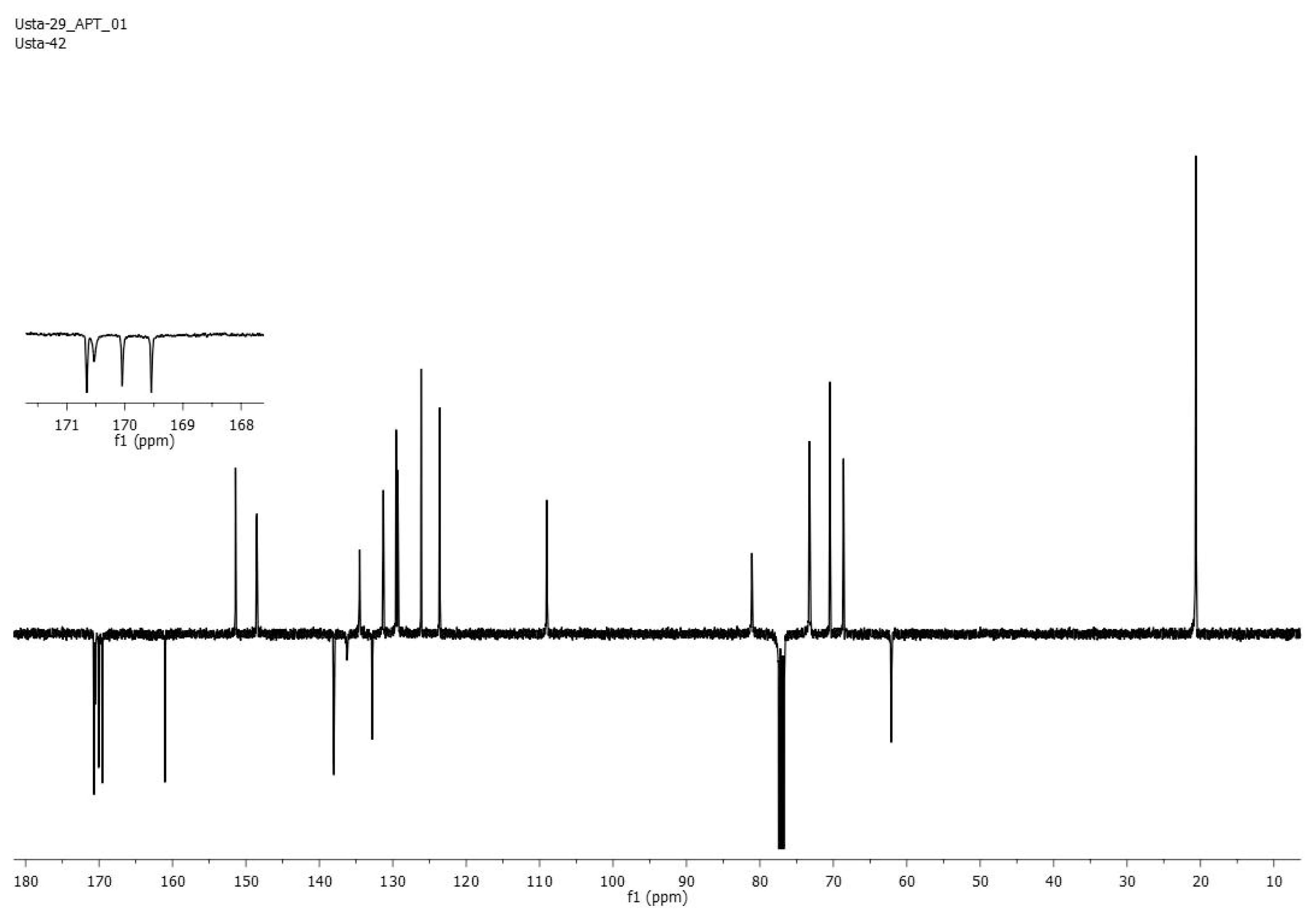

Supplement: Figure S42 — 13C-APT NMR spectrum of compound 11 (100 MHz, CDCl3, ppm). [file turkjchem-47-2-476s42.tif]

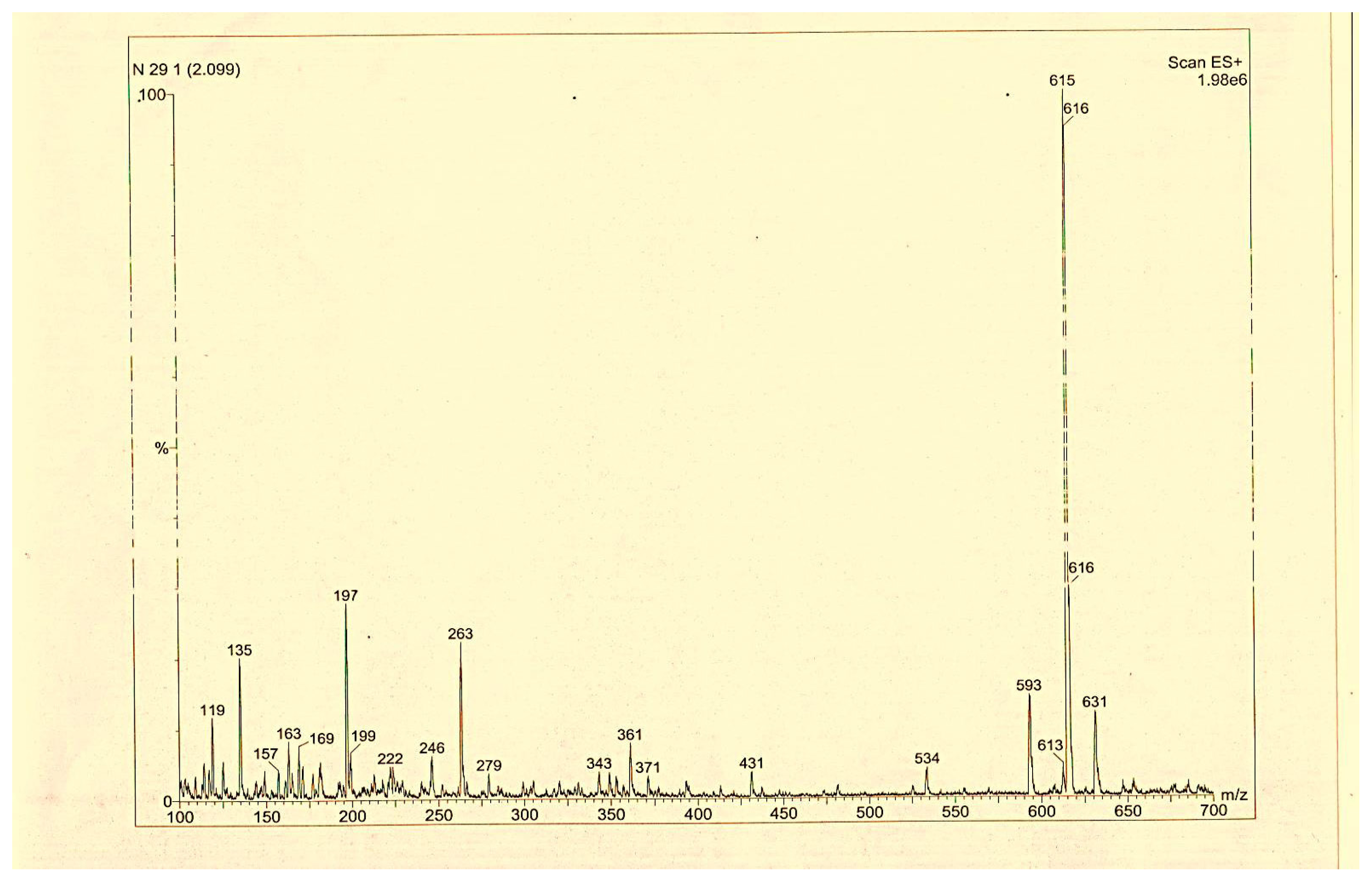

Supplement: Figure S43 — LC-MS/MS spectrum of compound 11. [file turkjchem-47-2-476s43.tif]

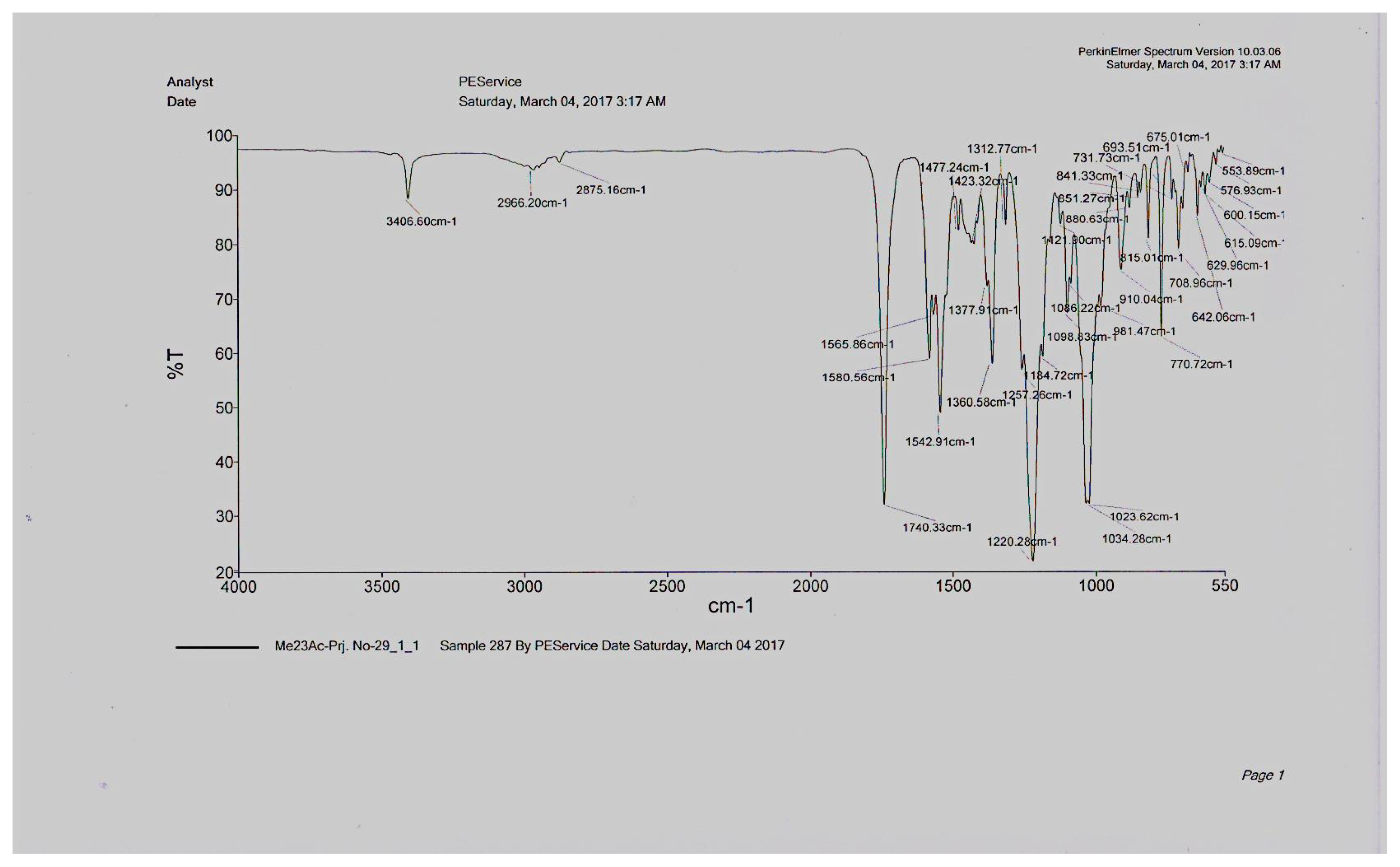

Supplement: Figure S44 — FT-IR spectrum of compound 11. [file turkjchem-47-2-476s44.tif]

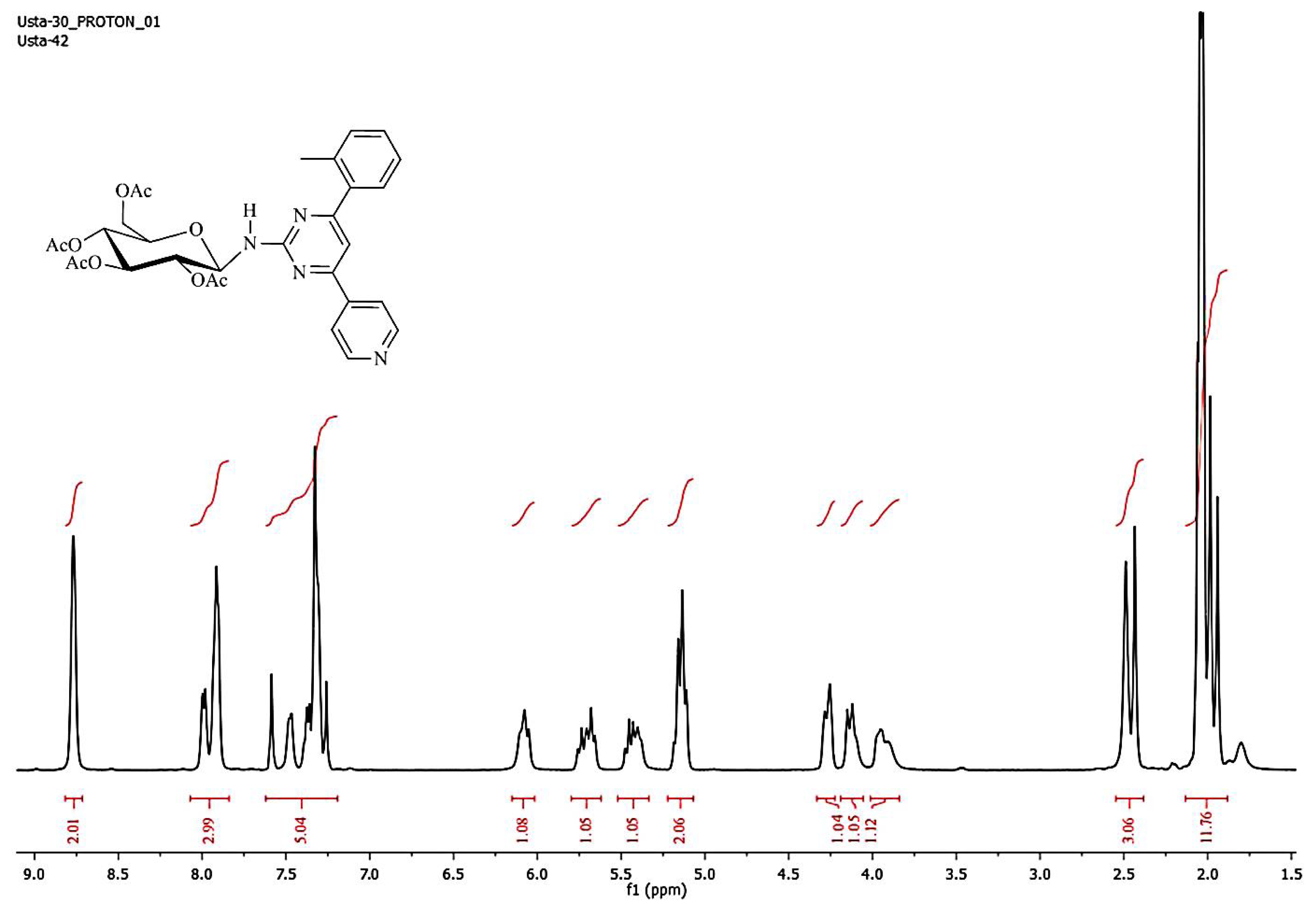

Supplement: Figure S45 — 1H-NMR spectrum of compound 12 (400 MHz, CDCl3, ppm). [file turkjchem-47-2-476s45.tif]

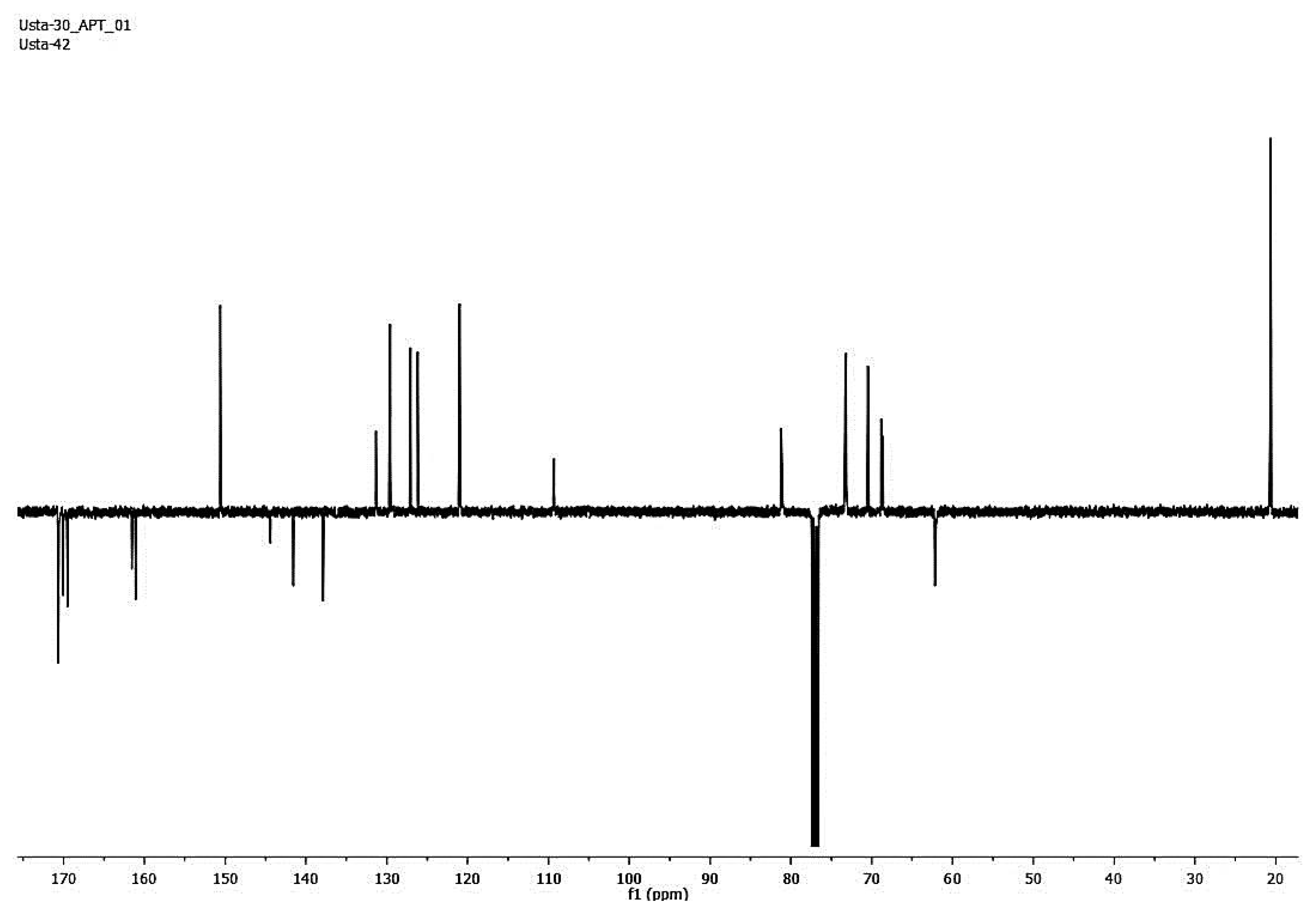

Supplement: Figure S46 — 13C-APT NMR spectrum of compound 12 (100 MHz, CDCl3, ppm). [file turkjchem-47-2-476s46.tif]

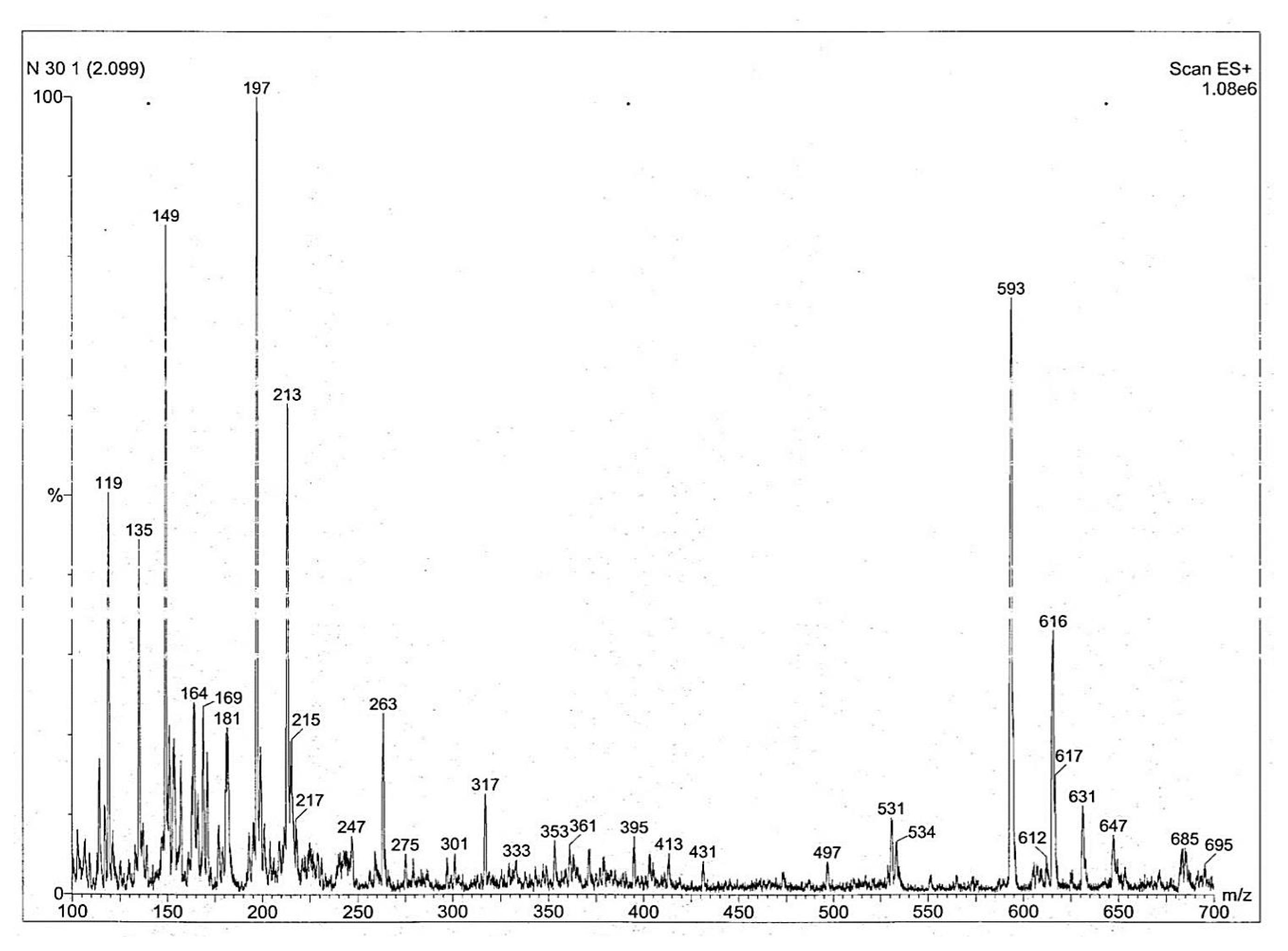

Supplement: Figure S47 — LC-MS/MS spectrum of compound 12. [file turkjchem-47-2-476s47.tif]

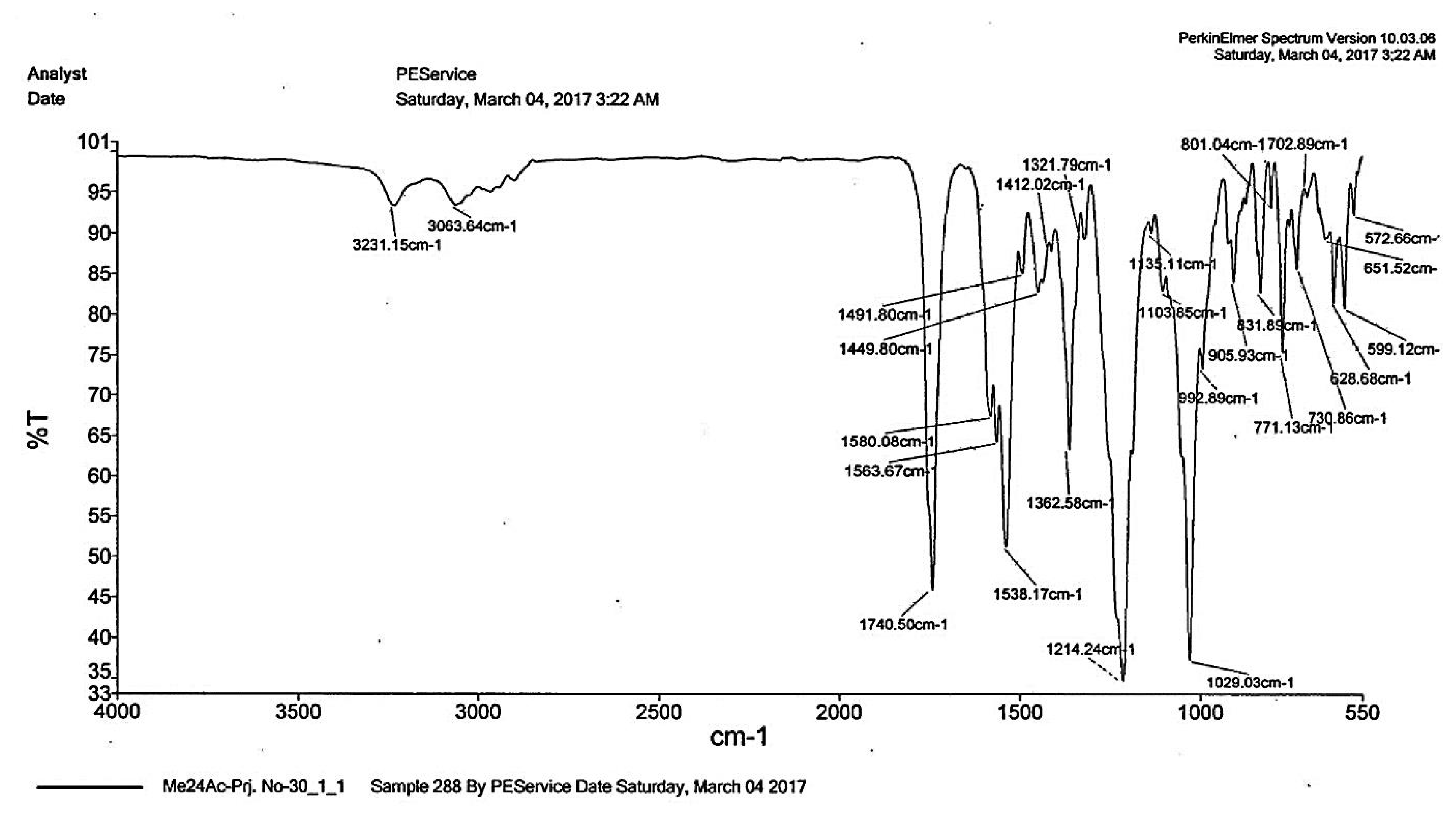

Supplement: Figure S48 — FT-IR spectrum of compound 12. [file turkjchem-47-2-476s48.tif]

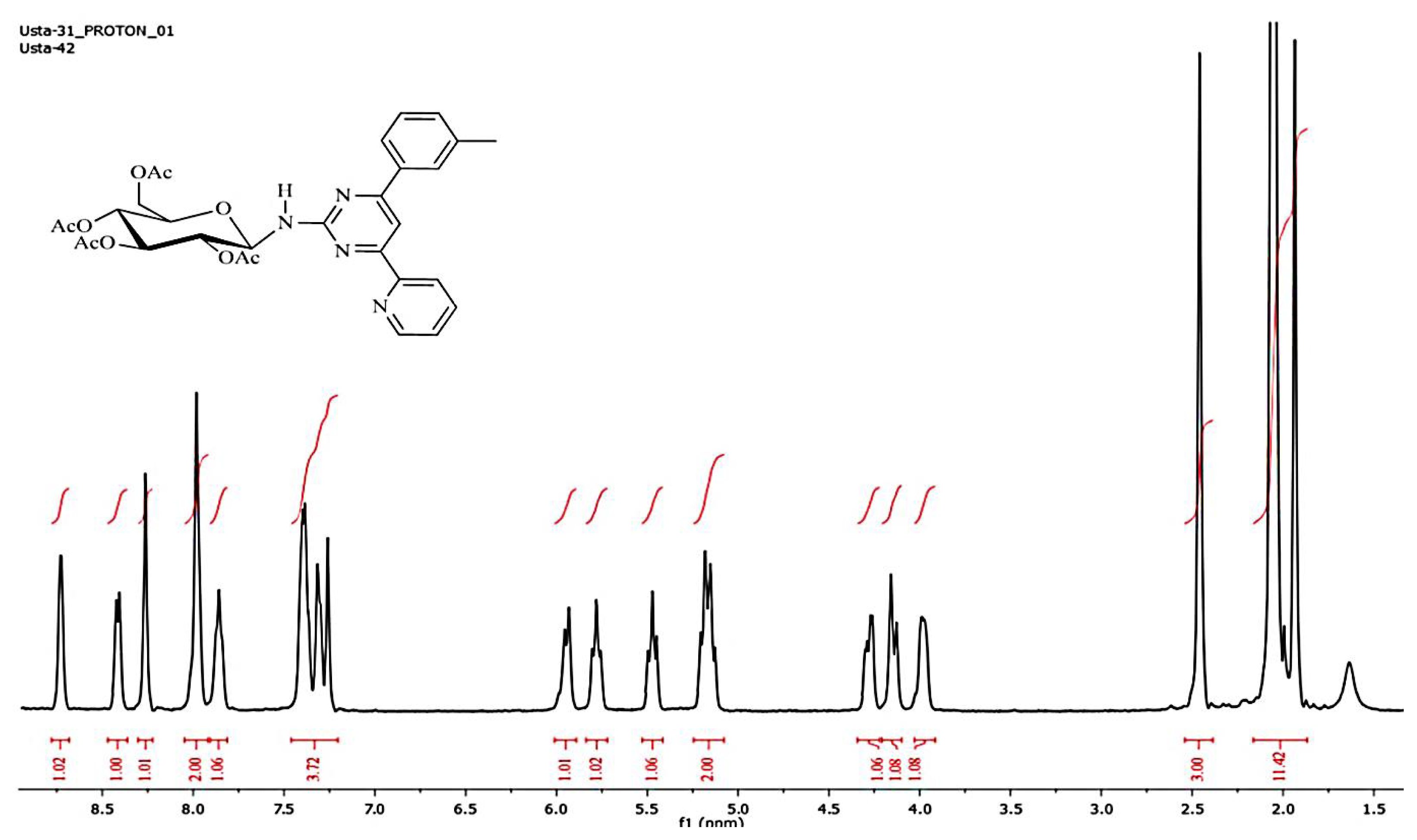

Supplement: Figure S49 — 1H-NMR spectrum of compound 13 (400 MHz, CDCl3, ppm). [file turkjchem-47-2-476s49.tif]

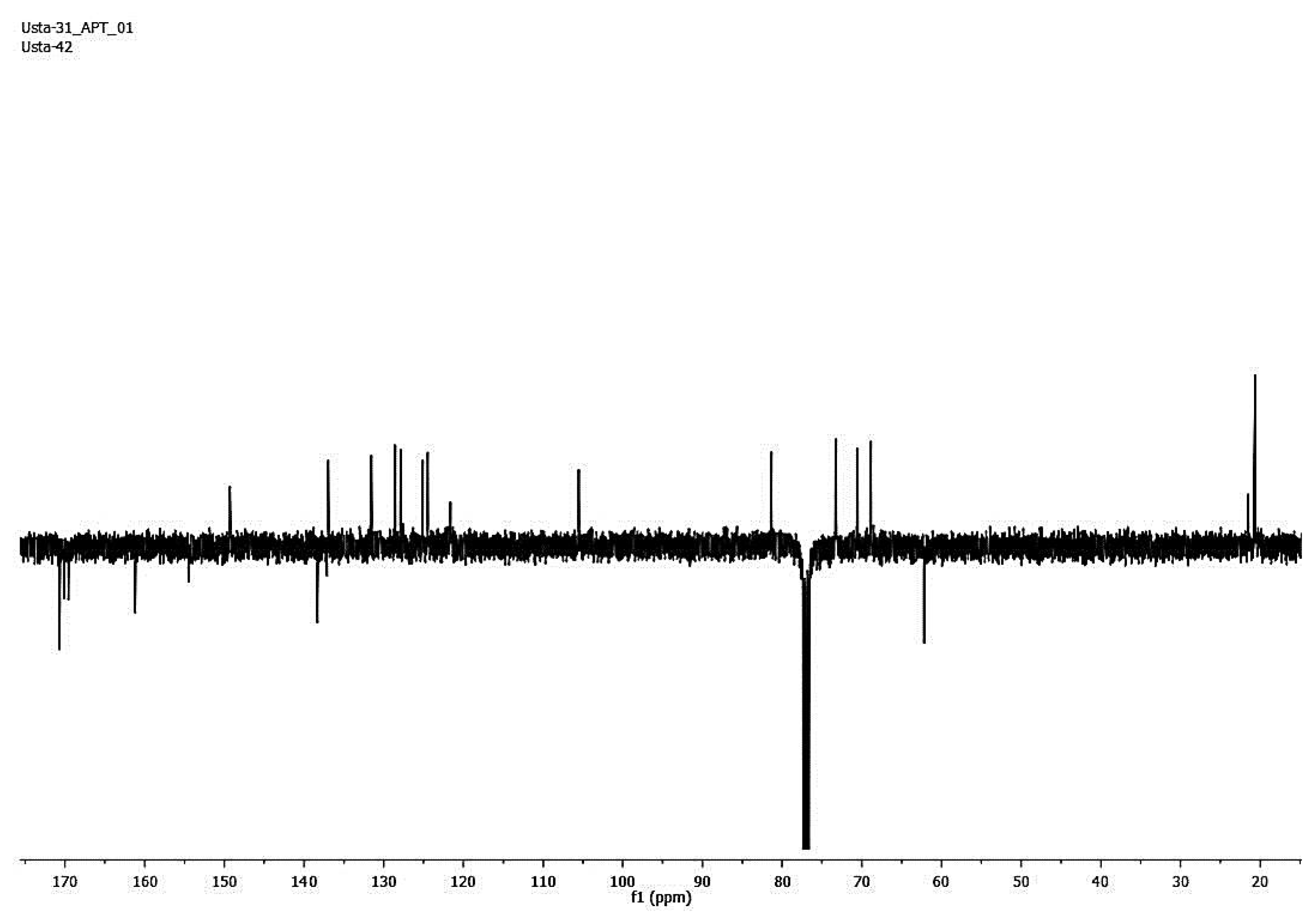

Supplement: Figure S50 — 13C-APT NMR spectrum of compound 13 (100 MHz, CDCl3, ppm). [file turkjchem-47-2-476s50.tif]

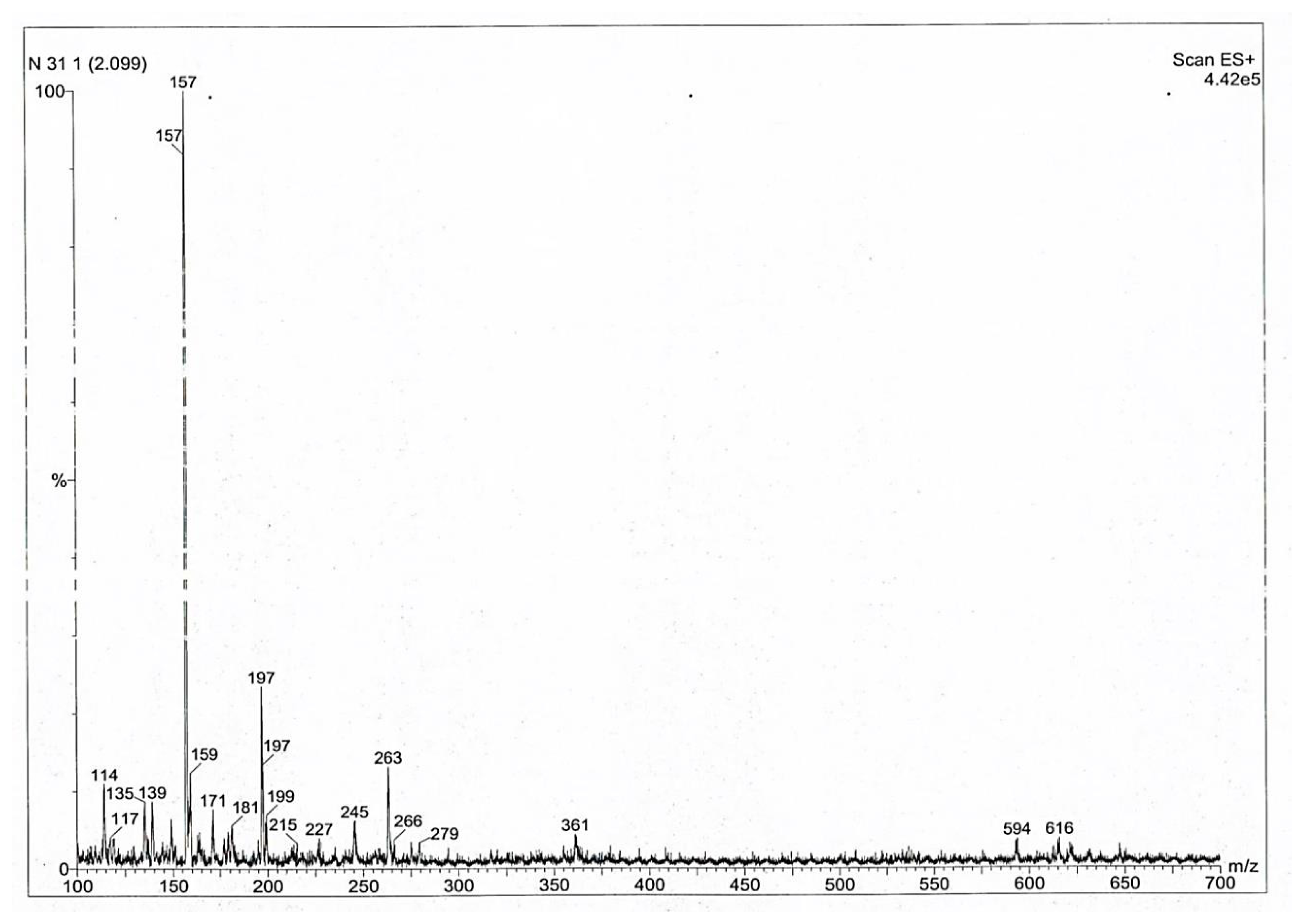

Supplement: Figure S51 — LC-MS/MS spectrum of compound 13. [file turkjchem-47-2-476s51.tif]

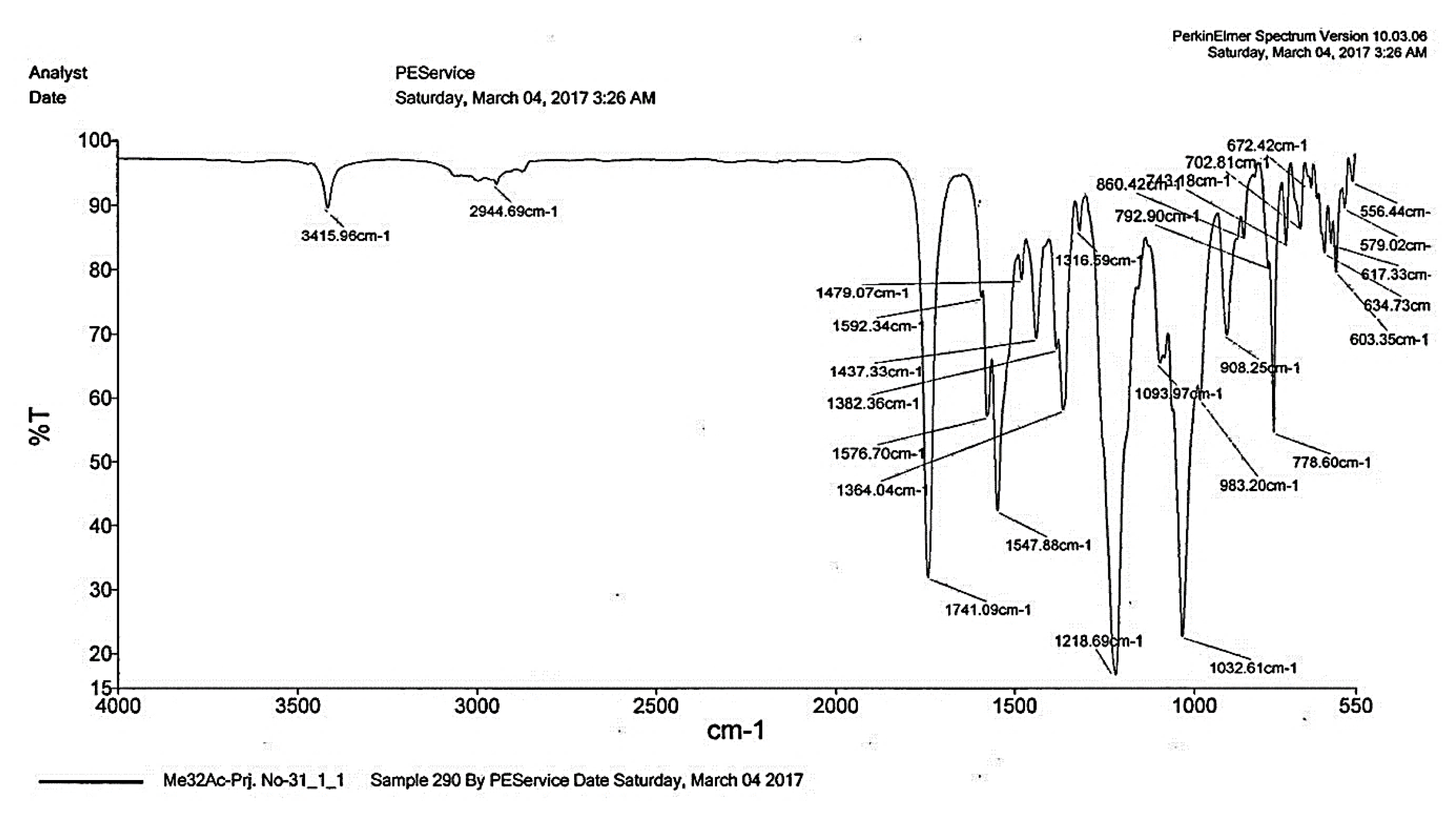

Supplement: Figure S52 — FT-IR spectrum of compound 13. [file turkjchem-47-2-476s52.tif]

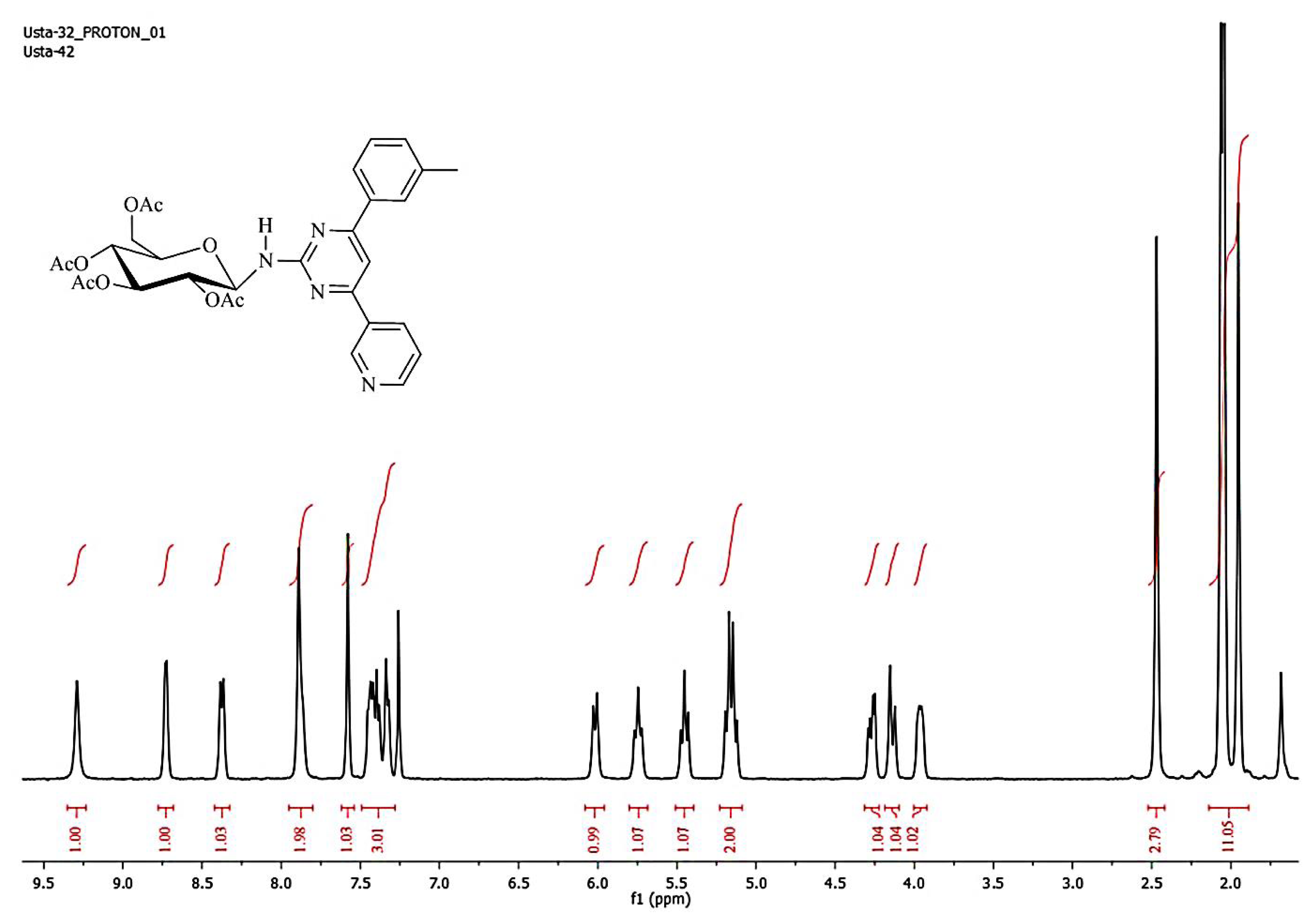

Supplement: Figure S53 — 1H-NMR spectrum of compound 14 (400 MHz, CDCl3, ppm). [file turkjchem-47-2-476s53.tif]

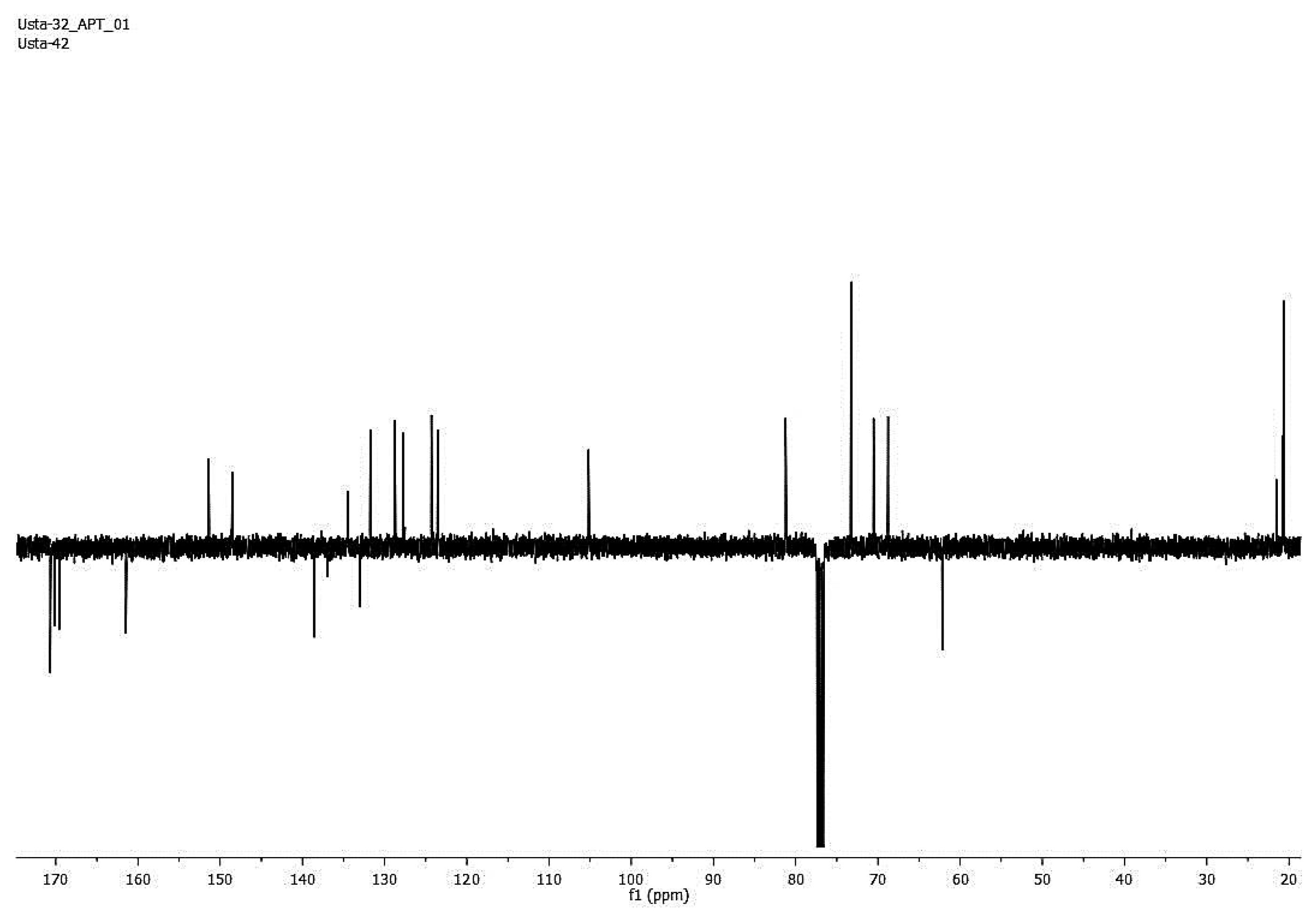

Supplement: Figure S54 — 13C-APT NMR spectrum of compound 14 (100 MHz, CDCl3, ppm). [file turkjchem-47-2-476s54.tif]

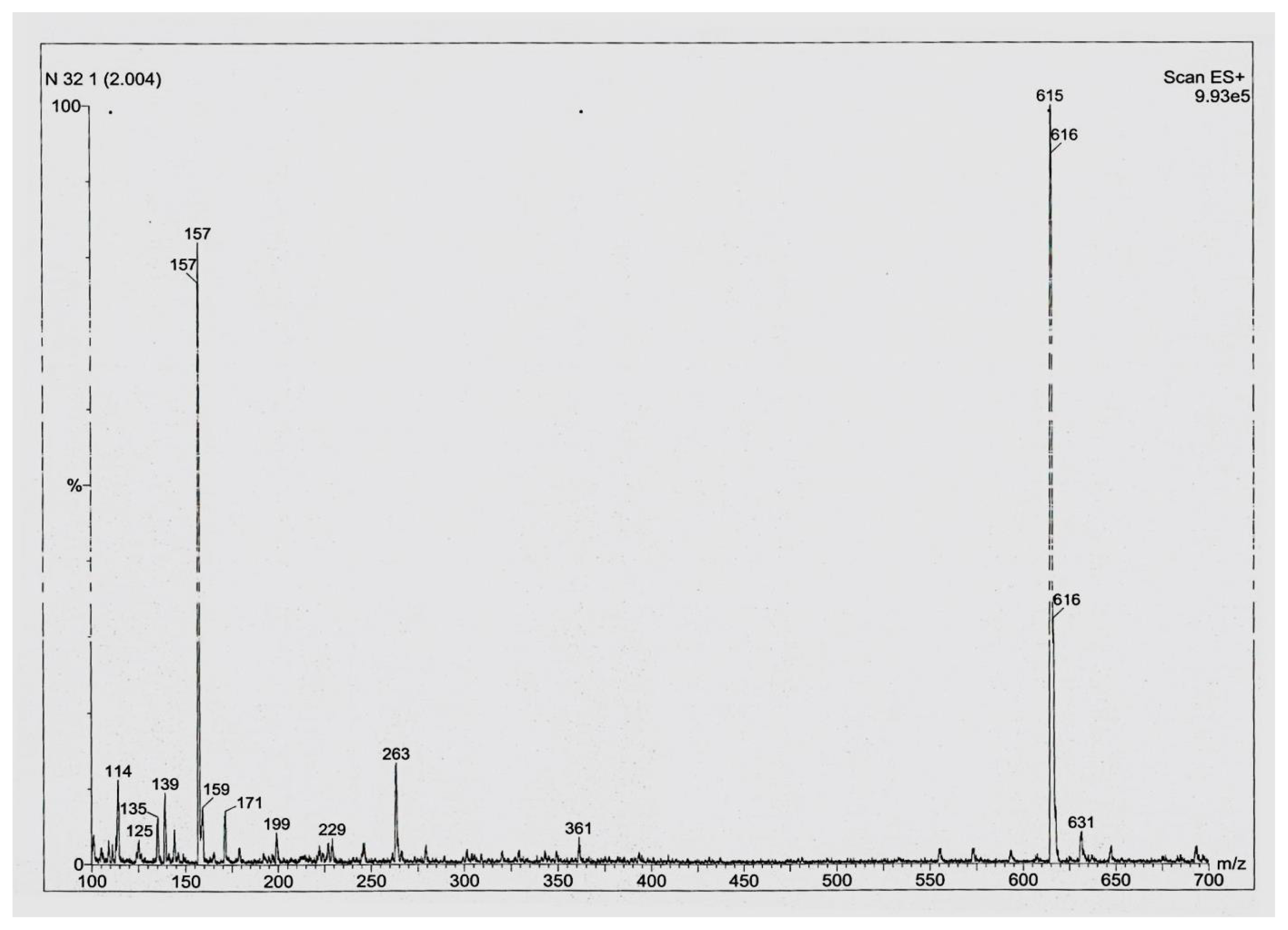

Supplement: Figure S55 — LC-MS/MS spectrum of compound 14. [file turkjchem-47-2-476s55.tif]

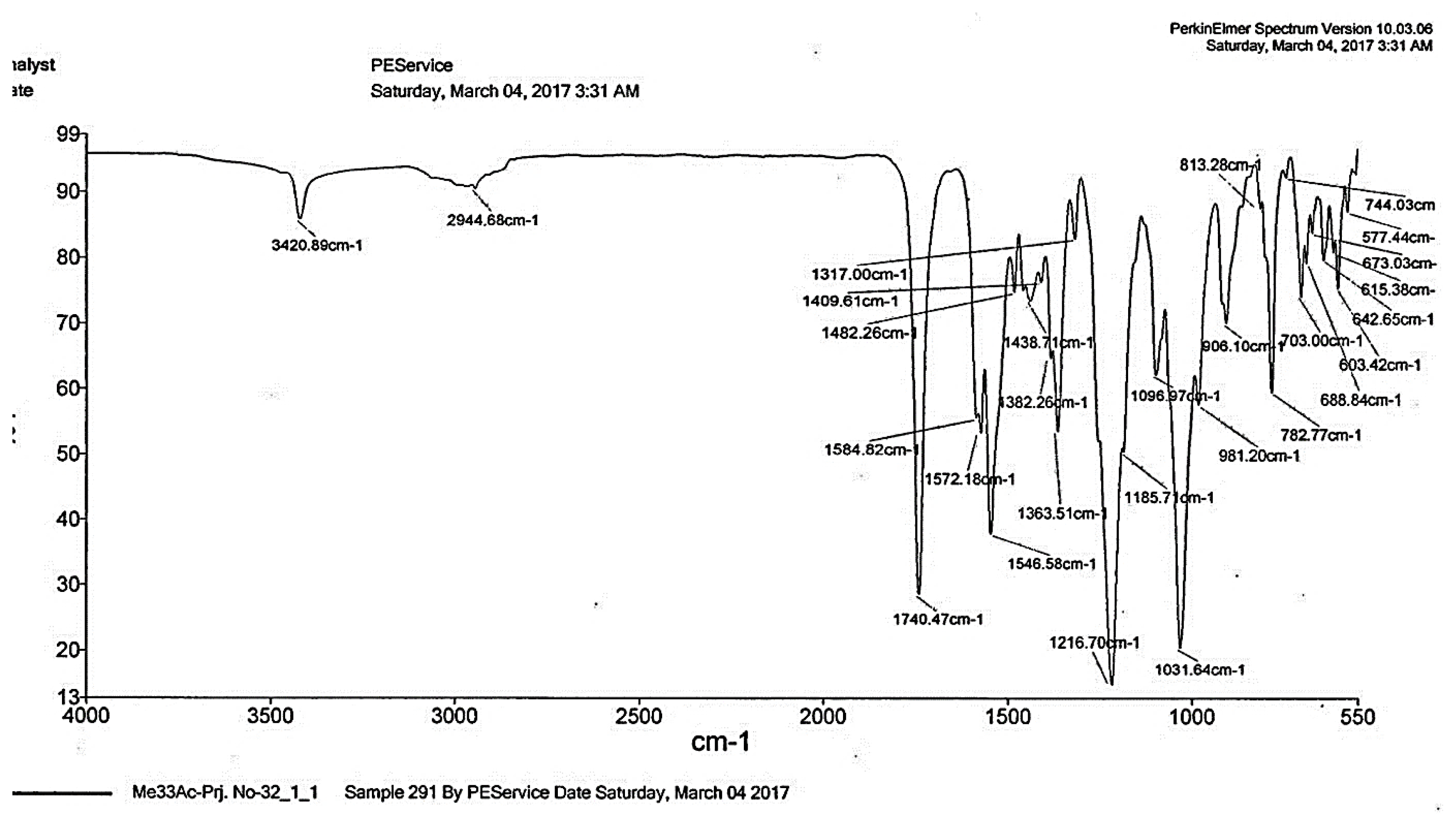

Supplement: Figure S56 — FT-IR spectrum of compound 14. [file turkjchem-47-2-476s56.tif]

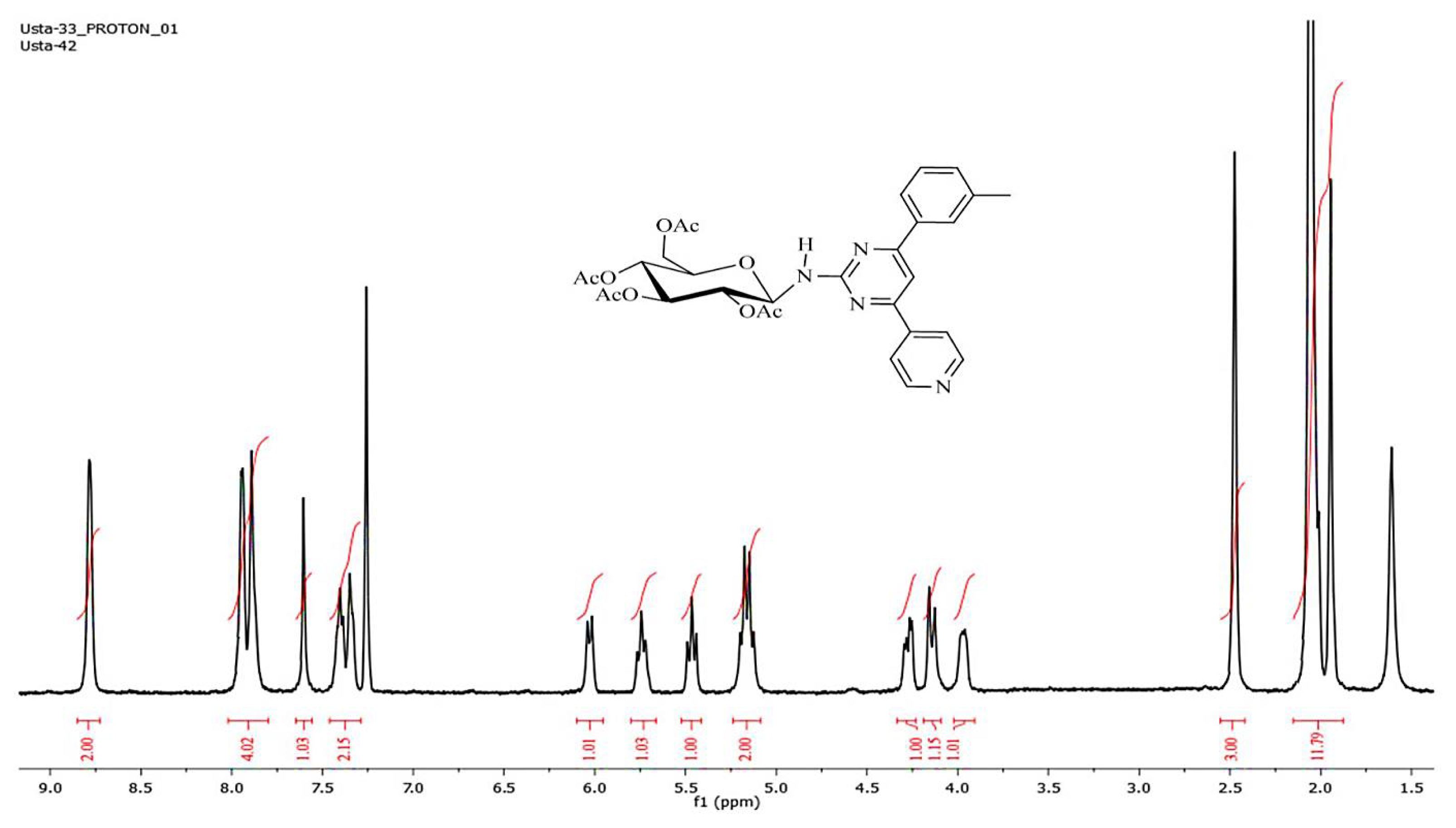

Supplement: Figure S57 — 1H-NMR spectrum of compound 15 (400 MHz, CDCl3, ppm). [file turkjchem-47-2-476s57.tif]

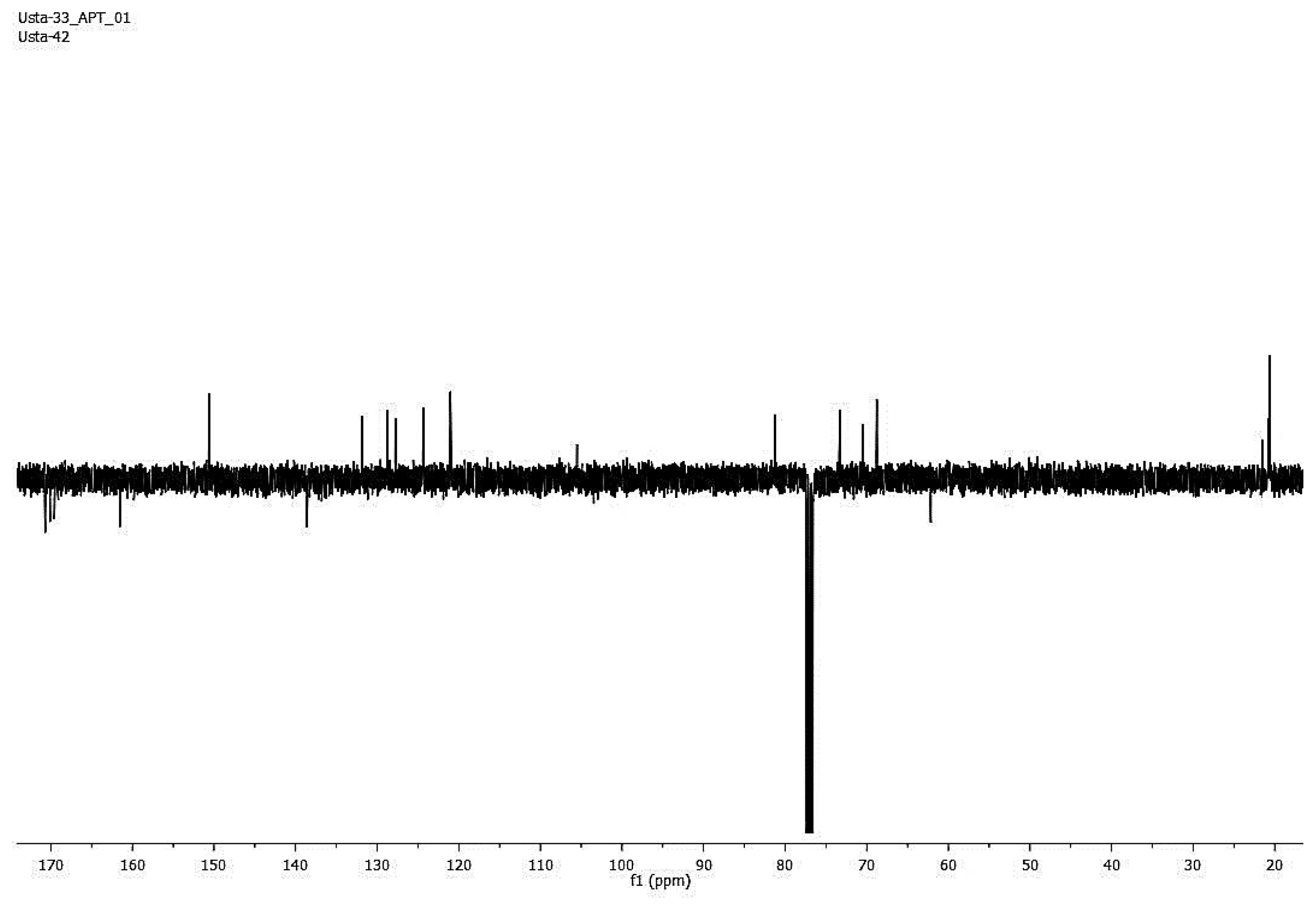

Supplement: Figure S58 — 13C-APT NMR spectrum of compound 15 (100 MHz, CDCl3, ppm). [file turkjchem-47-2-476s58.tif]

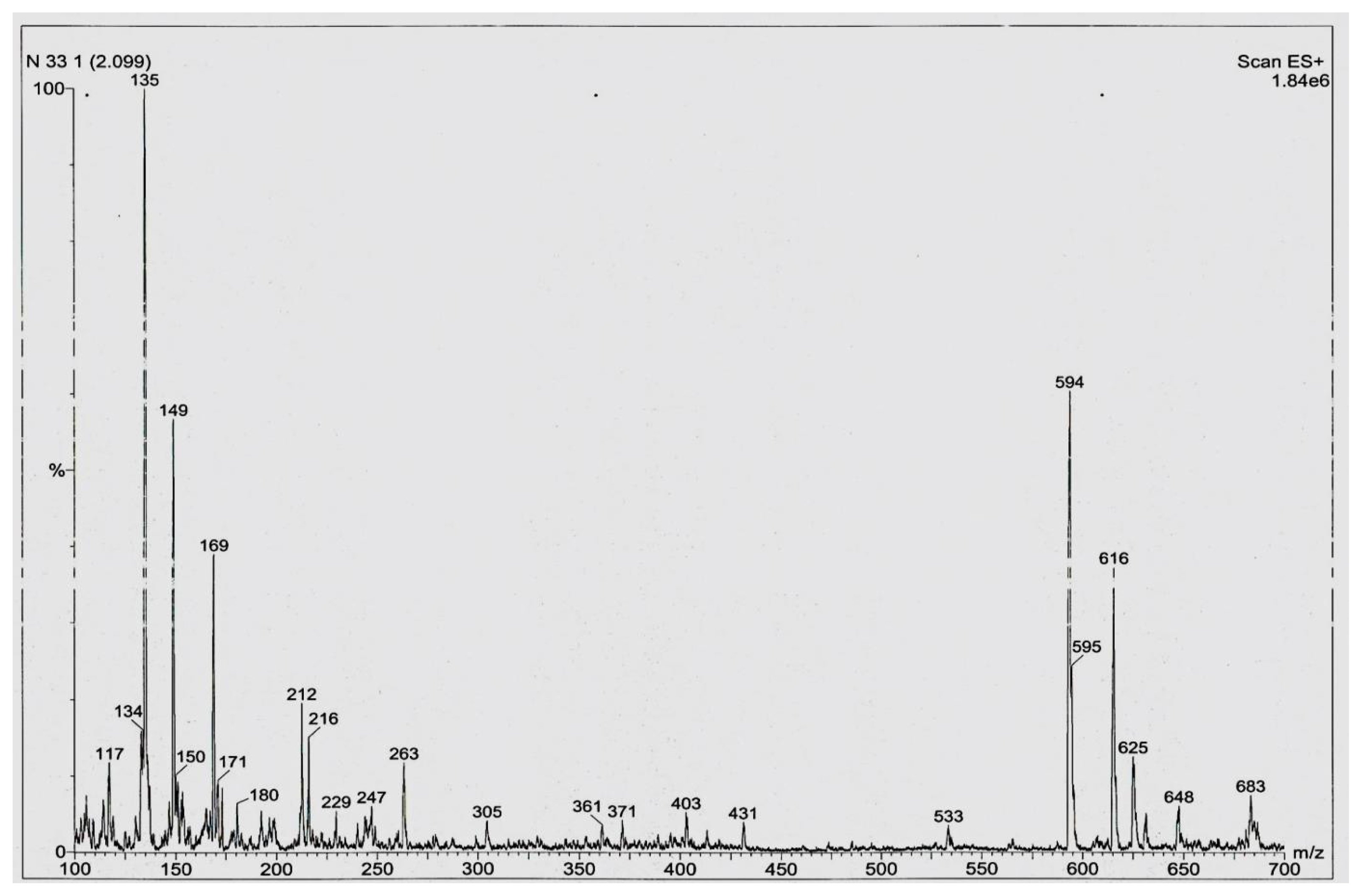

Supplement: Figure S59 — LC-MS/MS spectrum of compound 15. [file turkjchem-47-2-476s59.tif]

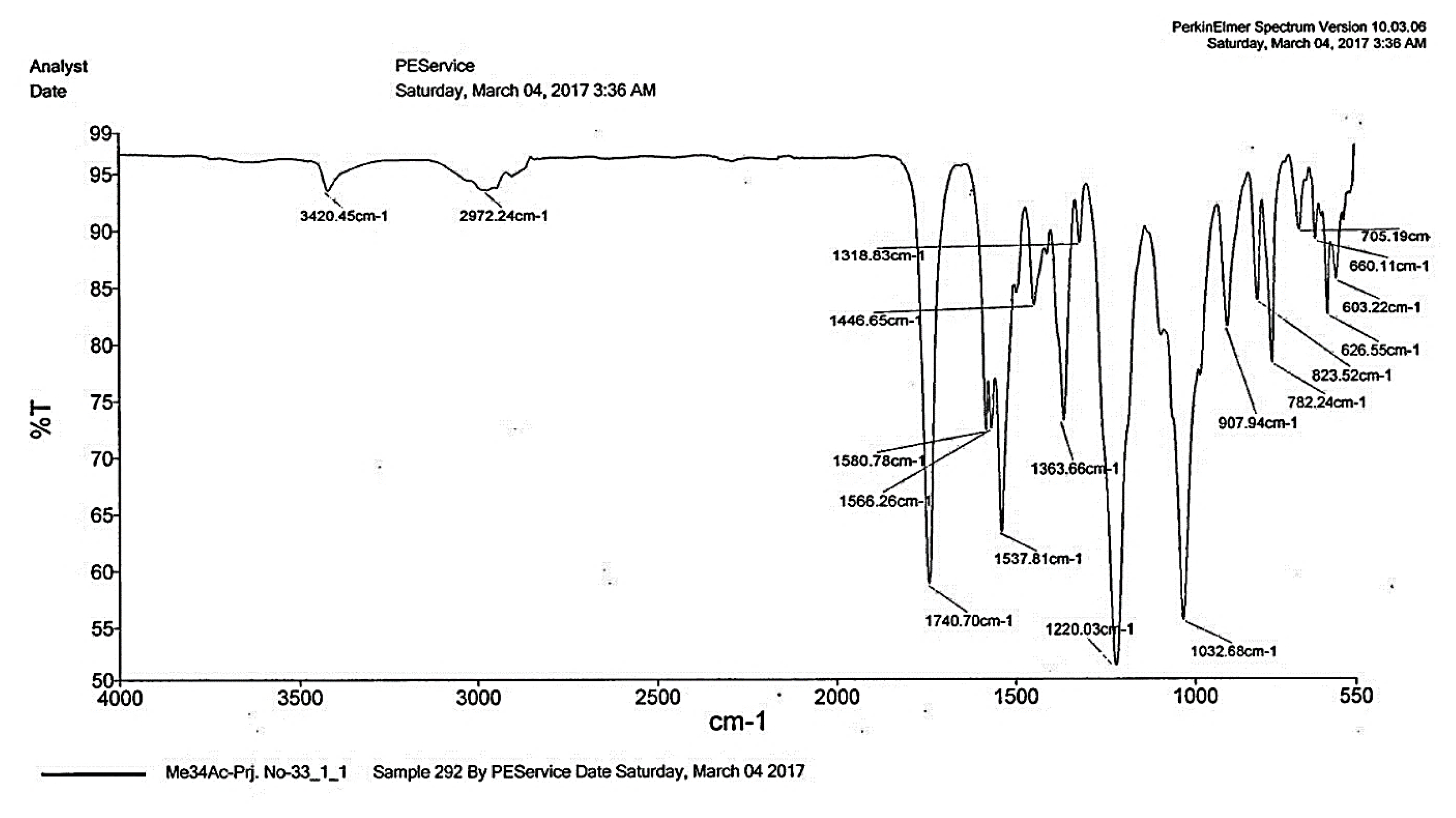

Supplement: Figure S60 — FT-IR spectrum of compound 15. [file turkjchem-47-2-476s60.tif]

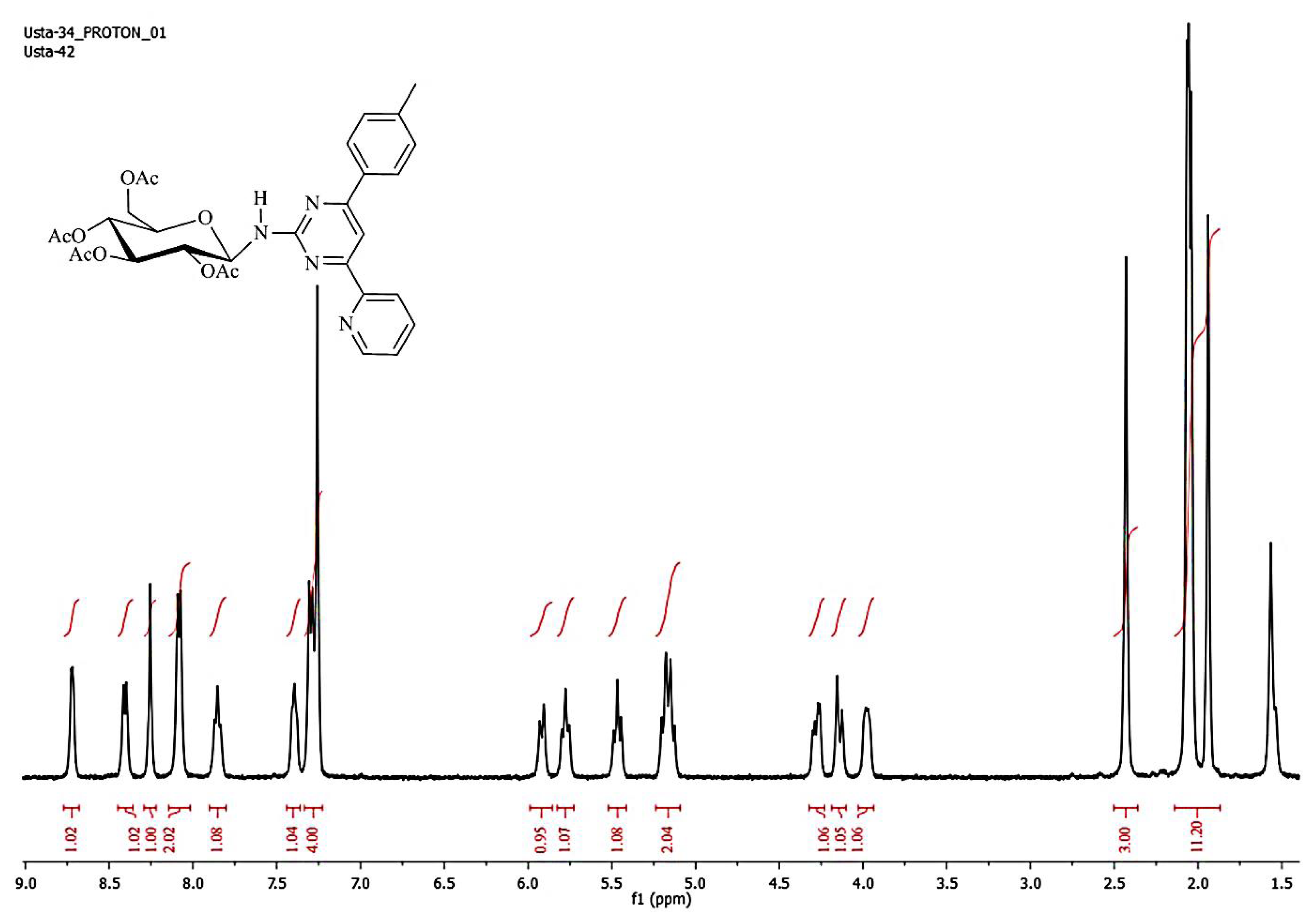

Supplement: Figure S61 — 1H-NMR spectrum of compound 16 (400 MHz, CDCl3/CD3OD (5:1), ppm). [file turkjchem-47-2-476s61.tif]

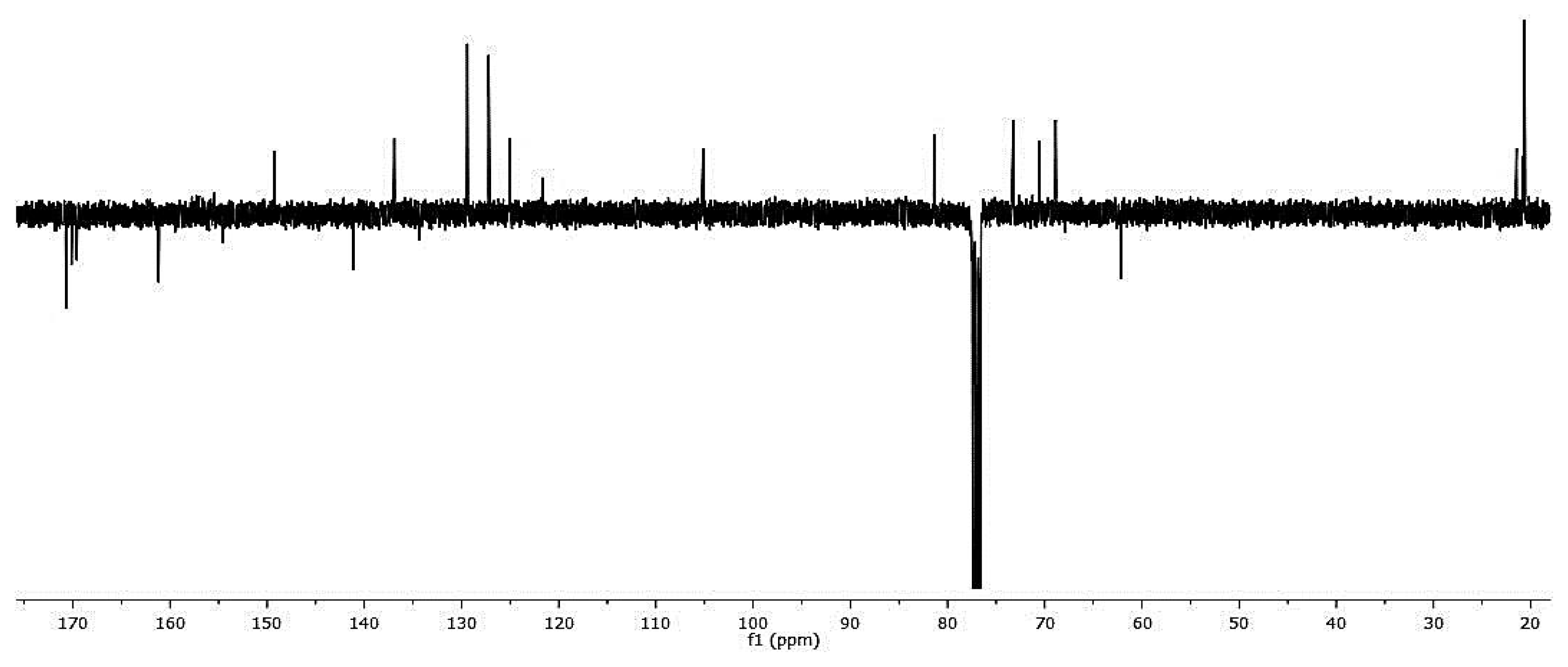

Supplement: Figure S62 — 13C-APT NMR spectrum of compound 16 (100 MHz, CDCl3/CD3OD (5:1), ppm). [file turkjchem-47-2-476s62.tif]

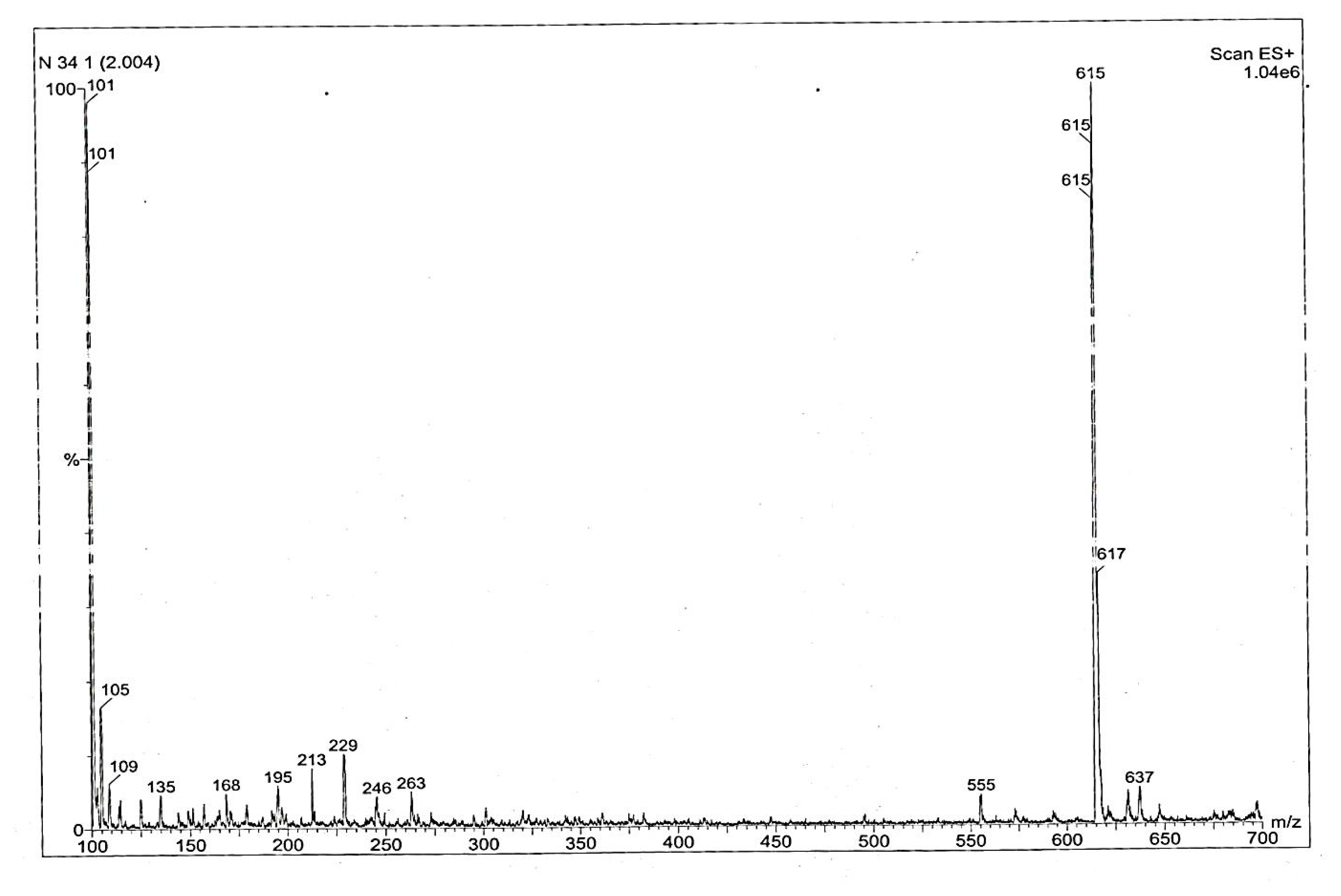

Supplement: Figure S63 — LC-MS/MS spectrum of compound 16. [file turkjchem-47-2-476s63.tif]

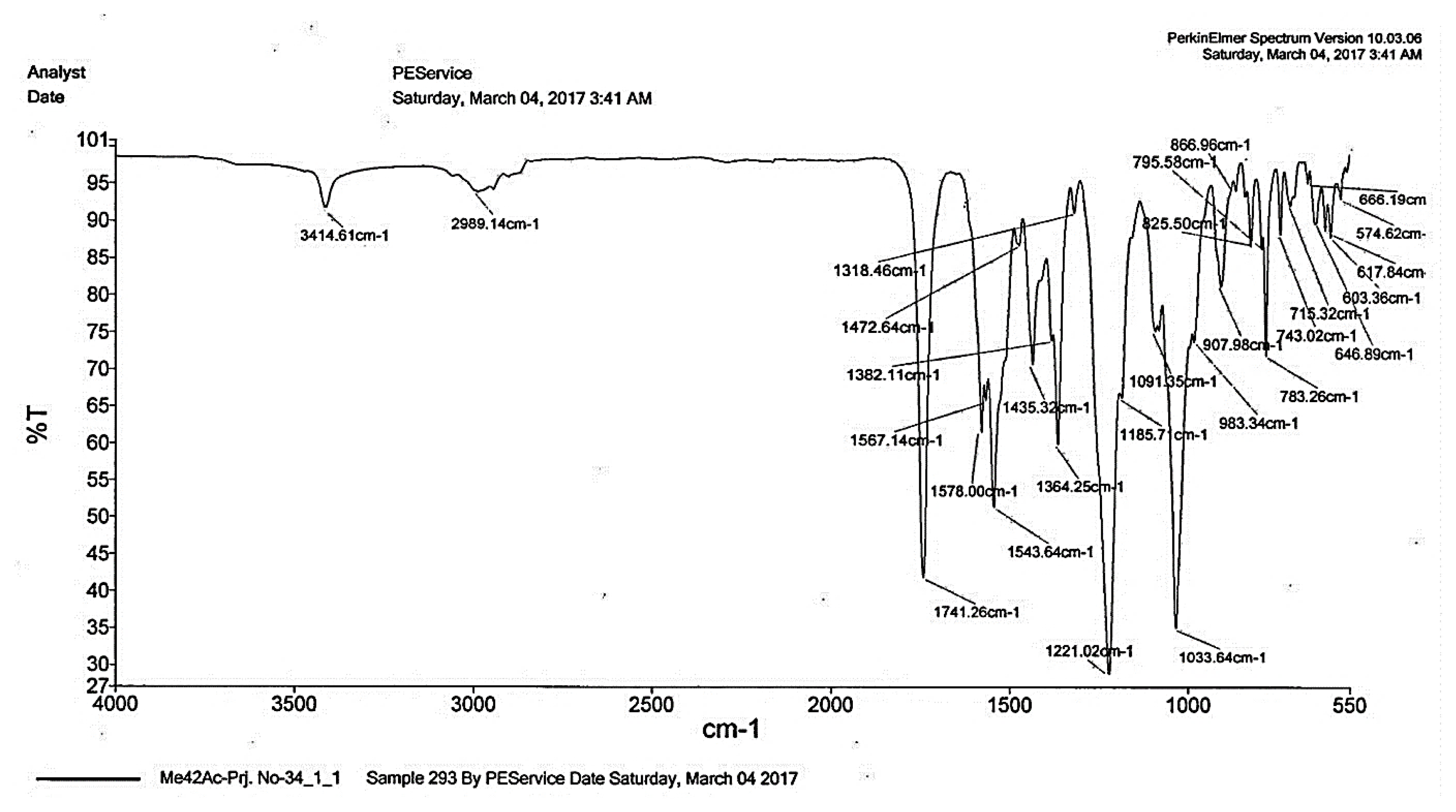

Supplement: Figure S64 — FT-IR spectrum of compound 16. [file turkjchem-47-2-476s64.tif]

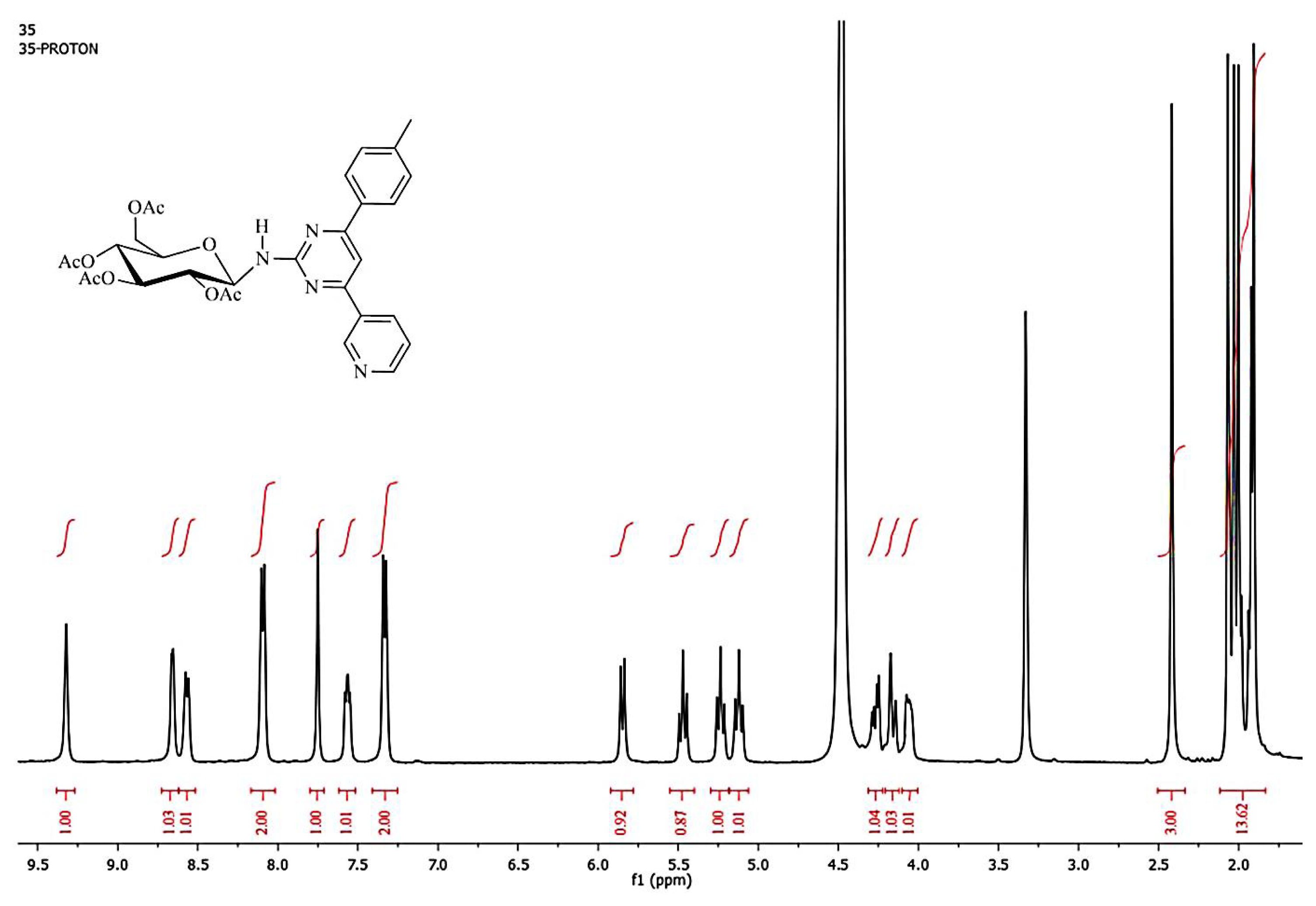

Supplement: Figure S65 — 1H-NMR spectrum of compound 17 (400 MHz, CDCl3/CD3OD (5:1), ppm). [file turkjchem-47-2-476s65.tif]

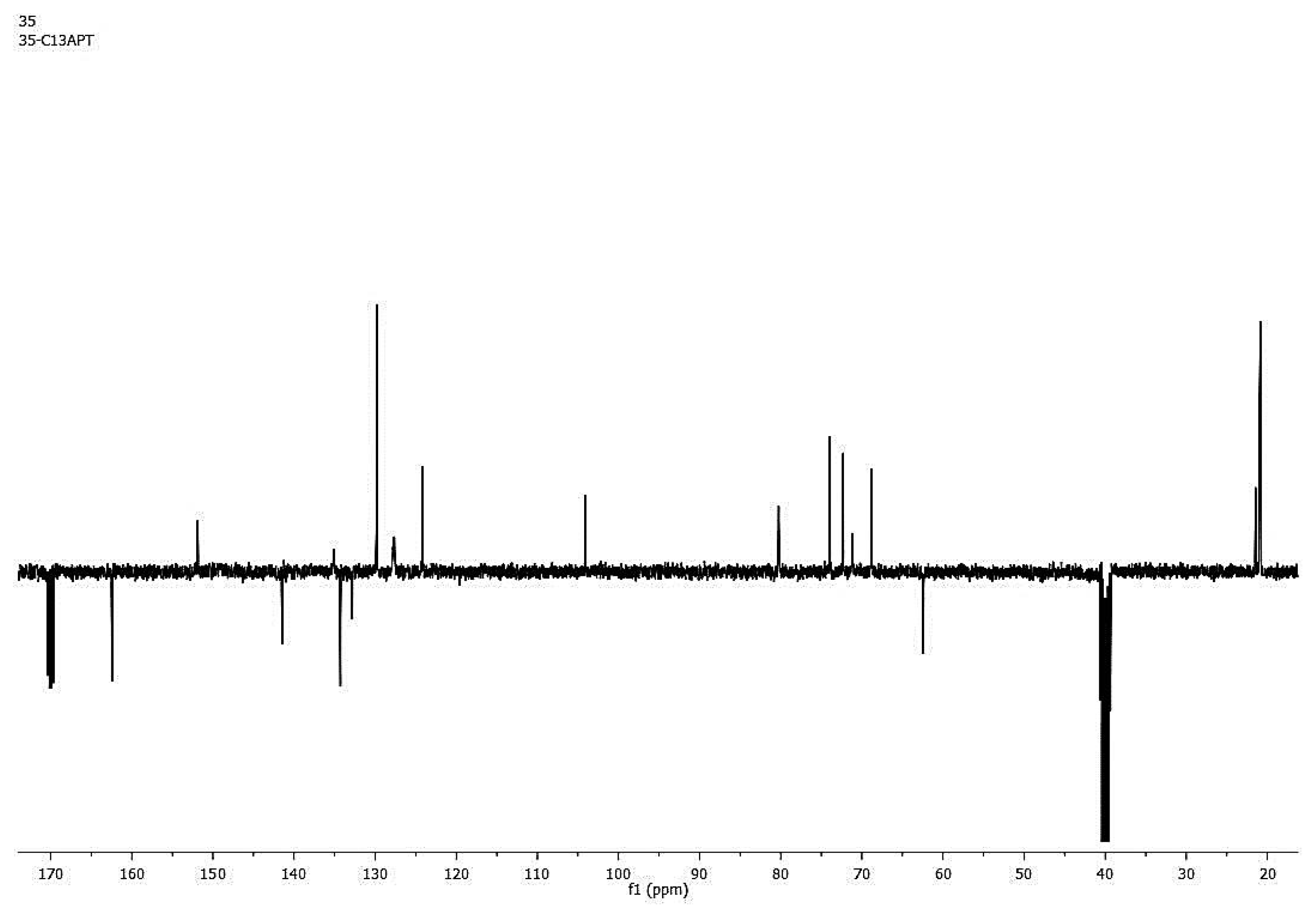

Supplement: Figure S66 — 13C-APT NMR spectrum of compound 17 (100 MHz, CDCl3, ppm). [file turkjchem-47-2-476s66.tif]

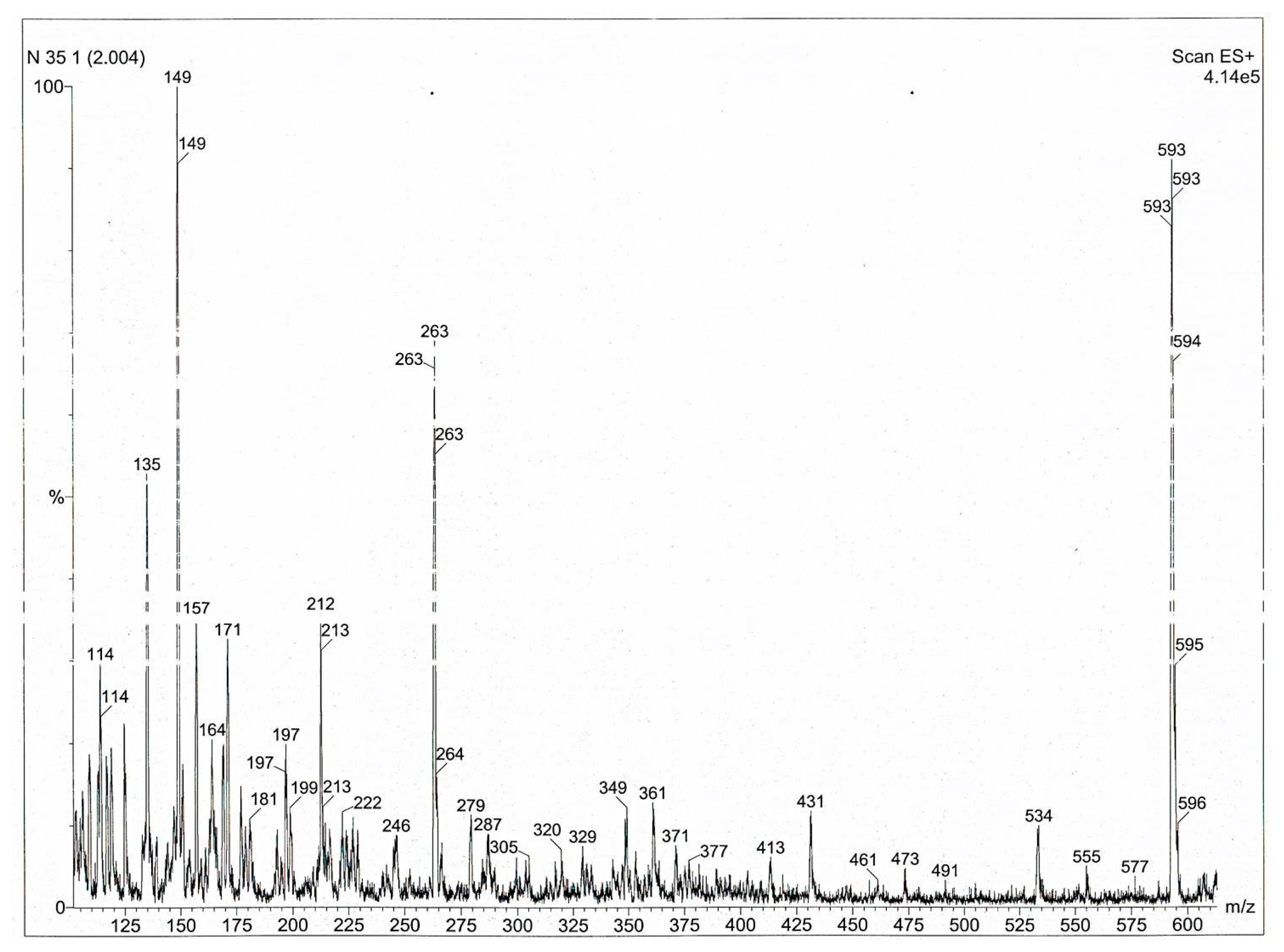

Supplement: Figure S67 — LC-MS/MS spectrum of compound 17. [file turkjchem-47-2-476s67.tif]

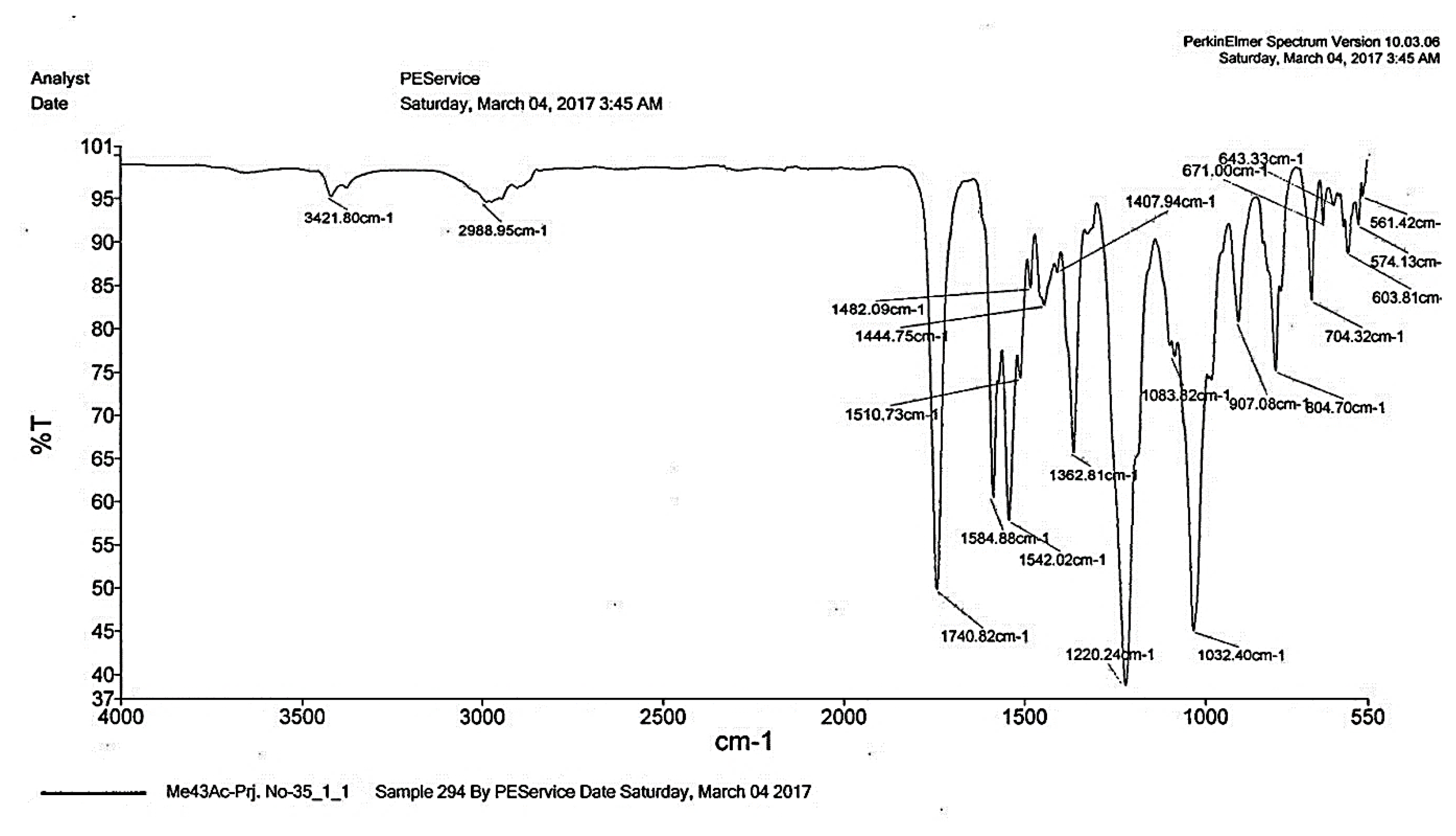

Supplement: Figure S68 — FT-IR spectrum of compound 17. [file turkjchem-47-2-476s68.tif]

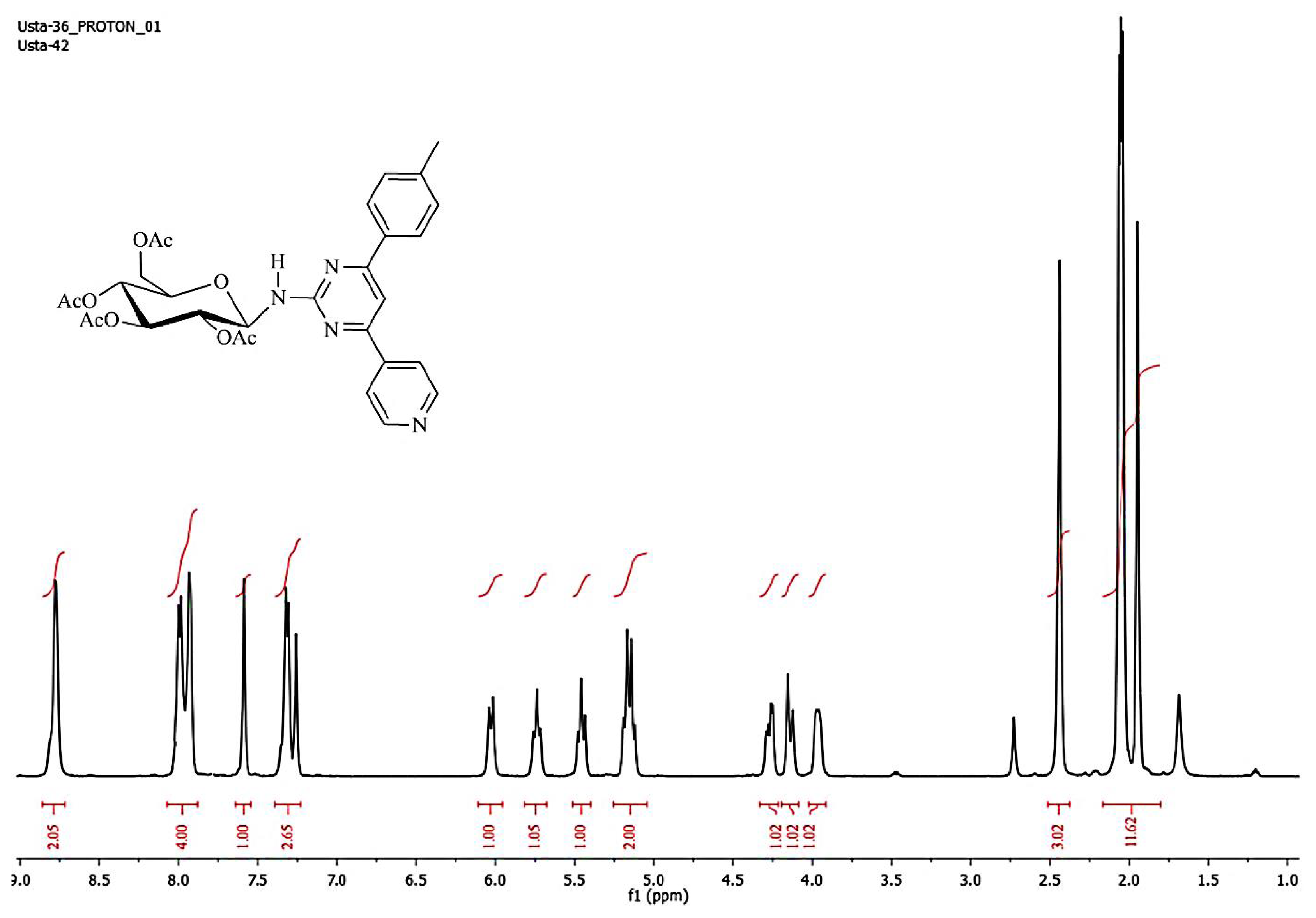

Supplement: Figure S69 — 1H-NMR spectrum of compound 18 (400 MHz, CDCl3, ppm). [file turkjchem-47-2-476s69.tif]

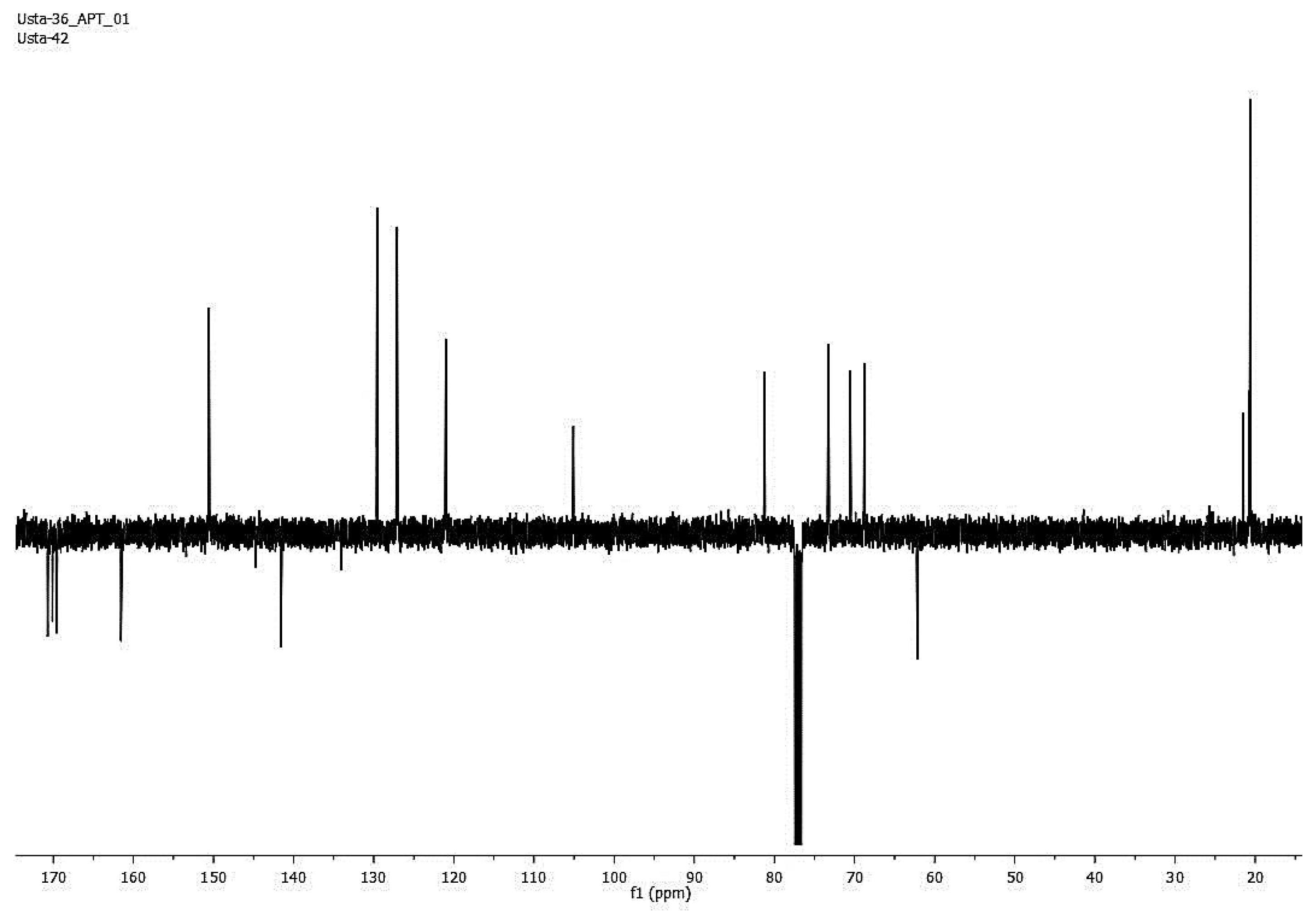

Supplement: Figure S70 — 13C-APT NMR spectrum of compound 18 (100 MHz, CDCl3, ppm). [file turkjchem-47-2-476s70.tif]

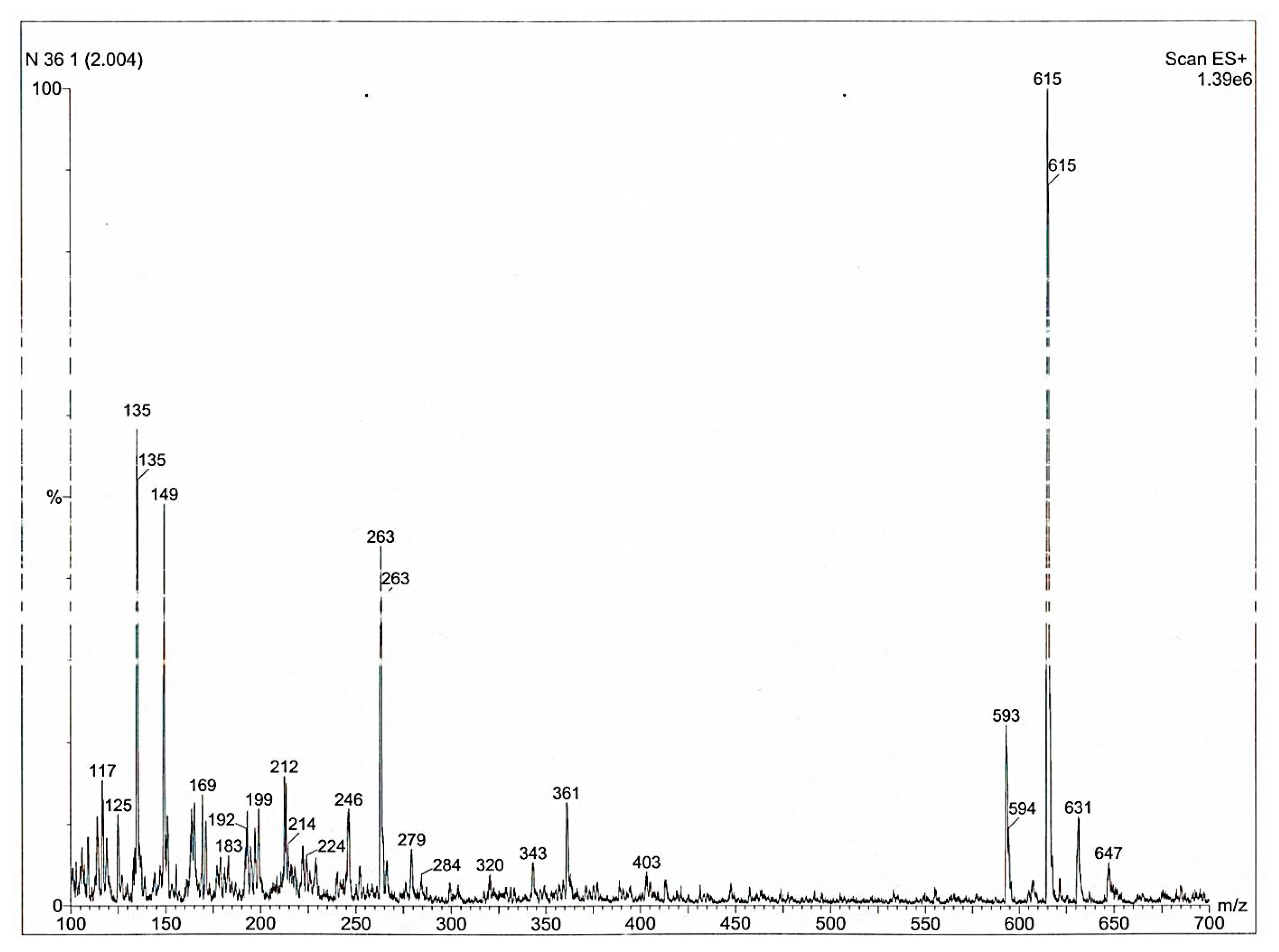

Supplement: Figure S71 — LC-MS/MS spectrum of compound 18. [file turkjchem-47-2-476s71.tif]

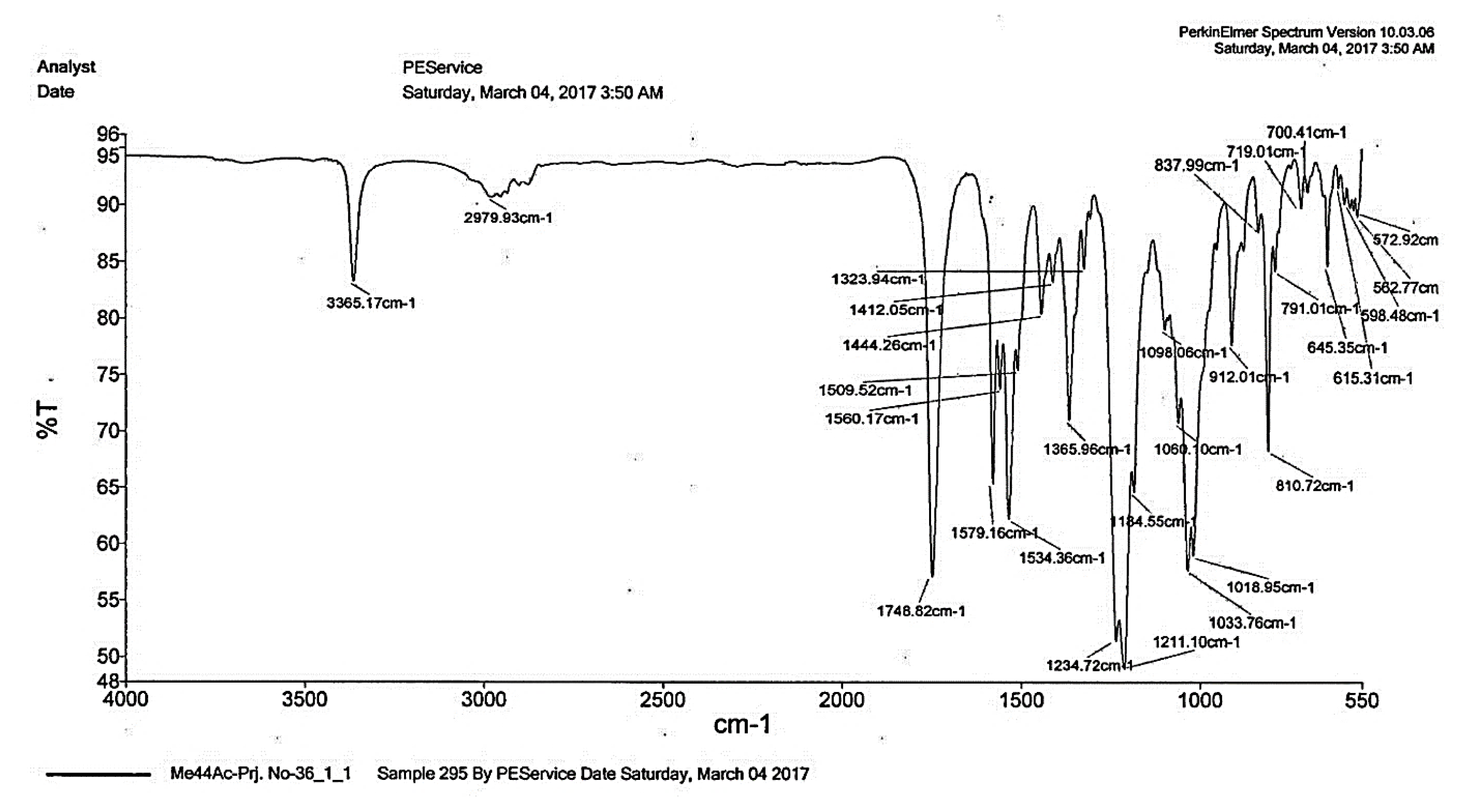

Supplement: Figure S72 — FT-IR spectrum of compound 18. [file turkjchem-47-2-476s72.tif]

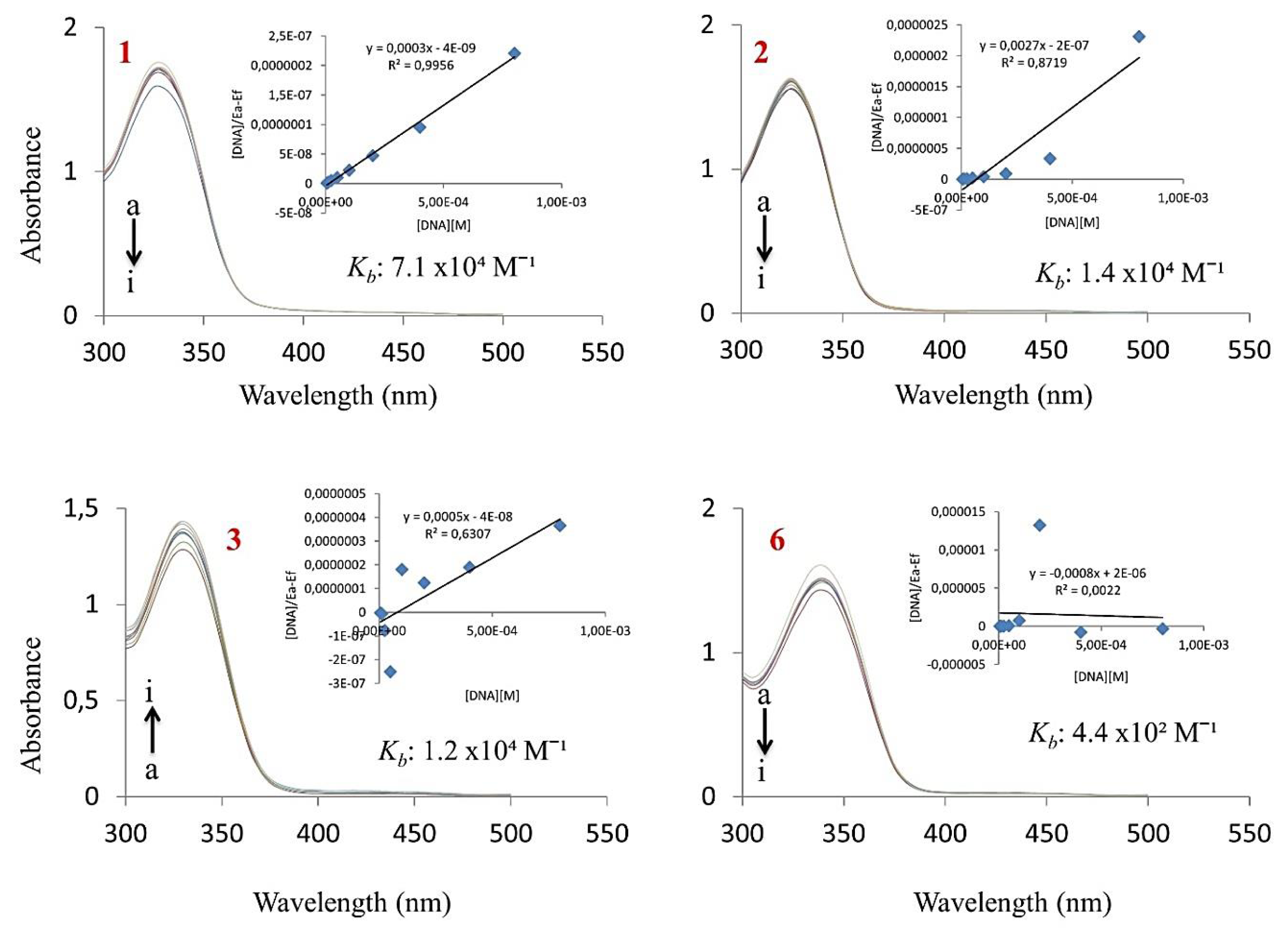

Supplement: Figure S73 — UV–Visible absorption spectra of 25 μM these compounds in the absence (a) and presence of 6.25 μM (b), 12.5 μM (c), 25 μM (d) 50 μM (e), 100 μM (f), 200 μM (g), 400 μM (h), and 800 μM (i) DNA. Note: The direction of arrow demonstrates increasing concentrations of DNA. Inside graph is the plot of [DNA] versus [DNA]/ɛa – ɛf to find the binding constant of complex–DNA adduct. [file turkjchem-47-2-476s73a.tif]

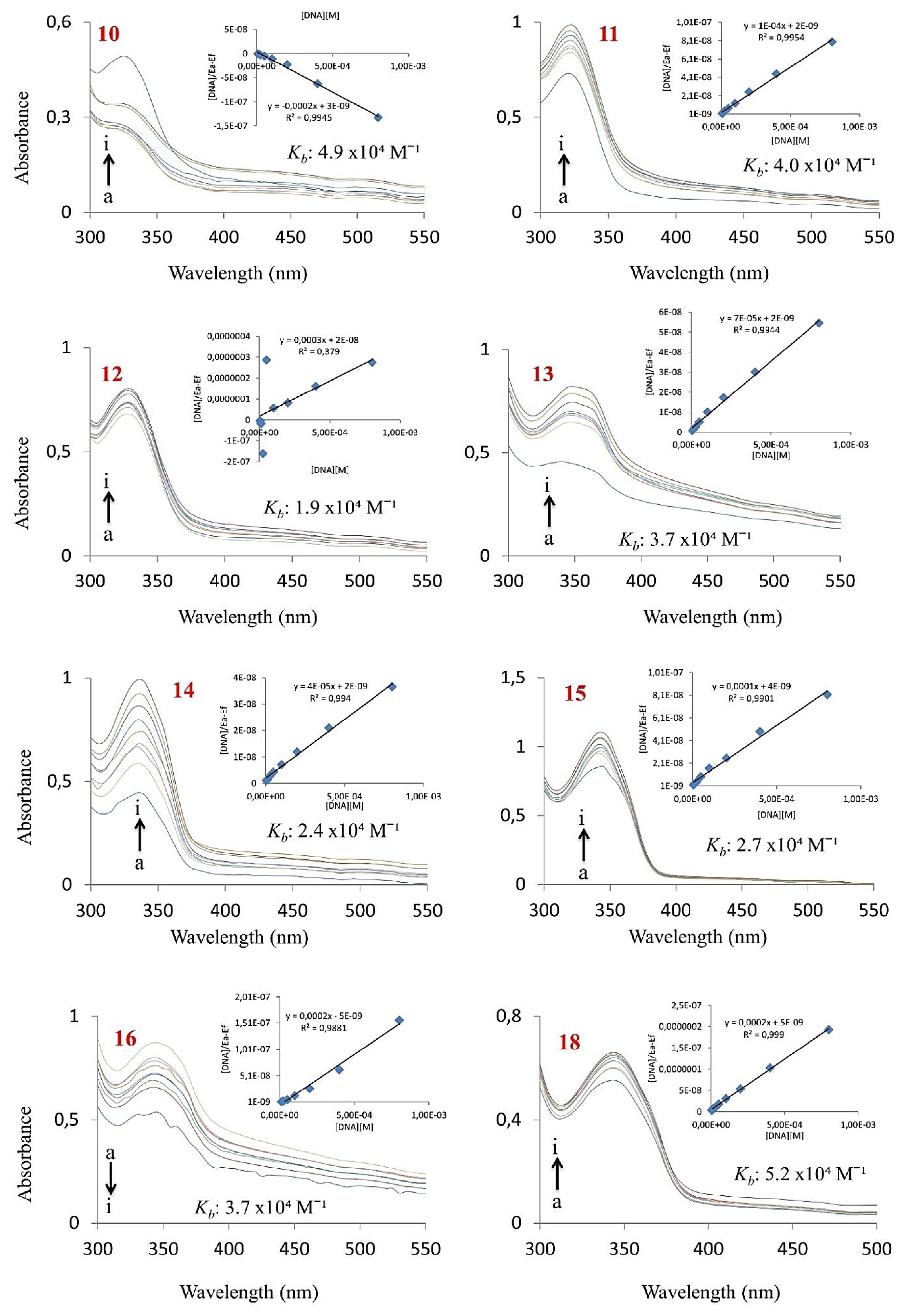

Supplement: Figure S73 — UV–Visible absorption spectra of 25 μM these compounds in the absence (a) and presence of 6.25 μM (b), 12.5 μM (c), 25 μM (d) 50 μM (e), 100 μM (f), 200 μM (g), 400 μM (h), and 800 μM (i) DNA. Note: The direction of arrow demonstrates increasing concentrations of DNA. Inside graph is the plot of [DNA] versus [DNA]/ɛa – ɛf to find the binding constant of complex–DNA adduct. [file turkjchem-47-2-476s73b.tif]

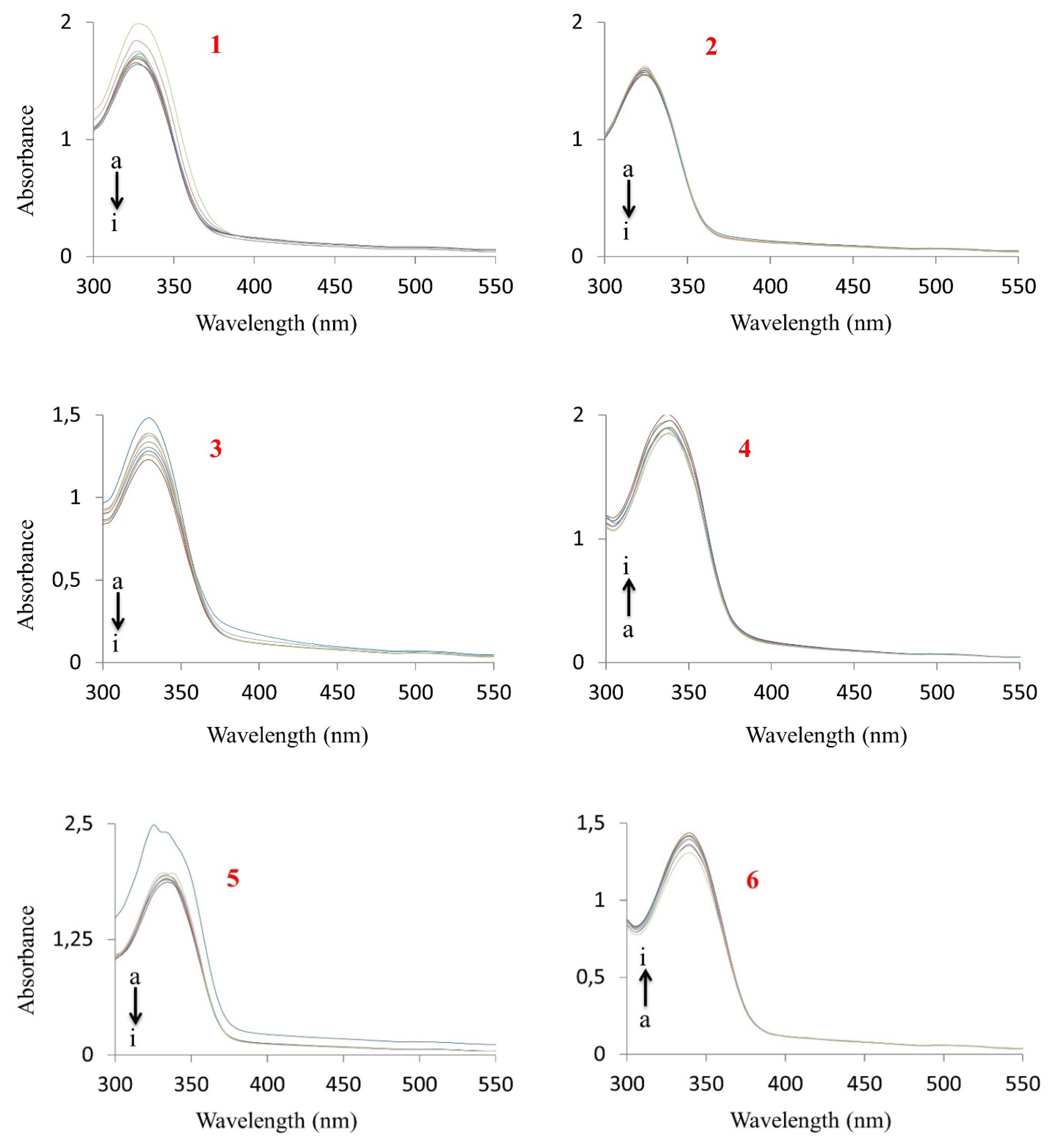

Supplement: Figure S74 — UV–Visible absorption spectra of 25 μM these compounds in the absence (a) and presence of 6.25 μM (b), 12.5 μM (c), 25 μM (d) 50 μM (e), 100 μM (f), 200 μM (g), 400 μM (h) and 800 μM (i) BSA. [file turkjchem-47-2-476s74a.tif]

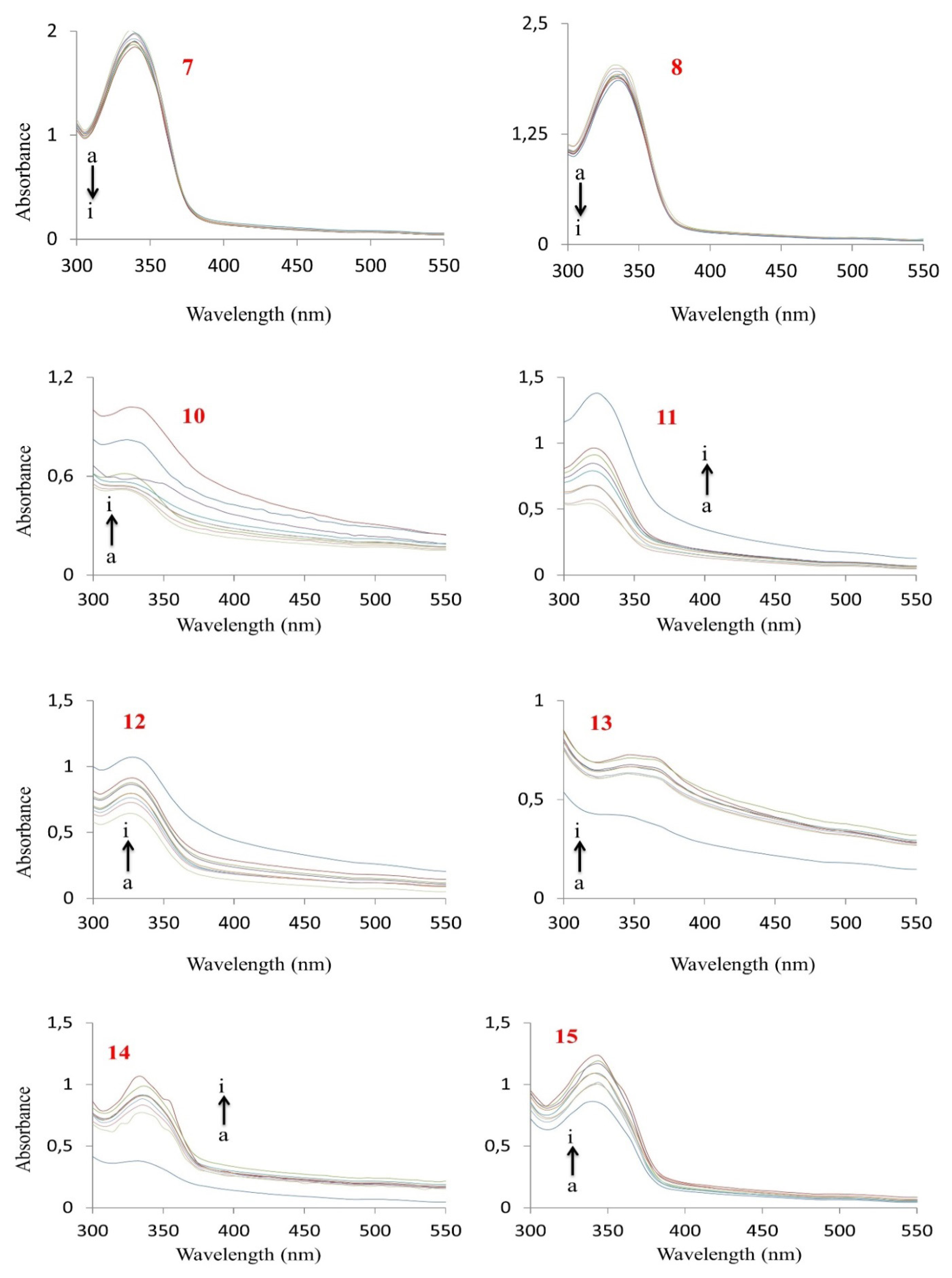

Supplement: Figure S74 — UV–Visible absorption spectra of 25 μM these compounds in the absence (a) and presence of 6.25 μM (b), 12.5 μM (c), 25 μM (d) 50 μM (e), 100 μM (f), 200 μM (g), 400 μM (h) and 800 μM (i) BSA. [file turkjchem-47-2-476s74b.tif]

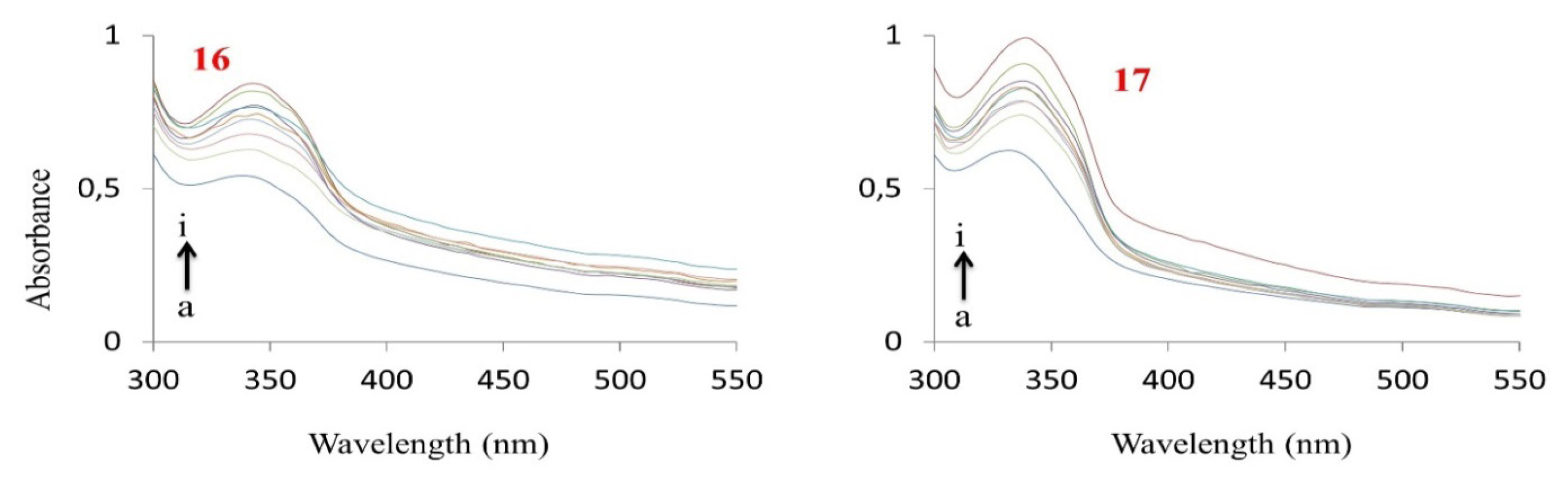

Supplement: Figure S74 — UV–Visible absorption spectra of 25 μM these compounds in the absence (a) and presence of 6.25 μM (b), 12.5 μM (c), 25 μM (d) 50 μM (e), 100 μM (f), 200 μM (g), 400 μM (h) and 800 μM (i) BSA. [file turkjchem-47-2-476s74c.tif]
